# Machine learning‐based multi‐omics models for diagnostic classification and risk stratification in diabetic kidney disease

**DOI:** 10.1002/ctm2.70133

**Published:** 2025-01-08

**Authors:** Xian Shao, Suhua Gao, Pufei Bai, Qian Yang, Yao Lin, Mingzhen Pang, Weixi Wu, Lihua Wang, Ying Li, Saijun Zhou, Hongyan Liu, Pei Yu

**Affiliations:** ^1^ NHC Key Laboratory of Hormones and Development Chu Hsien‐I Memorial Hospital and Tianjin Institute of Endocrinology, Tianjin Medical University Tianjin China; ^2^ Tianjin Key Laboratory of Metabolic Diseases Tianjin Medical University Tianjin China; ^3^ Division of Nephrology, National Clinical Research Center for Kidney Disease, State Key Laboratory of Organ Failure Research Nanfang Hospital, Southern Medical University Guangzhou China; ^4^ Department of Nephrology & Blood Purification Center The Second Hospital of Tianjin Medical University Tianjin China

Dear Editor,

The global prevalence of chronic kidney disease (CKD) is about 10%.^1^ Diabetic kidney disease (DKD) has emerged as the leading cause of end‐stage renal failure.^2^ Early identification of DKD is important for improving the survival rate and improving the quality of life. However, the preclinical stages of DKD may lack obvious symptoms and non‐invasive biomarkers.^3^ Through blood lipidomics, urine proteomics and metabolomics technologies, potential DKD markers are identified to establish an accurate early warning model for DKD. We aim to provide effective tools for the individualised prevention of DKD, and help to explain the associations between different molecules and their risk of DKD from multiple perspectives. The methods of study are shown in Appendix 1.

Figure 1 illustrates the overview of the common and unique changes in proteomics pathways observed at various stages of DKD. The pathways reflected the active biological processes closely related to multi‐omics during the development of DKD. Potential proteomic biomarkers were identified through a multi‐level screening process, with a comprehensive score used to assess their significance (Appendix 1). Finally, CD300LF, CST4, MMRN2, SERPINA14, L‐glutamic acid dimethyl ester (DLG) and phosphatidylcholine (PC) were selected. The results of study are provided in Appendix 2.

**FIGURE 1:**
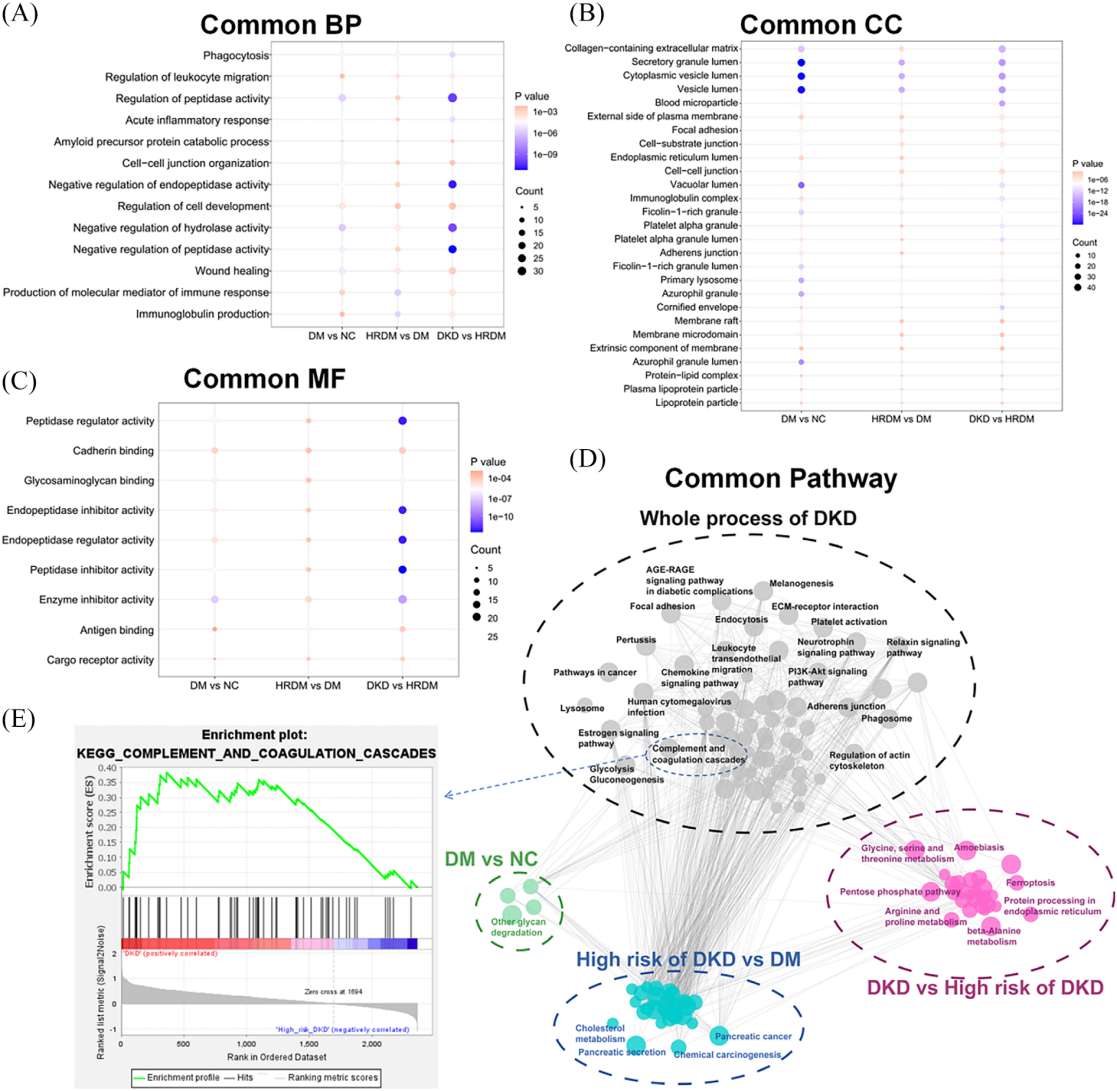
Changes in co‐enrichment and biological pathways across different DKD stages. (A) Changes of common BP in different disease stages; (B) Changes of CC in different disease stages; (C) Changes of MF in different disease stages; (D) Common and unique changes of enrichment pathways in different DKD stages; (E) GSEA enrichment analysis of complement and coagulation cascade pathways. In Figure A, the number of molecules involved in regulating the activity of peptidases, hydrolases and endopeptidases increases significantly in the early stages of DKD. Additionally, Figure B illustrates a reduction in the lumen of granules, vesicles and vesicles responsible for secretion, while an increase is observed in collagen‐containing extracellular matrix (ECM), blood particles and local adhesion molecules. Various cellular components such as the outer plasma membrane, endoplasmic reticulum lumen, ECM, interstitial cells, immunoglobulin complex, platelet α‐granules, protein–lipid complex and plasma lipoprotein granules are implicated in the pathogenicity of DKD from T2DM to DKD. The role of various peptidases and the regulation of endopeptidase activity in the pathogenesis stage of DKD is highlighted in Figures C. Figure D illustrates the involvement of different pathways in DKD progression. Grey represents the overall disease process, green shows the pathway changes in T2DM individuals, blue indicates pathway changes between different risk levels of DKD and purple demonstrates the pathway changes leading to DKD. Figure D outlines the common and unique changes in KEGG pathways at different stages of DKD, with common pathway alterations including complement and coagulation cascade pathways, ECM receptor interactions, platelet activity, actin cytoskeleton regulation, PI3K‐Akt, AGE‐RAGE, Relaxin signalling pathways, glycolysis processes and leukocyte migration across endothelial cells. Figure E presents the GSEA enrichment results of the complement and coagulation cascade pathway, indicating its activation at the critical stage of DKD pathogenesis. Additionally, pathways such as glycine, serine and threonine metabolism, β‐alanine metabolism, iron death, arginine and proline metabolism and the pentose phosphate pathway are implicated in the critical transition process of DKD, underscoring the significance of amino acid metabolism disorders in DKD. BP, biological pathways; CC, cellular components; DKD, diabetic kidney disease; GSEA, Gene Set Enrichment Analysis; KEGG, kyoto encyclopedia of genes and genomes; MF, molecular functions.

The cross‐sectional study included a total of 1500 patients (Figure S2 and Appendix 1). Patients were categorised into four groups: healthy control (HC, 30), type 2 diabetes mellitus (T2DM, 361), high‐risk DKD (HR‐DKD, 555) and DKD group (554). Baseline patient information is detailed in Appendix 2. The patients were categorised into two groups: a training and a test set (3:1). A total of seven prediction models for diagnosis classification were established, with the included indicators provided in Table S17 and Appendix 2. The integration of clinical indicators with multi‐omics indicators resulted in a substantial accuracy improvement (Accuracy = .923 [.893, .947]; Figure 2A–G). This integrated model was the most effective, with improved performance across all metrics, including area under the curve (AUC), sensitivity, specificity and accuracy. Additionally, the study utilised a total of 12 machine learning algorithms, all of which achieved AUC values above .940 (Figure 2H).

**FIGURE 2:**
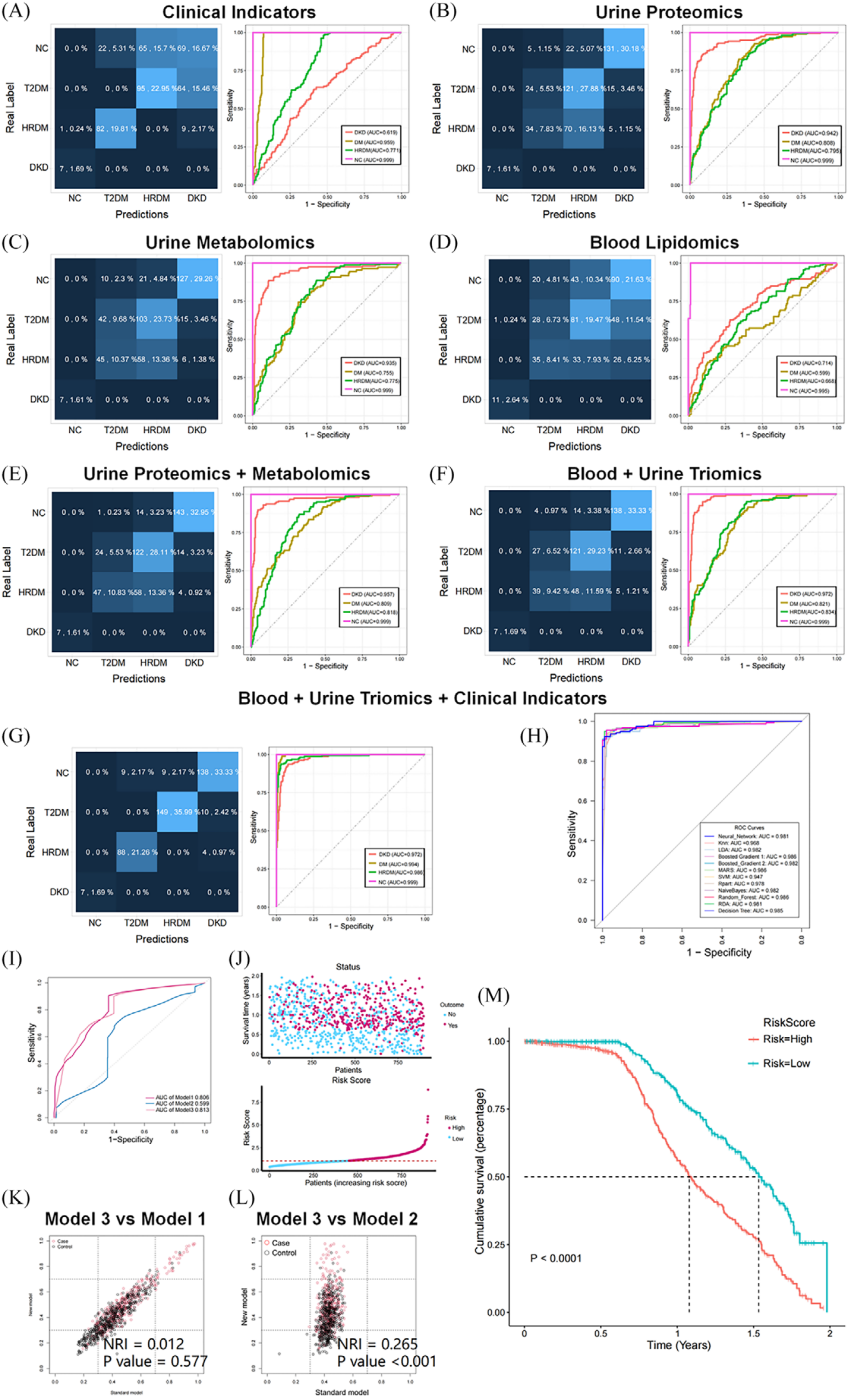
Classification and risk‐prognostic prediction models for diabetic kidney disease (DKD). (A) Clinical indicators. AUC: NC = .999, T2DM = .959, HRDKD = .771, DKD = .619. (B) Urine proteomic biomarkers. AUC: NC = .999, T2DM = .808, HRDKD = .795, DKD = .942. (C) Urine metabolomic biomarkers. AUC: NC = .999, T2DM = .755, HRDKD = .775, DKD = .935. (D) Blood lipidomic biomarkers. AUC: NC = .995, T2DM = .559, HRDKD = .668, DKD = .714. (E) Urine proteomic and metabolomic biomarkers. AUC: NC = .999, T2DM = .809, HRDKD = .818, DKD = .957. (F) Blood and urine triomics biomarkers. AUC: NC = .999, T2DM = .821, HRDKD = .834, DKD = .972. (G) Integrated model with clinical indicators, blood and urine triomics biomarkers. AUC: NC = .999, T2DM = .994, HRDKD = .986, DKD = .972. (H) Twelve machine learning algorithms‐based ROC curves for the integrated model. (I) ROC curves; (J) Risk score joint plot; (K) NRI of Model 3 compared with Model 1; (L) NRI index of Model 3 compared with Model 2; (M) KM survival curves for high‐risk and low‐risk populations. AUC, area under the curve; DM, diabetes mellitus; HC, healthy control; HR‐DKD, high‐risk diabetic kidney disease; KM, Kaplan–Meier; NRI, net reclassification improvement; RF, random forest; ROC, receiver operating characteristic.

The prospective cohort study involved 919 patients, with a median follow‐up duration of 1.07 years. Based on the clinical and multi‐omics indicators, three risk‐prognostic prediction models were developed: the biomarker model (Model 1), clinical indicators model (Model 2) and integrated model (Model 3). The specific indicators used are detailed in Table S22 and Appendix 2. Figure 2I displays the AUC curves of these models, with Model 3 achieving the highest AUC of .813. A risk score was calculated using Model 3 (score cut‐off = 1.06; Figure 2J). In Figure 2K,L, we compare the predictive performance of the two models using the net reclassification improvement (NRI) index. Furthermore, Figure 2M shows that high‐risk patients had higher risk of composite endpoint events (*p* < .001). The correlation analysis is shown in Figure 3A. The analysis revealed that all six biomarkers showed significant associations with the risk of DKD composite events (*p* < .001, Figure 3B–G).

**FIGURE 3:**
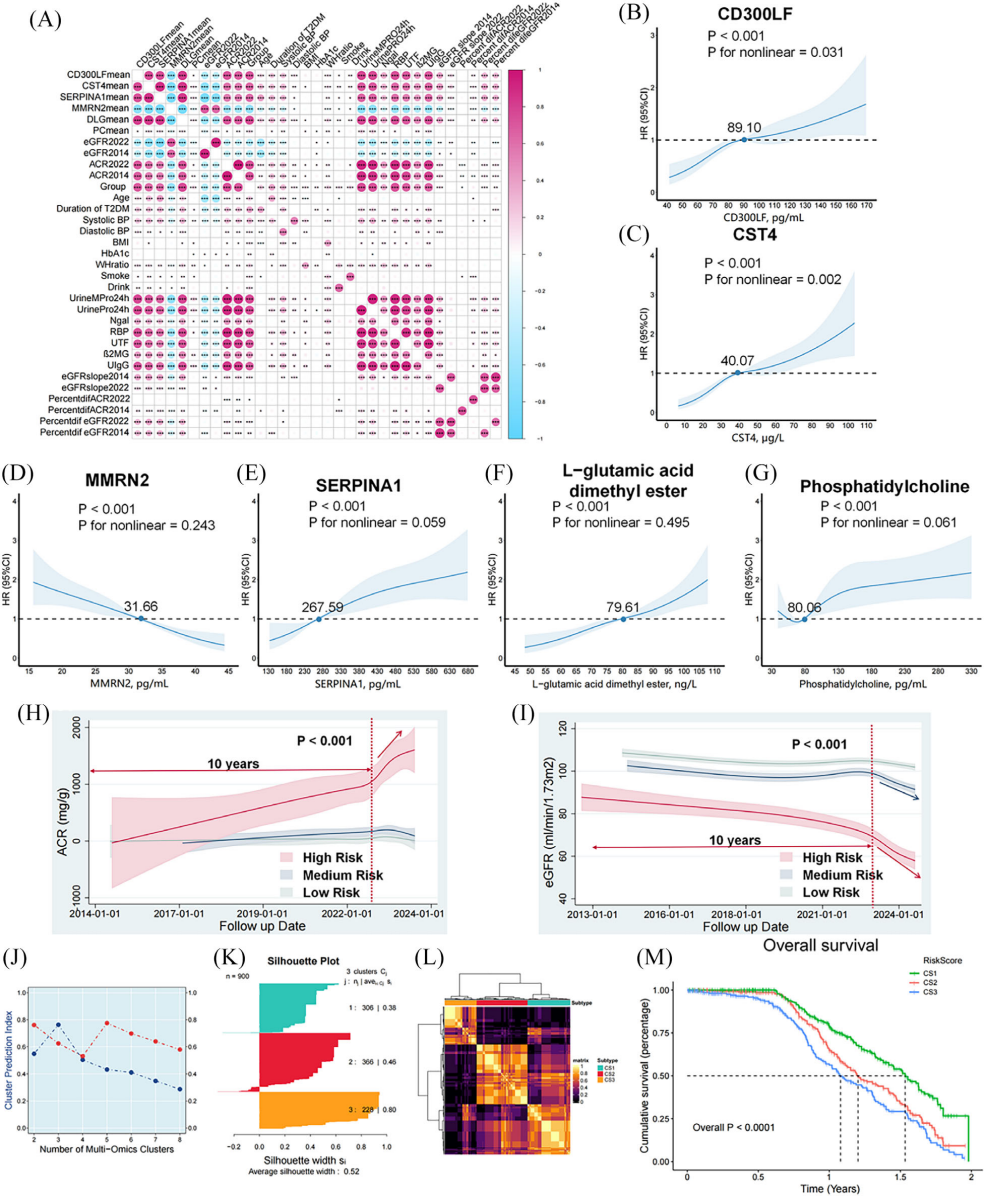
Identification of high‐risk molecular subtypes of DKD based on multi‐omics COX risk score and integrated clustering algorithms. (A) Correlation analysis; (B) RCS plot for CD300LF (reference point = 89.10); (C) RCS for CST4 (reference point = 40.07); (D) RCS plot for MMRN2 (reference point = 31.66); (E) RCS plot for SERPINA1 (reference point = 267.59); (F) RCS plot for DLG (reference point = 79.61); (G) RCS plot for PC (reference point = 80.06). (H) Trajectories of dynamic changes in ACR over 10 years based on the multi‐omics COX prognostic risk score; (I) Trajectories of dynamic changes in eGFR over 10 years based on the multi‐omics COX prognostic risk score; (J) Optimal number of subtypes based on the MOVICS multi‐omics integrated clustering algorithm; (K) Profile coefficients for three molecular subtypes (CS1, CS2, CS3); (L) Typing heatmap based on the MOVICS multi‐omics integrated clustering algorithm; (M) KM survival analysis based on three molecular subtypes. CD300LFmean, mean value of CD300LF; CST4mean, the mean value of CST4; DLG, L‐glutamic acid dimethyl ester; DLGmean, the mean value of DLG; eGFR2014 and ACR2014, the patient's earliest tested eGFR and ACR values; eGFR2022 and ACR2022, the baseline eGFR and ACR values; eGFRslope2014 and PercentdifeGFR2014, the annual rate and percentage of decline in eGFR for patients from 2014 until the follow‐up endpoint; eGFRslope2022 and PercentdifeGFR2022, the annual rate and percentage of decline in eGFR for patients from 2022 until the follow‐up endpoint; MMRN2mean, the mean value of MMRN2; PC, phosphatidylcholine; PCmean, the mean value of PC; PercentdifACFR2022, the annual rate and percentage of decline in eGFR for patients from 2022 until the follow‐up endpoint; PercentdifACR2014, the percentage decrease in ACR from 2014 to the follow‐up endpoint; PercentdifACR2022, the percentage decrease in ACR from 2022 to the follow‐up endpoint; RCS, restricted cubic spline; SERPINA1mean, the mean value of SERPINA1; UrineMPro24h quantifies urinary 24‐h microalbumin levels, while UrinePro24h quantifies urinary 24‐h albumin levels. The urine damage indicators include Ngal (N.acetylamino‐glucosidase), RBP (retinol‐binding protein), UTF (urinary transferrin quantification), β2MG (urinary β2 microglobulin) and UIgG (immunoglobulin G). ACR, albumin to creatinine ratio; eGFR, estimated glomerular filtration rate.

The Cox risk score was calculated using the multi‐omics risk prediction model (Model 3). Subsequently, the patients were classified into low‐, medium‐ and high‐risk categories. In Figure 3H–J, the high‐risk group exhibited a continuous increase in albumin to creatinine ratio (ACR) and decline in estimated glomerular filtration rate (eGFR) over 10 years, particularly steep around the 10th year of disease progression, significantly differing from the other groups (*p* < .001). The low‐risk group had the most gradual decline, while the medium‐risk group showed a more moderate decline. Interestingly, the medium‐risk group present with an insidious progression of DKD. These subgroups can be classified as stable, non‐proteinuric insidiously progressive and proteinuric rapidly progressive based on their trajectory changes. This study identified three different molecular subtypes using the MOVICS multi‐omics integrated clustering algorithm. Further survival analysis demonstrated that subtype 3 (CS3) was associated with a poorest prognosis (overall *p* < .001). The study flow is summarised in Graphical Abstract.

DKD is a complex metabolic disease, making it challenging to explain its intricate intrinsic alterations using a single omics data. The integration of multi‐omics data is anticipated to offer a comprehensive understanding of the mechanisms of DKD and identify potential biomarkers.^4^ In this study, a classification diagnostic and risk‐prognostic model for DKD was developed and validated by combining urinary proteomics, metabolomic, blood lipidomic and clinical data. Over a dozen algorithms related to machine learning were employed to enhance the diagnostic precision of the DKD model. The strengths of this study lie in the integration of multi‐omics data within a mixed cohort. Additionally, the study explored the relationship between multi‐omics scores and outcome indicators. By combining multi‐centre omics and genome‐wide association study data analysis, the study achieved dual‐screening, improving the precision and reliability of screening markers. Furthermore, the findings offered molecular clusters for identifying subtypes and presented reliable biomarkers of DKD. However, these biomarkers need further validation of biological functions through in vitro and in vivo experiments.

## 1 LIMITATIONS

Firstly, this study lacked renal biopsy as a gold standard for outcome, it also limited the ability to differentiate DKD from other renal disease. Secondly, the selection of multi‐omics biomarkers may be biased due to the limited sample size. Although we have made efforts to mitigate this issue through double screening, the potential for bias remains. Furthermore, it is important to evaluate the predictive model in external centres, which will be the subsequent phase of this study. The follow‐up period was relatively short, although sufficient endpoint events were recorded. Future efforts will aim to extend the duration of follow‐up and increase the sample size.

## 2 CONCLUSION

In summary, the integration of blood and urine multi‐omics data in diabetic patients can accurately classify their current status and predict the prognosis. This study identified molecular clusters to screen for high‐risk groups of DKD, especially those non‐proteinuric insidious progression of DKD, a subgroup of particular concern in clinical practice. The study also emphasised the importance of cost reduction in clinical scenarios and developed a simplified, reliable and practical prediction model. Furthermore, the identified biomarkers may serve as potential research targets for DKD, providing significant implications for its diagnosis and prevention.

## AUTHOR CONTRIBUTIONS

All authors read and approved the final manuscript. Xian Shao prepared and analysed the data and figures, interpreted the results and wrote the manuscript; Xian Shao and Pei Yu designed this study; Xian Shao, Qian Yang, Pufei Bai, Suhua Gao, Lihua Wang, Ying Li and Weixi Wu collected the data, Qian Yang, Pufei Bai, Suhua Gao, Lihua Wang, Ying Li and Weixi Wu validated the results, Xian Shao, Pufei Bai, Suhua Gao, Ying Li, Saijun Zhou, Mingzhen Pang and Hongyan Liu revised the manuscript.

## CONFLICT OF INTEREST STATEMENT

The authors declare no conflicts of interest.

## FUNDING INFORMATION

This study was funded by Tianjin Science and Technology Major Special Project and Engineering Public Health Science and Technology Major Special Project (No. 21ZXGWSY00100), Tianjin Natural Science Foundation Key Projects (No. 22JCZDJC00590), Tianjin Key Medical Discipline (Specialty) Construct Project (No. TJYXZDXK‐032A), Scientific Research Funding of Tianjin Medical University Chu Hsien‐I Memorial Hospital (No. ZXY‐ZDSYSZD‐1), First Level Leading Talent Project of ‘123 Climbing Plan’ for Clinical Talents of Tianjin Medical University. ‘Tianjin Medical Talents’ project for the second batch of high‐level talents selection project in health industry in Tianjin (No. TJSJMYXYC‐D2‐014). Science and technology project of Tianjin Health Commission (No. TJWJ2024QN032). The funder was not for profit and has no role in the data collection, analysis or interpretation; trial design; patient recruitment; or any aspect pertinent to the study.

## ETHICS STATEMENT

All procedures performed in studies involving human participants were in accordance with the Declaration of Helsinki Helsinki Declaration and the Regulations on the Management of Clinical Observation issued by the National Science and Technology Commission. This study is an anonymous, non‐interventional observational research that did not negatively impact the health of the subjects. Additionally, the participants remain anonymous throughout the study. Both the prospective and retrospective study were approved by the Ethics Committee (Institution of the Ethics Committee: Chu Hsien‐I Memorial Hospital of Tianjin Medical University; Ethics Approval No. ZXYJNYYKMEC2023‐47).

## CODE AVAILABILITY STATEMENT

The code are available from the corresponding author upon reasonable individual request.

## CONSENT FOR PUBLICATION

Written informed consent for publication was obtained from all participants. All authors agree to be accountable for all aspects of the work in ensuring that questions related to the accuracy or integrity of any part of the work are appropriately investigated and resolved.

## LETTER‐TO‐EDITOR WITH PREVIOUS SUBMISSION NUMBER

CTM2‐2024‐08‐2591 (REX‐PROD‐1‐625B58B2‐4E5A‐4DEB‐9F1A‐19FA03F4D87B‐0CDB5296‐8070‐4C97‐98DA‐BC07D51BA98E‐94625).

## Supporting information



SUPPORTING INFORMATION TITLES AND LEGENDS
APPENDIX 1 Inventory of Supporting Information. This appendix provides further methodological detail for this study.

APPENDIX 2 Inventory of Supporting Information. This supplement provides additional figures and tables containing more detailed results for this study.

## Data Availability

The blood and urine multi‐omics data are available from the iProx platform (ID: IPX0009293000, https://www.iprox.cn//page/project.html?id=IPX0009293000). The participants datasets generated and analysed during the current study are not publicly available and cannot be uploaded to any website due to the risk of compromising the individual privacy of participants. However, the data used to reach the study conclusions are available from the corresponding author upon reasonable request.
