# Supplementary material for: Machine learning‐based multi‐omics models for diagnostic classification and risk stratification in diabetic kidney disease
Source: Clin Transl Med. 2025 Jan 8;15(1):e70133. doi: 10.1002/ctm2.70133 (PMC11707431; doi:10.1002/ctm2.70133)
Supplement: Supplementary file 1 — SUPPORTING INFORMATION TITLES AND LEGENDS APPENDIX 1 Inventory of Supporting Information. This appendix provides further methodological detail for this study. [file CTM2-15-e70133-s001.docx]

**Appendix 1: Inventory of Supplemental Information**

This appendix provides further methodological detail for this study.

**Section 1.** Sample Size Calculation. 3

**Supplementary Figure 1.** Sample size calculation results from PASS 15

**Section 2.** Study design and Sample Size Calculation 3

**Section 3.** Definition of Groups 3

**Section 4.** Body Measurements 4

**Section 5.** Blood and Urine Sample Collection and Processing 4

**Section 6.** Urine Four Dimension Data Independent Acquisition (4D-DIA) Proteomics, Untargeted Metabolomics and Blood Untargeted Lipidomics detection 6

**Section 7.** Multicenter Proteomics and Metabolomics Satabase Scquisition and Pre-screening 6

**Section 8.** Acquisition and Pre-processing of Genetically Proxied Data for Exposure and Outcome 7

**Section 9.** Detection of Biomarkers using Enzyme-linked Immunosorbent Assay (ELISA) Kits 8

**Section 10.** Machine Learning Algorithms 8

**Section 11.** Quality Control of Data. 8

**Section 12.** Quantitative Statistical Analysis and Functional Analysis 8

**Section 13.** Hierarchical Clustering Analysis 9

**Section 14.** MFUZZ Temporal Expression Cluster Analysis 9

**Section 15.** KEGG and GO Analysis 9

**Section 16.** Protein-Protein Interaction (PPI) Network Analysis 10

**Section 17.** Weighted Gene Co-expression Network Analysis (WGCNA) 10

**Section 18.** Transcription factor (TF) Enrichment Analysis 10

**Section 19.** Metabolite Identifications and MSEA Enrichment Analysis. 10

**Section 20.** Multidimensional Intergroup Analysis of Variance (Metabolite) 10

**Section 21.** Differential Abundance Score (DAS) 11

**Section 22.** Enrichment Analysis of Differential Proteins Combined with Metabolites. 11

**Section 23.** O2PLSDA Analysis 11

**Section 24.** Lipid Identification and MSEA Enrichment Analysis 11

**Section 25.** Lipid Chain Length and Saturation Analysis 12

**Section 26.** Feature Engineering. 13

**Supplementary Figure 2.** Procession of screening biomarkers for DKD

**Section 27.** Baseline Screening Period and Follow-up Period Evaluation. 14

**Supplementary Table 1.** The schedule of clinical observation and evaluation

**Section 28.** Study flow chart of classification models for DKD 16

**Supplementary Figure 3.** Study flow chart of classification models for DKD

**Section 29.** Study flow chart of risk-prognostic models for DKD. 17

**Supplementary Figure 4.** Study flow chart of risk-prognostic models for DKD

**Section 30.** The Correlation Between Multi-omics Cox Risk Scores with Dynamic Patterns of Change in Renal Function Over a 10-year Period. 19

**Section 31.** Identification of High-risk Subtypes of DKD based on the Multi-omics Integrated Clustering Algorithms. 19

**Section 32.** Statistical Analysis. 19

**Reference** 20

#### Section 1. Sample Size Calculation.

In According to the Chinese Guidelines for the Prevention and Control of Diabetic Kidney Disease (DKD)^1^, the prevalence of DKD among type 2 diabetes mellitus (T2DM) was 21.8%. In the cross-sectional study (two-sided α = 0.05, tolerable margin of error = 20%), PASS 15 (Confidence Intervals for One Proportion) calculations determined that a minimum of 17 individuals needed to be included in each group.


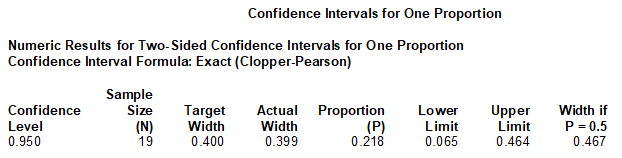


**Supplementary Figure 1. Sample size calculation results from PASS 15.**

The prevalence of DKD in T2DM patients was about 21.8% in China^1^ based on N=KxQ/P (P = the prevalence rate of DKD, Q=1-P) with a tolerance error range of 20%, Kx=100, and based on a loss to follow-up rate of 10%, the sample size was calculated to be about 394 individuals.

#### Section 2. Study design

Approximately 70% of urinary proteins and peptides are sourced from the kidneys, with the concentration of renal injury-associated proteins being significantly higher in urine compared to blood. Urine is a non-invasive, easy, and simple biosample to collect, which is highly reproducible, making it an ideal sample for discovering renal biomarkers^4,5^.DKD is a complex and multifactorial disease, and multiple biomarkers can cover different aspects of the DKD progression process compared to a single biomarker, and the synergistic effect between each other can help to reduce the bias and thus enhance the predictive ability, and also provide a strong methodological basis for finding the biological information for the diagnosis of DKD^6^. It is imperative to develop simple, reliable, cost-effective and accurate biomarker prediction models that address the challenges of cost, availability and reproducibility in clinical application scenarios^7^.

The term "metabolomics" was first proposed in 2001 by Oliver Fiehn, who defined it as "the comprehensive and quantitative analysis of all metabolites"^8^. Through metabolomic approaches, numerous metabolites have been identified as potential biomarkers for the onset of DKD, including amino acids, lipids, carbohydrates, and other molecular classes. Researchers such as Saulnier have found that aconitic acid and glycolic acid in urine are associated with damage to glomerular function^9^. Dyslipidemia is a notable characteristic of DKD, resulting from complex interactions among genetic, environmental, and kidney-specific factors. Lipidomics technology can be employed to systematically identify and quantify complex lipids, thereby elucidating the biological significance of lipidomics in DKD research^10^. In summary, protein and metabolic changes are more readily detectable in urine, whereas lipid changes are primarily more evident in blood.

By integrating and analyzing multi-omics data, we can leverage their complementary strengths and validate each other to assess individual status. This approach allows speculation on DKD progression and facilitates personalized and continuous detection and management of DKD. In this study, through blood lipidomics, urine proteomics, and metabolomics technologies, potential DKD markers are identified to establish an accurate early warning model for DKD. This study aims to provide effective tools for the individualized prevention and treatment of DKD, and help to explain the associations between different molecules and their risk of DKD from multiple perspectives.

The study was conducted at Chu Hsien-I Memorial Hospital in Tianjin, China, between 1 January 2022 and 1 February 2024. This mixed study included prospective, cross-sectional and retrospective studies (review of both prospective and cross-sectional studies).

**Inclusion criteria:** 1. T2DM (according to the "The American Diabetes Association (ADA) "Standards of Care in Diabetes"^2^: typical symptoms (polydipsia, polyphagia, polyuria and unexplained weight loss), and random blood glucose ≥ 11.1 mmol/L, or fasting blood glucose (at least 8h without calorie intake) ≥ 7.0 mmol/L, or blood glucose in oral glucose tolerance test (2h) ≥ 11.1 mmol/L. Those who do not have typical symptoms of T2DM need to be confirmed on another day; 2. Receive standard oral hypoglycemic drug therapy; 3. Residents of Tianjin who are 18 years or older (residents are defined as someone who have lived in the local area for at least 6 months); 4. All participants volunteered and signed informed consent forms.

**Exclusion criteria:** 1. Women who are pregnant, planning to become pregnant, breastfeeding, or have a desire to have children in the near future. 2. Individuals with serious diabetic complications such as diabetic ketoacidosis, hyperosmolar hyperglycemic syndrome, or lactic acidosis. 3. Patients combined with haematuria or urinary tract infections. 4. Participants who have experienced acute myocardial infarction, cerebrovascular accidents, or serious infections in the 6 months prior to enrollment. 5. Subjects with a history of heart failure, myocardial infarction, or previous percutaneous coronary intervention. 6. Individuals with poorly controlled hypertension, defined as blood pressure exceeding 180 mmHg systolic or 110 mmHg diastolic. 7. Those with history of immunocompromised conditions or malignant tumors. 8. Use of glucocorticoids or other medications affecting blood glucose within the last 6 months. 9. History of renal transplantation.

**Prospective Design, Primary and Secondary Endpoints:** The study cycle begins with enrollment on 1 January 2022 and ends with follow-up on 1 February 2024. Each subject is required to undergo screening (2 weeks prior to baseline), with at least one follow-up visit during the study period (follow-up > 6 months), and provide blood and urine samples. eGFR values should be collected from baseline to the follow-up endpoint, as well as retrospectively from each year since 2014. The average annual eGFR decline slope and percentage change for patients should be calculated.

The primary endpoint is defined as the diagnostic criteria for DKD. Secondary endpoints, as previous studies, require the fulfillment of one of the following conditions: 1. The presence of microalbuminuria (UACR > 30 mg/g) in at least one morning fasting urine sample. 2. ≥30% decrease in eGFR change or a 1-fold increase in serum creatinine from baseline, or ≥3 ml/min/1.73m^2^/year slope of decline in eGFR from baseline. 3. Fatal and non-fatal cardiovascular disease (myocardial infarction, stroke, coronary interventions, coronary bypass grafting, percutaneous transluminal coronary angioplasty, heart failure), and renal death. The composite end point include primary and secondary endpoints.

#### Section 3. Definition of Groups

The HC group consisted of individuals aged 18 years or older, residents of Tianjin, China, who voluntarily participated in the study, signed an informed consent form, and did not have diabetes or any chronic diseases. Age and gender composition were matched with the other three groups.

The HR-DKD group requires meeting specific criteria related to DKD risk factors as outlined in the literature^3, 4^. These criteria include: 1. Hypertension, defined as blood pressure ≥140/90 mmHg or receiving treatment for hypertension. 2. dyslipidemia as per the “Chinese guideline for lipid management”^5^, or use of lipid-lowering drugs. 3. Age ≥45 years. 4. Obesity, defined as body mass index (BMI) ≥28kg/m2. 5. Smoking or alcohol use. 6. History of T2DM for ≥10 years. 7. History of fundus diabetic retinopathy. 8. Estimated glomerular filtration rate (eGFR) ≥60 ml/min/1.73m2 and urinary albumin to creatinine ratio (UACR) <30mg/g. To be classified as HR DKD, the 8th criterion must be met, along with 3 or more of criteria 1-7. The T2DM group, on the other hand, must meet criterion 8 and have 2 or fewer of criteria 1-7 to be included.

According to the Chinese Guidelines for Diagnosis and Treatment of DKD^6^, diagnosis of DKD can be made if any of the following criteria are met: 1. Albuminuria, defined as a UACR ≥30mg/g, with confirmation of this level in 2 out of 3 tests conducted within 3-6 months, and after excluding interfering factors such as infections, strenuous exercise, and fever. or 2. a normal or mildly decreased eGFR of <60 ml/min/1.73m2 for over 3 months can also indicate DKD, even in the absence of proteinuria. 3. Other renal conditions must be ruled out, including primary nephropathy and hypertensive nephropathy. 4. The renal biopsy showing pathological changes consistent with DKD can confirm the diagnosis.

#### Section 4. Body Measurements

Height was measured by trained and certified observers using a wall-mounted gauge accurate to 0.1cm. Weight was measured on a calibrated scale with the subject barefoot in indoor singlet, accurate to 0.5kg. Waist circumference was measured horizontally at the end of expiration between the last rib and the iliac crest using a non-elastic ruler, accurate to 0.1cm. Hip circumference was measured with a tape measure horizontally at the most convex point of the anterior pubic symphysis and posterior gluteus maximus. BMI categories were defined as follows: 24-27.9kg/m2 for overweight and ≥28 for obese. Hip circumference was measured accurately to 0.1cm between the anterior pubic symphysis and the most convex part of the gluteus maximus muscle. Central obesity was defined as waist circumference ≥85cm for men and ≥80cm for women. Blood pressure was measured after 5 minutes of rest in a quiet environment, with no smoking, alcohol, or coffee consumption. Measurements were taken in the sitting position on the right side using a calibrated sphygmomanometer, averaged from two readings, and repeated three times for subjects with large fluctuations. Subjects also underwent additional examinations including electrocardiogram and fundoscopy, conducted by medical personnel following standardized procedures.

#### Section 5. Blood and Urine Sample Collection and Processing

Comprehensive information of patient were double-checked and completed in accordance with the questionnaire instructions. Clinical risk score for DKD is calculated based on the number of risk factors.

Fasting blood samples were collected to measure indicators including fasting blood glucose, fasting insulin, fasting C-peptide, glycated hemoglobin (HbA1c), liver and renal function indicators, lipid levels, blood routine and electrolytes. Additionally, morning and 24-hour urine samples were retained for further analysis, including measurements of ACR, 24-hour urine biochemistry, urine electrolytes, and renal injury-related proteins such as N-acetyl-glycosaminidase (NAG), α1-microglobulin (α1MG), immunoglobulin G (IgG), retinol binding protein (RBP), transferrin (TRF), β2-microglobulin(β2MG), and microalbumin.

Blood sample collection: Subjects were required to fast for a minimum of 10 hours prior to blood collection, with blood drawn after dinner. Venous blood was collected separately in non-anticoagulated tubes, serum was separated within 2 hours and placed in EDTA anticoagulation tubes. Plasma was obtained by centrifugation and stored at -80°C. Blood glucose was measured using the automated glucose oxidase method, fasting insulin and C-peptide were measured using chemiluminescence immunoassay, and glycated hemoglobin was measured using high-performance liquid chromatography. Liver function markers such as ghrelin, glutamine, glutamate dehydrogenase, and glutamine-alanine dehydrogenase were measured using UV-malate dehydrogenase and UV-lactate dehydrogenase methods. Renal function markers like blood urea nitrogen, creatinine, and uric acid were measured using various enzymatic methods. Cystatin C was assessed using a particle-enhanced scattering immunoturbidimetric assay. Lipid profile components (TC, TG, LDL-C, HDL-C) were measured using the oxidase method, while electrolytes were assessed using an ion-selective electrode method. The estimated glomerular filtration rate (eGFR) was calculated based on the CKD-EPI equation.

Urine specimen collection: Candidates were advised to follow a light diet for the first three days. It is recommended to clean the urethral opening and vulva before collecting specimens to prevent contamination from menstrual blood, leucorrhea, semen, feces, etc. Containers should be free from drugs and disinfectants to avoid damaging tangible components and affecting the detection of chemical components and hormone activity. 200ml of the first morning urine on the day of enrollment should be collected, ideally delivered within half an hour but no longer than 2 hours. Centrifugation at 5000g for 30-60 minutes at 4°C, followed by storage of the supernatant at -80°C.

24-hour urine collection: A clean container with preservative should be used to collect urine from 7 a.m. to 7 a.m. the following day. During collection, urine should be refrigerated or kept in a cool place for preservation.

Use the turbidimetric method to measure urinary microalbumin and the sarcosine oxidase method to measure urinary creatinine. Calculate the urinary albumin-to-creatinine ratio (mg/g) by dividing urinary microalbumin by urinary creatinine. For 24-hour urine biochemistry analysis, include quantification of urinary protein, urinary microalbumin, uric acid, glucose, creatinine, calcium, phosphorus, potassium, sodium, and chloride. The six items of urinary protein to be measured are urinary α1 microglobulin, immunoglobulin G, retinol-binding protein, transferrin, β2-microglobulin, and microalbumin using the immunoturbidimetric method.

All cases should be carefully observed and filled in the case report form according to the above plan. Various laboratory tests and test reports should be filled in in chronological order on the case report form, checked by two people, and filled in according to the instructions. The data is collected by a dedicated person, who fills in the paper case record form; then another person manages the input into the Excel table, and the imaging professional is responsible for collecting and analyzing the imaging data; after each data is statistically processed according to statistical requirements, an overall summary is made. Evaluate and save all research materials, including confirmation records of subjects, case report forms, informed consent forms.

#### Section 6. Urine Four Dimension Data Independent Acquisition (4D-DIA) Proteomics, Untargeted Metabolomics and Blood Untargeted Lipidomics detection

A total of 6 individuals from each group were randomly selected for testing. We utilized the 4D-DIA technique to analyze urine proteomics. The experimental process involved protein extraction, peptide digestion, chromatographic separation, liquid chromatography-tandem mass spectrometry (LC-MS/MS) DDA data acquisition, and database searching. Metabolites detection were carried out using the UnTargeted Ultrahigh Performance Liquid Chromatography-Tandem Mass Spectroscopy (UPLC-MS) method. An untargeted lipidomic analysis platform, utilizing the UPLC-Orbitrap mass spectrometry system in combination with LipidSearch software (Thermo Scientific™) and isotopic internal standards of 13 lipid molecules, was employed for lipid identification and data preprocessing. Before proceeding to data analysis, the instrument stability, experiment reproducibility, and reliability of data were comprehensively evaluated.

Utilizing 4D-DIA technology for urine proteomics analysis is a novel approach in mass spectrometry. DIA, a recent advancement in mass spectrometry, offers a comprehensive view of all ions present in a sample, unlike the selective approach of traditional DDA technology. The experimental workflow involves protein extraction, peptide enzymatic hydrolysis, chromatographic fractionation, liquid chromatography-tandem mass spectrometry (LC-MS/MS) for DDA data collection, followed by database search and other pertinent steps. This analysis was carried out by Shanghai Applied Protein Technology (project number: YAS202303200050-1).

The metabolites detection was conducted using Untargeted, Ultrahigh Performance Liquid Chromatography-Tandem Mass Spectrometry (UPLC-MS). This analysis was carried out by Shanghai Applied Protein Technology (project number: YAS202303200050-2).

A non-targeted lipidomics analysis platform utilizing the UPLC-Orbitrap mass spectrometry system, in conjunction with LipidSearch software from Thermo Scientific™ and isotope internal standards of 13 lipid molecules for lipid identification and data preprocessing, was employed for the analysis. The study was conducted by Shanghai Applied Protein Technology (project number: YAS202303200050-3).

#### Section 7. Multicenter Proteomics and Metabolomics Database Acquisition and Pre-screening

In collaboration with a multi-center proteomics database, protein markers were pre-screened using test results from a specific center, resulting in multiple biomarkers. This study seeks to improve the accuracy and reliability of these biomarkers by integrating data from public proteomics databases across different centers and countries. By validating expression differences and diagnostic precision of identified markers, this approach aims to enhance understanding. The findings are expected to provide strong evidence, identify disease-associated biomarkers, and offer a more accurate assessment of kidney damage in diabetic patients.The multicenter proteomics data acquisition and preprocessing process involved collecting five urine proteomics analysis datasets from the PRIDE database (https://www.ebi.ac.uk/pride/): PXD017213, PXD018996, PXD012413, PXD008683, and PXD016571. Specifically, the PXD017213 dataset, analyzed using the Thermo Scientific EASY-nLC1000 system, included 15 normal samples and 15 diabetic samples. The PXD018996 dataset consisted of 72 normal samples and 35 samples from patients with Diabetic Kidney Disease (DKD) (https://www.ebi.ac.uk/pride/archive/projects/PXD018996). The PXD012413 dataset contained 116 normal samples and 104 diabetic samples. The PXD008683 dataset had 5 normal samples and 5 diabetic samples, while the PXD016571 dataset included 54 samples from diabetic patients and 20 samples from DKD patients. By merging these datasets, the urine proteomic profiles of 208 normal subjects (NC), 158 diabetic patients (DM), and 55 DKD patients were ultimately obtained.

To evaluate the prediction performance of individual protein markers, the dataset was divided into a training set and a test set at a 3:1 ratio. The 15 proteins identified in the initial phase were assessed sequentially using random forest, with parameters chosen through 20 rounds of cross-validation. The model was then trained and its classification performance on the three groups (NC, DM, DKD) in the training set was measured using various metrics including accuracy, sensitivity, specificity, precision, recall, and AUC. The predictive value of the markers was analyzed and visualized through a confusion matrix and ROC curve, considering their biological relevance to identify reliable and accurate indicators. Twelve machine learning algorithms were employed to construct a prediction model utilizing selected indicators. The algorithms utilized encompass Linear Discriminant Analysis (LDA), Stochastic Boosted Gradient Trees, Multivariate Adaptive Regression Splines (MARS), Support Vector Machines (SVM), Naive Bayes, Recursive Partitioning Classification Trees (RPCT), Random Forest (RF), Regularized Discriminant Analysis (RDA), Decision Tree Classification, K-nearest neighbor classifier, and Neural Network Classifier.

OMIX002578, and its corresponding clinical information from the OMIX database (https://ngdc.cncb.ac.cn/omix/). The dataset comprised 14 Normal samples, 42 diabetic samples, and 132 diabetic nephropathy samples. Standardization of the data source was carried out, followed by statistical analysis of expression differences between groups using the 'limma' package in R language. Box plots depicting the expression trends of the identified proteins across different groups were generated using the ANOVA method. The dataset was divided into a training set and a test set at a ratio of 3:1. The prediction performance of the metabolic markers identified in the initial phase was then assessed using the random forest method. Subsequently, reliable and accurate indicators were selected based on their biological significance.

#### Section 8. Acquisition and Pre-processing of Genetically Proxied Data for Exposure and Outcome

Mendelian Randomization (MR) is a method that utilizes whole-genome sequencing data (GWAS summary data) and employs single nucleotide polymorphisms (SNPs) as instrumental variables (IV) to estimate causal relationships between exposure factors and outcomes with a higher level of evidence. Exposure and outcome data sets can be obtained from the GWAS Catalog website (https://www.ebi.ac.uk/gwas/). The exposure data includes L-glutamic acid (GCST90200412), 7,8-dihydrobiopterin (GCST90265032), L-carnitine (GCST90199621), 1-methylhistidine (GCST90199691), and L-methionine (GCST90199625), TG(18:3e_18:4_19:1) (GCST90024545), TG(20:5_14) :1_22:6)(GCST90024572), PE(16:0_18:1)(GCST90024351), PG(47:3)(GCST90024600), DG(8:0_11:2)(GCST90024706), PC(33:3)(GCST90060261), PC(35:3e)(GCST90060319), PE(18:0_18:1)(GCST90024365), PE(18:0_20:3)(GWAS ID: GCST90024367), PE(18:1_18:1)(GCST90024374), PE(39:5e)(GCST90060346), SM(t18:0_23:6)(GCST90200123). The outcome data pertains to Diabetic Nephropathy (GCST90018832), with a sample size of 585,264, comprising 584,012 controls and 1,252 cases.

Depending on the version of the reference genome, the chr:pos in the GWAS summary data was converted to SNP information using the "MungeSumstats" package. SNPs associated with the exposure group were screened using P-value < 1×10-4. Samples from Europe were clustered with an r2 = 0.1 threshold to assess linkage disequilibrium (LD). Instrumental variables (IVs) were selected from the outcome GWAS summary data, excluding SNPs strongly associated with the outcome (P > 5e-8). The exposure and outcome data were harmonized using the 'harmonise_data' function, followed by removal of palindromic data and calculation of R2 and F values. The MR analysis was conducted using the “TwoSampleMR” R package. Q-tests (MR Egger and IVW) were performed to assess heterogeneity and ensure the robustness of the results. And Steiger test were performed to test causal direction and avoid reverse causality relationship.

#### Section 9. Detection of Biomarkers using Enzyme-linked Immunosorbent Assay (ELISA) Kits

After centrifugation, the supernatant should be taken for detection. Standard wells and sample wells should be set up. In each standard well, add 50 μL of standards of different concentrations, and in the sample well, add 50 μL of the sample to be tested; no liquid should be added to the blank well. In the standard well and sample well, add 100 μL of horseradish peroxidase (HRP)-labeled detection antibody to each well, seal with a film, and incubate in a 37°C water bath or incubator for 60 minutes. Discard the liquid, pat dry on absorbent paper, fill each well with washing solution (350 μL), let it stand for 1 minute, shake off the washing solution, pat dry on absorbent paper, and repeat washing the plate 5 times (the plate can also be washed with a plate washer). Add 50 μL each of substrates A and B to each well, and incubate at 37°C in the dark for 15 minutes. Add 50 μL of stop solution to each well, and within 15 minutes, measure the OD value of each well at a wavelength of 450 nm. Take the OD value of the measured standard substance as the abscissa and the concentration value of the standard substance as the ordinate. Draw a standard curve on graph paper or use relevant software to obtain a linear regression equation. Substitute the OD value of the sample into the equation to calculate the sample concentration.

#### Section 10. Machine Learning Algorithms

A multi-classification prediction model is constructed using 12 machine learning algorithms, including Linear Discriminant Analysis (LDA), Stochastic Boosted Gradient Trees, Multivariate Adaptive Regression Splines (MARS), Support Vector Machines (SVM), Naive Bayes, Recursive Partitioning Classification Trees (RRCT), Random Forest (RF), Regularized Discriminant Analysis (RDA), Decision Tree Classification, K-nearest neighbor classifier, and Neural Network Classifier.

The dataset was divided into a training set and a test set in a 3:1 ratio. The most optimal model was explored by combining different omics data using four approaches: mono-omics model, multi-omics model, multi-omics combined clinical data model and clinical data model. A total of 12 machine learning algorithms were used to build multiclassification prediction models. The receiver operating characteristic (ROC) curve and confusion matrices was employed to assess the diagnostic performance of the models, with a higher Area under curve (AUC) indicating better classification. Accuracy, Sensitivity and specificity of the models were also evaluated. The Net Reclassification Improvement Index (NRI) was used to evaluate the improvement effect in prediction models. A risk score union plot was utilized to differentiate between high- and low-risk populations based on the median risk score. The Kaplan-Meier (KM) survival curves were used to assess the cumulative risk of DKD.

#### Section 11. Quality Control of Data.

Quality control measures were implemented to ensure the stability and reliability of experimental data. A QC sample, representing a mix of all samples, was included at regular intervals within the sample cohort. The consistency of each QC sample inserted during the experiment was assessed using metrics such as Coefficient of Variation (CV), Principal Component Analysis (PCA), and Pearson correlation analysis. A lower CV value, greater sample aggregation in PCA, and a correlation coefficient closer to 1 indicated a stable experimental system.

#### Section 12. Quantitative Statistical Analysis and Functional Analysis

Bar charts were employed to illustrate the results obtained from the analysis. The Metascape database (http://metascape.org) was utilized to identify the organ sources, spatial distributions, and subcellular localization of differential proteins across different comparison groups. The findings were quantified and visualized through bar charts. Proteins originating from various organs, spatial distributions, and subcellular organelles exhibit diverse cellular functions. Studying protein localization can enhance our understanding of their roles within cells. Organ sources include bladder/urethra, blood/bone marrow/lymph nodes, kidneys, and liver. Spatial distributions encompass secretory, transmembrane, membrane surface, cytoplasmic, and kinase types. Subcellular organelle sources consist of actin filaments, cell junctions, cell membrane, endoplasmic reticulum, Golgi apparatus, mitochondria, nucleus, plasma membrane, and vesicles.

Protein domains are specific regions within larger protein molecules that possess unique three-dimensional structures and perform distinct biological functions. Interactions between proteins and other molecules often depend on these structural domains. Alterations in amino acids or modifications within a domain can affect the essential functions of the protein. The prediction of domains is crucial for investigating the functional regions and biological roles of proteins. Domain prediction for proteins that are expressed differentially was conducted using the InterPro website, and a histogram was generated to visualize the top 20 proteins with domains. To identify enriched domains and their corresponding proteins, Fisher's Exact test was employed to analyze domain enrichment in the differentially expressed proteins.

#### Section 13. Hierarchical Clustering Analysis

To examine the expression patterns of samples both within and between groups in order to visualize changes in protein expression at different disease stages, a hierarchical clustering algorithm is used to categorize differentially expressed proteins in the comparison group and organize them based on similarity, depicted in a diagram format. Generally, the clustering outcomes demonstrate high data pattern similarity within groups and low similarity between groups, enabling clear differentiation between the groups. The 'pheatmap' package is utilized for cluster heatmap analysis and to display the Top50 results of differentially expressed proteins.

#### Section 14. MFUZZ Temporal Expression Cluster Analysis

The study aims to analyze the expression pattern of protein molecule clusters in various sample groups at different stages of diabetic nephropathy to illustrate the changing trends in protein expression. The optimal number of clusters is determined using 'NbClust' and 'vegan', and the fuzzy c-means (FCM) algorithm in the 'Mfuzz' package is utilized for analysis. Proteins are categorized into different expression modules based on their expression trends. Proteins within a cluster display similar temporal expression characteristics, while the dynamic patterns of proteins across different clusters exhibit significant variations.

#### Section 15. KEGG and GO Analysis

KEGG and GO analysis are vital tools for obtaining a thorough understanding of protein function, location, and biological pathways in organisms. Proteins are annotated using the Gene Ontology (GO) system, which categorizes functional annotations into Biological Process (BP), Molecular Function (MF), and Cellular Component (CC). Differences between these categories are assessed using Fisher's exact test to identify enriched functional categories among differentially expressed proteins (P < 0.05). Bar graphs are commonly utilized to visually display the enrichment of GO entries across the three main categories. To comprehensively analyze biological processes, disease mechanisms, and drug action mechanisms, it is often essential to interpret changes in protein interactions and metabolic pathways. Proteins are further examined and annotated using the KEGG pathway database, with significant differences identified by Fisher's exact test to pinpoint enriched pathway categories among differentially expressed proteins (P < 0.05). Bar graphs are typically employed to visually represent changes in pathway enrichment, and investigations into pathway alterations at various disease stages are facilitated by CytoScape software.

#### Section 16. Protein-Protein Interaction (PPI) Network Analysis

Protein-Protein Interaction (PPI) analysis involves leveraging protein interaction data from the STRING database (https://cn.string-db.org/) and CytoScape software to construct network diagrams illustrating the interactions of differentially expressed proteins within the comparison group.

#### Section 17. Weighted Gene Co-expression Network Analysis (WGCNA)

WGCNA involves correlating phenotypes with omics data by analyzing protein expression patterns to identify highly cooperative protein modules. These modules can serve as potential biomarkers or therapeutic targets based on their interconnectivity and association with specific traits. By calculating expression correlation coefficients and constructing hierarchical clustering trees, co-expression modules can be identified and merged based on similar expression patterns. These modules can then be linked to specific traits through grouping. Furthermore, key modules can be selected based on correlation diagrams, with a correlation value close to 1 and a P value less than 0.05 indicating their importance in determining the trait. Core proteins within these key modules can be identified by observing the scatter distribution of Gene Significance and Module Membership within each module.

#### Section 18. Transcription factor (TF) Enrichment Analysis

TF enrichment analysis is conducted by predicting significantly differentially expressed genes (DEGs) through the DAVID website and visualizing the transcriptional regulatory network of key transcription factors using Cytoscape software.

#### Section 19. Metabolite Identifications and MSEA Enrichment Analysis.

Statistical analysis was conducted on the number of metabolite identifications and Metabolite Set Enrichment Analysis (MSEA) was performed. The total number of metabolites identified in positive and negative ion modes was classified based on their chemical classification and affiliation information. MSEA, available at https://www.metaboanalyst.ca/MetaboAnalyst/, was used to analyze differentially accumulating metabolites (DAMs) using Over Representation Analysis (ORA) mode. The analysis involved databases such as SMPDB, KEGG, RaMP, chemical structure classification (Superclass, Mainclass, Subclass), Locations, Exposure, SNPs, among others. Common pathways in different disease stages were identified and displayed using the 'ggplot2' package in R language.

#### Section 20. Multidimensional Intergroup Analysis of Variance (Metabolite)

PCA is an unsupervised data analysis method that linearly combines identified metabolites to create a new comprehensive set of variables. These variables are carefully selected to capture as much information as possible, leading to dimensionality reduction. PCA is utilized to examine the overall distribution trend and differences between sample groups. On the other hand, Partial Least Squares Discrimination Analysis (PLS-DA) is a supervised statistical method that uses partial least squares regression to predict sample categories based on metabolite expression and sample grouping. By establishing a discriminant model, PLS-DA can identify differential metabolites associated with groupings. Orthogonal Partial Least Squares Discrimination Analysis (OPLS-DA) refines PLS-DA by filtering out noise unrelated to classification information, enhancing the model's analytical capabilities. These multidimensional statistical analyses, including PCA, OPLS-DA, provide insights into group differences and variability within groups. Metabolomics data, characterized by high dimensions and variable correlations, benefits from PCA, PLS-DA, and OPLS-DA for dimensionality reduction while preserving essential information.

#### Section 21. Differential Abundance Score (DAS)

DAS is a pathway-based metabolic change analysis method. The differential abundance score can capture the average and overall changes of all metabolites in a certain pathway.

#### Section 22. Enrichment Analysis of Differential Proteins Combined with Metabolites.

It aims to understand the dynamic changes in a living system through proteomics and the retrospective changes in the body through metabolomics. These two omics approaches can mutually influence each other, and a combined analysis can provide mutual validation and supplementation, thereby focusing on the research target and delving deeper into the mechanisms of Diabetic Kidney Disease (DKD). By performing joint enrichment analysis on the identified differential metabolites and proteins using the MetaboAnalyst website (https://www.metaboanalyst.ca/MetaboAnalyst/), common metabolic pathways and differential metabolites and proteins in the same processes can be identified. Additionally, to further integrate the metabolite-disease-protein interaction network, the 'ggplot2' package in R language and CytoScape software are utilized for visualizing the results.

#### Section 23. O2PLSDA Analysis

O2PLSDA analysis is a method that combines differential protein and metabolome data through bidirectional modeling and prediction. This analysis uncovers internal connections between the two groups, reflecting the overall impact and weight of different variables in the model. The greater the weight of a variable, the more significant its disturbance on the other group, helping determine the main genes, metabolites, or proteins driving the association. The loading value evaluates each variable's contribution to group differences, with positive or negative values indicating correlation direction. Visualizing correlated metabolites and proteins through loading plots and bar charts showcases their impact. O2PLSDA analysis is conducted using the 'OmicsPLS' package in R language.

#### Section 24. Lipid Identification and MSEA Enrichment Analysis

Lipid identification involves quantitative statistical analysis and MSEA enrichment analysis, which includes identification of quantitative statistics, lipid composition analysis, and lipid difference analysis. The analysis of lipid composition encompasses lipid subclass composition and lipid content distribution analysis, with lipid composition being sample-specific. Different types of samples, such as cell membranes, mitochondria, and endoplasmic reticulum, contain varying lipid categories and proportions under steady-state conditions. Changes in lipid composition occur under different processing conditions or biological processes, leading to alterations in membrane biophysical properties and functions. Changes in the content of lipid subclasses can indicate changes in lipid function. By comparing the expression changes of lipid subclasses in different samples, important lipid subclasses that may be involved in relevant biological processes can be identified and linked to specific lipid subclass characteristics. This can help explain relevant biological processes or phenotypes. The analysis of lipid content changes involves examination in multiple dimensions, including the whole lipid profile, subclasses, and individual molecules. Lipid difference analysis includes analysis of lipid content, chain length, and chain saturation.

#### Section 25. Lipid Chain Length and Saturation Analysis

Chain length analysis: Lipids exhibit specific physiological and pathological properties based on their chain length. This length impacts the thickness and fluidity of cell membranes, as well as the activity of lipid transport and target proteins. By summing the contents of lipids with the same chain length and calculating differences among various chain lengths within each category, further research can be conducted. This research will focus on clinical parameters, lipid metabolism enzymes, lipid transport proteins, target proteins, and other relevant factors to provide a detailed explanation of the molecular mechanisms involved. Chain saturation analysis: Chain saturation refers to the total number of double bonds in the fatty acid chains of lipid molecules. This saturation level significantly influences lipid function by introducing kinks, reducing acyl chain density, and preventing the transition of cell membranes from fluid to solid states. The saturation of lipids plays a crucial role in disease occurrence and stress response by impacting cell membrane fluidity, which in turn affects cell processes like division, migration, and signal transduction. By examining changes in chain saturation, researchers can identify key lipid metabolism enzymes or sensors and delve deeper into the associated molecular mechanisms.

#### Section 26. Feature Engineering.

Screening of potential proteomic biological markers involves multiple levels and methods. Initially, the Pearson correlation coefficient is computed to assess the relationship among proteins, renal function indicators, and protein expression, identifying proteins with correlation coefficients >0.7. Subsequently, various machine learning algorithms are employed for feature selection, including the LASSO algorithm with 10-fold cross-validation and 1000 iterations, decision tree model with automatic feature selection and nested resampling using inner and outer 5-fold cross-validation, as well as feature importance algorithms like decision tree and random forest. Box plots are used to visualize the expression patterns of selected proteins across different groups using ANOVA method. Differential proteins are further analyzed for significant differences in pairwise comparisons, identification as Hub genes, and correlation with disease progression. Target molecules with high and low intermolecular correlation are identified, and their relationship with renal function indicators is validated through regression analysis. Subsequently, a multi-class prediction model is developed.

It also includes assessing feature importance using various machine learning algorithms, including the O2PLSDA algorithm. By merging common characteristics of metabolites from different groups, key features are identified to pinpoint molecules strongly correlated with disease progression and with low inter-molecule correlation. This information is then used to build a multi-classification prediction model.


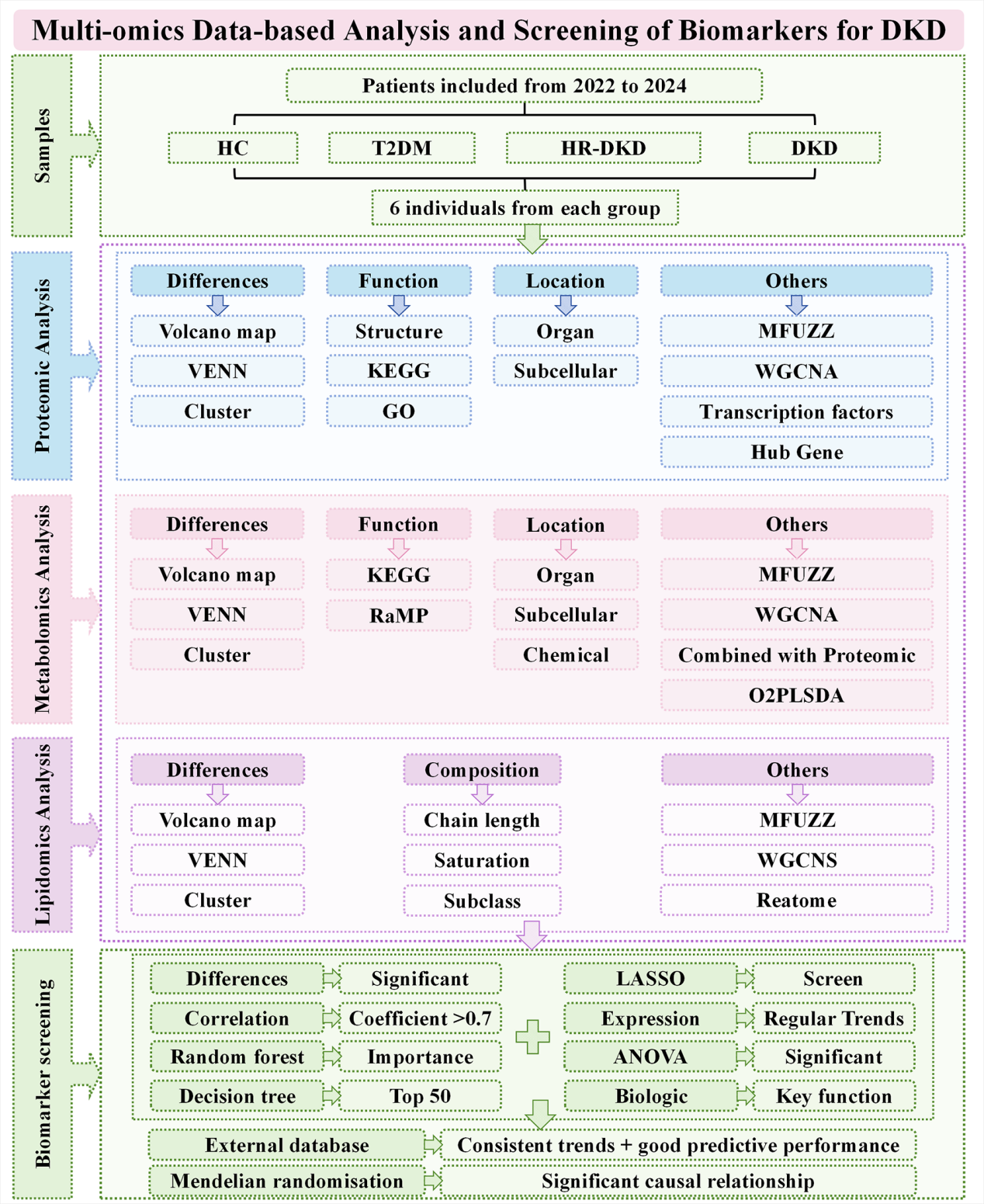


**Supplementary Figure 2. Procession of screening biomarkers for DKD.**

#### Section 27. Baseline Screening Period and Follow-up Period Evaluation.

Screening must be completed within 2 weeks prior to enrollment. The evaluations outlined below must be conducted during baseline screening and at 0, 12, and 24 months of follow-up. The table below summarizes the schedule of clinical observation and evaluation throughout the study.

**Supplementary Table 1. The schedule of clinical observation and evaluation**

| Stage | Screening phase | Incorporation phase |
| --- | --- | --- |
| Collect basic information |  |  |
| Determine inclusion/exclusion criteria | √ |  |
| Completion of demographic information | √ |  |
| Past medical history and treatment | √ |  |
| Current medical history | √ |  |
| Record medication use | √ |  |
| Vital signs | √ |  |
| Relevant laboratory tests |  |  |
| Blood and urine histology |  | √ |
| Abdominal Nuclear Magnetic Examination |  | √ |
| Urine routine | √ | √ |
| Blood Tests | √ | √ |
| Biochemistry | √ | √ |
| Electrocardiogram | √ | √ |
| Fundoscopy | √ | √ |
| Recording of endpoints |  | √ |

#### Section 28. Study flow chart of classification models for DKD


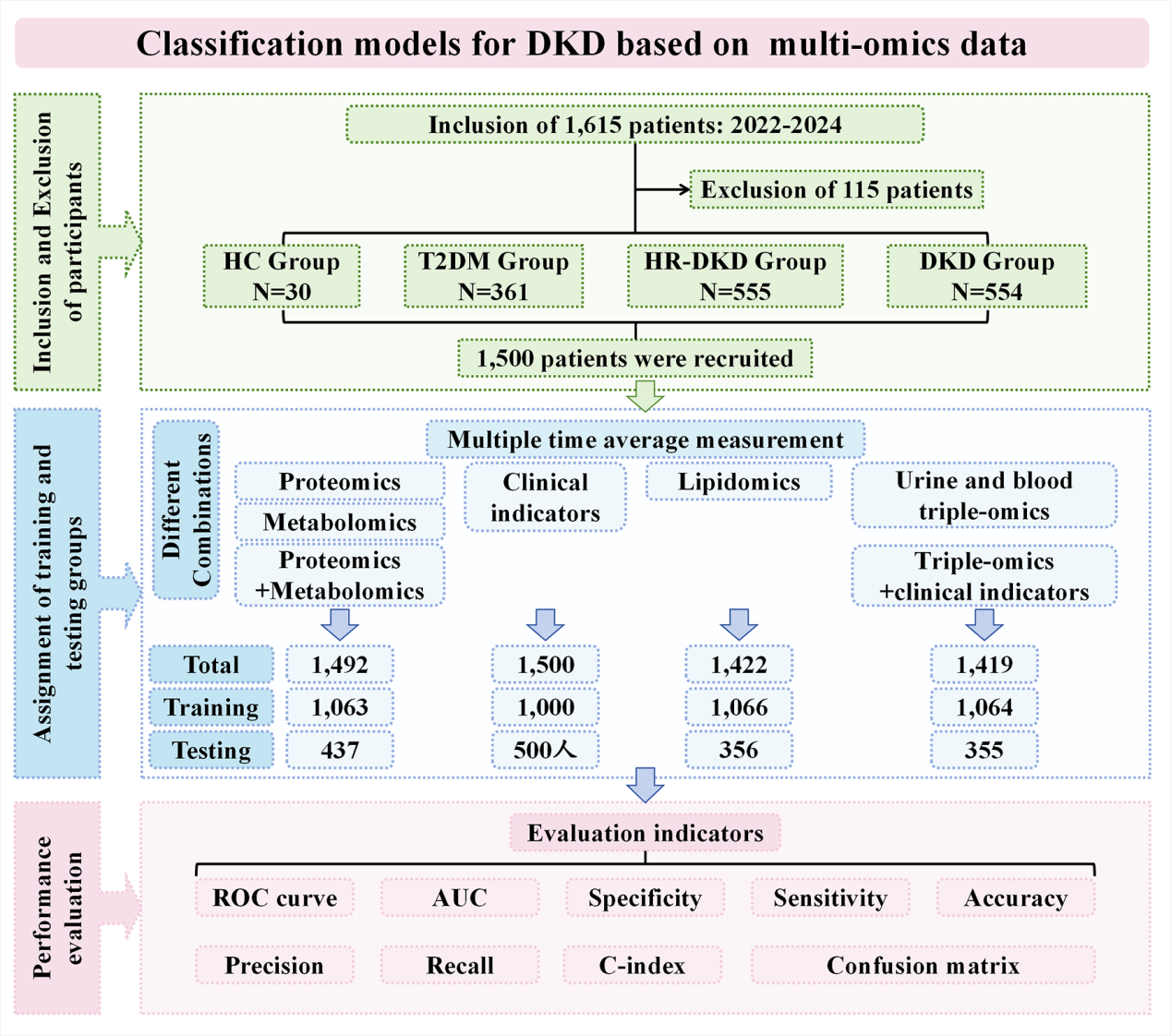


**Supplementary Figure 3. Study flow chart of classification models for DKD**

A total of 1,500 people were enrolled in this study during the period 2022-2024, and a total of seven different combinations of categorical prediction models were established, with patients divided into training and testing groups according to a ratio of 3:1, and the prediction models were evaluated by 9 metrics (ROC curve, AUC, Specificity, Sensitivity, Accuracy, Precision, Recall, C-index, Confusion matirx).

Abbreviations: DKD: diabetic kidney disease; HC: healthy control; T2DM: type 2 diabetes mellitus; HR-DKD: high-risk diabetic kidney disease; ROC: receiver operating characteristic.

#### Section 29. Study flow chart of risk-prognostic models for DKD


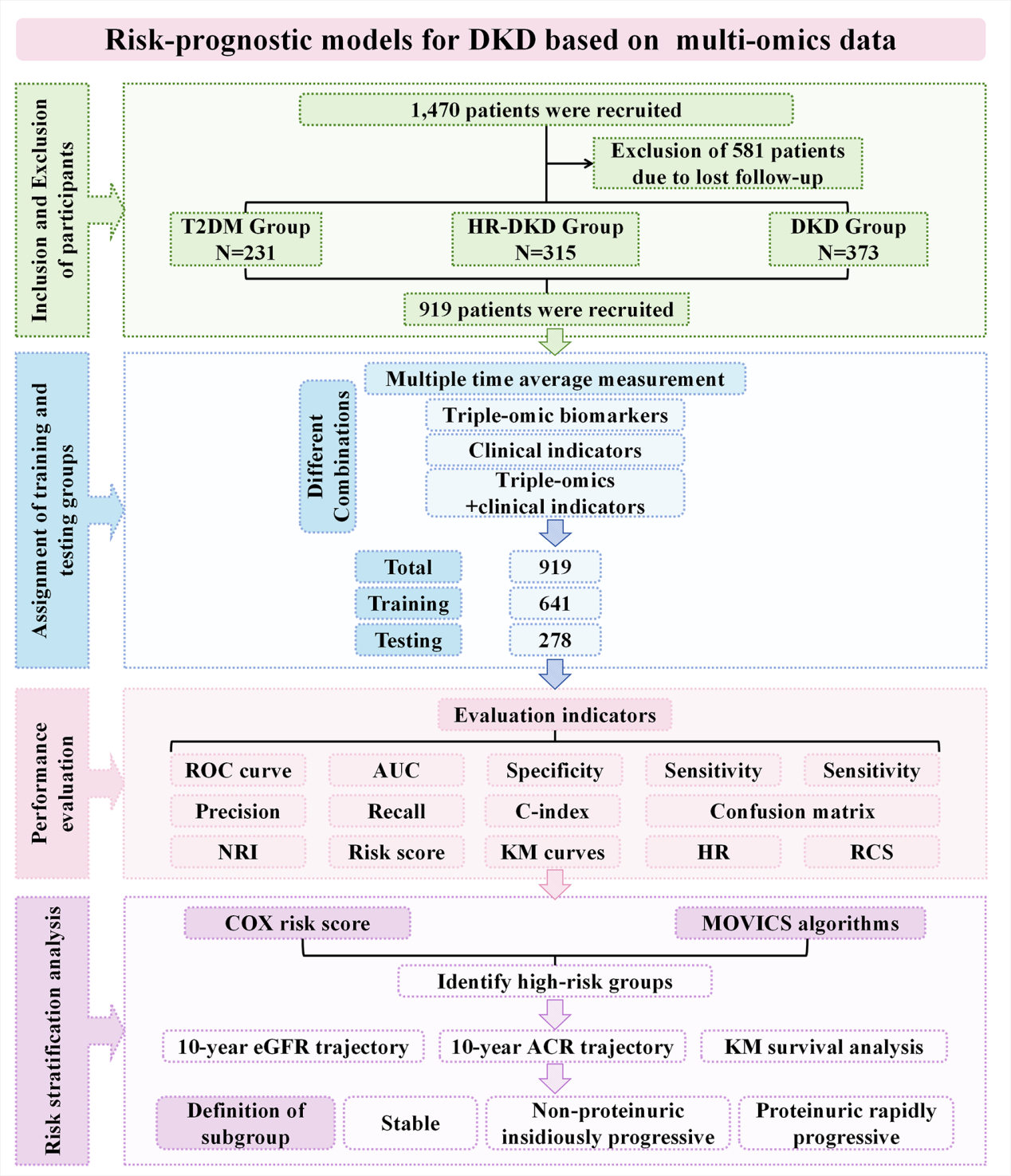


**Supplementary Figure 4. Study flow chart of risk-prognostic models for DKD**

A total of 919 people were enrolled in this study and a total of three different combinations of risk-prognosis prediction models were established, with patients divided into training and testing groups according to a ratio of 3:1, and the prediction models were evaluated by 14 metrics.

Abbreviations: DKD: diabetic kidney disease; HC: healthy control; T2DM: type 2 diabetes mellitus; HR-DKD: high-risk diabetic kidney disease; ROC: receiver operating characteristic; NRI: Net Reclassification Improvement Index; KM: Kaplan-Meier; HR:Hazard ratio; RCS: restricted cubic spline; eGFR: estimated glomerular filtration rate; ACR: albumin-to-creatinine ratio.

#### Section 30. The Correlation Between Multi-omics Cox Risk Scores with Dynamic Patterns of Change in Renal Function Over a 10-year Period

The yearly changes of individuals in eGFR and ACR over a 10-year period were monitored in order to understand the trends in the progression of DKD. The risk scores were determined using a multi-omics Cox risk prediction model and grouped based on tertiles of the scores. Using a multiple-measurement mixed-effects model, the trends of eGFR and ACR were monitored for different groups to investigate the ability of multi-omics scores to differentiate prognostic outcomes.

#### Section 31. Identification **of High-risk Subtypes of DKD based on the Multi-omics Integrated Clustering Algorithms (**MOVICS**)**

A total of 10 clustering algorithms were used in this study. These algorithms were employed to classify patients with DKD into subtypes based on the optimal number of clusters. The risk prognosis of DKD was then evaluated based on the molecular typing of individuals, and the changes in cumulative survival of DKD in different subgroups were analyzed using KM survival curves.

#### **Section 31. Statistical Analysis**

Data analysis was conducted using R (version 4.1.0) and CytoScape software. The measurement data underwent normal distribution and variance chi-square tests, with the results presented as mean ± standard deviation (SD). Group comparisons were performed using T-tests or Mann-Whitney U tests. Count data were represented as n (%), and group comparisons were carried out using the Chi-square test or Fisher's exact test. A two-sided P-value < 0.05 was considered statistically significant. For comparisons involving more than three groups, ANOVA or Kruskal-Wallis H tests were utilized.

#### Reference

1. Care CDSNOoBPHSPfPD: National technical guidelines for the prevention and treatment of diabetic kidney disease in primary care (2023). 2023

2. Committee ADAPP: 2. Diagnosis and Classification of Diabetes: Standards of Care in Diabetes-2024. 2024

3. Zhang JQ, Su BH, Zhang J, Guo XH: Expert consensus on early prediction and diagnosis of diabetic kidney disease. *Zhonghua nei ke za zhi,* 60**:** 522-532, 2021 10.3760/cma.j.cn112138-20200603-00550

4. Jiang W, Wang J, Shen X, Lu W, Wang Y, Li W, et al.: Establishment and Validation of a Risk Prediction Model for Early Diabetic Kidney Disease Based on a Systematic Review and Meta-Analysis of 20 Cohorts. 2020

5. Li JJ, Zhao SP, Zhao D, Lu GP, Peng DQ, Liu J, et al.: 2023 Chinese guideline for lipid management. 2023

6. Diseases CDSCSoR: Chinese guidelines for diagnosis and treatment of diabetic kidney disease. *Chinese Journal of Nephrology,* 11, 2019 10.3760/cma.j.issn.1674?5809.2019.01.004
