# Supplementary material for: Machine learning‐based multi‐omics models for diagnostic classification and risk stratification in diabetic kidney disease
Source: Clin Transl Med. 2025 Jan 8;15(1):e70133. doi: 10.1002/ctm2.70133 (PMC11707431; doi:10.1002/ctm2.70133)
Supplement: Supplementary file 2 — APPENDIX 2 Inventory of Supporting Information. This supplement provides additional figures and tables containing more detailed results for this study. [file CTM2-15-e70133-s002.docx]

**Appendix 2: Inventory of Supplemental Information**

This supplement provides additional figures and tables containing more detailed results for this study.

**[I. Supplementary Tables](#_Toc13505)**

**[Section 1.1.](#_Toc12510)** [Baseline Characteristics of the Multi-omics Detection 6](#_Toc12510)

**[Supplementary Table 1.](#_Toc26194)** [Baseline characteristics of the multi-omics detection 7](#_Toc26194)

**[Section 1.2.](#_Toc11840)** [Pre-screening and Composite Scores for Protein Markers 9](#_Toc11840)

**[Supplementary Table 2.](#_Toc5127)** [Pre-screening and composite scores for protein markers 9](#_Toc5127)

**[Section 1.3.](#_Toc1715)** [Information on Urine Proteomics Databases 10](#_Toc1715)

**Supplementary Table 3.** [Information on urine proteomics databases 10](#_Toc27802)

**[Section 1.4.](#_Toc17237)** [Expression Trend Validation of Protein Markers in the Combined Dataset 10](#_Toc17237)

**[Supplementary Table 4.](#_Toc8004)** [Summary of logFC and P-values of pre-screened protein markers in external databases 10](#_Toc8004)

**[Section 1.5.](#_Toc16067)** [Validation of Predictive Performance of Single Protein Markers using Random Forest Algorithm 11](#_Toc16067)

**[Supplementary Table 5.](#_Toc2896)** [Assessment of predictive performance of single protein markers 12](#_Toc2896)

**[Section 1.6.](#_Toc5562)** [Evaluate and Pre-screen Metabolomic Biomarkers from Multiple Scoring. 13](#_Toc5562)

**[Supplementary Table 6.](#_Toc4794)** [Multiple perspectives evaluation and pre-screening of metabolomics biomarkers 13](#_Toc4794)

**[Supplementary Table 7.](#_Toc16813)** [Biological significance of screened metabolites 15](#_Toc16813)

**[Section 1.7.](#_Toc4842)** [Dual Screening of Metabolic Markers by Two-sample Mendelian Randomization. 19](#_Toc4842)

**[Supplementary Table 8.](#_Toc21792)** [Information on mendelian randomization genetic data 19](#_Toc21792)

**[Supplementary Table 9.](#_Toc15399)** [Genetic associations of metabolic markers with risk of diabetic kidney disease 21](#_Toc15399)

**[Section 1.8.](#_Toc20426)** [Evaluating the Prediction Performance of Metabolic Markers based on the Random Forest Algorithm 23](#_Toc20426)

**[Supplementary Table 10.](#_Toc1455)** [Evaluating the prediction performance of metabolic markers based on the random forest algorithm 23](#_Toc1455)

**[Section 1.9.](#_Toc28869)** [Number and Percentage of Differential Lipid Subclasses 23](#_Toc28869)

**[Supplementary Table 11.](#_Toc11170)** [Number and percentage of differential lipid subclasse 23](#_Toc11170)

**[Section 1.10.](#_Toc24415)** [Comprehensive Score of Lipidomics Biomarkers. 24](#_Toc24415)

**[Supplementary Table 12.](#_Toc19738)** [Comprehensive score of lipidomics biomarkers. 25](#_Toc19738)

**[Section 1.11.](#_Toc1861)** [Secondary Screening for Lipid Markers Performed by Two-sample Mendelian Randomization. 25](#_Toc1861)

**[Supplementary Table 13.](#_Toc13348)** [Details of mendelian randomized genetic agent datasets 25](#_Toc13348)

**[Supplementary Table 14a.](#_Toc11031)** [Pre-screening for genetic associations of lipid metabolites with risk of DKD 27](#_Toc11031)

**[Supplementary Table 14b.](#_Toc24191)** [Pre-screening for genetic associations of lipid metabolites with risk of DKD 28](#_Toc24191)

**[Section 1.12.](#_Toc2226)** [Baseline demographic characteristics and biochemical indicators for patients in the cross-sectional study 30](#_Toc2226)

**[Supplementary Table 15.](#_Toc21830)** [Baseline demographic characteristics and biochemical indicators for patients in the cross-sectional study 31](#_Toc21830)

**Supplementary Table 16.** Baseline demographic characteristics of the training

and testing group 31

**[Section 1.13.](#_Toc1200)** [Inclusion Indicators for Different Combinations of Classification Models 35](#_Toc1200)

**[Supplementary Table 17.](#_Toc4848)** [Summary of inclusion indicators for different combinations of classification models 35](#_Toc4848)

**[Section 1.14.](#_Toc16102)** [Summary of Evaluation Indicators for Classification Models 35](#_Toc16102)

**[Supplementary Table 18.](#_Toc29260)** [Summary of evaluation indicators for classification models 36](#_Toc29260)

**[Section 1.15.](#_Toc16372)** [Baseline Data of the Training and Testing Group for the Risk-prognostic Models. 39](#_Toc16372)

**[Supplementary Table 19.](#_Toc13922)** [Baseline demographic characteristics and biochemical indicators for the training and testing Groups 39](#_Toc13922)

**[Section 1.16.](#_Toc12312)** [Establish and Validate COX risk Prognostic Models based on Multi-omics Data 41](#_Toc12312)

**[Supplementary Table 20.](#_Toc31331)** [Baseline demographic characteristics and biochemical indicators for the prospective cohort study 43](#_Toc31331)

**Supplementary Table 21.** COX proportional risk regression analysis of multiomics indicators and outcome events 43

**[Supplementary Table 22.](#_Toc31331)** [Indicators used in prognostic risk prediction models 43](#_Toc31331)

### [II. Supplementary Figures](#_Toc10845)

**[Section 2.1.](#_Toc18923)** [Results of Quality Control for Proteomics 44](#_Toc18923)

**[Supplementary Figure 1.](#_Toc4620)** [Evaluation of QC samples 44](#_Toc4620)

**[Supplementary Figure 2.](#_Toc4014)** [Intergroup sample identification and evaluation of the DIA system 45](#_Toc4014)

**[Supplementary Figure 3.](#_Toc26151)** [Histogram of DIA identification and quantification results 46](#_Toc26151)

**[Section 2.2.](#_Toc16463)** [Differential Protein Analysis between Subgroups 46](#_Toc16463)

**[Supplementary Figure 4.](#_Toc30224)** [Volcano plots 46](#_Toc30224)

**[Supplementary Figure 5.](#_Toc12626)** [Statistics on the number of up-regulated and down-regulated differential proteins compared between different subgroups 47](#_Toc12626)

**[Section 2.3.](#_Toc11880)** [Overlap in Differential Protein Identification between Groups 47](#_Toc11880)

**[Supplementary Figure 6.](#_Toc7488)** [Overlap of differential protein identification between different groups and Upset plots 48](#_Toc7488)

**[Section 2.4.](#_Toc1629)** [Heat Map and Functional Analysis of Differential Proteins 48](#_Toc1629)

**[Supplementary Figure 7.](#_Toc12872)** [Top 50 differential proteins display and functional analysis 49](#_Toc12872)

**[Supplementary Figure 8.](#_Toc8238)** [Top 50 differential proteins display and functional analysis 50](#_Toc8238)

**[Supplementary Figure 9.](#_Toc19257)** [Top 50 differential proteins display and functional analysis 51](#_Toc19257)

**[Section 2.5.](#_Toc20180)** [Total Up- and Down-regulated Differential Proteins 52](#_Toc20180)

**[Supplementary Figure 10.](#_Toc19711)** [Heatmap and functional analysis of shared up-regulated proteins among multiple groups 52](#_Toc19711)

**[Supplementary Figure 11.](#_Toc12794)** [Heatmap and functional analysis of shared down-regulated proteins among multiple groups 53](#_Toc12794)

**[Section 2.6.](#_Toc13635)** [Mfuzz Temporal Expression Clustering Analysis and Functional Clustering 54](#_Toc13635)

**[Supplementary Figure 12.](#_Toc25258)** [Mfuzz protein time series expression trend analysis 54](#_Toc25258)

**[Supplementary Figure 13.](#_Toc2790)** [KEGG and GO analysis of Cluster 1 and 5 55](#_Toc2790)

**[Section 2.7.](#_Toc15951)** [Statistical Analysis of Molecular Characterization and Organ Origin of Differential Proteins 56](#_Toc15951)

**[Supplementary Figure 14.](#_Toc10088)** [Tissue and organ sources and classification of differential proteins in different stages 56](#_Toc10088)

**[Supplementary Figure 15.](#_Toc2829)** [Tissue and organ sources and classification of 3 or more groups of shared up-regulated and down-regulated differential proteins 57](#_Toc2829)

**[Supplementary Figure 16.](#_Toc18560)** [Subcellular localization of differential proteins at different stages 58](#_Toc18560)

**[Section 2.8.](#_Toc18289)** [Weighted Gene Co-expression Network Analysis 59](#_Toc18289)

**[Supplementary Figure 17.](#_Toc4971)** [Weighted gene co-expression network analysis 59](#_Toc4971)

**[Section 2.9.](#_Toc18698)** [Enrichment Analysis of Key Transcription Factors and Hub gene Screening 60](#_Toc18698)

**[Supplementary Figure 18.](#_Toc16315)** [Screening of transcription factors and hub genes 60](#_Toc16315)

**[Supplementary Figure 19.](#_Toc10349)** [Screening of transcription factors and hub genes 61](#_Toc10349)

**[Supplementary Figure 20.](#_Toc3510)** [Screening of transcription factors and hub genes 62](#_Toc3510)

**[Supplementary Figure 21.](#_Toc11950)** [Screening of transcription factors and hub genes 63](#_Toc11950)

**[Section 2.10.](#_Toc3198)** [Evaluation and Pre-screening of Proteomics Biomarkers from Multiple Perspectives 64](#_Toc3198)

**[Supplementary Figure 22.](#_Toc22361)** [Multidimensional evaluation and screening of proteomic features 64](#_Toc22361)

**[Supplementary Figure 23.](#_Toc5780)** [The correlation between pre-screened proteins and expression trends across subgroups in our cohort. 66](#_Toc5780)

**[Section 2.11.](#_Toc21355)** [Validation of Protein Markers in the Combined Dataset 67](#_Toc21355)

**[Supplementary Figure 24.](#_Toc24449)** [Validation of protein marker expression trends in the combined dataset. 67](#_Toc24449)

**[Supplementary Figure 25.](#_Toc7389)** [Predictive performance of pre-screened proteins based on the Random Forest algorithm in the public database of urinary proteomics. 68](#_Toc7389)

**[Section 2.12.](#_Toc928)** [The Results of the Quality Control Analysis in Metabolomics Analysis 69](#_Toc928)

**[Supplementary Figure 26.](#_Toc26558)** [QC sample evaluation for metabolomics. 69](#_Toc26558)

**[Supplementary Figure 27.](#_Toc21345)** [QC sample correlation and fluctuation range measurement. 70](#_Toc21345)

**[Section 2.13.](#_Toc25843)** [Differential Metabolite Analysis among Different Groups. 71](#_Toc25843)

**[Supplementary Figure 28.](#_Toc7550)** [Volcano plots 71](#_Toc7550)

**[Supplementary Figure 29.](#_Toc11279)** [Statistics on the number of up-regulated and down-regulated differential metabolites compared between different groups. 72](#_Toc11279)

**[Section 2.14.](#_Toc6132)** [Overlap of Differential Metabolite Identifications among Different Groups. 73](#_Toc6132)

**[Supplementary Figure 30.](#_Toc3306)** [Overlap of differential metabolite identification between different groups. 73](#_Toc3306)

**[Section 2.15.](#_Toc17864)** [Heat Map and Functional Analysis of Differential Metabolites between Different Groups. 73](#_Toc17864)

**[Supplementary Figure 31.](#_Toc20185)** [Display and functional analysis of the Top50 differential metabolite. 74](#_Toc20185)

**[Supplementary Figure 32.](#_Toc26586)** [Display and functional analysis of the Top50 differential metabolite. 75](#_Toc26586)

**[Supplementary Figure 33.](#_Toc21169)** [Display and functional analysis of the Top50 differential metabolite. 76](#_Toc21169)

**[Section 2.16.](#_Toc14667)** [Differential Metabolites among Multiple Groups 77](#_Toc14667)

**[Supplementary Figure 34.](#_Toc30218)** [Heat map and functional analysis of shared differential metabolites between multiple groups 77](#_Toc30218)

**[Section 2.17.](#_Toc31828)** [Mfuzz Temporal Expression Cluster Analysis 78](#_Toc31828)

**[Supplementary Figure 35.](#_Toc18770)** [Mfuzz Time series expression trend analysis 78](#_Toc18770)

**[Section 2.18.](#_Toc17012)** [Statistical Analysis of Molecular Characteristics and Organ Sources of Differential Metabolites. 79](#_Toc17012)

**[Supplementary Figure 36.](#_Toc30637)** [Tissue and organ-specific sources of differential metabolites in different stages of DKD 79](#_Toc30637)

**[Supplementary Figure 37.](#_Toc29529)** [Subcellular localization of differential metabolites in different stages of DKD 80](#_Toc29529)

**[Supplementary Figure 38.](#_Toc30397)** [Chemical classification and assignment statistics of differential metabolites in different stages of DKD 81](#_Toc30397)

**[Section 2.19.](#_Toc28677)** [Common Pathway Enrichment Changes and Metabolic Pathway Changes in Different DKD Stages. 82](#_Toc28677)

**[Supplementary Figure 39.](#_Toc1478)** [Common RaMP pathway enrichment changes and metabolic pathways enriched in different DKD stages 82](#_Toc1478)

**[Section 2.20.](#_Toc23527)** [WGCNA Analysis 83](#_Toc23527)

**[Supplementary Figure 40.](#_Toc32455)** [Weighted gene co-expression network analysis 83](#_Toc32455)

**[Section 2.21.](#_Toc9228)** [Enrichment Analysis of Differential Protein Combined Metabolome. 84](#_Toc9228)

**[Supplementary Figure 41.](#_Toc20861)** [Differential protein-metabolite-disease network relationship diagram 84](#_Toc20861)

**[Supplementary Figure 42.](#_Toc23704)** [Differential protein-metabolite-disease network relationship diagram 85](#_Toc23704)

**[Supplementary Figure 43.](#_Toc32626)** [Differential protein-metabolite-disease network relationship diagram 86](#_Toc32626)

**[Section 2.22.](#_Toc3862)** [O2PLSDA Analysis 87](#_Toc3862)

**[Supplementary Figure 44.](#_Toc27007)** [O2PLSDA analysis of differential metabolites combined with differential proteins (TOP50) 87](#_Toc27007)

**[Section 2.23.](#_Toc10357)** [Evaluate and Pre-screen Metabolomic Biomarkers from Multiple Perspectives. 88](#_Toc10357)

**[Supplementary Figure 45.](#_Toc623)** [Using random forest to evaluate and screen metabolomics biomarkers. 88](#_Toc623)

**[Supplementary Figure 46.](#_Toc27552)** [The correlation between pre-screened metabolites and expression trends across subgroups in our cohort. 89](#_Toc27552)

**[Section 2.24.](#_Toc22434)** [Verify the Expression Trend of Metabolic Markers through the Metabolomics Database. 90](#_Toc22434)

**[Supplementary Figure 47.](#_Toc13838)** [Expression trend of metabolites screened based on metabolite dataset verification 90](#_Toc13838)

**[Supplementary Figure 48.](#_Toc10209)** [Evaluate the prediction performance of metabolites in external databases based on the random forest algorithm. 91](#_Toc10209)

**[Section 2.25.](#_Toc3534)** [Quality Control Analysis for Lipidomics 92](#_Toc3534)

**[Supplementary Figure 49.](#_Toc16840)** [QC sample evaluation. 92](#_Toc16840)

**[Section 2.26.](#_Toc27851)** [Differential Lipids Analysis between Different Groups. 93](#_Toc27851)

**[Supplementary Figure 50.](#_Toc28260)** [Volcano plots 93](#_Toc28260)

**[Supplementary Figure 51.](#_Toc7008)** [Statistics on the number of up-regulated and down-regulated differential lipids between different groups 94](#_Toc7008)

**[Section 2.27.](#_Toc4613)** [The Overlap of Differential Lipids Identification between Different Groups 95](#_Toc4613)

**[Supplementary Figure 52.](#_Toc15485)** [Overlap and Upset diagram of differential lipid identification between different groups. 95](#_Toc15485)

**[Section 2.28.](#_Toc15438)** [Lipid Level and Quantity Statistics. 96](#_Toc15438)

**[Supplementary Figure 53.](#_Toc32737)** [Statistics of overall lipid levels and differential lipid types. 96](#_Toc32737)

**[Section 2.29.](#_Toc27140)** [Differential Lipid Heat Map and Functional Analysis between Different Groups 97](#_Toc27140)

**[Supplementary Figure 54.](#_Toc21304)** [Differential metabolite and functional enrichment analysis. 97](#_Toc21304)

**[Supplementary Figure 55.](#_Toc3637)** [Differential metabolites and functional enrichment analysis. 98](#_Toc3637)

**[Supplementary Figure 56.](#_Toc28961)** [Differential metabolite and functional enrichment analysis. 99](#_Toc28961)

**[Section 2.30.](#_Toc26022)** [Mfuzz Temporal Expression Clustering Analysis 99](#_Toc26022)

**[Supplementary Figure 57.](#_Toc25957)** [Mfuzz lipidomics time series expression trend analysis and clustering group classification 100](#_Toc25957)

**[Section 2.31.](#_Toc28337)** [Weighted Co-expression Network Analysis. 101](#_Toc28337)

**[Supplementary Figure 58.](#_Toc6118)** [Weighted gene co-expression network analysis 101](#_Toc6118)

**[Section 2.32.](#_Toc28909)** [Chain Saturation and Length Analysis. 102](#_Toc28909)

**[Supplementary Figure 59.](#_Toc15379)** [Chain saturation analysis of the main different lipids 102](#_Toc15379)

**[Supplementary Figure 60.](#_Toc7010)** [Chain length analysis of the main different lipids 103](#_Toc7010)

**[Section 2.33.](#_Toc12511)** [Evaluation and Pre-screening of Lipidomics Biomarkers from Multiple Perspectives. 104](#_Toc12511)

**[Supplementary Figure 61.](#_Toc13593)** [Use random forest algorithm to evaluate and screen lipidomic features 104](#_Toc13593)

**[Supplementary Figure 62.](#_Toc32142)** [The correlation between pre-screened metabolites and expression trends across subgroups in our cohort. 105](#_Toc32142)

**Section 2.34.** Discussion of the biological significance of screening biomarkers. 107

### Section 1.1. Baseline Characteristics of the Multi-omics Detection

A total of 6 patients each from the healthy control (HC) group, T2DM group, HR-DKD group, and DKD group were sampled, and serum and urine samples were collected to complete urine proteomics and metabolomics, and blood lipidomics, and the detailed demographic information table is shown in sTable 1. Age, gender, marital status, and ethnicity were matched among the groups (*P* > 0.05). Baseline conditions such as lifestyle risk factors (smoking, alcohol consumption), duration of T2DM, HbA1c, history of hypertension, and renal function indices (eGFR, ACR, and urinary protein testing) were significantly different (*P* < 0.05), which was in line with the requirements of this study for the samples to be sent for detection.

#### **Supplementary Table 1. Baseline characteristics of the multi-omics detection**

| Characteristics | HC group | T2DM group | HR-DKD group | DKD group | *P* value |
| --- | --- | --- | --- | --- | --- |
| N | 6 | 6 | 6 | 6 | 1.000 |
| Ethnicity (Han), N | 6 | 6 | 6 | 6 | 1.000 |
| Marital status (married), N | 6 | 6 | 6 | 6 | 1.000 |
| Sex (male), N | 4 | 4 | 4 | 4 | 1.000 |
| Age, years | 56.50 (43.50, 64.25) | 49.00 (32.00, 68.25) | 56.00 (49.50, 58.75) | 56.00 (52.00, 63.00) | 0.970 |
| Hypertension, N | 0 | 1 | 6 | 3 | 0.002 |
| Smoking, N | 0 | 0 | 3 | 2 | 0.078 |
| Drinking, N | 0 | 0 | 3 | 2 | 0.078 |
| Duration of T2DM, years | 0 | 1.38 (0.56, 2.75) | 12.00 (10.00, 15.50) | 12.00 (7.75, 18.50) | <0.001 |
| BMI, kg/m^2^ | 23.85 (0.66) | 23.55 (2.29) | 26.56 (2.21) | 26.36 (3.60) | 0.387 |
| HbA1c, % | 5.84 (0.96) | 6.92 (0.72) | 8.92 (1.17) | 7.87 (1.86) | 0.005 |
| eGFR, mL/min/1.73m^2^ | 114.90 (11.97) | 113.64 (18.46) | 107.02 (7.45) | 89.50 (21.71) | 0.043 |
| Baseline ACR, mg/g | 2.50 (0.00) | 4.93 (1.94) | 16.80 (4.55) | 268.57 (360.24) | 0.048 |
| NGAL, U/L | 5.42 (3.53, 7.65) | 4.70 (2.60, 5.00) | 8.30 (8.00, 9.16) | 14.25 (13.35, 34.80) | <0.001 |
| RBP, mg/L | 0.02 (0.01, 0.07) | 0.15 (0.15, 0.39) | 0.20 (0.17, 0.26) | 1.44 (0.25, 2.73) | 0.002 |
| UTF, mg/L | 0.25 (0.15, 0.38) | 0.45 (0.45, 1.12) | 0.58 (0.49, 0.82) | 3.13 (0.28, 25.33) | 0.657 |
| Uβ2-MG, mg/L | 0.15 (0.02, 0.24) | 0.13 (0.04, 0.13) | 0.18 (0.10, 0.30) | 0.23 (0.11, 0.30) | 0.437 |
| UIgG, mg/L | 1.03 (0.08, 1.63) | 5.25 (3.75, 6.75) | 4.24 (2.48, 6.73) | 6.39 (3.80, 47.51) | 0.014 |

Non-normally distributed data were statistically described using the median (interquartile spacing), and comparisons between groups were made using a nonparametric test (Kruskal-Wallis rank sum test); normally distributed data were statistically described using the mean±standard deviation, and comparisons between groups were made using analysis of variance; and categorical data were compared using Fisher's exact test.

**Abbreviations:** DKD: diabetic kidney disease; T2DM: type 2 diabetes mellitus; HR-DKD: high-risk diabetic kidney disease; HC: healthy control; BMI: body mass index; HbA1c: glycated haemoglobin; eGFR: estimated glomerular filtration rate; ACR: urine albumin to creatinine ratio; NGAL: N. acetylaminoglucosidase; RBP: retinol-binding protein; UTF: urinary transferrin quantification; β2MG: urinary β2 microglobulin; UIgG: urinary immunoglobulin G.

### Section 1.2. Pre-screening and Composite Scores for Protein Markers

The screened protein markers were assigned a composite score to assess their significance. Proteins with scores equal to or greater than 8 were subjected to composite scoring, which included SERPINA3, SERPINA1, EML4, ETNK2, CADM1, CD300LF, CHMP3, GP1BA, RAC1, CST4, EGF, FBP2, FTL, KNG1, LRG1, MMRN2, SDC4, SERPINA6, SUSd2, TMEM198, TRABD2B, CD63, CSF1R, APOD, EMCN, CHGA, GPLD1.

#### Supplementary Table 2. Pre-screening and composite scores for protein markers

| ID | Frequency of incorporation in feature engineering | Correlation with renal function or grouping | Significant difference | Number of groups with differences | Hub Gene | Total score |
| --- | --- | --- | --- | --- | --- | --- |
| SERPINA1 | 5 | 5 | 0.00026 | 3 | 1 | 10 |
| SERPINA3 | 1 | 5 | 0.0014 | 3 | 1 | 11 |
| EML4 | 3 | 5 | <0.0001 | 1 | 0 | 10 |
| ETNK2 | 1 | 5 | 0.0032 | 3 | 0 | 10 |
| CADM1 | 2 | 5 | <0.0001 | 0 | 1 | 9 |
| CD300LF | 1 | 5 | 0.0098 | 2 | 0 | 9 |
| CHMP3 | 1 | 5 | 0.016 | 2 | 0 | 9 |
| GP1BA | 1 | 5 | 0.021 | 1 | 1 | 9 |
| RAC1 | 0 | 5 | 0.0041 | 2 | 1 | 9 |
| CST4 | 1 | 5 | 0.019 | 1 | 0 | 8 |
| EGF | 0 | 5 | 0.029 | 1 | 1 | 8 |
| FBP2 | 1 | 5 | 0.0025 | 1 | 0 | 8 |
| FTL | 1 | 5 | 0.0051 | 1 | 0 | 8 |
| KNG1 | 0 | 5 | 0.0093 | 1 | 1 | 8 |
| LRG1 | 0 | 5 | 0.021 | 1 | 1 | 8 |
| MMRN2 | 1 | 5 | 0.0022 | 1 | 0 | 8 |
| SDC4 | 1 | 5 | 0.021 | 0 | 1 | 8 |
| SERPINA6 | 0 | 5 | 0.0043 | 1 | 1 | 8 |
| SUSD2 | 1 | 5 | 0.0094 | 1 | 0 | 8 |
| TMEM198 | 1 | 5 | 0.0069 | 1 | 0 | 8 |
| TRABD2B | 1 | 5 | 0.0038 | 1 | 0 | 8 |
| CD63 | 0 | 5 | 0.0073 | 1 | 1 | 8 |
| CSF1R | 0 | 5 | 0.040 | 1 | 1 | 8 |
| APOD | 0 | 5 | 0.029 | 1 | 1 | 8 |
| EMCN | 0 | 5 | 0.026 | 1 | 1 | 8 |
| CHGA | 0 | 5 | 0.025 | 1 | 1 | 8 |
| GPLD1 | 0 | 5 | 0.002 | 1 | 1 | 8 |

### Section 1.3. **Information on Urine Proteomics Databases**

Secondary screening and validation of 27 proteins were conducted by analyzing five urine proteomics datasets: PXD017213, PXD018996, PXD012413, PXD008683, and PXD016571. The study included a total of 421 participants, with 208 classified as normal (NC), 158 as diabetic (DM), and 55 as DKD patients. Detailed information on the specific subgroups and participant numbers can be found in sTables 3.

#### Supplementary Table 3. **Information on urine proteomics databases**

| Database ID | Year | Total number of individuals | Groups | Number of proteins detected | Region |
| --- | --- | --- | --- | --- | --- |
| PXD017213 | 2020 | 30 | DM vs NC | 2294 | Canada |
| PXD018996 | 2023 | 107 | DKD vs NC | 1744 | China |
| PXD012413 | 2017 | 220 | DM vs NC | 2015 | America |
| PXD016571 | 2020 | 54 | DKD vs DM | 1211 | Korea |
| PXD008683 | 2018 | 10 | DM vs NC | 1774 | Japan |

### Section 1.4. **Expression Trend Validation of Protein Markers in the Combined Dataset**

To ensure the consistency of the expression trends of the selected metrics, LogFC and P-values of the pre-screened protein markers in each database were further calculated. A total of 25 metrics were confirmed. Some of these proteins, such as EGF, LRG1, CD63, and SERPINA1, have been previously identified as DKD biomarkers.

#### Supplementary Table 4. Summary of logFC and P-values of pre-screened protein markers in external databases

|  | PXD017213 | | PXD018996 | | PXD012413 | | PXD016571 | | PXD008683 | |
| --- | --- | --- | --- | --- | --- | --- | --- | --- | --- | --- |
|  | logFC | P.Value | logFC | P.Value | logFC | P.Value | logFC | P.Value | logFC | P.Value |
| SERPINA1 | 0.386 | 0.250 | 3.916 | <0.001 | 0.489 | 0.503 | 3.941 | <0.001 | -1.491 | 0.152 |
| SERPINA3 | 0.956 | 0.004 | 0.302 | 0.627 | 1.333 | 0.154 | 3.281 | <0.001 | -1.095 | 0.306 |
| EML4 | 1.527 | 0.320 | . | . | . | . | . | . | . | . |
| ETNK2 | . | . | . | . | . | . | . | . | . | . |
| CADM1 | -0.143 | 0.676 | -26.201 | <0.001 | -1.136 | 0.553 | 2.162 | 0.028 | -0.335 | 0.731 |
| CD300LF | -3.154 | 0.470 | -11.318 | <0.001 | 0.447 | 0.666 | 2.381 | 0.082 | 4.202 | 0.561 |
| CHMP3 | -1.527 | 0.320 | . | . | . | . | . | . | -4.611 | 0.318 |
| GP1BA | 11.870 | 0.001 | -4.418 | 0.023 | 3.349 | 0.001 | 6.804 | 0.014 | 4.464 | 0.575 |
| RAC1 | -0.354 | 0.887 | -22.038 | <0.001 | -1.084 | 0.524 | -0.776 | 0.650 | -11.067 | 0.074 |
| CST4 | 4.095 | 0.126 | -4.258 | 0.016 | 4.288 | 0.001 | -0.784 | 0.439 | 4.469 | 0.532 |
| EGF | -0.302 | 0.282 | -23.273 | <0.001 | -0.210 | 0.120 | 1.057 | 0.581 | -1.458 | 0.128 |
| FBP2 | -5.612 | 0.225 | -0.346 | 0.493 | -0.128 | 0.343 | . | . | -6.580 | 0.364 |
| FTL | . | . | 1.315 | 0.042 | 0.156 | 0.247 | 0.417 | 0.869 | -2.061 | 0.070 |
| KNG1 | -0.344 | 0.178 | -2.776 | <0.001 | -0.129 | 0.339 | 2.830 | <0.001 | -0.988 | 0.267 |
| LRG1 | 2.195 | 0.000 | 0.033 | 0.958 | 0.578 | <0.001 | 3.158 | 0.000 | -0.847 | 0.456 |
| MMRN2 | 0.009 | 0.979 | -20.899 | <0.001 | -0.081 | 0.548 | 2.311 | 0.190 | -2.726 | 0.062 |
| SDC4 | -4.799 | 0.163 | -24.402 | <0.001 | -0.114 | 0.397 | -1.320 | 0.474 | -1.871 | 0.131 |
| SERPINA6 | 0.519 | 0.116 | -0.176 | 0.763 | 0.411 | 0.002 | 2.404 | 0.000 | -6.738 | 0.206 |
| SUSD2 | 0.096 | 0.766 | -24.279 | <0.001 | 0.154 | 0.253 | 9.204 | 0.000 | -1.786 | 0.123 |
| TMEM198 | . | . | . | . | -0.128 | 0.343 | -1.800 | 0.301 | -10.244 | 0.116 |
| TRABD2B | . | . | . | . | . | . | . | . | . | . |
| CD63 | . | . | -12.577 | <0.001 | -0.128 | 0.343 | . | . | -14.887 | 0.029 |
| CSF1R | -0.190 | 0.508 | -3.294 | 0.014 | 0.102 | 0.448 | 7.677 | 0.001 | . | . |
| APOD | -0.063 | 0.825 | -6.521 | <0.001 | 0.228 | 0.090 | 3.034 | 0.016 | -2.189 | 0.054 |
| EMCN | 1.687 | 0.319 | -5.186 | 0.035 | -0.035 | 0.796 | . | . | . | . |
| CHGA | 1.725 | 0.632 | -9.956 | <0.001 | -0.144 | 0.284 | -1.270 | 0.411 | . | . |
| GPLD1 | 0.492 | 0.918 | . | . | 0.143 | 0.290 | 1.315 | 0.614 | . | . |

### Section 1.5. **Validation of Predictive Performance of Single Protein Markers using Random Forest Algorithm**

The impact of protein markers on 3 subgroups (NC, DM, DKD) was analyzed using the random forest algorithm to determine their classification effect. Various metrics such as accuracy, sensitivity, specificity, precision, recall, and AUC were used to assess the classification prediction value. The model's accuracy was demonstrated through the confusion matrix and ROC curves. Analysis revealed that SERPINA1, CADM1, CD300LF, GP1BA, RAC1, CST4, MMRN2, SDC4, SUSD2, and APOD had categorical AUCs above 0.6. The mean values for each metric were also above 0.6, with values of 0.623, 0.771, 0.852, 0.890, 0.878, 0.846, 0.820, 0.764, 0.827, and 0.752.

#### Supplementary Table **5. Assessment of predictive performance of single protein markers**

| Gene ID | Accuracy | | AUC | | | Sensitivity | | | Specificity | | | Precision | | | Recall | | | Average |
| --- | --- | --- | --- | --- | --- | --- | --- | --- | --- | --- | --- | --- | --- | --- | --- | --- | --- | --- |
|  |  | 95% CI | NC | DM | DKD | NC | DM | DKD | NC | DM | DKD | NC | DM | DKD | NC | DM | DKD |  |
| SERPINA1 | 0.56 | (0.47, 0.65) | 0.67 | 0.63 | 0.67 | 0.70 | 0.35 | 0.67 | 0.51 | 0.77 | 0.95 | 0.55 | 0.50 | 0.71 | 0.70 | 0.35 | 0.67 | 0.623 |
| SERPINA3 | 0.43 | (0.34, 0.52) | 0.59 | 0.56 | 0.55 | 0.49 | 0.37 | 0.39 | 0.57 | 0.63 | 0.87 | 0.49 | 0.39 | 0.33 | 0.49 | 0.37 | 0.39 | 0.494 |
| CADM1 | 0.72 | (0.63, 0.80) | 0.88 | 0.81 | 0.93 | 0.78 | 0.61 | 0.78 | 0.78 | 0.82 | 0.93 | 0.75 | 0.70 | 0.67 | 0.79 | 0.61 | 0.78 | 0.771 |
| CD300LF | 0.83 | (0.75, 0.89) | 0.96 | 0.96 | 0.83 | 0.81 | 0.88 | 0.78 | 0.91 | 0.91 | 0.92 | 0.88 | 0.86 | 0.63 | 0.81 | 0.88 | 0.78 | 0.852 |
| GP1BA | 0.86 | (0.79, 0.92) | 0.98 | 0.99 | 0.98 | 0.81 | 0.94 | 0.83 | 0.93 | 0.99 | 0.90 | 0.90 | 0.98 | 0.58 | 0.81 | 0.94 | 0.83 | 0.891 |
| RAC1 | 0.82 | (0.74, 0.89) | 0.92 | 0.90 | 0.99 | 0.84 | 0.73 | 0.99 | 0.81 | 0.88 | 0.99 | 0.79 | 0.80 | 0.99 | 0.84 | 0.77 | 0.99 | 0.878 |
| CST4 | 0.82 | (0.74, 0.88) | 0.94 | 0.95 | 0.85 | 0.77 | 0.86 | 0.83 | 0.94 | 0.91 | 0.89 | 0.92 | 0.86 | 0.55 | 0.77 | 0.86 | 0.83 | 0.847 |
| EGF | 0.50 | (0.41, 0.59) | 0.65 | 0.66 | 0.72 | 0.60 | 0.39 | 0.50 | 0.51 | 0.71 | 0.93 | 0.51 | 0.46 | 0.56 | 0.60 | 0.39 | 0.50 | 0.574 |
| KNG1 | 0.50 | (0.41, 0.59) | 0.62 | 0.54 | 0.56 | 0.60 | 0.45 | 0.33 | 0.60 | 0.68 | 0.90 | 0.56 | 0.48 | 0.35 | 0.60 | 0.45 | 0.34 | 0.535 |
| LRG1 | 0.60 | (0.51, 0.68) | 0.83 | 0.82 | 0.66 | 0.79 | 0.57 | 0.05 | 0.61 | 0.81 | 0.91 | 0.63 | 0.67 | 0.09 | 0.79 | 0.57 | 0.06 | 0.591 |
| MMRN2 | 0.77 | (0.69, 0.84) | 0.89 | 0.90 | 0.94 | 0.82 | 0.69 | 0.83 | 0.76 | 0.91 | 0.95 | 0.75 | 0.83 | 0.75 | 0.82 | 0.69 | 0.83 | 0.821 |
| SDC4 | 0.70 | (0.61, 0.78) | 0.83 | 0.89 | 0.91 | 0.75 | 0.61 | 0.77 | 0.66 | 0.91 | 0.93 | 0.65 | 0.81 | 0.67 | 0.75 | 0.61 | 0.78 | 0.764 |
| SERPINA6 | 0.48 | (0.39, 0.57) | 0.71 | 0.68 | 0.79 | 0.51 | 0.51 | 0.28 | 0.63 | 0.68 | 0.85 | 0.54 | 0.51 | 0.24 | 0.51 | 0.51 | 0.56 | 0.562 |
| SUSD2 | 0.77 | (0.68, 0.84) | 0.87 | 0.85 | 0.94 | 0.77 | 0.69 | 0.94 | 0.78 | 0.83 | 0.99 | 0.75 | 0.72 | 0.94 | 0.77 | 0.69 | 0.94 | 0.828 |
| APOD | 0.73 | (0.64, 0.80) | 0.89 | 0.88 | 0.91 | 0.86 | 0.65 | 0.50 | 0.74 | 0.87 | 0.93 | 0.74 | 0.76 | 0.56 | 0.86 | 0.65 | 0.50 | 0.752 |
| EML4* | 0.51 | (0.42, 0.60)* | 0.64 | 0.67 | 0.86 | 0.52 | 0.48 | 0.60 | 0.57 | 0.74 | 0.87 | 0.54 | 0.57 | 0.30 | 0.52 | 0.48 | 0.60 | 0.592 |
| CHMP3* | 0.61 | (0.52, 0.70)* | 0.74 | 0.71 | 0.92 | 0.71 | 0.50 | 0.60 | 0.60 | 0.78 | 0.94 | 0.63 | 0.63 | 0.46 | 0.71 | 0.50 | 0.60 | 0.665 |
| FBP2* | 0.90 | (0.83, 0.95)* | 0.98 | 0.87 | 0.99 | 0.90 | 0.88 | 0.99 | 0.90 | 0.93 | 0.99 | 0.90 | 0.90 | 0.91 | 0.90 | 0.88 | 0.99 | 0.926 |
| FTL* | 0.91 | (0.84, 0.95)* | 0.98 | 0.87 | 0.99 | 0.97 | 0.82 | 0.99 | 0.85 | 0.97 | 0.99 | 0.86 | 0.95 | 0.99 | 0.97 | 0.82 | 0.99 | 0.933 |
| TMEM198* | 0.92 | (0.86, 0.97)* | 0.99 | 0.91 | 0.99 | 0.97 | 0.88 | 0.90 | 0.92 | 0.97 | 0.98 | 0.92 | 0.96 | 0.82 | 0.97 | 0.88 | 0.90 | 0.930 |
| CD63* | 0.87 | (0.80, 0.93)* | 0.99 | 0.90 | 0.99 | 0.97 | 0.74 | 0.99 | 0.88 | 0.97 | 0.94 | 0.89 | 0.95 | 0.63 | 0.97 | 0.74 | 0.99 | 0.901 |
| EMCN* | 0.89 | (0.82, 0.94)* | 0.99 | 0.87 | 0.96 | 0.93 | 0.84 | 0.60 | 0.90 | 0.88 | 0.98 | 0.90 | 0.84 | 0.75 | 0.93 | 0.84 | 0.60 | 0.856 |
| CHGA* | 0.86 | (0.79, 0.92)* | 0.97 | 0.91 | 0.99 | 0.88 | 0.88 | 0.99 | 0.93 | 0.90 | 0.98 | 0.93 | 0.86 | 0.83 | 0.88 | 0.88 | 0.99 | 0.916 |
| GPLD1* | 0.93 | (0.87, 0.97)* | 0.99 | 0.90 | 0.89 | 0.96 | 0.90 | 0.90 | 0.92 | 0.97 | 0.99 | 0.92 | 0.96 | 0.90 | 0.97 | 0.90 | 0.90 | 0.931 |

*Indicates calculation using imputation data, the results are for reference only, EML4 is missing 4 sets of data, CHMP3 is missing 3 sets of data, TMEM198, EMCN, GPLD1 is missing 2 sets of data, CD63, FTL, CHGA, FBP2 is missing 1 set of data, so the results of the indicators with no missing data are prioritized. AUC: area under the curve.

### Section 1.6. Evaluate and Pre-screen Metabolomic Biomarkers from Multiple **Scoring**.

Screening of potential metabolomics biomarkers was conducted using multiple levels and methods. A comprehensive scoring of metabolites was performed to determine their importance. Among the top 30 metabolites with high comprehensive scores are L-glutamic acid dimethyl ester (DLG), Bisphenol E, 3-nitrobenzenesulfonic acid, Trans-3'-hydroxycotinine, 2-hydroxyatorvastatin lactone, among others.

#### Supplementary Table 6. Multiple scoring evaluation and pre-screening of metabolomics biomarkers

| Metabolites ID | Decision tree algorithm^1^ | Feature importance | Decision tree algorithm^2^ | Random forest algorithm | Correlation coefficient > 0.7 with renal function and grouping | Number of groups with differences | O2PLSDA TOP 100 | Total score |
| --- | --- | --- | --- | --- | --- | --- | --- | --- |
| L-glutamic acid, dimethyl ester | 1 | 1 | 1 | 1 | 2 | 3 | 0 | 9 |
| Bisphenol e | 1 | 1 | 1 | 1 | 3 | 1 | 0 | 8 |
| 3-nitrobenzenesulfonic acid | 1 | 0 | 0 | 0 | 2 | 4 | 1 | 8 |
| Trans-3'-hydroxycotinine | 1 | 0 | 0 | 1 | 2 | 2 | 1 | 7 |
| 2-hydroxyatorvastatin lactone | 0 | 1 | 0 | 0 | 2 | 3 | 0 | 6 |
| 1h-indole-1-pentanoic acid | 0 | 1 | 0 | 0 | 2 | 3 | 0 | 6 |
| L-carnitine | 0 | 0 | 0 | 0 | 2 | 4 | 0 | 6 |
| 7,8-dihydrobiopterin | 0 | 1 | 0 | 1 | 3 | 1 | 0 | 6 |
| Hypericin | 0 | 0 | 0 | 1 | 1 | 3 | 0 | 5 |
| M495T296 | 1 | 0 | 0 | 0 | 1 | 3 | 0 | 5 |
| Secoisolariciresinol | 0 | 0 | 1 | 0 | 1 | 3 | 0 | 5 |
| Nonanoic acid | 1 | 0 | 0 | 1 | 2 | 1 | 0 | 5 |
| Tabersonine | 0 | 0 | 0 | 1 | 2 | 2 | 0 | 5 |
| Isoeugenyl acetate | 1 | 0 | 1 | 0 | 0 | 2 | 0 | 4 |
| 1-methylhistidine | 1 | 0 | 0 | 1 | 1 | 1 | 0 | 4 |
| Blood group a trisaccharide | 1 | 0 | 0 | 1 | 1 | 1 | 0 | 4 |
| Hydroxybupropion | 1 | 1 | 0 | 0 | 1 | 1 | 0 | 4 |
| Spinosine | 1 | 0 | 0 | 1 | 1 | 1 | 0 | 4 |
| D-mannitol 1-phosphate | 0 | 0 | 0 | 0 | 1 | 3 | 0 | 4 |
| Nicotine | 0 | 0 | 0 | 0 | 2 | 2 | 1 | 5 |
| Tebufenpyrad | 0 | 0 | 0 | 0 | 2 | 2 | 1 | 5 |
| Stachydrine | 0 | 0 | 0 | 0 | 0 | 2 | 2 | 4 |
| Rhamnetin | 0 | 0 | 0 | 0 | 0 | 3 | 1 | 4 |
| Creatine | 0 | 0 | 0 | 0 | 0 | 1 | 2 | 3 |
| 3-hydroxykynurenine | 0 | 0 | 0 | 0 | 0 | 2 | 1 | 3 |
| Coproporphyrin i | 0 | 0 | 0 | 0 | 0 | 2 | 1 | 3 |
| L-methionine | 0 | 0 | 0 | 0 | 0 | 2 | 1 | 3 |

M495T296:(1r,4s,7r,8s)-7-[(2e,4e)-hexa-2,4-dienoyl]-3-hydroxy-8-[(2s)-3-hydroxy-2 ,4-dimethyl-5-oxo-2,5-dihydrofuran-2-yl]-5-[(2e,4e)-1-hydroxyhexa-2,4-dien-1-ylidene]-1,3-dimethylbicyclo[2.2.2]octane-2,6-dione.

The biological significance of the 30 metabolites was further investigated using the literature, Pubchem, and HMDB databases. Metabolites associated with environmental exposures, specific drugs, food, or plant metabolites were excluded due to their direct link to lifestyle habits. Following the identification of metabolites, six potential biomarkers - DLG, 7,8-dihydrobiopterin, L-carnitine, 1-methylhistidine, Coproporphyrin i, and L-methionine - were identified as pre-screening results.

#### Supplementary Table 7. Biological significance of screened metabolites

| Metabolites ID | Alternative name | Biological Significance | |
| --- | --- | --- | --- |
| L-glutamic acid dimethyl ester | Glutamic acid gamma-methyl ester | . | Belongs to the glutamic acid and its derivatives class of organic compounds, which inhibit KATP channel activity, inhibit the growth of Ciliophora and lead to abnormal cell division, which can be used in diabetes, glucose transport, phosphorylation and further metabolism studies |
| 3-nitrobenzenesulfonic acid | nitrobenzene sulfonic acid | Environmental exposures | Plays an anti-inflammatory role in the mammalian intestine. May play a role in maintaining the intestinal barrier, important in diabetes. |
| Trans-3'-hydroxycotinine | Trans-3'-hydroxycotinine | Food metabolites | Is a member of the pyrrolidinopyridine group of compounds. Present in many foods such as annual wild rice, macadamia nuts, corn salad, and radishes, and is a potential biomarker for consumption of these foods. |
| 7,8-dihydrobiopterin | Dihydrobiopterin | Markers known to be associated with kidney disease | It belongs to a group of organic compounds known as biopterins and their derivatives. In humans, dihydrobiopterin has been associated with a variety of metabolic disorders and diseases, including chronic kidney disease, colorectal cancer, folate metabolism |
| 2-hydroxyatorvastatin lactone | ortho-hydroxyatorvastatin | Drug Metabolites | It is a metabolite of atorvastatin |
| 1h-indole-1-pentanoic acid, 3-(1-naphthalenylcarbonyl)- | . | . | . |
| L-carnitine | L-Carnitine | Known markers associated with kidney disease | Carnitine is a non-essential amino acid and quaternary ammonium compound. Also classified as an alcohol, carnitine is concentrated in skeletal and cardiac muscle and other tissues that metabolise fatty acids as a source of energy, and is an essential nutrient for preterm infants and people who are unable to eat a normal diet (e.g., non-ketotic hypoglycaemia, renal dialysis patients) |
| Nicotine | Nicotine | Food Metabolites | Nicotine binds to nicotinic acetylcholine receptors on chromaffin cells in the adrenal medulla, the binding opens ion channels allowing sodium influx which leads to cellular depolarisation and activation of voltage-gated calcium channels, calcium triggers the release of adrenaline from intracellular vesicles into the bloodstream which leads to vasoconstriction, elevated blood pressure, increased heart rate, and elevated blood glucose. |
| Nonanoic acid | Pelargonic acid | Food Metabolites | Geranic acid or nonanoic acid is a fatty acid that occurs naturally in geranium oil as an ester. |
| Tabersonine | Tabersonine | Plant Metabolites | https://www.genome.jp/dbget-bin/www_bget?C09244 |
| Tebufenpyrad | Tebufenpyrad | Environmental exposures | Tebufenpyran, also known as pyran, belongs to a class of organic compounds known as phenylpropanes. Tebufenpyran is not a naturally occurring metabolite and is only found in individuals exposed to the compound or its derivatives. |
| Hypericin | Hypericin | Plant Metabolites | Hypericin is found in alcoholic beverages. Hypericin is widely distributed in the genus Hypericum and, along with hypericin, is one of the main active components of Hypericum. |
| M495T296 | . | . | . |
| Secoisolariciresinol | Secoisolariciresinol | Environmental exposures | It belongs to the dibenzylbutanediol lignans class of organic compounds, and secoisoleucine has been detected in several different foods, such as cocoa beans, Swiss chard, parsley, and kohlrabi, and is a potential biomarker for consumption of these foods. |
| Bisphenol e | . | Environmental exposures | . |
| Rhamnetin | Isorhamnetin | Plant Metabolites | Isorhamnetin is a methylated metabolite of quercetin. Quercetin is an important dietary flavonoid with in vitro antioxidant activity. Isorhamnetin is antioxidant and attenuates endothelial cell damage. |
| Stachydrine | Proline betaine | Food Metabolites | Belonging to the class of organic compounds called proline and its derivatives, it is present in all organisms from bacteria to humans, with the highest average content in chilli peppers and detected in several different foods. |
| 1-methylhistidine | 1-Methylhistidine | Muscle breakdown | In addition to muscle protein degradation, 1-methylhistidine levels are moderately affected by intestinal protein degradation and meat intake. 1-methylhistidine has been found to be associated with a variety of diseases, such as Alzheimer's disease, pre-eclampsia, obesity, and kidney disease. |
| Blood group a trisaccharide | Alpha-Trisaccharide | Food Metabolites | This is a carbohydrate derivative containing a hexose fraction in which the oxygen atom is replaced by an n-acyl group. alpha-trisaccharides have been detected in several different foods and are potential biomarkers for consumption of these foods. |
| Hydroxybupropion | . | Food Metabolites | It has been detected, but not quantified, in several different foods, such as bright berries, kale, cactus, and hazelnuts, and is a potential biomarker for the consumption of these foods. |
| Spinosine | . | Food Metabolites | Spinosin A belongs to a class of organic compounds known as flavonoid c-glycosides. Flavonoid C-glycosides are compounds that contain a carbohydrate portion that is linked to the 2-phenylchromen-4-one flavonoid backbone C-glycoside. |
| D-mannitol 1-phosphate | Mannitol 1-phosphate | Gut Flora Metabolites | Mannitol 1-phosphate is a sugar alcohol. Mannitol 1-phosphate is also produced in many organisms (various flora) that have a range of biological interactions with humans. |
| Isoeugenyl acetate | Isoeugenyl acetate | Food metabolites | Isoeugenol acetate, also known as acetylated isoeugenol, belongs to the phenolic ester group of organic compounds. These are aromatic compounds containing benzene rings substituted with hydroxyl and ester groups. |
| 3-hydroxykynurenine | Hydroxykynurenine | Involved in tryptophan metabolism | Hydroxykynurenine is a very strong alkaline compound involved in tryptophan metabolism. |
| Coproporphyrin i | Coproporphyrin I | Known endogenous biomarkers associated with kidney disease | Coproporphyrin I is a porphyrin metabolite produced during heme synthesis. Elevated faecal porphyrin levels can indicate congenital erythropoietic porphyria or ferroblastic anaemia, liver damage |
| L-methionine | Methionine | Known endogenous biomarkers associated with kidney disease | Also known as L-methionine, it is an alpha-amino acid and essential amino acid that must be obtained from the diet. Acute doses of methionine result in an acute increase in plasma homocysteine, which can be an indicator of susceptibility to cardiovascular disease. |
| Creatine | . | Known endogenous biomarkers associated with kidney disease | Creatine is a naturally occurring non-protein compound that belongs to the class of organic compounds known as alpha-amino acids and their derivatives. Creatine is found in all vertebrates and facilitates the cycling of adenosine triphosphate (ATP), and its primary metabolic role is to bind to the phosphate group via creatine kinase to produce phosphocreatine, which is used to regenerate ATP. |
| 3-hydroxybenzaldehyde | . | Food metabolites | It belongs to the organic compound class of hydroxybenzaldehyde. Highest average concentrations were found in vinegar and oats. It is a potential biomarker for food consumption. |

### Section 1.7. Dual Screening of Metabolic Markers by Two-sample Mendelian Randomization.

This subsection delves into the causal relationship between metabolite concentration levels and the risk of DKD, focusing on genetic variation. Through Mendelian randomization analysis of SNPs identified from 5 GWAS studies, the study evaluated the causal impact using various estimation methods.

#### Supplementary Table 8. Information on mendelian randomization genetic data

| Name | GWAS ID | Sample size | Time | Source |
| --- | --- | --- | --- | --- |
| L-glutamic acid, dimethyl ester | GCST90200412 | 8,287 | 2023/1/12 | European |
| 7,8-dihydrobiopterin | GCST90265032 | 3,644 | 2023/6/5 | European |
| L-carnitine | GCST90199621 | 8,192 | 2023/1/12 | European |
| 1-methylhistidine | GCST90199691 | 8,226 | 2023/1/12 | European |
| L-methionine | GCST90199625 | 8,243 | 2023/1/12 | European |
| Diabetic Nephropathy | GCST90018832 | 585,264 | 2021/9/30 | European (Finland, U.K.)&East Asian (Japan) |

The analysis results in Table 1-8 reveal 333 significantly related SNPs as independent variables for DLG. According to the Mendelian randomization hypothesis and utilizing the IVW method, these SNPs are identified as causative factors associated with DKD outcome, with an odds ratio of 1.18 (1.10-1.26) and a p-value of 1.73E-06, indicating a causal relationship. The MR PRESSO method further confirms a significant causal relationship (MR P = 8.1E-10), with a Causal Estimate of 0.166, consistent with the IVW direction. The Global Test P > 0.05 suggests no horizontal pleiotropy. Heterogeneity is evaluated through the Q test (MR Egger and IVW) to ensure the robustness of the results, with a Q p-value > 0.05 indicating no heterogeneity. The Steiger direction test supports the direction of causality as true, indicating no reverse causality.

7,8-dihydrobiopterin (Dihydrobiopterin) is associated with 81 SNPs as independent variables. Following the Mendelian randomization hypothesis and utilizing the IVW method, it was identified as a causative factor in DKD outcome (OR = 1.27 (1.15-1.41), P = 5.60E-06), demonstrating a causal relationship. The MR PRESSO method further validated this causal association (MR P = 2.48E-07). A Global Test P value > 0.05 indicates no horizontal pleiotropy, while the Q pval > 0.05 suggests no heterogeneity. The Steiger direction test supports the correct causal direction, ruling out reverse causality. Additionally, 1-methylhistidine shows a positive causal link with DKD, with no observed heterogeneity or horizontal pleiotropy. L-carnitine displays significant IVW results with heterogeneity but no horizontal pleiotropy, while L-methionine shows significant IVW results without heterogeneity but potential horizontal pleiotropy, indicating less robust outcomes. In conclusion, the study highlights DLG and dihydrobiopterin as the most reliable factors.

#### Supplementary Table 9. Genetic associations of metabolic markers with risk of diabetic kidney disease

| Metabolites | | L-glutamic acid, dimethyl ester | 7,8-dihydrobiopterin | L-carnitine | 1-methylhistidine | L-methionine |
| --- | --- | --- | --- | --- | --- | --- |
| Outcome ID | | GCST90018832 | | | | |
| Sample size | | 585,264 | | | | |
| Number of case and control | | case: 1,252 control: 584,012 | | | | |
| GWAS ID | | GCST90200412 | GCST90265032 | GCST90199621 | GCST90199691 | GCST90199625 |
| No. of SNP（*P* < 1e-5） | | 333 | 81 | 1359 | 754 | 160 |
| IVW (fixed effects) | OR (95% CI) | 1.18 (1.10-1.26) | 1.27 (1.15-1.41) | 0.94 (0.91-0.96) | 1.26 (1.22-1.30) | 0.87 (0.81-0.94) |
|  | P value | 1.73E-06 | 5.60E-06 | 2.40E-06 | 2.29E-49 | 3.54E-04 |
|  | Q pval | 1.000 | 0.994 | 4.24E-14 | 1.000 | 0.462 |
| Weighted median | OR (95% CI) | 1.23 (1.12-1.35) | 1.44 (1.25-1.66) | 0.99 (0.95-1.03) | 1.28 (1.23-1.34) | 0.99 (0.89-1.11) |
|  | P value | 1.66E-05 | 2.92E-07 | 4.98E-01 | 5.77E-33 | 9.02E-01 |
| Weighted mode | OR (95% CI) | 1.30 (0.99-1.71) | 1.66 (1.15-2.38) | 1.08 (0.97-1.20) | 1.29 (1.14-1.46) | 1.02 (0.93-1.12) |
|  | P value | 6.19E-02 | 8.07E-03 | 1.60E-01 | 7.29E-05 | 7.04E-01 |
| MR Egger | OR (95% CI) | 0.52 (0.42-0.63) | 1.12 (0.85-1.46) | 0.95 (0.90-1.00) | 1.18 (1.07-1.30) | 0.99 (0.91-1.09) |
|  | P value | 2.84E-10 | 4.26E-01 | 6.90E-02 | 1.03E-03 | 8.62E-01 |
|  | Q pval | 1.000 | 0.994 | 4.6E-14 | 1.000 | 0.148 |
| Simple mode | OR (95% CI) | 1.31 (0.98-1.76) | 1.61 (1.12-2.32) | 1.08 (0.97-1.20) | 1.29 (1.13-1.47) | 1.05 (0.72-1.52) |
|  | P value | 6.71E-02 | 1.22E-02 | 1.60E-01 | 2.01E-04 | 8.16E-01 |
| MR PRESSO | Global Test RSSobs | 190.85 | 53.36 | NA* | 375.60 | 189.53 |
|  | Causal Estimate | 0.166 | 0.241 | NA* | 0.231 | -0.134 |
|  | Global Test P value | 1 | 0.993 | NA* | 1 | 0.124 |
|  | MR P value | 8.1E-10 | 2.48E-07 | NA* | 4.56E-77 | 4.69E-04 |
| MR-Egger regression | Egger intercept | 9.14E-02 | 1.95E-02 | -2.49E-03 | 9.65E-03 | -2.92E-02 |
|  | P value | 2.04E-16 | 0.296 | 0.545 | 0.148 | 2.92E-05 |
| Steiger Test | | TRUE | TRUE | TRUE | TRUE | TRUE |

### Section 1.8. Evaluating the Prediction Performance of Metabolic Markers based on the Random Forest Algorithm

The classification effects of DLG, L-Carnitine, and L-Methionine in public databases (NC, DM, DKD) were further assessed to determine their accuracy, sensitivity, and specificity. Various metrics such as accuracy, recall, and AUC were utilized to evaluate the predictive capabilities of these substances. sTable 10 displays the accuracy rates of DLG, L-Carnitine, and L-Methionine as 0.617, 0.580, and 0.553, respectively. DLG exhibited higher AUC values for the NC group, DM group, and DKD group at 0.807, 0.804, and 0.645, respectively, indicating superior predictive performance compared to L-Carnitine and L-Methionine. By employing Mendelian randomization and conducting secondary screening of the database, DLG was identified as the most reliable biomarker for DKD.

#### Supplementary **Table 10. Evaluating the prediction performance of metabolic markers based on the random forest algorithm**

| Evaluation parameters | | L-glutamic acid dimethyl ester | L-Carnitine | L-Methionine |
| --- | --- | --- | --- | --- |
| Accuracy |  | 0.617 | 0.580 | 0.553 |
|  | 95% CI | (0.464, 0.755) | (0.432, 0.718) | (0.401, 0.698) |
| AUC | NC | 0.807 | 0.545 | 0.814 |
|  | DM | 0.804 | 0.636 | 0.567 |
|  | DKD | 0.645 | 0.631 | 0.615 |
| Sensitivity | NC | 0.000 | 0.222 | 0.286 |
|  | DM | 0.667 | 0.583 | 0.111 |
|  | DKD | 0.742 | 0.690 | 0.742 |
| Specificity | NC | 0.975 | 0.976 | 0.975 |
|  | DM | 0.763 | 0.711 | 0.789 |
|  | DKD | 0.500 | 0.571 | 0.250 |
| Precision | NC | 0.000 | 0.667 | 0.667 |
|  | DM | 0.400 | 0.389 | 0.111 |
|  | DKD | 0.742 | 0.670 | 0.657 |
| Recall | NC | 0.000 | 0.222 | 0.286 |
|  | DM | 0.667 | 0.583 | 0.111 |
|  | DKD | 0.742 | 0.690 | 0.742 |

### Section 1.9. **Number and Percentage of Differential Lipid Subclasses**

#### Supplementary Table 11. Number and percentage of differential lipid subclasse

|  | T2DM vs HC | | HRDKD vs T2DM | | DKD vs HRDKD | |
| --- | --- | --- | --- | --- | --- | --- |
| No. of DEGs | 292 | | 116 | | 28 | |
| Class | n | % | n | % | n | % |
| PE | 52 | 17.81 | 25 | 21.55 | 0 | 0.00 |
| PC | 54 | 18.49 | 28 | 24.14 | 0 | 0.00 |
| TG | 42 | 14.38 | 12 | 10.34 | 1 | 3.57 |
| PS | 3 | 1.03 | 2 | 1.72 | 0 | 0.00 |
| PG | 29 | 9.93 | 0 | 0.00 | 2 | 7.14 |
| DG | 18 | 6.16 | 6 | 5.17 | 2 | 7.14 |
| CL | 6 | 2.05 | 4 | 3.45 | 0 | 0.00 |
| LPC | 1 | 0.34 | 0 | 0.00 | 0 | 0.00 |
| PI | 6 | 2.05 | 2 | 1.72 | 5 | 17.86 |
| Hex1Cer | 1 | 0.34 | 2 | 1.72 | 0 | 0.00 |
| LPI | 1 | 0.34 | 0 | 0.00 | 0 | 0.00 |
| SM | 40 | 13.70 | 12 | 10.34 | 0 | 0.00 |
| Cer | 14 | 4.79 | 4 | 3.45 | 0 | 0.00 |
| SPH | 7 | 2.40 | 0 | 0.00 | 0 | 0.00 |
| ChE | 1 | 0.34 | 1 | 0.86 | 0 | 0.00 |
| LSM | 1 | 0.34 | 1 | 0.86 | 0 | 0.00 |
| phSM | 1 | 0.34 | 1 | 0.86 | 0 | 0.00 |
| LPE | 4 | 1.37 | 0 | 0.00 | 0 | 0.00 |
| PIP | 0 | 0.00 | 0 | 0.00 | 7 | 25.00 |
| PIP2 | 0 | 0.00 | 0 | 0.00 | 7 | 25.00 |
| PIP3 | 0 | 0.00 | 0 | 0.00 | 4 | 14.29 |
| GM3 | 2 | 0.68 | 0 | 0.00 | 0 | 0.00 |
| Hex2Cer | 1 | 0.34 | 0 | 0.00 | 0 | 0.00 |
| LPG | 1 | 0.34 | 0 | 0.00 | 0 | 0.00 |
| FA | 1 | 0.34 | 0 | 0.00 | 0 | 0.00 |
| WE | 1 | 0.34 | 0 | 0.00 | 0 | 0.00 |
| ST | 3 | 1.03 | 0 | 0.00 | 0 | 0.00 |
| ZyE | 2 | 0.68 | 0 | 0.00 | 0 | 0.00 |

LPC: lysophosphatidylcholine; PAF: platelet-activating factor; PC: phosphatidylcholine; MePC: methylphosphatidylcholine; LPE: lysophosphatidylethanolamine; LdMePE: lysophosphatidylethanolamine; PE: phosphatidylethanolamine; LPS: lysophosphatidylserine; PS: phosphatidylserine; LPG: lysophosphatidylglycerol; PG: phosphatidyl glycerol; LPI: lysophosphatidylinositol; PI, PIP, PIP2, PIP3: phosphatidylinositol; LPA: lysophosphatidic acid; PA: phosphatidic acid; CL: cardiolipin; SM: sphingomyelin; LSM: lysophosphatidylmuscle myosin; So: sphingosine; SoP: sphingosine phosphate; SoG1: glucosylsphingosine; ST: sulphide; Cer: ceramide; CerP: phosphoceramide; GM3, GM2, GM1, GD1a, GD1b, GD2, GD3, GT1a, GT1b, GT1c, GT2, GT3, GQ1c, GQ1b: gangliosides; MG: monoglyceride; DG: diglyceride; TG: triglyceride; ChE: cholesteryl ester; ZyE: zeylenol; Co: coenzyme; WE : waxy lipid; FA: fatty acid.

### Section 1.10. Comprehensive Score of Lipidomics Biomarkers.

A multidimensional comparison and comprehensive scoring was conducted on the lipid metabolites, resulting in the identification of top-ranking lipids. These lipids include TG(18:3e_18:4_19:1), TG(20:5_14:1_22:6), PE(16:0_18:1), DG(8:0_11:2), PC(33:3), PC(35:3e), PE(18:0_18:1), PE(18:1_18:1), PE(39:5e), PG(47:3), WE(4:0_18:3), PE(18:0_20:3), and SM(t18:0_23:6), totaling 12 lipid markers identified through pre-screening.[润色](https://www.citexs.com/Editing)

#### Supplementary Table 12. Comprehensive score of lipidomics biomarkers.

| Lipidomics ID | Decision tree1 | Decision tree2 | Random forest | Feature engineering | Correlation coefficient>0.7 | Number of groups with differences | Total score |
| --- | --- | --- | --- | --- | --- | --- | --- |
| TG(18:3e_18:4_19:1) | 0 | 0 | 1 | 1 | 2 | 1 | 5 |
| TG(20:5_14:1_22:6) | 0 | 0 | 1 | 1 | 0 | 1 | 3 |
| PE(16:0_18:1) | 0 | 0 | 0 | 1 | 2 | 0 | 3 |
| DG(8:0_11:2) | 0 | 0 | 1 | 1 | 0 | 0 | 2 |
| PC(33:3) | 0 | 0 | 1 | 1 | 0 | 0 | 2 |
| PC(35:3e) | 0 | 0 | 1 | 1 | 0 | 0 | 2 |
| PE(18:0_18:1) | 0 | 0 | 1 | 1 | 0 | 0 | 2 |
| PE(18:1_18:1) | 0 | 0 | 1 | 1 | 0 | 0 | 2 |
| PE(39:5e) | 0 | 0 | 1 | 1 | 0 | 0 | 2 |
| PG(47:3) | 0 | 1 | 0 | 1 | 0 | 0 | 1 |
| WE(4:0_18:3) | 0 | 1 | 0 | 1 | 0 | 0 | 1 |
| PE(18:0_20:3) | 1 | 0 | 0 | 1 | 0 | 0 | 1 |
| SM(t18:0_23:6) | 1 | 0 | 0 | 1 | 0 | 0 | 1 |

### Section 1.11. Secondary Screening for Lipid Markers Performed by Two-sample Mendelian Randomization.

This study examines the relationship between lipid metabolite levels, genetic variants, and the risk of diabetic kidney disease (DKD) through Mendelian randomization (MR) analysis. Genetic information from SNPs associated with lipid levels was obtained from genome-wide association studies (GWAS). Significant SNPs were identified based on a threshold of P < 1e-5. Causal effects were evaluated using IVW, Weighted median, Weighted mode, MR Egger, and Simple mode methods after excluding SNPs associated with the outcome.

#### Supplementary Table 13. Details of mendelian randomized genetic agent datasets

| Name | GWAS ID | Sample size | Time | Source |
| --- | --- | --- | --- | --- |
| TG(18:3e_18:4_19:1) | GCST90024545 | 4492 | 2022/6/6 | European (Australia) |
| TG(20:5_14:1_22:6) | GCST90024572 | 4492 | 2022/6/6 | European (Australia) |
| PE(16:0_18:1) | GCST90024351 | 4492 | 2022/6/6 | European (Australia) |
| PG(47:3) | GCST90024600 | 4492 | 2022/6/6 | European (Australia) |
| DG(8:0_11:2) | GCST90024706 | 6057 | 2022/6/6 | European (U.S., Australia) |
| PC(33:3) | GCST90060261 | 13814 | 2021/9/10 | European (U.K.) |
| PC(35:3e) | GCST90060319 | 13814 | 2021/9/10 | European (U.K.) |
| PE(18:0_18:1) | GCST90024365 | 4492 | 2022/6/6 | European (Australia) |
| PE(18:0_20:3) | GCST90024367 | 4492 | 2022/6/6 | European (Australia) |
| PE(18:1_18:1) | GCST90024374 | 4492 | 2022/6/6 | European (Australia) |
| PE(39:5e) | GCST90060346 | 13814 | 2021/9/10 | European (U.K.) |
| SM(t18:0_23:6) | GCST90200123 | 8277 | 2023/1/12 | European (Canada) |

PC(35:3e) is associated with 64 significantly related SNPs as independent variables. Following the Mendelian randomization (MR) hypothesis and using the IVW method, it has been identified as a causative factor significantly linked to the outcome of DKD (IVW OR = 0.80 (0.73-0.87), P = 2.68E-07), indicating a causal relationship. The MR PRESSO method also confirms this significant causal relationship (MR P value=1.68E-7), with a Causal Estimate of -0.224. Both IVW and MR Egger methods show consistent directions, with a Global Test P value > 0.05 suggesting no horizontal pleiotropy. Heterogeneity is assessed through Q test (MR Egger and IVW), with Q Test's Q pval > 0.05 indicating no heterogeneity. The Steiger direction test supports the causal direction as true, ruling out reverse causality. Overall, the MR findings for PC(35:3e) are robust, with P < 0.05 across all analysis methods, consistent beta direction, and no evidence of heterogeneity or horizontal pleiotropy.

PC(33:3) and PE(39:5e) were found to have 35 and 41 significantly associated SNPs, respectively, as independent variables. According to the MR hypothesis using the IVW method, PC(33:3) was identified as a causative factor associated with DKD outcome (IVW OR = 0.56 (0.43-0.74), P = 2.36 E-05), indicating a causal relationship. The MR PRESSO method also confirmed the significant causal relationship (MR P = 0.008), with Global Test P value > 0.05, suggesting no horizontal pleiotropy, and Q test Q pval > 0.05, indicating no heterogeneity. The Steiger direction test supported the causal direction as true, ruling out reverse causality. Similarly, PE(39:5e) was determined to be related to DKD outcome with 41 significantly associated SNPs. The IVW method showed a causative factor (IVW OR = 1.32 (1.15-1.51), P = 7.46E-05), and the MR PRESSO method confirmed the significant causal relationship (MR P = 1.01E -10). Global Test P value > 0.05, Q pval of Q test > 0.05, and Steiger direction test all supported the absence of horizontal pleiotropy, heterogeneity, and reverse causality, respectively.

The remaining lipids in the Mendelian randomization test did not meet the screening criteria due to heterogeneity and horizontal pleiotropy. Specifically, results of TG(18:3e_18:4_19:1), TG(20:5_14:1_22:6), and SM(t18:0_23:6) were not significant. Additionally, PE(16:0_18:1), PG(47:3), DG(8:0_11:2), PE(18:0_18:1), and PE(18:1_18:1) showed horizontal pleiotropy. The MR result of PE(18:0_20:3) was significant (IVW OR = 0.93 (0.91-0.94), P = 1.46E-22), but may have been affected by horizontal pleiotropy. SM(t18:0_23:6) results were significant (IVW OR = 0.91 (0.87-0.95), P = 1.10E-05), yet heterogeneity was observed (Q pval of MR Egger and IVW are both <0.05). Ultimately, the most reliable results were found for PC(35:3e), PC(33:3), and PE(39:5e).

#### Supplementary **Table 14a. Pre-screening for genetic associations of lipid metabolites with risk of DKD**

| Lipid Metabolites ID | | TG(18:3e_18:4_19:1) | TG(20:5_14:1_22:6) | PE(16:0_18:1) | PG(47:3) | DG(8:0_11:2) | PC(33:3) |
| --- | --- | --- | --- | --- | --- | --- | --- |
| DKD Outcome | GWAS ID | GSTC90018832 | | | | | |
|  | Sample size | 585,264 | | | | | |
|  | Number of case and control | Case: 1,252; Control: 584,012 | | | | | |
| GWAS ID | | GCST90024545 | GCST90024572 | GCST90024351 | GCST90024600 | GCST90024706 | GCST90060261 |
| No. of SNP (*P* < 1e-5) | | 1460 | 303 | 437 | 649 | 129 | 35 |
| IVW (fixed effects) | OR (95% CI) | 1.09 (1.06-1.12) | 1.44 (1.34-1.55) | 0.90 (0.88-0.92) | 1.02 (1.00-1.05) | 0.99 (0.94-1.05) | 0.76 (0.63-0.92) |
|  | P value | 2.01E-11 | 2.81E-23 | 9.73E-20 | 0.075 | 0.809 | 0.005 |
|  | Q pval | 3.93E-94 | 1.38E-13 | 0.969 | 1.000 | 0.977 | 0.498 |
| Weighted median | OR (95% CI) | 1.13 (1.10-1.17) | 1.65 (1.51-1.81) | 0.96 (0.92-1.00) | 1.07 (1.02-1.11) | 0.95 (0.86-1.06) | 0.56 (0.43-0.74) |
|  | P value | 1.66E-05 | 4.57E-28 | 8.09E-02 | 0.004 | 0.352 | 2.36E-05 |
| Weighted mode | OR (95% CI) | 1.48 (1.24-1.76) | 2.84 (1.79-4.51) | 0.99 (0.95-1.04) | 1.10 (1.02-1.19) | 0.96 (0.87-1.05) | 0.55 (0.37-0.82) |
|  | P value | 1.85E-05 | 1.25E-05 | 0.792 | 0.012 | 0.399 | 5.92E-03 |
| MR Egger | OR (95% CI) | 1.45 (1.36-1.56) | 0.52 (0.43-0.63) | 1.02 (0.98-1.06) | 0.92 (0.87-0.98) | 1.01 (0.93-1.10) | 0.27 (0.10-0.76) |
|  | P value | 2.32E-25 | 1.93E-10 | 0.451 | 0.006 | 0.779 | 0.019 |
|  | Q pval | 1.84E-79 | 2.94E-03 | 1.000 | 1.000 | 0.975 | 0.646 |
| Simple mode | OR (95% CI) | 0.72 (0.55-0.93) | 2.84 (1.87-4.31) | 0.75 (0.66-0.86) | 1.10 (0.97-1.25) | 1.27 (1.00-1.61) | 0.55 (0.35-0.86) |
|  | P value | 1.33E-02 | 1.47E-06 | 4.02E-05 | 0.127 | 0.055 | 1.22E-02 |
| MR PRESSO | Global Test RSSobs | NA | 104.795 | 386.936 | 321.101 | 100.067 | 35.018 |
|  | Causal Estimate | NA | 0.366 | -0.104 | 0.021 | -0.007 | -0.275 |
|  | Global Test Pvalue | NA | 0.693 | 0.977 | 1.000 | 0.980 | 0.528 |
| MR-Egger regression | Egger intercept | -0.049 | 0.121 | -0.032 | 0.018 | -0.005 | 0.086 |
|  | P value | 7.03E-18 | 3.06E-23 | 2.33E-11 | 1.69E-04 | 0.544 | 0.055 |
| Steiger Test | | TRUE | TRUE | TRUE | TRUE | TRUE | TRUE |

#### Supplementary **Table 14b. Pre-screening for genetic associations of lipid metabolites with risk of DKD**

| Lipid Metabolites ID | | PC(35:3e) | PE(18:0_18:1) | PE(18:0_20:3) | PE(18:1_18:1) | PE(39:5e) | SM(t18:0_23:6) |
| --- | --- | --- | --- | --- | --- | --- | --- |
| DKD Outcome | GWAS ID | GSTC90018832 | | | | | |
|  | Sample size | 585,264 | | | | | |
|  | Number of case and control | Case: 1,252; Control: 584,012 | | | | | |
| GWAS ID | | GCST90060319 | GCST90024365 | GCST90024367 | GCST90024374 | GCST90060346 | GCST90200123 |
| No. of SNP (*P* < 1e-5) | | 64 | 432 | 661 | 425 | 41 | 424 |
| IVW (fixed effects) | OR (95% CI) | 0.80 (0.73-0.87) | 0.88 (0.86-0.91) | 0.93 (0.91-0.94) | 0.87 (0.85-0.89) | 1.31 (1.18-1.45) | 0.91 (0.87-0.95) |
|  | P value | 2.68E-07 | 5.98E-22 | 1.46E-22 | 3.52E-34 | 3.35E-07 | 1.10E-05 |
|  | Q pval | 0.916 | 0.940 | 0.787 | 0.998 | 1.000 | 7.66E-05 |
| Weighted median | OR (95% CI) | 0.79 (0.70-0.89) | 0.85 (0.82-0.88) | 0.89 (0.87-0.91) | 0.85 (0.82-0.88) | 1.32 (1.15-1.51) | 0.90 (0.84-0.97) |
|  | P value | 7.34E-05 | 1.18E-17 | 1.16E-19 | 9.49E-22 | 7.46E-05 | 0.003 |
| Weighted mode | OR (95% CI) | 0.78 (0.70-0.88) | 0.76 (0.66-0.87) | 0.89 (0.86-0.92) | 0.85 (0.80-0.90) | 1.32 (1.12-1.55) | 0.56 (0.46-0.70) |
|  | P value | 4.98E-05 | 1.14E-04 | 7.38E-12 | 4.42E-08 | 0.002 | 1.90E-07 |
| MR Egger | OR (95% CI) | 0.73 (0.60-0.88) | 1.00 (0.93-1.07) | 0.89 (0.86-0.92) | 0.96 (0.91-1.02) | 1.53 (1.13-2.09) | 0.86 (0.77-0.96) |
|  | P value | 0.002 | 0.899 | 7.49E-14 | 0.235 | 0.009 | 0.008 |
|  | Q pval | 0.920 | 0.975 | 0.849 | 1.000 | 1.000 | 7.67887E-05 |
| Simple mode | OR (95% CI) | 0.79 (0.68-0.92) | 0.76 (0.66-0.87) | 0.88 (0.83-0.93) | 0.84 (0.77-0.92) | 1.32 (1.09-1.60) | 0.57 (0.40-0.81) |
|  | P value | 3.49E-03 | 8.24E-05 | 3.75E-06 | 7.71E-05 | 0.007 | 0.002 |
| MR PRESSO | Global Test RSSobs | 48.654 | 388.315 | 633.014 | 347.269 | 14.095 | 544.651 |
|  | Causal Estimate | -0.224 | -0.123 | -0.077 | -0.142961975 | 0.268 | -0.100 |
|  | Global Test Pvalue | 0.941 | 0.933 | 0.804 | 0.996 | 1.000 | <0.001 |
| MR-Egger regression | Egger intercept | 0.014 | -0.025 | 0.013 | -0.025 | -0.025 | 0.008 |
|  | P value | 0.300 | 6.51E-04 | 0.002 | 1.84E-04 | 0.284 | 0.344 |
| Steiger Test | | TRUE | TRUE | TRUE | TRUE | TRUE | TRUE |

### Section 1.12. Baseline demographic characteristics and biochemical indicators for patients in the cross-sectional study

Based on the enrollment criteria and risk factors, the patients were categorized into four groups: a healthy control group (30 participants), a diabetes group (361 participants), a high-risk group for diabetic nephropathy (555 participants), and a diabetic nephropathy group (554 participants). The median slopes of decline in eGFR since 2022 were 2.75, 3.93, and 4.45 ml/min/1.73 m^2^/year for the respective groups, except for the healthy control group. Similarly, the median slopes of decline in eGFR since 2014 were 1.14, 0.97, and 2.40 ml/min/1.73 m^2^/year for the same groups. Factors such as age, smoking and alcohol consumption, past medical history (including hypertension, coronary artery disease, and diabetic retinopathy), duration of type 2 diabetes mellitus, BMI, waist-to-hip ratio, systolic and diastolic blood pressure showed significant increases with the DKD subgroup (P < 0.001). Moreover, in routine blood and biochemical indicators, the levels of monocytes, HbA1c, urea nitrogen, blood uric acid, and TG also increased with the DKD group (P < 0.001). In terms of 24-hour urine biochemical indicators, urinary calcium levels decreased quantitatively with the increase in DKD group, while indicators related to renal injury such as urinary protein levels, N.Acetylaminoglucosidase, urinary transferrin levels, urinary β2-microglobulin, and immunoglobulin G all showed quantitative increases with the DKD grouping (P < 0.001).

#### Supplementary Table 15. Baseline demographic characteristics and biochemical indicators for patients in the cross-sectional study

| Characteristics | HC Group | T2DM Group | HR-DKD Group | DKD Group | *P* value |
| --- | --- | --- | --- | --- | --- |
| All, N(%) | 30 (2.00) | 361 (24.07) | 555 (37.00) | 554 (36.93) |  |
| Ethnicity (Han), N(%) | 30 (100.00) | 358 (99.17) | 545 (98.20) | 538 (97.11) | 0.155 |
| Marital status (married), N(%) | 30 (100.00) | 352 (97.51) | 544 (98.02) | 531 (95.85) | 0.036 |
| Sex (male), N(%) | 13 (43.33) | 201 (55.68) | 323 (58.20) | 353 (63.72) | 0.020 |
| Age, years | 50.00 (38.00, 55.00) | 54.00 (41.00, 62.00) | 59.00 (52.00, 66.00) | 60.00 (52.00, 66.00) | <0.001 |
| Smoking, N(%) | 0 (0.00) | 31 (8.59) | 177 (31.89) | 159 (28.70) | <0.001 |
| Drinking, N(%) | 0 (0.00) | 5 (1.44) | 146 (25.70) | 124 (22.38) | <0.001 |
| Hypertension, N(%) | 0 (0.00) | 6 (1.66) | 302 (54.41) | 384 (69.31) | <0.001 |
| Hyperlipidemia, N(%) | 0 (0.00) | 217 (60.11) | 505 (90.99) | 485 (87.55) | <0.001 |
| Coronary heart disease, N(%) | 0 (0.00) | 43 (11.91) | 236 (42.52) | 246 (44.40) | <0.001 |
| Fatty liver disease, N(%) | 0 (0.00) | 50 (13.85) | 251 (45.23) | 218 (39.35) | <0.001 |
| Diabetic retinopathy, N(%) | 0 (0.00) | 7 (1.94) | 209 (37.66) | 290 (52.35) | <0.001 |
| Obesity, N(%) | 0 (0.00) | 61 (16.90) | 208 (37.48) | 198 (35.74) | <0.001 |
| Duration of T2DM, years | 0 (0.00) | 2.00 (1.00, 6.00) | 10.00 (5.00, 16.00) | 11.00 (5.00, 19.00) | <0.001 |
| BMI, kg/m^2^ | 22.82 (2.26) | 25.58 (3.35) | 25.74 (4.00) | 26.87 (4.07) | <0.001 |
| WHratio | 0.88 (0.08) | 0.92 (0.06) | 0.94 (0.06) | 0.95 (0.06) | <0.001 |
| Systolic BP, mmHg | 119.13 (3.91) | 125.89 (12.13) | 132.83 (14.91) | 138.44 (18.68) | <0.001 |
| Diastolic BP, mmHg | 75.6 (3.90) | 78.26 (7.32) | 79.01 (10.18) | 81.58 (11.17) | <0.001 |
| Medications |  |  |  |  |  |
| Aspirin | 0 (0.00) | 21 (5.82) | 169 (30.45) | 146 (26.35) | <0.001 |
| β-blocker | 0 (0.00) | 7 (1.94) | 112 (20.18) | 140 (25.27) | <0.001 |
| Finerenone | 0 (0.00) | 1 (0.28) | 2 (0.36) | 55 (9.93) | <0.001 |
| Lipid-lowering medication | 0 (0.00) | 120 (33.24) | 337 (60.72) | 349 (63.00) | <0.001 |
| Anti-hypertensive drugs | 0 (0.00) | 7 (1.94) | 225 (40.54) | 330 (59.57) | <0.001 |
| SGLT-2 inhibitors | 0 (0.00) | 158 (43.77) | 277 (49.91) | 271 (48.92) | <0.001 |
| GLP-1 agonists | 0 (0.00) | 60 (16.62) | 116 (20.90) | 99 (17.87) | 0.007 |
| Metformin | 0 (0.00) | 211 (58.45) | 334 (60.18) | 243 (43.86) | <0.001 |
| Insulin | 0 (0.00) | 107 (29.64) | 279 (50.27) | 316 (57.04) | <0.001 |
| Blood biochemical indicators |  |  |  |  |  |
| PLT, *10^9/L | 239.93 (76.50) | 250.50 (65.03) | 239.40 (59.68) | 240.52 (63.97) | 0.125 |
| NEUT, *10^9/L | 4.08 (1.93) | 4.14 (1.45) | 4.21 (1.41) | 4.81 (1.54) | <0.001 |
| LYM, *10^9/L | 1.75 (0.68) | 2.13 (0.69) | 1.99 (0.66) | 1.82 (0.75) | <0.001 |
| MONO, *10^9/L | 0.33 (0.13) | 0.45 (0.13) | 0.46 (0.12) | 0.48 (0.13) | <0.001 |
| FBG, mmol/L | 5.65 (2.29) | 8.42 (3.08) | 8.40 (2.87) | 8.20 (3.01) | <0.001 |
| HbA1c, % | 5.84 (0.96) | 7.65 (2.55) | 7.87 (1.70) | 7.87 (1.76) | <0.001 |
| TSH, mIU/L | 1.43 (1.37, 1.65) | 2.29 (1.36, 2.68) | 1.86 (1.29, 2.68) | 2.11 (1.36, 2.94) | <0.001 |
| ALT, U/L | 23.00 (19.50, 28.30) | 21.90 (15.50, 31.40) | 20.90 (15.30,32.10) | 19.20 (13.70, 27.20) | <0.001 |
| AST, U/L | 18.65 (16.80, 26.30) | 21.10 (17.60, 26.20) | 21.40 (17.30, 27.50) | 20.60 (16.30, 25.30) | 0.006 |
| GGT, U/L | 24.90 (23.30, 32.50) | 24.90 (18.10, 40.00) | 24.70 (18.00, 37.50) | 24.60 (18.10, 37.00) | 0.712 |
| TBIL, μmol/L | 14.36 (9.52, 22.50) | 13.91 (11.05, 17.74) | 14.04 (10.96, 17.46) | 12.07 (9.10, 15.91) | <0.001 |
| ALB, g/L | 44.61 (2.60) | 45.42 (3.38) | 44.73 (5.19) | 41.95 (7.36) | <0.001 |
| BUN, mmol/L | 5.69 (2.01) | 5.57 (2.37) | 6.00 (1.75) | 8.91 (5.72) | <0.001 |
| UA, μmol/L | 278.30 (263.60, 305.50) | 310.00 (257.50, 366.00) | 319.30 (266.00, 382.60) | 355.05 (300.90, 435.00) | <0.001 |
| Baseline eGFR, ml/min/1.73m^2^ | 117.34 (8.23) | 105.14 (15.64) | 98.95 (13.86) | 74.70 (33.12) | <0.001 |
| eGFR decline slope 2014 | 0 (0.00) | 1.14 (0, 3.03) | 0.97 (-0.27, 2.92) | 2.40 (0.18, 7.73) | <0.001 |
| eGFR decline slope 2022 | 0 (0.00) | 2.75 (0.63, 6.09) | 3.93 (0.18, 8.60) | 4.45 (-0.35, 10.64) | <0.001 |
| Baseline ACR, mg/g | 2.65 (0.69, 3.00) | 5.84 (3.70, 10.16) | 6.03 (4.15, 10.49) | 199.98 (58.85, 956.49) | <0.001 |
| TC, mmol/L | 3.76 (3.42, 4.31) | 4.99 (4.26, 5.81) | 4.96 (4.06, 5.71) | 5.00 (4.09, 6.10) | <0.001 |
| TG, mmol/L | 1.37 (1.17, 1.49) | 1.44 (1.04, 2.06) | 1.53 (1.12, 2.26) | 1.64 (1.21, 2.33) | <0.001 |
| LDL-c, mmol/L | 2.51 (1.86, 2.83) | 3.26 (2.73, 3.92) | 3.20 (2.57, 3.83) | 3.29 (2.61, 4.07) | <0.001 |
| HDL-c, mmol/L | 1.13 (0.84, 1.21) | 1.20 (1.05, 1.38) | 1.19 (1.00, 1.40) | 1.17 (0.98, 1.38) | 0.003 |
| Calcium, mmol/L | 2.36 (0.05) | 2.37 (0.11) | 2.36 (0.11) | 2.31 (0.18) | <0.001 |
| Phosphorus, mmol/L | 1.17 (0.11) | 1.17 (0.16) | 1.19 (0.17) | 1.28 (0.28) | <0.001 |
| CRP, mg/L | 0.50 (0.50, 0.50) | 0.50 (0.50, 0.50) | 0.50 (0.50, 0.88) | 0.50 (0.50, 1.40) | <0.001 |
| 24h urine biochemistry |  |  |  |  |  |
| Urine protein quantification, g/24h | 0.02 (0.01, 0.05) | 0.05 (0.03, 0.08) | 0.04 (0.03, 0.08) | 0.89 (0.2, 4.01) | <0.001 |
| Urine microalbumin quantification, mg/24h | 6.00 (3.05, 8.63) | 8.01 (5.60, 12.80) | 7.78 (6.25, 13.08) | 403.81 (82.125, 2164.98) | <0.001 |
| Urine calcium quantification, mmol/24h | 6.02 (3.85, 8.56) | 5.96 (3.94, 8.22) | 5.88 (3.71, 7.81) | 2.78 (0.99, 5.28) | <0.001 |
| Urine phosphorus quantification, mmol/24h | 20.36 (15.97, 24.35) | 21.51 (15.67, 25.89) | 22.09 (16.66, 29.01) | 20.67 (14.88, 26.03) | 0.044 |
| NGAL, U/L | 5.42 (3.53, 7.65) | 9.35 (6.45, 17.60) | 10.40 (6.20, 14.80) | 15.50 (10.00, 28.60) | <0.001 |
| RBP, mg/L | 0.02 (0.01, 0.07) | 0.05 (0.02, 0.30) | 0.06 (0.02, 0.23) | 4.05 (1.01, 18.72) | <0.001 |
| UTF, mg/L | 0.25 (0.15, 0.38) | 0.61 (0.36, 1.07) | 0.82 (0.44, 1.18) | 10.68 (3.07, 40.95) | <0.001 |
| Uβ2-MG, mg/L | 0.15 (0.02, 0.24) | 0.17 (0.03, 0.38) | 0.21 (0.07, 0.41) | 0.47 (0.22, 1.33) | <0.001 |
| UIgG, mg/L | 1.03 (0.08, 1.63) | 2.16 (0.94, 5.74) | 2.75 (1.20, 5.35) | 26.10 (8.87, 108.96) | <0.001 |

Normally distributed data were presented as mean ± standard deviation, with group comparisons analyzed using a t-test. Non-normally distributed data were displayed as median and quartile, with group comparisons assessed using non-parametric tests. Count data were shown as n (%), and comparisons between groups were made using chi-square test or Fisher's exact test. A two-sided P < 0.05 was considered statistically significant.

**Abbreviations:** DKD: diabetic kidney disease; T2DM: type 2 diabetes mellitus; HR-DKD: high-risk diabetic kidney disease; HC: healthy control; SGLT-2: sodium-glucose transporter 2; GLP-1: glucagon-like peptide-1; BMI: body mass index; WHratio: waist-to-hip ratio; Systolic BP: systolic blood pressure; Diastolic BP: diastolic blood pressure; PLT: platelet; NEUT: neutrophil; LYM: lymphocyte; MONO: monocyte; FBG: fasting blood glucose; HbA1c: glycated haemoglobin; TSH: thyroid stimulating hormone; ALT: alglutaminase; AST: alglutaminase; γ-GGT: γ-glutamyl transpeptidase; TBIL: total bilirubin; ALB: albumin; BUN: blood urea nitrogen; UA: blood uric acid; eGFR: estimated glomerular filtration rate; ACR: urine albumin to creatinine ratio; TC: total cholesterol; TG: triglycerides; LDL-c: low-density lipoprotein cholesterol; HDL-c: high-density lipoprotein cholesterol; CRP: C-reactive protein; NGAL: N. acetylaminoglucosidase; RBP: retinol-binding protein; UTF: urinary transferrin quantification; β2MG: urinary β2 microglobulin; UIgG: urinary immunoglobulin G.

###

#### Supplementary **Table 16. Baseline demographic characteristics of the training and testing group**

| Characteristics | Training (N = 1,063) | Testing (N = 437) | *P* value |
| --- | --- | --- | --- |
| HC Group, N(%) | 20 (1.88) | 10 (2.29) | 0.526 |
| T2DM Group, N(%) | 246 (23.14) | 115 (26.32) |  |
| HR-DKD Group, N(%) | 396 (37.25) | 159 (36.38) |  |
| DKD Group, N(%) | 401 (37.72) | 153 (35.01) |  |
| Ethnicity (Han), N(%) | 1044 (98.21) | 427 (97.71) | 0.522 |
| Marital status (married), N(%) | 1031 (96.99) | 426 (97.48) | 0.396 |
| Sex (male), N(%) | 622 (58.51) | 268 (61.33) | 0.313 |
| Age, years | 59.00 (50.00, 66.00) | 58.00 (49.00, 65.00) | 0.274 |
| Smoking, N(%) | 275 (25.87) | 92 (21.05) | 0.049 |
| Drinking, N(%) | 197 (18.53) | 78 (17.85) | 0.756 |
| Hypertension, N(%) | 513 (48.26) | 180 (41.19) | 0.013 |
| Hyperlipidemia, N(%) | 872 (82.03) | 335 (76.66) | 0.017 |
| Coronary heart disease, N(%) | 386 (36.31) | 139 (31.81) | 0.097 |
| Fatty liver disease, N(%) | 389 (36.59) | 130 (29.75) | 0.011 |
| Diabetic retinopathy, N(%) | 370 (34.81) | 137 (31.35) | 0.198 |
| Obesity, N(%) | 330 (31.04) | 137 (31.35) | 0.903 |
| Duration of T2DM, years | 9.00 (2.00, 15.00) | 8.00 (2.00, 15.00) | 0.282 |
| BMI, kg/m^2^ | 26.42 (3.77) | 26.47 (4.27) | 0.858 |
| WHratio | 0.93 (0.06) | 0.94 (0.06) | 0.700 |
| Systolic BP, mmHg | 133.36 (16.80) | 131.97 (15.85) | 0.241 |
| Diastolic BP, mmHg | 79.98 (10.24) | 79.07 (9.32) | 0.075 |
| Medications, N(%) |  |  |  |
| Aspirin | 237 (22.30) | 99 (22.65) | 0.892 |
| β-blocker | 195 (18.34) | 64 (14.65) | 0.098 |
| Finerenone | 590 (55.50) | 216 (49.43) | 0.035 |
| Lipid-lowering medication | 15 (3.43) | 43 (4.05) | 0.660 |
| Anti-hypertensive drugs | 418 (39.32) | 145 (33.18) | 0.026 |
| SGLT-2 inhibitors | 502 (47.22) | 204 (46.68) | 0.865 |
| GLP-1 agonists | 188 (17.69) | 87 (19.91) | 0.340 |
| Metformin | 554 (52.12) | 234 (53.55) | 0.649 |
| Insulin | 516 (48.54) | 186 (42.56) | 0.035 |
| Blood biochemical indicators |  |  |  |
| MONO, *10^9/L | 0.46 (0.13) | 0.46 (0.13) | 0.340 |
| FBG, mmol/L | 8.20 (2.90) | 8.46 (3.19) | 0.362 |
| HbA1c, % | 7.80 (2.05) | 7.72 (1.77) | 0.640 |
| BUN, mmol/L | 7.02 (4.06) | 6.83 (4.21) | 0.108 |
| UA, μmol/L | 331.40 (270.20, 401.10) | 329.80 (272.20, 387.30) | 0.245 |
| Baseline eGFR, ml/min/1.73m^2^ | 90.62 (27.34) | 94.84 (25.25) | 0.004 |
| Baseline ACR, mg/g | 10.91 (4.97, 86.72) | 9.85 (4.59, 71.40) | 0.235 |
| TC, mmol/L | 4.92 (4.12, 5.86) | 5.07 (4.12, 5.86) | 0.679 |
| TG, mmol/L | 1.54 (1.13, 2.26) | 1.51 (1.12, 2.16) | 0.404 |
| LDL-c, mmol/L | 3.20 (2.61, 3.93) | 3.29 (2.61, 3.96) | 0.516 |
| HDL-c, mmol/L | 1.18 (1.00, 1.38) | 1.18 (1.02, 1.37) | 0.496 |
| Ca, mmol/L | 2.34 (0.14) | 2.34 (0.16) | 0.865 |
| P, mmol/L | 1.22 (0.22) | 1.21 (0.21) | 0.147 |
| 24h urine biochemistry |  |  |  |
| Urine protein quantification, g/24h | 0.11 (0.04, 0.84) | 0.11 (0.04, 1.23) | 0.887 |
| Urine microalbumin quantification, mg/24h | 20.48 (7.54, 309.50) | 20.80 (7.22, 585.23) | 0.978 |
| Urine calcium quantification, mmol/24h | 4.37 (2.08, 6.67) | 4.94 (2.51, 7.92) | 0.020 |
| Urine phosphorus quantification, mmol/24h | 21.02 (15.58, 26.39) | 22.41 (16.18, 28.95) | 0.176 |
| NGAL, U/L | 12.2 (7.30, 20.60) | 12.45 (7.45, 21.45) | 0.799 |
| RBP, mg/L | 0.33 (0.04, 3.39) | 0.40 (0.05, 4.90) | 0.415 |
| UTF, mg/L | 1.29 (0.59, 9.00) | 1.40 (0.69, 14.32) | 0.264 |
| Uβ2-MG, mg/L | 0.31 (0.12, 0.70) | 0.31 (0.11, 0.62) | 0.553 |
| UIgG, mg/L | 6.16 (2.22, 25.04) | 6.70 (2.05, 29.47) | 0.851 |
| Blood sample, N(%) | 1061 (99.81) | 431 (98.63) |  |
| Urine sample, N(%) | 1012 (95.20) | 410 (93.82) |  |
| Blood and urine sample, N(%) | 1011 (71.25) | 408 (28.75) |  |

Normally distributed data were expressed as mean ± standard deviation (SD), and corrected one-way ANOVA was used for between-group comparisons; non-normally distributed data were expressed as median and quartiles, and Kruskal-Wallis H test was used for between-group comparisons. Count data were expressed as N (%), and the Fisher exact test was used for between-group comparisons. A difference was considered statistically significant at a two-sided P < 0.05. eGFR decline slope 2014 refers to the rate and percentage of decline in eGFR per year for patients from 2014 to the follow-up endpoint, and eGFR decline slope 2022 refers to the rate and percentage of decline in eGFR per year for patients from 2022 to the follow-up endpoint.

**Abbreviations:** DKD: diabetic kidney disease; T2DM: type 2 diabetes mellitus; HR-DKD: high-risk diabetic kidney disease; HC: healthy control; SGLT-2: sodium-glucose transporter 2; GLP-1: glucagon-like peptide-1; BMI: body mass index; WHratio: waist-to-hip ratio; Systolic BP: systolic blood pressure; Diastolic BP: diastolic blood pressure; PLT: platelet; NEUT: neutrophil; LYM: lymphocyte; MONO: monocyte; FBG: fasting blood glucose; HbA1c: glycated haemoglobin; TSH: thyroid stimulating hormone; ALT: alglutaminase; AST: alglutaminase; γ-GGT: γ-glutamyl transpeptidase; TBIL: total bilirubin; ALB: albumin; BUN: blood urea nitrogen; UA: blood uric acid; eGFR: estimated glomerular filtration rate; ACR: urine albumin to creatinine ratio; TC: total cholesterol; TG: triglycerides; LDL-c: low-density lipoprotein cholesterol; HDL-c: high-density lipoprotein cholesterol; CRP: C-reactive protein; NGAL: N. acetylaminoglucosidase; RBP: retinol-binding protein; UTF: urinary transferrin quantification; β2MG: urinary β2 microglobulin; UIgG: urinary immunoglobulin G.

### Section 1.13. Inclusion Indicators for Different Combinations of Classification Models

A total of 7 prediction models were developed by integrating various data sources, including clinical indicators, urine protein omics, urine metabolomics, blood lipidomics, and combinations of these data sets. The models developed include clinical indicator prediction models, urine protein omics prediction models, urine metabolomics prediction models, blood lipidomics prediction models, prediction models combining urine protein and metabolomics, prediction models combining blood lipidomics with urine protein and metabolomics, three-omics combination prediction models of blood and urine, and three-omics combination prediction models of blood and urine along with clinical indicators.

#### Supplementary Table 17. Summary of inclusion indicators for different combinations of classification models

| Predictive Model | Inclusion indicators |
| --- | --- |
| Clinical Indicators | Clinical Risk Factor Score, Metformin, Aspirin, Waist-to-Hip Ratio, Systolic Blood Pressure, Diastolic Blood Pressure, Calcium Urine, HbA1c, LDL-c |
| Urine Proteomics | CD300LF，CST4，MMRN2, SERPINA1 |
| Urine Metabolomics | L-glutamic acid, dimethyl ester |
| Blood Lipidomics | PC |
| Urine Proteomics + Metabolomics | L-glutamic acid, dimethyl ester，CD300LF，CST4，MMRN2及SERPINA1 |
| Blood + Urine Triomics | PC，L-glutamic acid, dimethyl ester，CD300LF，CST4，MMRN2及SERPINA1 |
| Blood + Urine Triomics + Clinical Indicators | Clinical Risk Factor Score, Metformin, Aspirin, Waist-to-Hip Ratio, Systolic Blood Pressure, Diastolic Blood Pressure, Calcium Urine, HbA1c, LDL-c, PC, L-glutamic acid dimethyl ester, CD300LF, CST4, MMRN2, SERPINA1 |

PC, L-glutamic acid, dimethyl ester, CD300LF, CST4, MMRN2 and SERPINA1 are averages collected at multiple time points.

### Section 1.14. Summary of Evaluation Indicators for Classification Models

#### Supplementary Table 18. Summary of evaluation indicators for classification models

| Predictive models | | Clinical Indicators | Urine Proteomics | Urine Metabolomics | Blood Lipidomics | Urine Proteomics + Metabolomics | Blood + Urine Triomics | Blood + Urine Triomics + Clinical Indicators |
| --- | --- | --- | --- | --- | --- | --- | --- | --- |
| Accuracy |  | 0.611 | 0.684 | 0.650 | 0.522 | 0.735 | 0.737 | 0.923 |
|  | 95% CI | (0.562, 0.658) | （0.638, 0.728） | (0.603, 0.695) | (0.472, 0.571) | (0.691, 0.776) | (0.692, 0.779) | (0.893, 0.947) |
| AUC | NC | 0.999 | 0.999 | 0.999 | 0.995 | 0.999 | 0.999 | 0.999 |
|  | DM | 0.959 | 0.808 | 0.755 | 0.599 | 0.809 | 0.821 | 0.994 |
|  | HRDKD | 0.771 | 0.795 | 0.775 | 0.668 | 0.818 | 0.834 | 0.986 |
|  | DKD | 0.619 | 0.942 | 0.935 | 0.714 | 0.957 | 0.972 | 0.972 |
| Sensitivity | NC | 0.999 | 0.999 | 0.999 | 0.999 | 0.999 | 0.999 | 0.999 |
|  | DM | 0.891 | 0.312 | 0.413 | 0.372 | 0.431 | 0.434 | 0.957 |
|  | HRDKD | 0.597 | 0.756 | 0.644 | 0.513 | 0.763 | 0.761 | 0.937 |
|  | DKD | 0.442 | 0.829 | 0.804 | 0.588 | 0.911 | 0.884 | 0.885 |
| Specificity | NC | 0.997 | 0.999 | 0.999 | 0.998 | 0.999 | 0.999 | 0.999 |
|  | DM | 0.932 | 0.911 | 0.840 | 0.851 | 0.923 | 0.904 | 0.972 |
|  | HRDKD | 0.745 | 0.664 | 0.712 | 0.705 | 0.737 | 0.757 | 0.965 |
|  | DKD | 0.717 | 0.928 | 0.924 | 0.719 | 0.938 | 0.938 | 0.946 |
| Precision | NC | 0.875 | 0.999 | 0.999 | 0.917 | 0.999 | 0.999 | 0.999 |
|  | DM | 0.788 | 0.312 | 0.464 | 0.422 | 0.653 | 0.557 | 0.907 |
|  | HRDKD | 0.594 | 0.756 | 0.566 | 0.516 | 0.629 | 0.661 | 0.943 |
|  | DKD | 0.486 | 0.829 | 0.858 | 0.549 | 0.894 | 0.896 | 0.908 |
| Recall | NC | 0.999 | 0.999 | 0.999 | 0.999 | 0.999 | 0.999 | 0.999 |
|  | DM | 0.891 | 0.312 | 0.413 | 0.372 | 0.431 | 0.424 | 0.957 |
|  | HRDKD | 0.597 | 0.756 | 0.644 | 0.513 | 0.763 | 0.761 | 0.937 |
|  | DKD | 0.442 | 0.829 | 0.804 | 0.588 | 0.911 | 0.884 | 0.885 |

### Section 1.15. Baseline Data of the Training and Testing Group for the Risk-prognostic Models.

Participants were divided into a training set and a test set at a ratio of 3:1. Detailed baseline demographic characteristics are presented in Tables 2-5, including age, gender, ethnicity, marital status, group size, smoking and drinking habits, medical history (hypertension, coronary heart disease, diabetic retinopathy, obesity), duration of type 2 diabetes, BMI, waist-to-hip ratio, blood pressure, and medication. No significant differences were observed between the two groups in terms of demographic and medical history variables (P > 0.05). Analysis of blood routine and biochemical indicators revealed no significant variations in blood routine parameters, urea nitrogen, uric acid, baseline eGFR, total blood lipids, blood calcium, and phosphorus levels between the training and test sets (P > 0.05). Similarly, there were no significant differences in most urine biochemical indicators, except for NGAL (P > 0.05). Overall, the lack of statistical variance in most indicators between the training and test sets allows for the further development and validation of the model.

#### Supplementary Table 19. Baseline demographic characteristics and biochemical indicators for the training and testing Groups

| Characteristics | Training (N = 641) | Testing (N = 278) | *P* value |
| --- | --- | --- | --- |
| HC Group, N(%) |  |  |  |
| T2DM Group, N(%) | 156 (24.34) | 75 (26.98) | 0.353 |
| HR-DKD Group, N(%) | 215 (33.54) | 100 (35.97) |  |
| DKD Group, N(%) | 270 (42.12) | 103 (37.05) |  |
| Ethnicity (Han), N(%) | 633 (98.75) | 271 (97.48) | 0.167 |
| Marital status (married), N(%) | 623 (97.19) | 274 (98.56) | 0.439 |
| Sex (male), N(%) | 378 (58.97) | 164 (58.99) | 1.000 |
| Age, years | 59.00 (50.00, 66.00) | 58.00 (50.00, 65.00) | 0.377 |
| Smoking, N(%) | 145 (22.62) | 70 (25.18) | 0.398 |
| Drinking, N(%) | 103 (16.07) | 51 (18.35) | 0.389 |
| Hypertension, N(%) | 287 (44.77) | 121 (43.53) | 0.773 |
| Hyperlipidemia, N(%) | 513 (80.03) | 230 (82.73) | 0.362 |
| Coronary heart disease, N(%) | 216 (33.70) | 97 (34.89) | 0.762 |
| Fatty liver disease, N(%) | 215 (33.54) | 91 (32.73) | 0.820 |
| Diabetic retinopathy, N(%) | 222 (34.63) | 90 (32.37) | 0.544 |
| Obesity, N(%) | 218 (34.01) | 88 (31.65) | 0.494 |
| Duration of T2DM, years | 9.00 (4.00, 15.00) | 9.00 (3.00, 15.00) | 0.685 |
| BMI, kg/m^2^ | 26.70 (3.85) | 26.71 (3.88) | 0.874 |
| WHratio | 0.94 (0.06) | 0.93 (0.07) | 0.564 |
| Systolic BP, mmHg | 133.52 (16.74) | 133.99 (16.60) | 0.806 |
| Diastolic BP, mmHg | 79.64 (9.90) | 79.71 (10.54) | 0.759 |
| Medications, N(%) |  |  |  |
| Aspirin | 147 (22.93) | 58 (20.86) | 0.546 |
| β-blocker | 108 (16.85) | 50 (17.99) | 0.704 |
| Finerenone | 342 (53.35) | 154 (55.40) | 0.614 |
| Lipid-lowering medication | 25 (3.90) | 10 (3.60) | 1.000 |
| Anti-hypertensive drugs | 238 (37.13) | 91 (32.73) | 0.231 |
| SGLT-2 inhibitors | 330 (51.48) | 141 (50.72) | 0.886 |
| GLP-1 agonists | 126 (19.66) | 54 (19.42) | 1.000 |
| Metformin | 362 (56.47) | 141 (50.72) | 0.113 |
| Insulin | 305 (47.58) | 123 (44.24) | 0.388 |
| Blood biochemical indicators |  |  |  |
| MONO, *10^9/L | 0.46 (0.13) | 0.47 (0.14) | 0.427 |
| FBG, mmol/L | 8.42 (2.97) | 7.77 (2.56) | 0.006 |
| HbA1c, % | 7.67 (2.16) | 7.49 (1.49) | 0.417 |
| BUN, mmol/L | 7.25 (4.54) | 6.79 (3.58) | 0.161 |
| UA, μmol/L | 329.80 (274.70, 395.20) | 339.05 (271.90, 406.10) | 0.477 |
| Baseline eGFR, ml/min/1.73m^2^ | 89.58 (27.86) | 91.81 (25.98) | 0.244 |
| eGFR decline slope 2014 | 1.99 (0.25, 5.19) | 1.23 (-0.22, 4.37) | 0.034 |
| eGFR decline slope 2022 | 1.28 (0, 6.42) | 0.57 (0, 5.87) | 0.505 |
| Baseline ACR, mg/g | 12.35 (5.25, 161.84) | 11.39 (5.38, 91.93) | 0.491 |
| TC, mmol/L | 4.85 (4.03, 5.71) | 4.90 (4.04, 5.90) | 0.747 |
| TG, mmol/L | 1.52 (1.12, 2.17) | 1.58 (1.12, 2.32) | 0.566 |
| LDL-c, mmol/L | 3.17 (2.54, 3.86) | 3.16 (2.56, 3.93) | 0.891 |
| HDL-c, mmol/L | 1.18 (1.00, 1.38) | 1.16 (0.99, 1.38) | 0.848 |
| Ca, mmol/L | 2.35 (0.13) | 2.34 (0.15) | 0.842 |
| P, mmol/L | 1.23 (0.24) | 1.21 (0.22) | 0.566 |
| 24h urine biochemistry |  |  |  |
| Urine protein quantification, g/24h | 0.11 (0.05, 1.70) | 0.11 (0.05, 1.09) | 0.177 |
| Urine microalbumin quantification, mg/24h | 58.58 (8.75, 905.16) | 24.335 (8.40, 393.99) | 0.301 |
| Urine calcium quantification, mmol/24h | 3.92 (1.68, 6.60) | 3.80 (1.83, 6.56) | 0.960 |
| Urine phosphorus quantification, mmol/24h | 21.93 (15.48, 27.77) | 21.48 (15.58, 28.62) | 0.912 |
| NGAL, U/L | 12.50 (8.70, 22.90) | 11.60 (6.40, 19.30) | 0.039 |
| RBP, mg/L | 0.84 (0.09, 7.76) | 0.04 (0.10, 3.77) | 0.137 |
| UTF, mg/L | 2.02 (0.68, 20.31) | 1.49 (0.44, 7.76) | 0.058 |
| Uβ2-MG, mg/L | 0.31 (0.11, 0.70) | 0.35 (0.10, 0.68) | 0.718 |
| UIgG, mg/L | 8.99 (2.73, 54.01) | 6.01 (2.00, 27.76) | 0.085 |

Normally distributed data were expressed as mean ± standard deviation (SD), and corrected one-way ANOVA was used for between-group comparisons; non-normally distributed data were expressed as median and quartiles, and Kruskal-Wallis H test was used for between-group comparisons. Count data were expressed as N (%), and the Fisher exact test was used for between-group comparisons. A difference was considered statistically significant at a two-sided P < 0.05. eGFR decline slope 2014 refers to the rate and percentage of decline in eGFR per year for patients from 2014 to the follow-up endpoint, and eGFR decline slope 2022 refers to the rate and percentage of decline in eGFR per year for patients from 2022 to the follow-up endpoint.

**Abbreviations:** DKD: diabetic kidney disease; T2DM: type 2 diabetes mellitus; HR-DKD: high-risk diabetic kidney disease; HC: healthy control; SGLT-2: sodium-glucose transporter 2; GLP-1: glucagon-like peptide-1; BMI: body mass index; WHratio: waist-to-hip ratio; Systolic BP: systolic blood pressure; Diastolic BP: diastolic blood pressure; PLT: platelet; NEUT: neutrophil; LYM: lymphocyte; MONO: monocyte; FBG: fasting blood glucose; HbA1c: glycated haemoglobin; TSH: thyroid stimulating hormone; ALT: alglutaminase; AST: alglutaminase; γ-GGT: γ-glutamyl transpeptidase; TBIL: total bilirubin; ALB: albumin; BUN: blood urea nitrogen; UA: blood uric acid; eGFR: estimated glomerular filtration rate; ACR: urine albumin to creatinine ratio; TC: total cholesterol; TG: triglycerides; LDL-c: low-density lipoprotein cholesterol; HDL-c: high-density lipoprotein cholesterol; CRP: C-reactive protein; NGAL: N. acetylaminoglucosidase; RBP: retinol-binding protein; UTF: urinary transferrin quantification; β2MG: urinary β2 microglobulin; UIgG: urinary immunoglobulin G.

### Section 1.16. Establish and Validate COX risk Prognostic Models based on Multi-omics Data

#### **Supplementary Table 20. Baseline demographic characteristics and biochemical indicators for the prospective cohort study**

| Characteristics | Composite Endpoint | | *P* value |
| --- | --- | --- | --- |
|  | No (N = 526) | Yes (N = 393) |  |
| Ethnicity (Han), N(%) | 519 (98.67) | 385 (97.96) | 0.439 |
| Marital status (married), N(%) | 513 (97.53) | 384 (97.71) | 0.271 |
| Sex (male), N(%) | 308 (58.56) | 234 (59.54) | 0.786 |
| Age, years | 59.00 (51.00, 66.00) | 59.00 (49.00, 65.00) | 0.225 |
| High-risk DKD, N(%) | 347 (65.97) | 285 (72.52) | 0.034 |
| Smoking, N(%) | 116 (22.05) | 99 (25.19) | 0.271 |
| Drinking, N(%) | 79 (15.02) | 75 (19.08) | 0.109 |
| Hypertension, N(%) | 224 (42.59) | 184 (46.82) | 0.203 |
| Hyperlipidemia, N(%) | 427 (81.18) | 316 (80.41) | 0.800 |
| Coronary heart disease, N(%) | 180 (34.22) | 133 (33.84) | 0.944 |
| Fatty liver disease, N(%) | 182 (34.60) | 124 (31.55) | 0.358 |
| Diabetic retinopathy, N(%) | 165 (31.37) | 147 (37.40) | 0.056 |
| Obesity, N(%) | 165 (31.37) | 141 (35.88) | 0.086 |
| Duration of T2DM, years | 9.00 (3.00, 15.00) | 9.00 (3.00, 15.00) | 0.331 |
| BMI, kg/m^2^ | 26.54 (3.69) | 26.93 (4.06) | 0.331 |
| WHratio | 0.94 (0.06) | 0.94 (0.07) | 0.723 |
| Systolic BP, mmHg | 133.86 (17.22) | 133.40 (15.96) | 0.863 |
| Diastolic BP, mmHg | 79.90 (9.73) | 79.33 (10.56) | 0.342 |
| Medications |  |  |  |
| Aspirin | 106 (20.15) | 99 (25.19) | 0.078 |
| β-blocker | 88 (16.73) | 70 (17.81) | 0.724 |
| Finerenone | 20 (3.80) | 15 (3.82) | 1.000 |
| Lipid-lowering medication | 276 (52.47) | 220 (55.98) | 0.316 |
| Anti-hypertensive drugs | 176 (33.46) | 153 (38.93) | 0.095 |
| SGLT-2 inhibitors | 167 (53.02) | 190 (50.94) | 0.693 |
| GLP-1 agonists | 98 (18.63) | 82 (20.87) | 0.402 |
| Metformin | 281 (53.42) | 222 (56.49) | 0.384 |
| Insulin | 228 (43.35) | 200 (50.89) | 0.027 |
| Blood biochemical indicators |  |  |  |
| PLT, *10^9/L | 238.79 (61.46) | 243.66 (64.04) | 0.290 |
| NEUT, *10^9/L | 4.41 (1.54) | 4.47 (1.60) | 0.627 |
| LYM, *10^9/L | 2.00 (0.81) | 1.97 (0.64) | 0.870 |
| MONO, *10^9/L | 0.46 (0.13) | 0.46 (0.13) | 0.698 |
| FBG, mmol/L | 8.28 (2.92) | 8.16 (2.79) | 0.661 |
| HbA1c, % | 7.69 (2.16) | 7.50 (1.70) | 0.063 |
| TSH, mIU/L | 2.13 (1.32, 2.68) | 2.13 (1.42, 2.68) | 0.296 |
| ALT, U/L | 20.65 (15.00,29.20) | 20.70 (13.60, 30.50) | 0.802 |
| AST, U/L | 20.90 (17.10, 25.80) | 21.00 (17.00, 27.20) | 0.309 |
| GGT, U/L | 24.60 (18.40, 36.10) | 24.00 (17.50, 38.10) | 0.415 |
| TBIL, umol/L | 13.44 (10.43, 16.96) | 13.33 (10.25, 16.67) | 0.438 |
| ALB, g/L | 44.15 (5.97) | 43.85 (6.77) | 0.379 |
| BUN, mmol/L | 7.06 (4.44) | 7.19 (4.05) | 0.346 |
| UA, umol/L | 332.90 (271.90, 400.50) | 333.70 (279.40, 394.30) | 0.943 |
| Baseline eGFR, ml/min/1.73m^2^ | 89.50 (27.18) | 91.26 (27.49) | 0.201 |
| eGFR decline slope 2014 | 0.44 (-0.92, 1.83) | 4.36 (1.71, 8.98) | <0.001 |
| eGFR decline slope 2022 | 0.00 (-0.57, 0.07) | 7.58 (4.38, 12.76) | <0.001 |
| Baseline ACR, mg/g | 11.39 (5.10, 105.72) | 12.95 (5.53, 199.34) | 0.109 |
| TC, mmol/L | 4.88 (3.99, 5.77) | 4.85 (4.15, 5.73) | 0.593 |
| TG, mmol/L | 1.50 (1.12, 2.09) | 1.57 (1.10, 2.33) | 0.233 |
| LDL-c, mmol/L | 3.18 (2.52, 3.88) | 3.14 (2.59, 3.91) | 0.863 |
| HDL-c, mmol/L | 1.17 (0.99, 1.38) | 1.18 (1.01, 1.37) | 0.892 |
| Calcium, mmol/L | 2.35 (0.14) | 2.34 (0.14) | 0.407 |
| Phosphorus, mmol/L | 1.21 (0.23) | 1.22 (0.21) | 0.781 |
| CRP, mg/L | 0.50 (0.50, 0.74) | 0.50 (0.50, 0.60) | 0.532 |
| 24h urine biochemistry |  |  |  |
| Urine protein quantification, g/24h | 0.12 (0.05, 1.11) | 0.26 (0.06, 2.23) | 0.003 |
| Urine microalbumin quantification, mg/24h | 24.38 (8.01, 530.52) | 107.16 (11.34, 1185.95) | 0.004 |
| Urine glucose quantification, g/24h | 14.88 (1.51, 31.82) | 8.89 (1.03, 31.96) | 0.652 |
| Urine calcium quantification, mmol/24h | 4.05 (1.92, 6.55) | 3.58 (1.48, 6.63) | 0.796 |
| NGAL, U/L | 12.30 (8.10, 21.50) | 12.40 (7.30, 21.70) | 0.934 |
| RBP, mg/L | 0.45 (0.10, 5.57) | 0.83 (0.09, 7.88) | 0.152 |
| UTF, mg/L | 1.41 (0.53, 11.04) | 2.94 (0.72, 21.95) | 0.023 |
| Uβ2-MG, mg/L | 0.30 (0.37, 0.11) | 0.37 (0.11, 0.93) | 0.301 |
| UIgG, mg/L | 8.03 (2.03, 27.76) | 8.71 (3.02, 65.99) | 0.109 |

Normally distributed data were expressed as mean ± standard deviation (SD), and corrected one-way ANOVA was used for between-group comparisons; non-normally distributed data were expressed as median and quartiles, and Kruskal-Wallis H test was used for between-group comparisons. Count data were expressed as N (%), and the Fisher exact test was used for between-group comparisons. A difference was considered statistically significant at a two-sided P < 0.05. eGFR decline slope 2014 refers to the rate and percentage of decline in eGFR per year for patients from 2014 to the follow-up endpoint, and eGFR decline slope 2022 refers to the rate and percentage of decline in eGFR per year for patients from 2022 to the follow-up endpoint.

**Abbreviations:** DKD: diabetic kidney disease; T2DM: type 2 diabetes mellitus; HR-DKD: high-risk diabetic kidney disease; HC: healthy control; SGLT-2: sodium-glucose transporter 2; GLP-1: glucagon-like peptide-1; BMI: body mass index; WHratio: waist-to-hip ratio; Systolic BP: systolic blood pressure; Diastolic BP: diastolic blood pressure; PLT: platelet; NEUT: neutrophil; LYM: lymphocyte; MONO: monocyte; FBG: fasting blood glucose; HbA1c: glycated haemoglobin; TSH: thyroid stimulating hormone; ALT: alglutaminase; AST: alglutaminase; γ-GGT: γ-glutamyl transpeptidase; TBIL: total bilirubin; ALB: albumin; BUN: blood urea nitrogen; UA: blood uric acid; eGFR: estimated glomerular filtration rate; ACR: urine albumin to creatinine ratio; TC: total cholesterol; TG: triglycerides; LDL-c: low-density lipoprotein cholesterol; HDL-c: high-density lipoprotein cholesterol; CRP: C-reactive protein; NGAL: N. acetylaminoglucosidase; RBP: retinol-binding protein; UTF: urinary transferrin quantification; β2MG: urinary β2 microglobulin; UIgG: urinary immunoglobulin G.

#### **Supplementary Table 21. COX proportional risk regression analysis of multiomics indicators and outcome events**

| Models | | CD300LF | CST4 | MMRN2 | SERPINA1 | L-glutamic acid, dimethyl ester | Phosphatidylcholine |
| --- | --- | --- | --- | --- | --- | --- | --- |
| Crude Model 1^a^ | HR | 1.010 | 1.018 | 0.946 | 1.003 | 1.027 | 1.003 |
|  | 95% CI | 1.007-1.013 | 1.014-1.023 | 0.934-0.959 | 1.002-1.003 | 1.020-1.035 | 1.002-1.004 |
|  | *P* value | <0.001 | <0.001 | <0.001 | <0.001 | <0.001 | <0.001 |
| Multivariable Model 2^b^ | HR | 1.010 | 1.019 | 0.944 | 1.003 | 1.028 | 1.003 |
|  | 95% CI | 1.007-1.014 | 1.015-1.024 | 0.932-0.957 | 1.002-1.003 | 1.020-1.036 | 1.002-1.004 |
|  | *P* value | <0.001 | <0.001 | <0.001 | <0.001 | <0.001 | <0.001 |
| Multivariable Model 3^c^ | HR | 1.008 | 1.033 | 0.926 | 1.007 | 1.044 | 1.003 |
|  | 95% CI | 1.002-1.013 | 1.023-1.043 | 0.901-0.953 | 1.004-1.009 | 1.027-1.061 | 1.001-1.005 |
|  | *P* value | 0.007 | <0.001 | <0.001 | <0.001 | <0.001 | 0.014 |
| Multivariable Model 4^d^ | HR | 1.004 | 1.034 | 0.932 | 1.006 | 1.041 | 1.002 |
|  | 95% CI | 0.996-1.011 | 1.018-1.050 | 0.895-0.970 | 1.002-1.009 | 1.016-1.067 | 0.999-1.005 |
|  | *P* value | 0.335 | <0.001 | 0.001 | 0.001 | 0.001 | 0.128 |

^a^Not adjusted; ^b^Adjusted for age and sex; ^c^Adjusted for age, sex, number of risk factors, HbA1c, urinary calcium, LDLc, and WHratio; ^d^Based on model 3, adjusted for baseline ACR, baseline eGFR, 24h urinary albumin, 24h urinary microalbumin, urinary transferrin, N. acetylaminoglucosidase, retinol-binding protein, β2-microglobulin, immunoglobulin G.

Three prediction models were developed based on the screening of clinical and multi-omics indicators. These models include the omics biomarker model (Model 1), clinical indicators model (Model 2), and joint prediction model (Model 3).

#### Supplementary Table 22. Indicators used in prognostic risk prediction models

| Predictive Model | Indicators |
| --- | --- |
| Model 1: Blood + urine triple omics | PC，L-glutamic acid, dimethyl ester，CD300LF，CST4，MMRN2, and SERPINA1 |
| Model 2: Clinical indicators | Clinical Risk Factor Score, Metformin, Aspirin, Waist-to-Hip Ratio, Systolic Blood Pressure, Diastolic Blood Pressure, Calcium Urine, HbA1c，LDL-c |
| Model 3: Blood + urine omics + clinical indicators | Clinical Risk Factor Score, Metformin, Aspirin, Waist-to-Hip Ratio, Systolic Blood Pressure, Diastolic Blood Pressure, Calcium Urine, HbA1c，LDL-c，PC，L-glutamic acid, dimethyl ester，CD300LF，CST4，MMRN2, and SERPINA1 |

### Section 2.1. Results of Quality Control for Proteomics


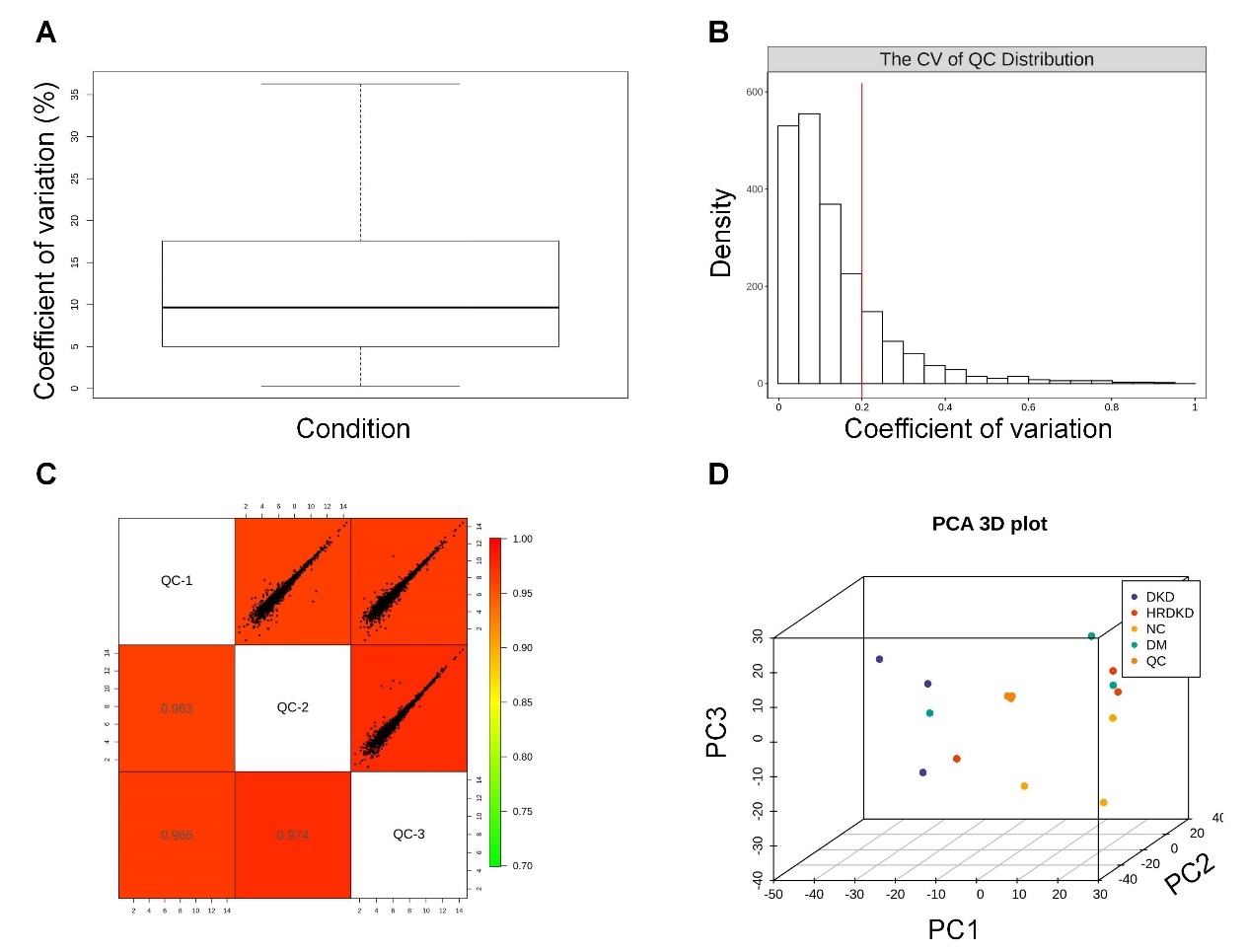


#### Supplementary Figure 1. Evaluation of QC samples

1. Box line plot of CV value distribution of QC; B. CV value distribution of QC; C. Correlation analysis plot of QC sample; D. 3D PCA plot of QC sample.

Coefficient of variation (CV), principal component analysis (PCA) and Pearson correlation analysis (PCA) were used to monitor and evaluate the stability of the system and the reliability of the experimental data.The smaller the CV value, the higher the aggregation of samples within the group in PCA; the closer the correlation coefficient is to 1, the more stable the experimental system is. The Pearson coefficients of protein abundance of the QC samples in the three technical replicates were 0.963, 0.966, and 0.974, respectively. sFig. 1D reflects the degree of aggregation of QC samples, indicating that the system was stable during the collection of this experiment.


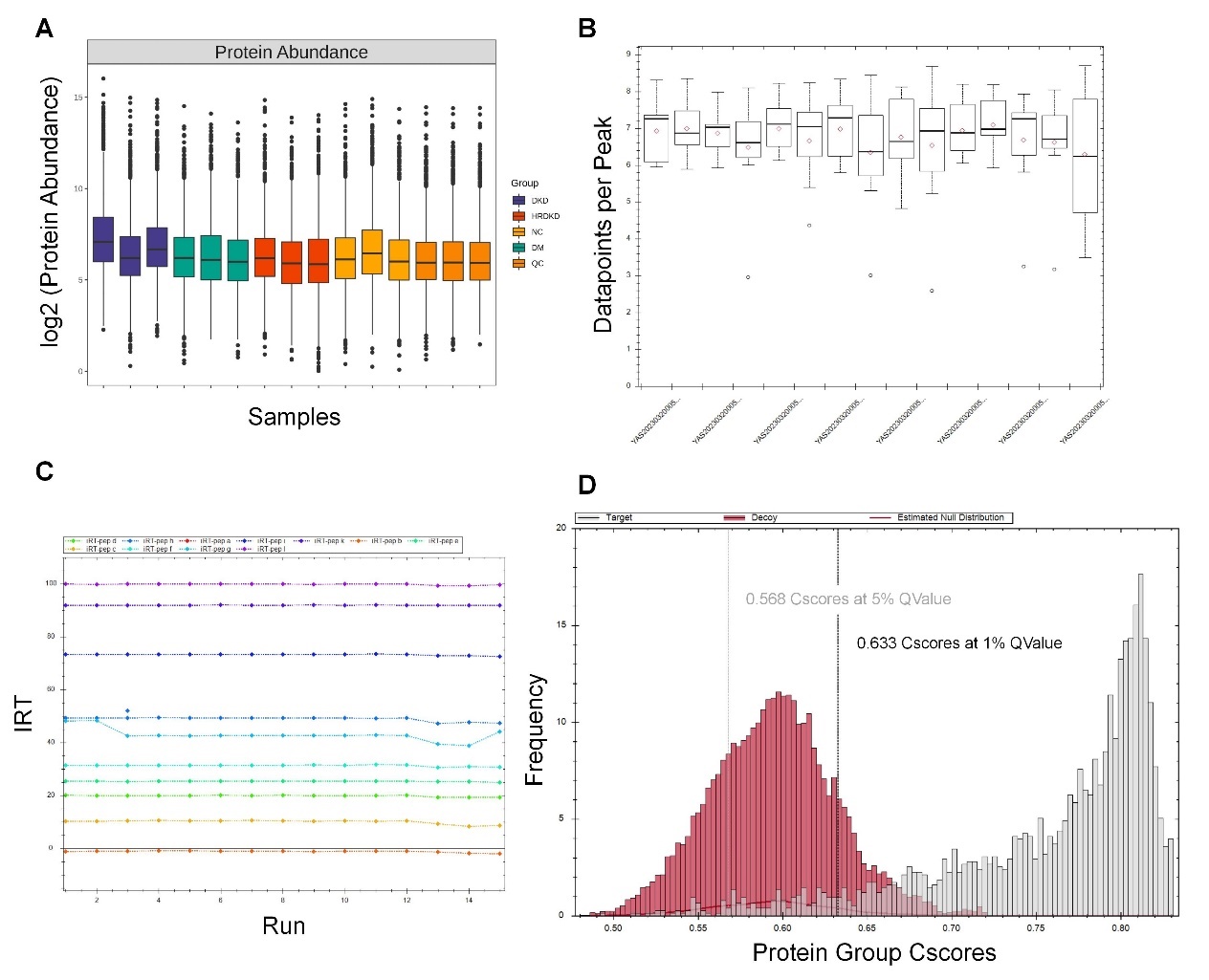


#### Supplementary Figure 2. Intergroup sample identification and evaluation of the DIA system

1. Distribution of sample quantitative intensity jitter; B. Distribution of chromatographic peak mean strongholds; C. Plot of elution times of iRT peptides (corrected peptides); D. Distribution of Protein FDRs.

The number of samples identified between different groups was compared to assess potential differences. The distribution of quantitative values for different samples and proteins was visualized using a sample quantification jitter diagram. The results indicated no outliers in the observations. The average number of chromatographic peaks in the DIA data met the quantitative analysis requirements (≥5 data points per peak). The chromatographic behavior of iRT peptides in individual sample analysis demonstrated overall stability, with major iRTs detected. The Q Value in this experiment had a Cscore of 0.633 at a significance level of 0.01, suggesting highly reliable qualitative results.


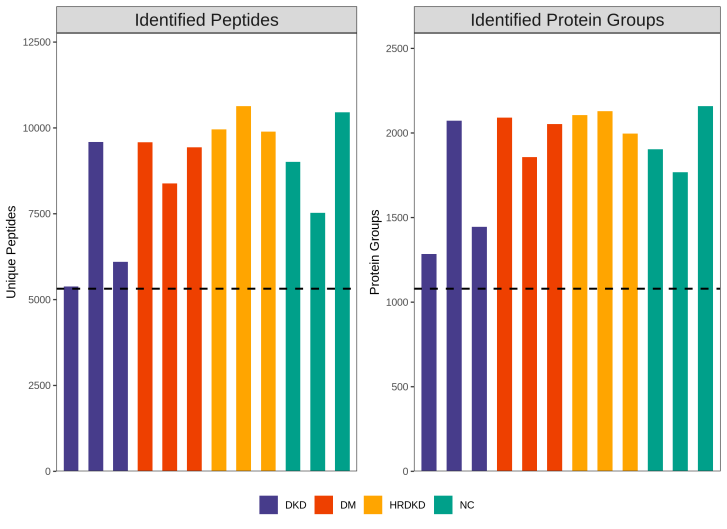


#### Supplementary Figure 3**. Histogram of DIA identification and quantification results**

A total of 2544 proteins and 14,244 peptides were identified in the experiment. The statistics detailing the number of peptides and proteins identified in each sample are presented in sFigs. 3. Each group had over 1000 proteins and 5000 peptides identified, indicating a comprehensive acquisition of proteomic information. Overall, the results of the 4D-DIA proteomics qualitative and quantitative assay demonstrate reliability, high stability, quantification depth, and confidence, supporting further comprehensive analysis.

### Section 2.2. Differential Protein Analysis between Subgroups


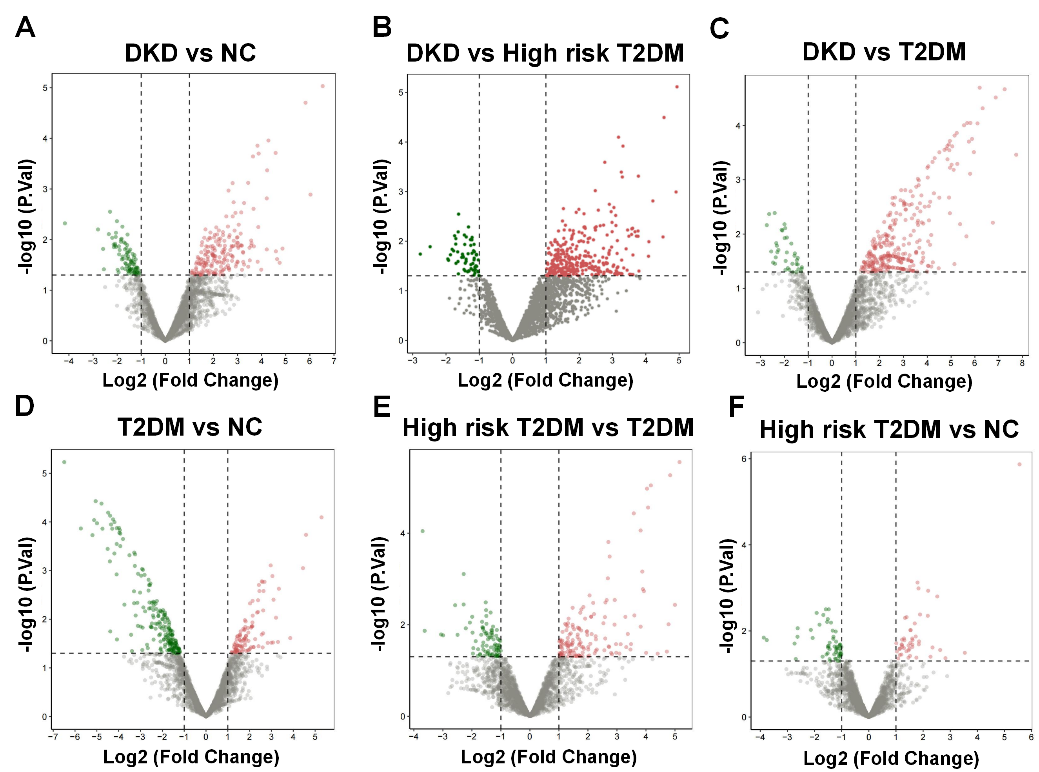


#### Supplementary Figure 4**. Volcano plots**

1. DKD vs HC group; B. DKD vs HR-DKD group; C. DKD vs T2DM group; D. DKD vs HC group; E. DKD vs T2DM group; F. HR-DKD vs HC group.

**Abbreviations:** DKD: diabetic kidney disease; T2DM: type 2 diabetes mellitus; HR-DKD: high-risk diabetic kidney disease; HC: healthy control;

The differential proteins were identified using a significance threshold of P 0.5. The proteins were then grouped for pairwise comparison and visualized using volcano plots.
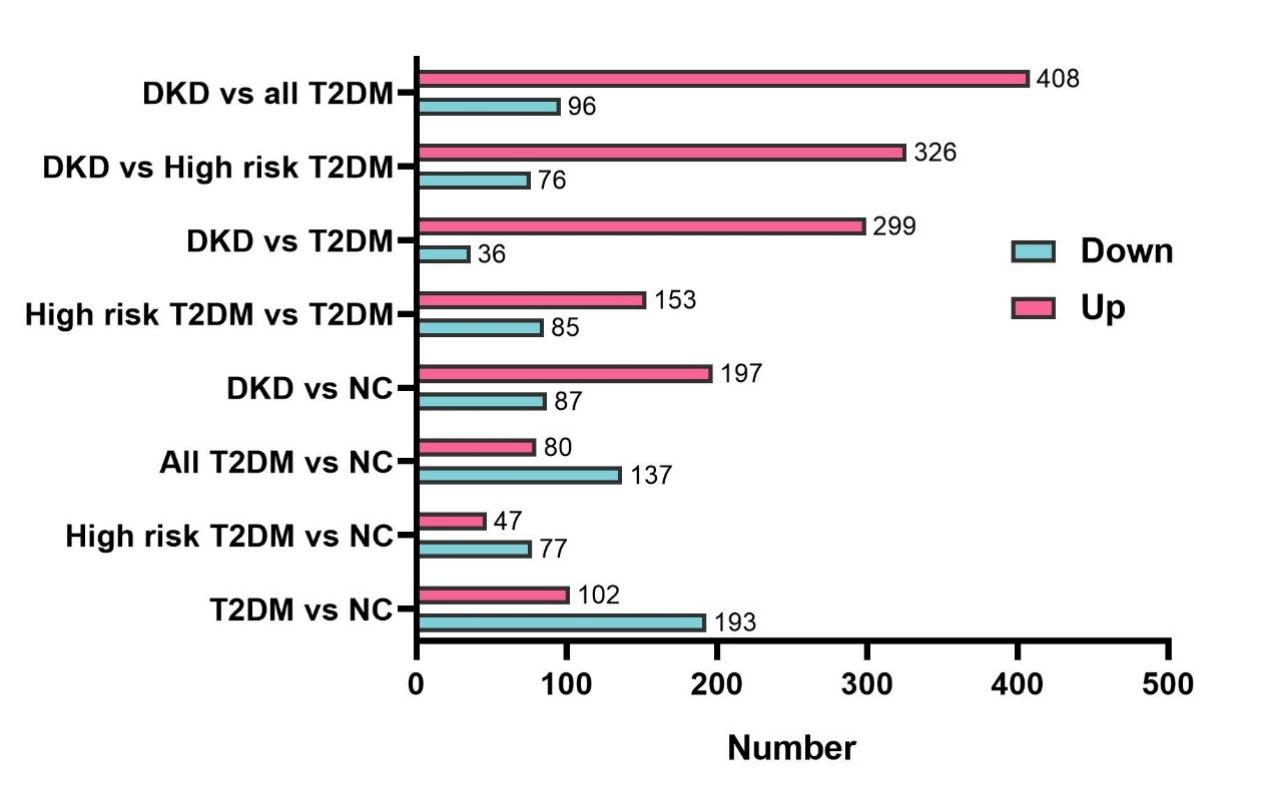


#### Supplementary Figure 5**. Statistics on the number of up-regulated and down-regulated differential proteins compared between different subgroups**

Bar charts were utilized to represent the number of differential proteins, providing a clearer comparison of differential expression patterns across different groups. Distinct proteomic differential expression landscapes were observed between the early and late stages of the disease. In the early stages, a majority of proteins were down-regulated, whereas in the late stages, a majority of proteins were up-regulated.

### Section 2.3. Overlap in Differential Protein Identification between Groups


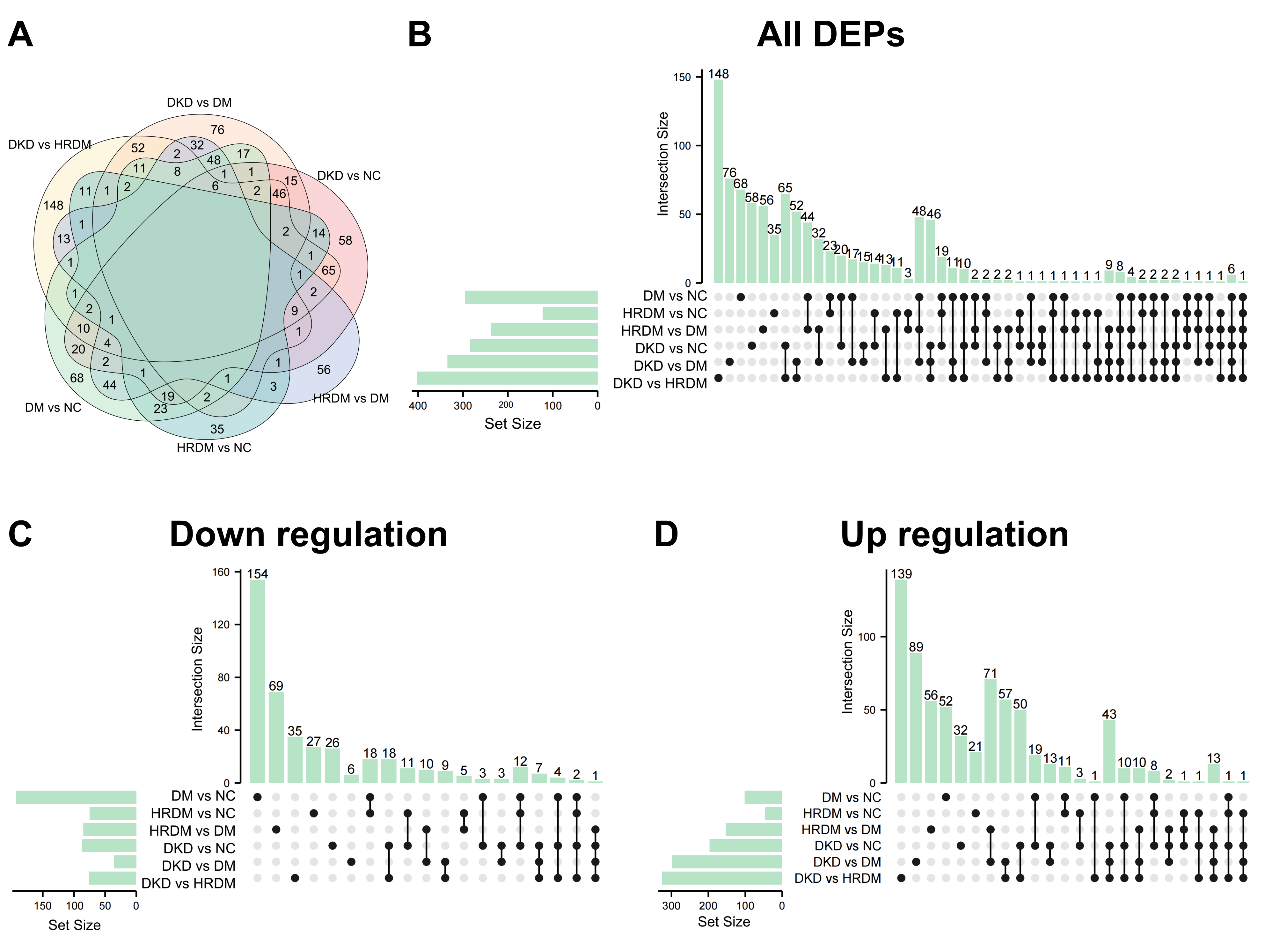


#### Supplementary Figure 6**. Overlap of differential protein identification between different groups and Upset plots**

1. VENN petal plot; B. Overlap of all differential proteins; C. Overlap of down-regulated proteins; D. Overlap of up-regulated proteins.

The study identified 20 shared differential proteins among the three groups of DM vs NC, HRDKD vs DM, and DKD vs HRDKD, as well as 65 shared differential metabolites in DKD compared to the other groups. Additionally, a total of 90 up-regulated and 26 down-regulated proteins were found across more than 3 groups. The expression of these shared differential proteins may play a significant role in the progression and development of DKD.

### Section 2.4. Heat Map and Functional Analysis of Differential Proteins

**T2DM vs. HC group**


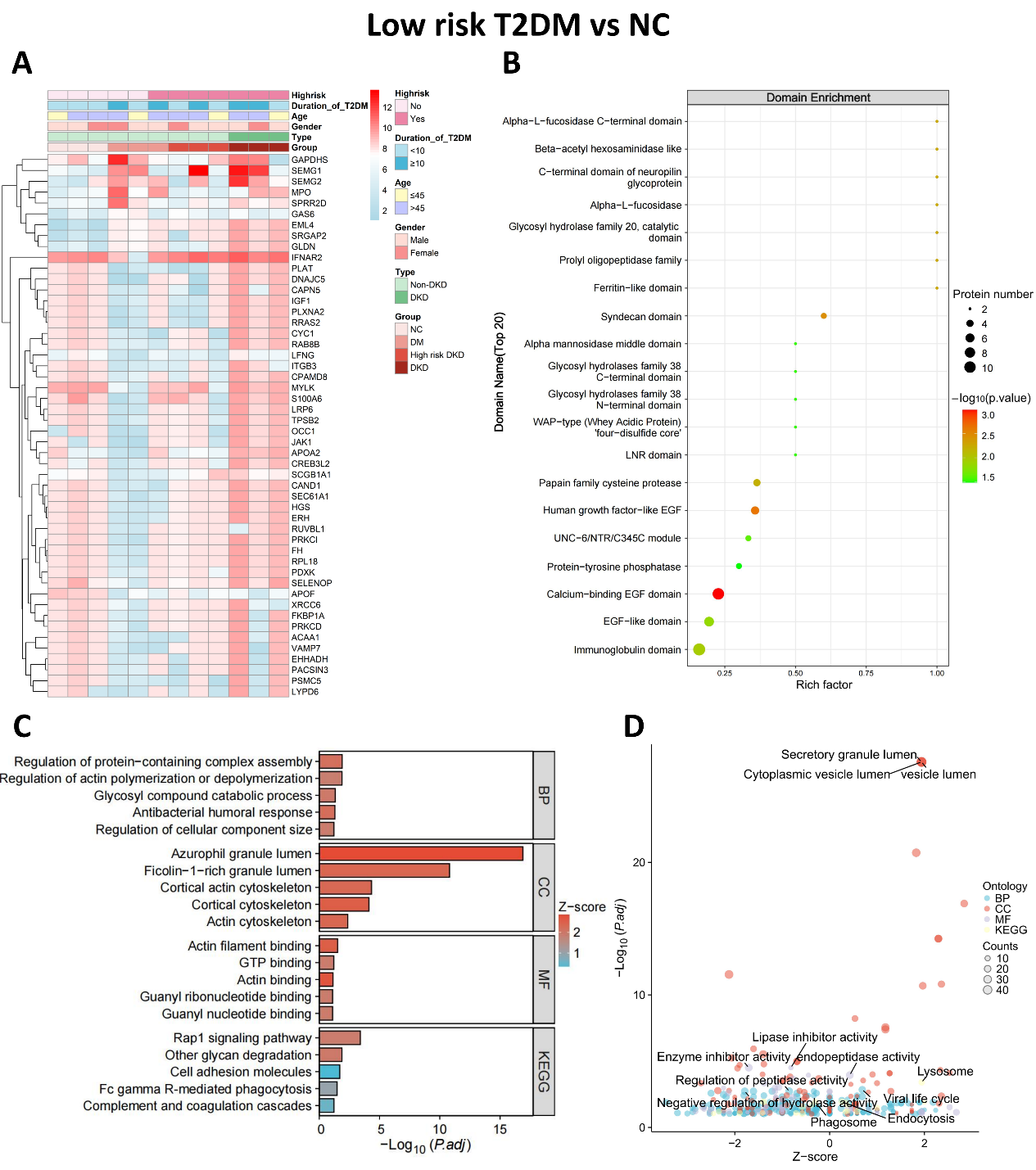


#### Supplementary Figure 7**. Top 50 differential proteins display and functional analysis**

1. Heat map of Top 50 DEPs; B. Top 20 results of differential protein structural domain analysis; C. Bar chart of KEGG/GO analysis; D. Bubble chart of KEGG/GO analysis Note: BP: biological process; MF : molecular function (molecular function); CC: cellular component (cellular component)

To specifically illustrate the expression profile of DEPs under different clinical characteristics between groups, we selected the top 50 DEPs based on |log2FC| sorting. Heatmaps were utilized to depict the expression pattern of these Top50 DEPs in DKD subgroups (yes/no), high/low risk subgroups, duration of type 2 diabetes mellitus (≥10 or <10 years), age (≥45 or <45 years), and gender (male or female). Figures 1-10 showcase the Top50 DEPs in the diabetes group compared to healthy controls, along with KEGG/GO functional enrichment analysis and protein structural domain analysis. Notably, SEMG1 and SEMG2 proteins exhibited significant elevation in the diabetes group, with structural domain enrichment primarily focusing on calcium-binding EGF, immunoglobulin, and humanoid EGF domains. Functional analysis revealed differences between the two proteins mainly in the azurophilic granule lumen, Rap1 signalling pathway, and complement and coagulation cascade pathway.

**HRDKD vs. T2DM group**


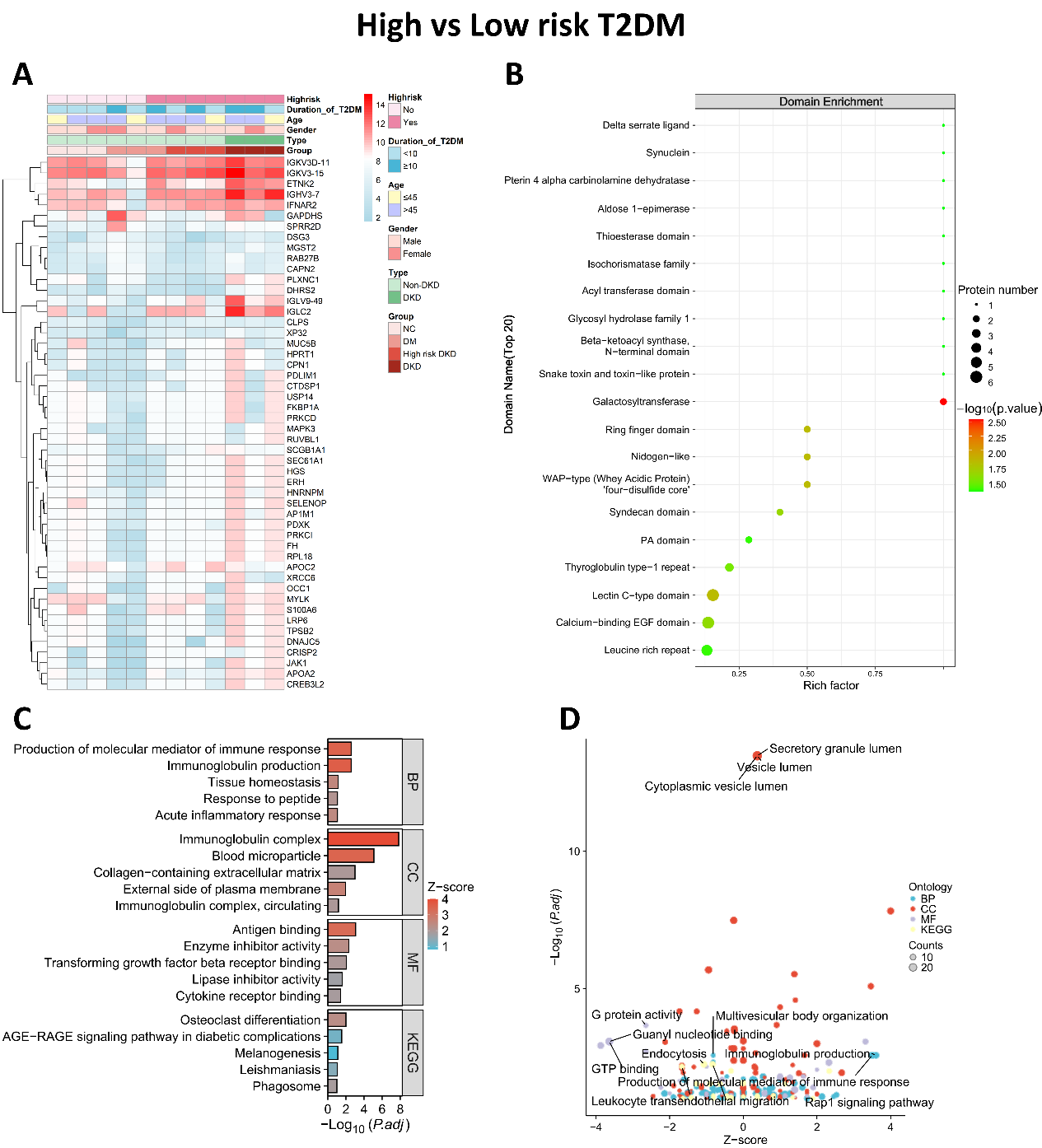


#### Supplementary Figure 8**. Top 50 differential proteins display and functional analysis**

A. Heatmap of Top 50 DEPs; B. Top20 Results of Differential Protein Structural Domain Analyses; C. KEGG/GO Analysis Bar Chart; D. KEGG/GO Analysis Bubble Chart.

BP: biological process; MF:molecular function; C: cellular component.

Proteins such as ETNK2 and IGLC2 were found to be significantly upregulated in the high-risk group for diabetic nephropathy compared to the diabetic group. Analysis of KEGG/GO functional enrichment and protein structural domains revealed that pathways with higher structural domain enrichment included lectin C-type structural domains, calcium-binding EGF structural domains, and leucine-rich repetitive sequences. Additionally, GO terms were predominantly associated with immunoglobulin complexes, blood microparticles, and antigen binding.

**DKD group versus HR-DKD group**


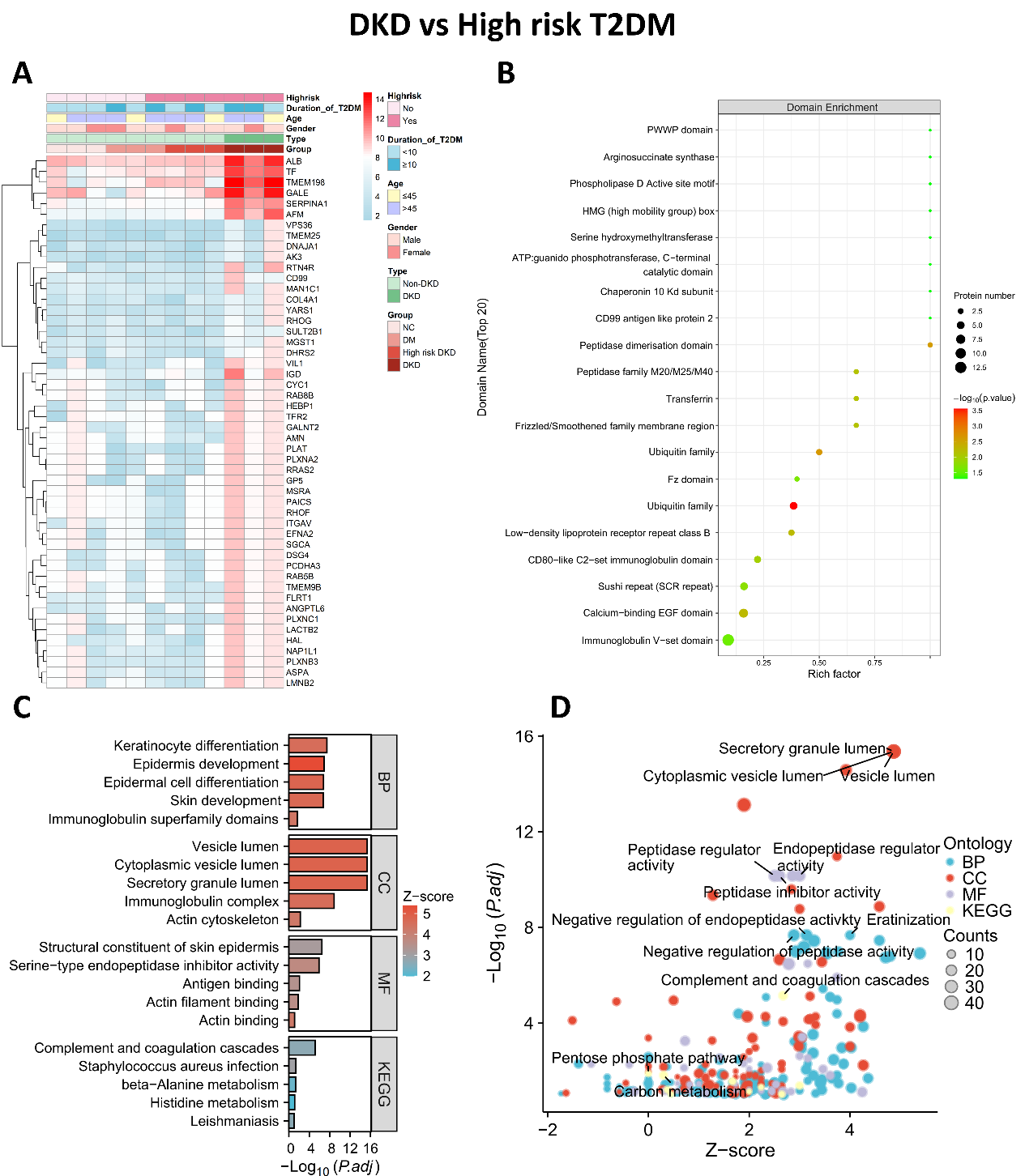


#### Supplementary Figure 9**. Top 50 differential proteins display and functional analysis**

A. Heatmap of Top 50 DEPs; B. Top20 Results of Differential Protein Structural Domain Analyses; C. KEGG/GO Analysis Bar Chart; D. KEGG/GO Analysis Bubble Chart. BP: biological process; MF:molecular function; C: cellular component.

The transition from high-risk diabetic status to diabetic kidney disease (DKD) represents a critical stage in disease progression. In comparing the diabetic nephropathy group to the high-risk group, it was observed that most proteins were up-regulated, including SERPINA1, AFM, GALE, TMEM198, and TF. Through KEGG/GO functional enrichment analysis of differential proteins and protein structural domain analysis, it was found that pathways with higher structural domain enrichment were primarily related to the ubiquitin family, calcium-binding EGF structural domains, and immunoglobulin V-set structural domains. Additionally, KEGG terms were associated with the complement and coagulation cascade, while GO terms were linked to keratinocyte differentiation and immunoglobulin superfamily structural domains.

### Section 2.5. **Total Up- and Down-regulated Differential Proteins**


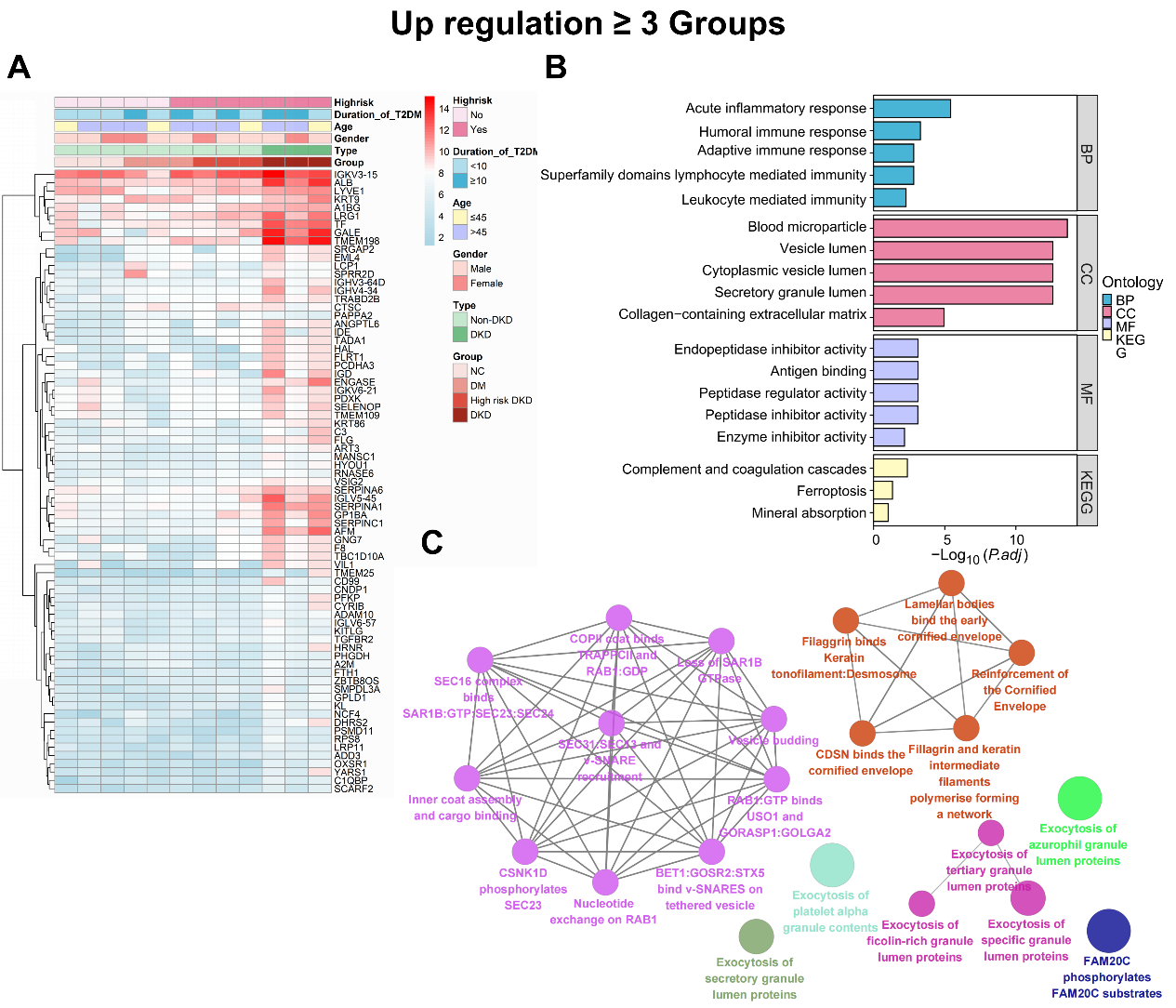


#### Supplementary Figure 10**. Heatmap and functional analysis of shared up-regulated proteins among multiple groups**

1. Heatmap visualization of DEPs; B. KEGG/GO analysis of differential protein structural domains; C. Reactome pathway analysis

A total of 90 up-regulated proteins were identified across multiple groups, including LYVE, TF, TMEM198, AFM, and SERPINA1, showing increased levels with disease progression. Functional enrichment analysis using KEGG/GO and Reactome pathways revealed that GO terms were related to blood particles, acute inflammatory response, and endopeptidase inhibitor activity. KEGG pathways were associated with complement and coagulation cascade, while Reactome pathways included RAB1:GTP binding to USO1 and GORASP1:GOLGA2, SEC16 complex binding to SAR1B:GTP:SEC23:SEC24, exocytosis of platelet alpha-granule contents, and activation of protein fiber bundle formation.


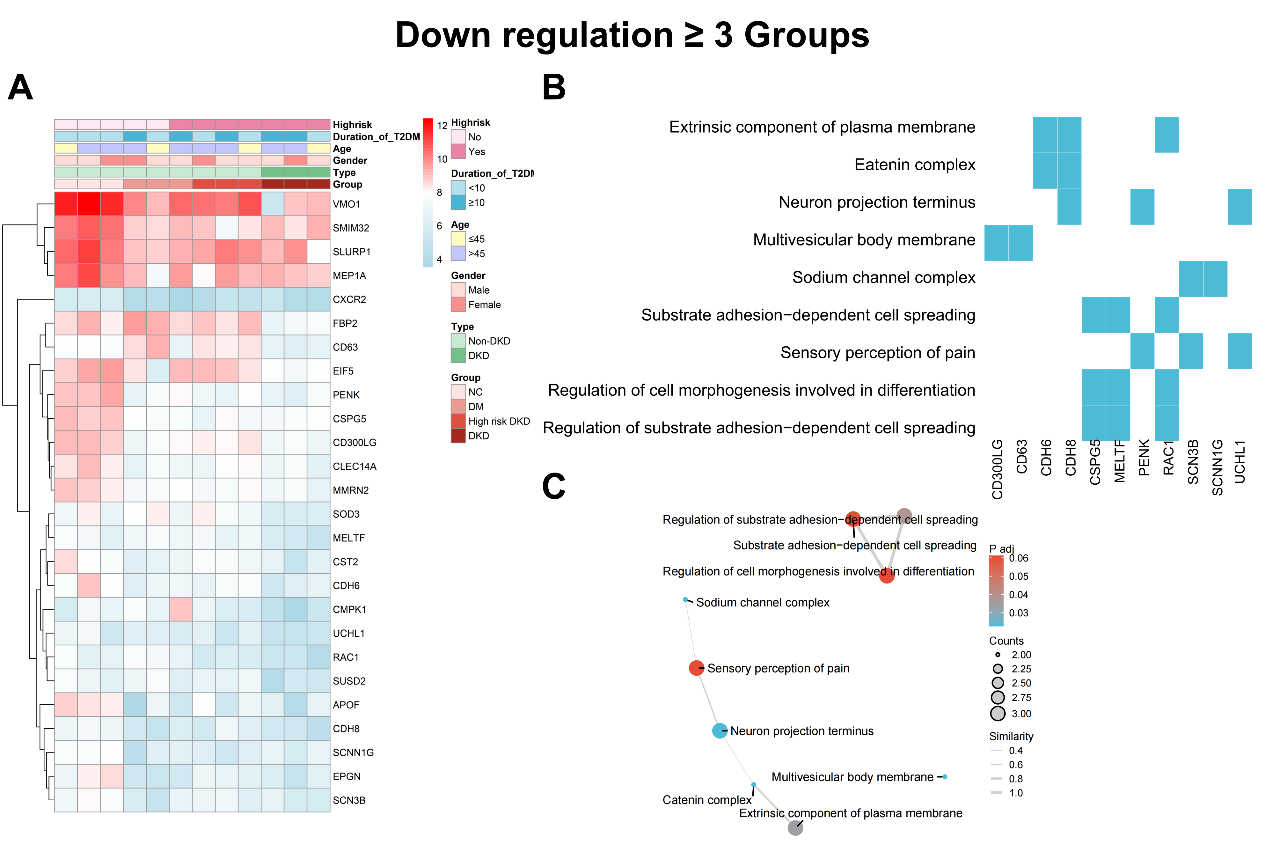


#### Supplementary Figure 11**. Heatmap and functional analysis of shared down-regulated proteins among multiple groups**

A. Heatmap visualization of DEPs; B. GO analysis of differential protein structural domains and corresponding genes; C. EMAP analysis. EMAP analysis indicates pairwise similarity between Terms by line thickness.

A total of 26 proteins were down-regulated across multiple groups, with VMO1, SMIM32, SLURP1, MEP1A, and FBP2 showing a decline as the disease progressed. The results of the GO function enrichment analysis indicated that the GO term primarily involved the regulation of matrix adhesion-dependent cell spreading and the regulation of cell morphogenesis involved in differentiation. However, the KEGG pathway could not be enriched due to the limited number of molecules.

### Section 2.6. **Mfuzz Temporal Expression Clustering Analysis and Functional Clustering**


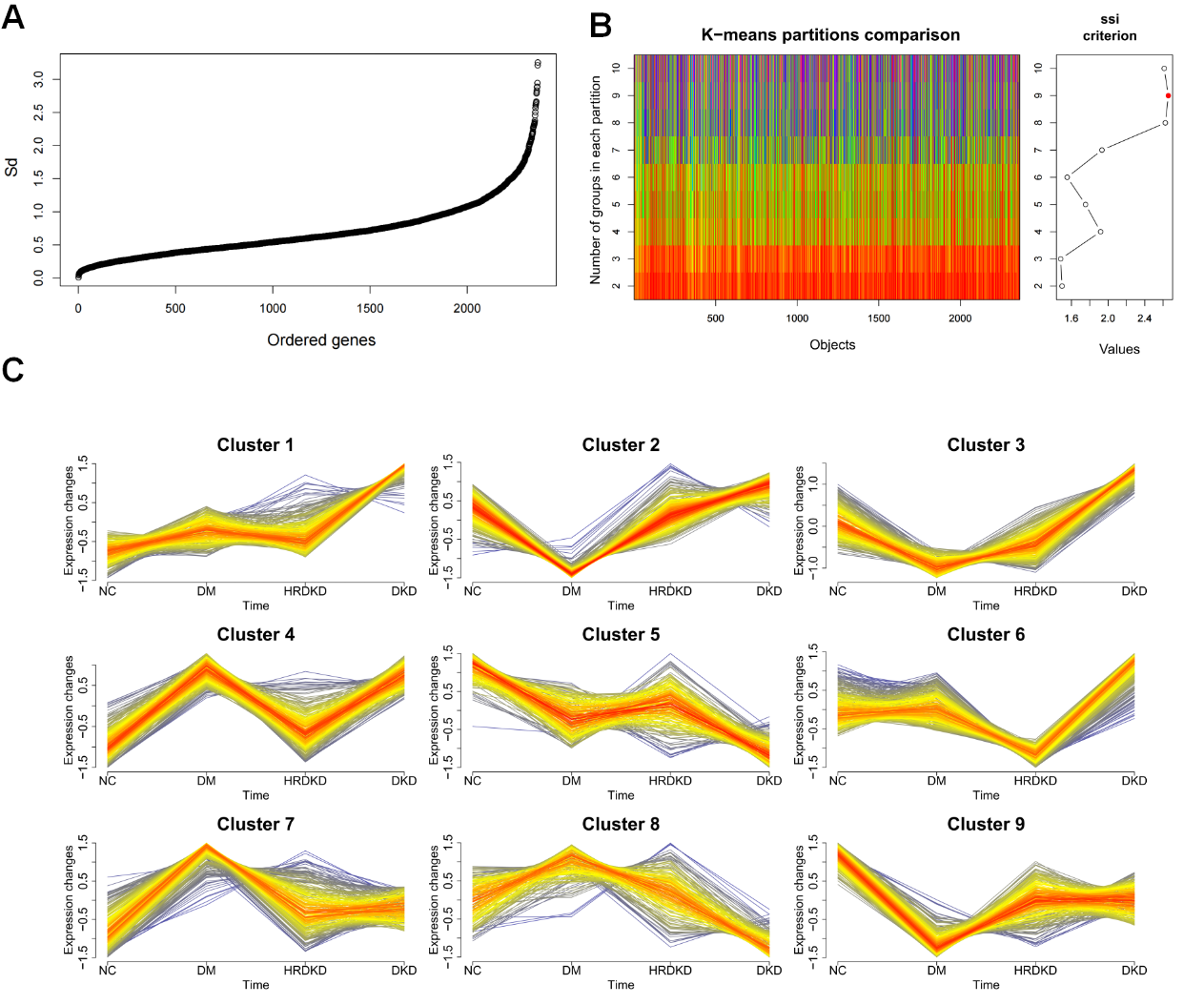


#### Supplementary Figure 12**.** Mfuzz protein time series expression trend analysis

1. Filtering of proteins by standard deviation method; B. Evaluation of the optimal number of clusters by NbClust method; C. Clustering of protein expression time series based on fuzzy c-means algorithm (9 clusters).

The study examined the temporal dynamics of protein expression profiles using Mfuzz to cluster proteins with similar patterns. After filtering the proteins, 9 clusters were identified as optimal using the NbClust method. These clusters displayed distinct kinetic patterns, with significant differences observed between them. For example, Cluster 1 showed a positive correlation with the disease process, whereas Cluster 5 exhibited a negative correlation.


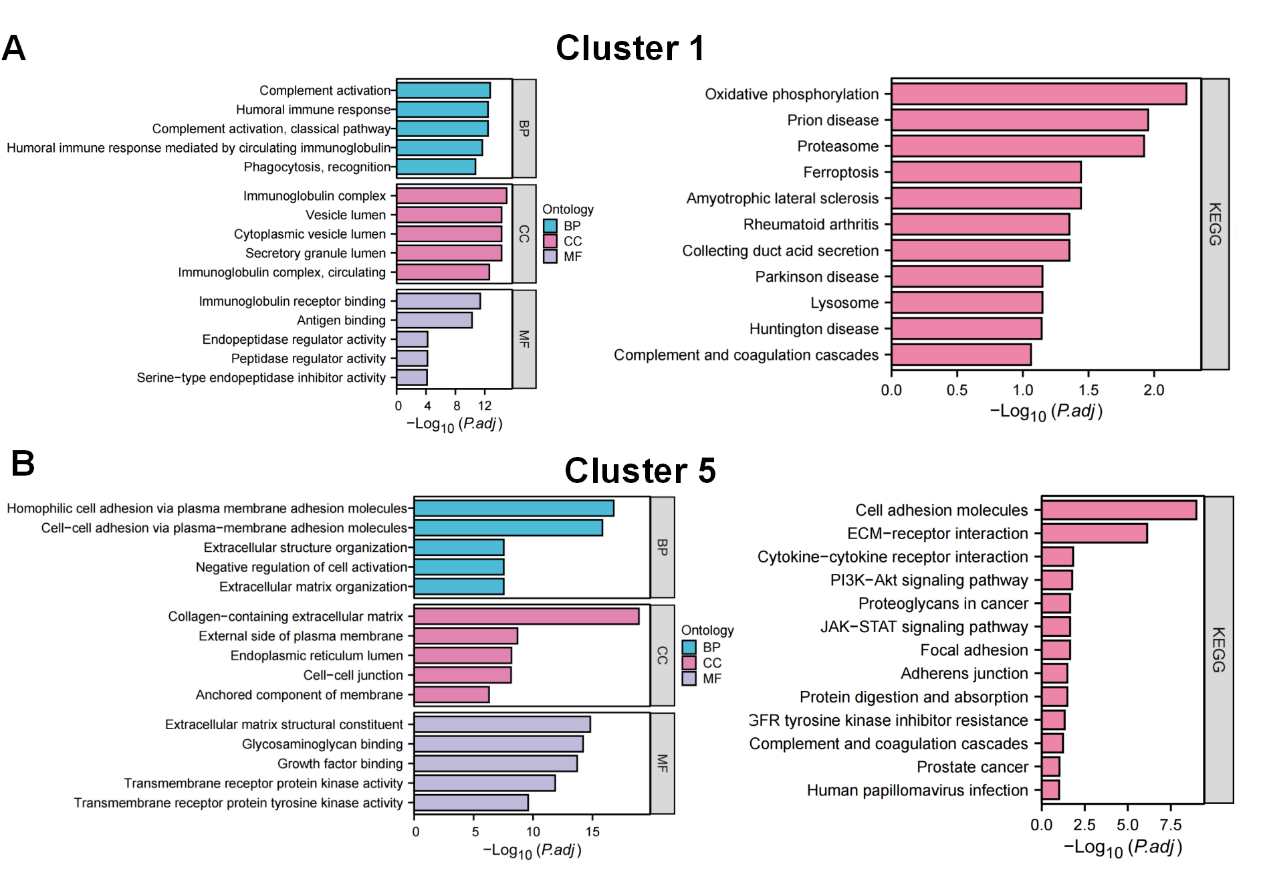


#### Supplementary Figure 13**.** KEGG and GO analysis of Cluster 1 and 5

1. KEGG and GO analysis of Cluster 1; B. KEGG and GO analysis of Cluster 5

Further examination of the protein expression matrices revealed that molecular cluster 1 displayed functional changes enriched in the classical pathway of complement activation, humoral immune response, phagocytosis, and recognition process. Additionally, this cluster showed enrichment in oxidative phosphorylation, iron death, complement activation, and coagulation cascade response pathways. On the other hand, altered molecular functions in cluster 5 were mainly associated with adhesion molecules and structural components of the extracellular matrix, while the pathways were enriched in cell adhesion molecules, ECM-receptor interactions, and cytokines and their receptor interactions.

### Section 2.7. Statistical Analysis of Molecular Characterization and Organ Origin of Differential Proteins


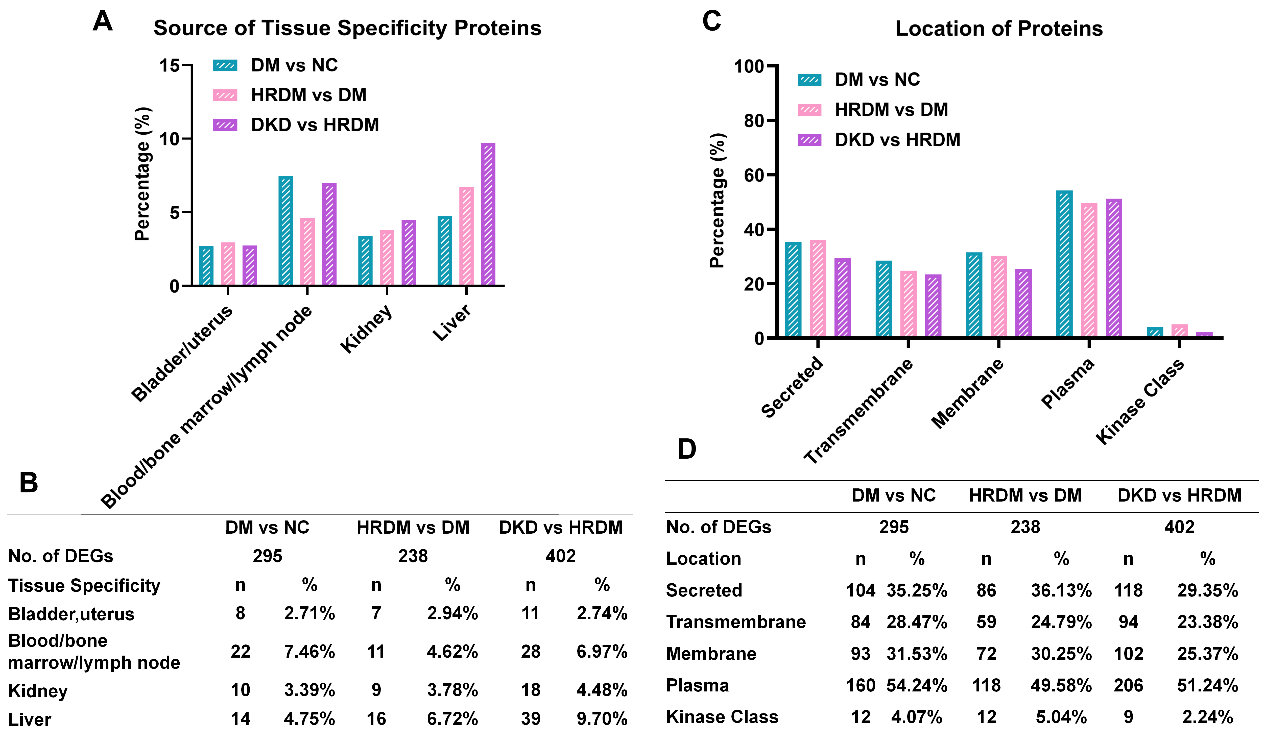


#### Supplementary Figure 14**.** Tissue and organ sources and classification of differential proteins in different stages

1. Tissue-specific sources of differential proteins; B. Different intracellular distributions of differential proteins and kinase species statistics

Organ and intercellular communication, along with the mediators involved, may play a role in driving the development of Diabetic Kidney Disease (DKD). Proteins are key components in organ and intercellular communication, acting as signaling molecules. This study aimed to identify the various molecules originating from different organs and subcellular locations to understand the roles of inter-organ communication in different stages of DKD. The analysis reveals an increase in the number and percentage of proteins originating from the kidney and liver as the disease progresses. Notably, there is a significant rise in liver-derived proteins during critical stages of DKD pathogenesis.


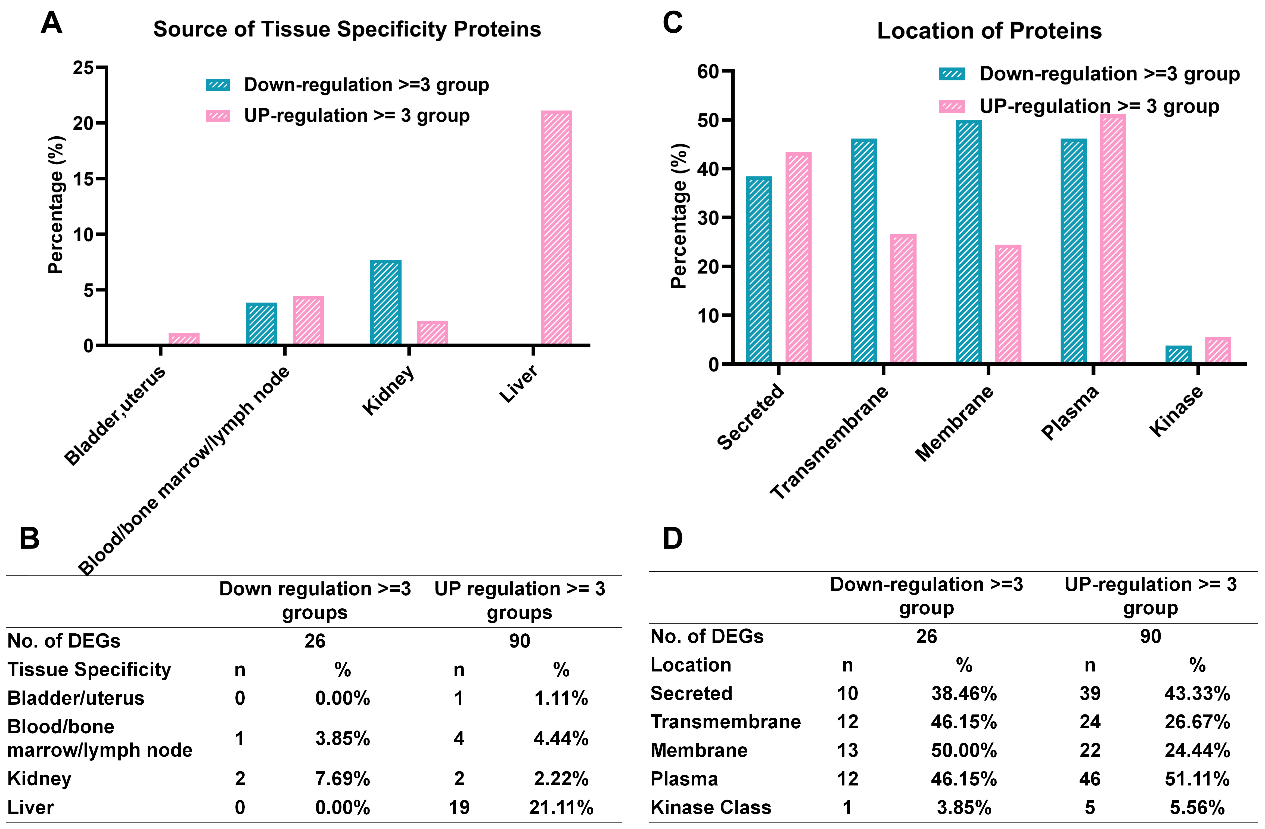


#### Supplementary Figure 15. Tissue and organ sources and classification of 3 or more groups of shared up-regulated and down-regulated differential proteins

1. Tissue-specific sources of differential proteins; B. Different intracellular localization of differential proteins and kinase species statistics

The liver exhibited the highest number of up-regulated differential proteins (19, 21.1%), indicating the significant involvement of the liver-kidney interaction mechanism in DKD. Notable proteins identified in this category include SELENOP, DHRS2, AZGP1, and KNG1. There was no significant variance observed in the intra- and extracellular distribution and kinase source across different disease stages. However, among the differential proteins shared by more than 3 groups, there was a prevalent up-regulation of secretory and kinase-type proteins in expression.


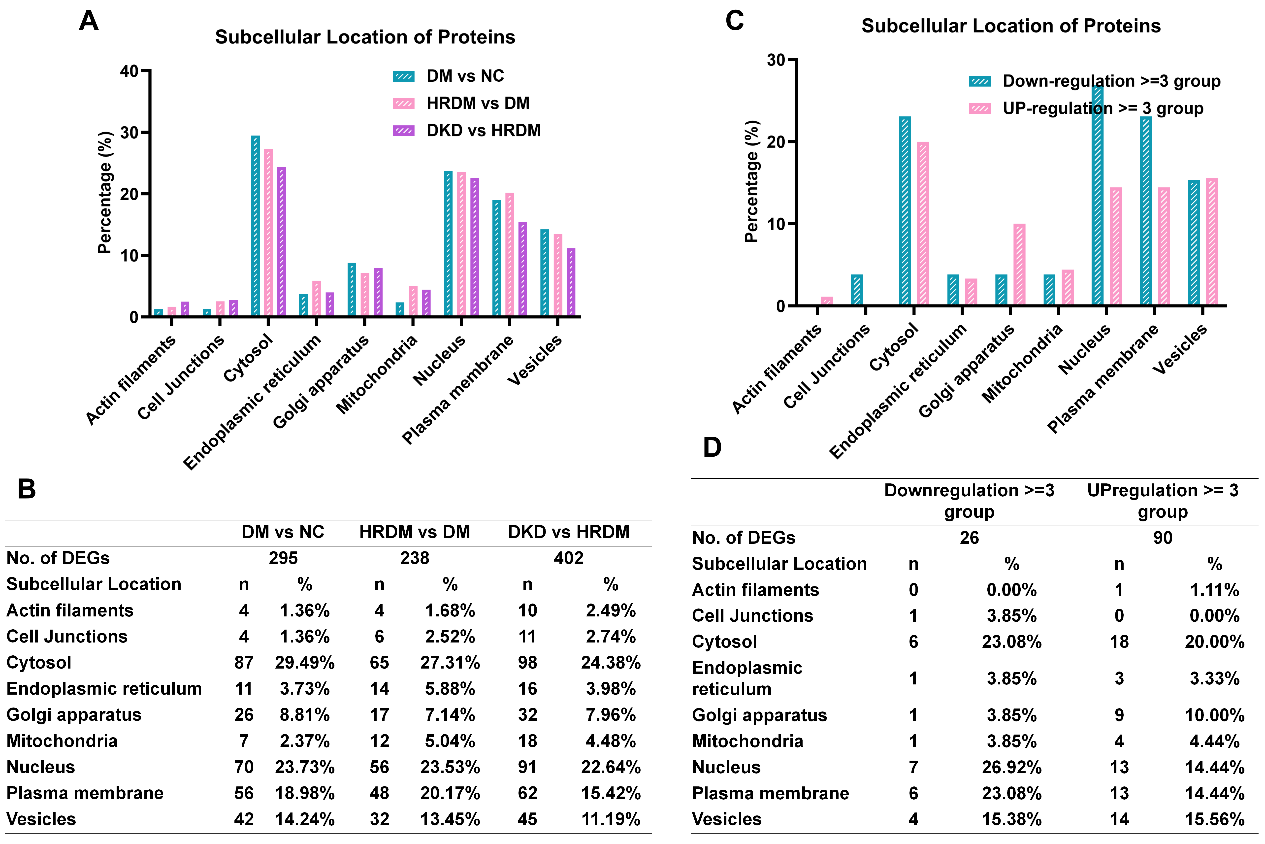


#### Supplementary Figure 16. Subcellular localization of differential proteins at different stages

1. Sources of subcellular structural specificity of differential proteins at different disease stages; B. Sources of subcellular structural specificity of 3 or more groups of shared up- and down-regulated differential proteins

The study reveals the subcellular localization of various proteins at different disease stages. Actin filaments, cell junctions, and mitochondria-derived proteins exhibited an increasing trend with disease progression, while Golgi volume, actin filaments, and mitochondria-derived proteins also displayed a continuous upward trend. Statistical analysis identified several mitochondrial-derived proteins such as PLXNC1, DHRS2, GPX4, CYC1, HRNR, SMPDL3A, EFNA2, GLUD1, and C1QBP.

### Section 2.8. Weighted Gene Co-expression Network Analysis


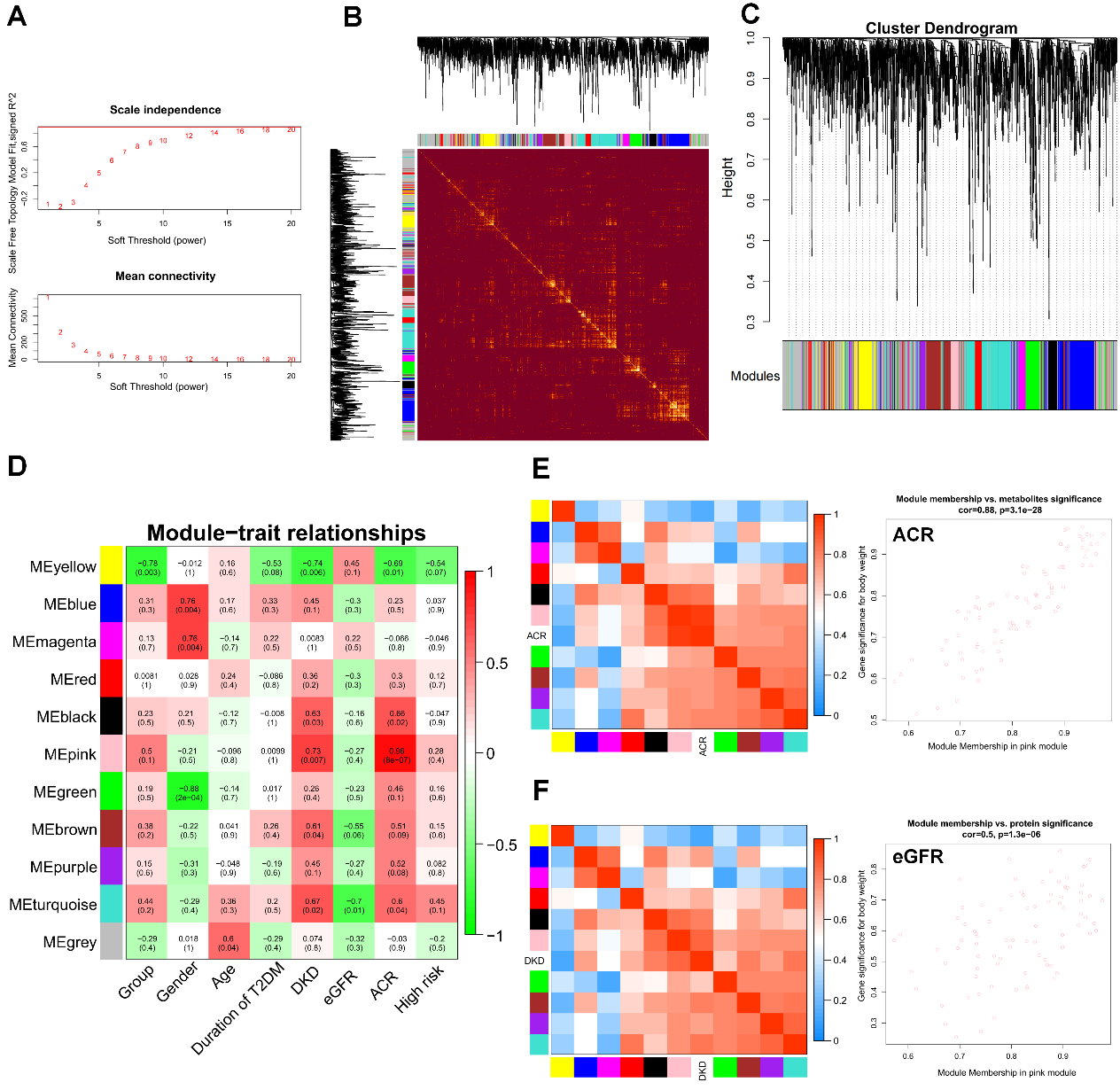


#### Supplementary Figure 17. Weighted gene co-expression network analysis

1. Power diagram to screen for appropriate thresholds; B. Topological overlap matrix to construct co-expression network; C. Clustering dendrogram; D. Heatmap of module feature correlation; E. ACR vs. pink module association diagram; F. eGFR vs. pink module association diagram

Genes with similar expression patterns may be co-regulated and functionally related in the same pathway. Therefore, in this study, we investigated gene co-expression in Diabetic Kidney Disease (DKD) using weighted gene co-expression network analysis (WGCNA). We clustered genes to identify different modules and pinpoint the core genes within these modules. The WGCNA tool was utilized to explore co-expression patterns in DKD. The soft threshold was calculated using the pick soft threshold function, with a threshold of 18 selected for this analysis. The neighbor-joining matrix was transformed into a topological overlap matrix to reduce noise, and a TOM diagram was generated. The clustering dendrogram, based on the distance matrix derived from the TOM matrix calculation, categorized 2363 genes into 13 modules. Through module-phenotype feature association mapping, the pink module was found to be closely linked to DKD and Albumin-to-Creatinine Ratio (ACR) phenotypes (P = 0.007 & 8e-7). Further analysis revealed that the pink module included genes such as LDHA, ALDOA, PKM, PRDX5, and ACTB. Scatter plots of Module Membership (MM) versus Gene Significance (GS) indicated that higher MM values in the pink module corresponded to greater protein significance.

### Section 2.9. Enrichment Analysis of Key Transcription Factors and Hub gene Screening

**T2DM and HC group**


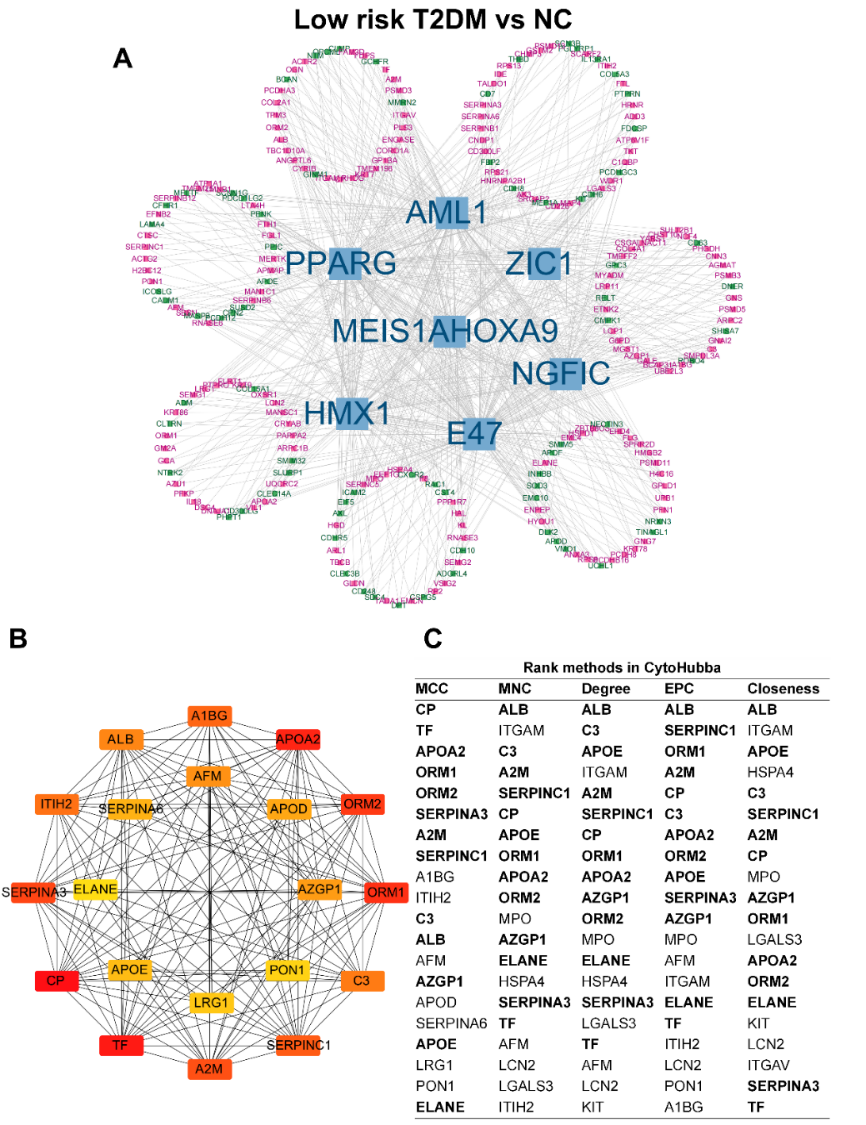


#### Supplementary Figure 18. Screening of transcription factors and hub genes

A. Core transcription factor regulatory network map; B. Protein interaction network analysis (PPI); C. hub genes obtained based on 5 algorithms

A. Blue squares are TFs, green squares are down-regulated genes, and red squares are up-regulated genes; B. Maximum group centrality (MCC) algorithm, the darker the color of the squares, the higher the score and the higher the significance; C. Graphs demonstrating the results of 5 algorithms, including maximum group centrality (MCC), maximum neighborhood component (MNC), Degree algorithm, edge penetration component algorithm (EPC), and Closeness algorithm, and the bolded font shows the genes obtained by taking the intersection of the results of these 5 algorithms, with the scores decreasing from top to bottom.

The transcription factors regulating the differentially expressed proteins can be identified through enrichment analysis, while the key hub genes can be identified through protein interaction network analysis. The enriched transcription factors include AML1, ZIC1, PPARG, NGFIC, MEISIAHOXA9, HMX1, and E47. The core genes identified in the protein-protein interaction (PPI) analysis were determined by calculating scores using five algorithms, selecting the top 20, and identifying the intersection to obtain the Hub genes. These Hub genes comprise CP, TF, APOA2, ORM1, ORM2, SERPINA3, SERPINAC1, C3, ALB, AZGP1, APOE, and ELANE.

**HRDKD group vs. T2DM group**


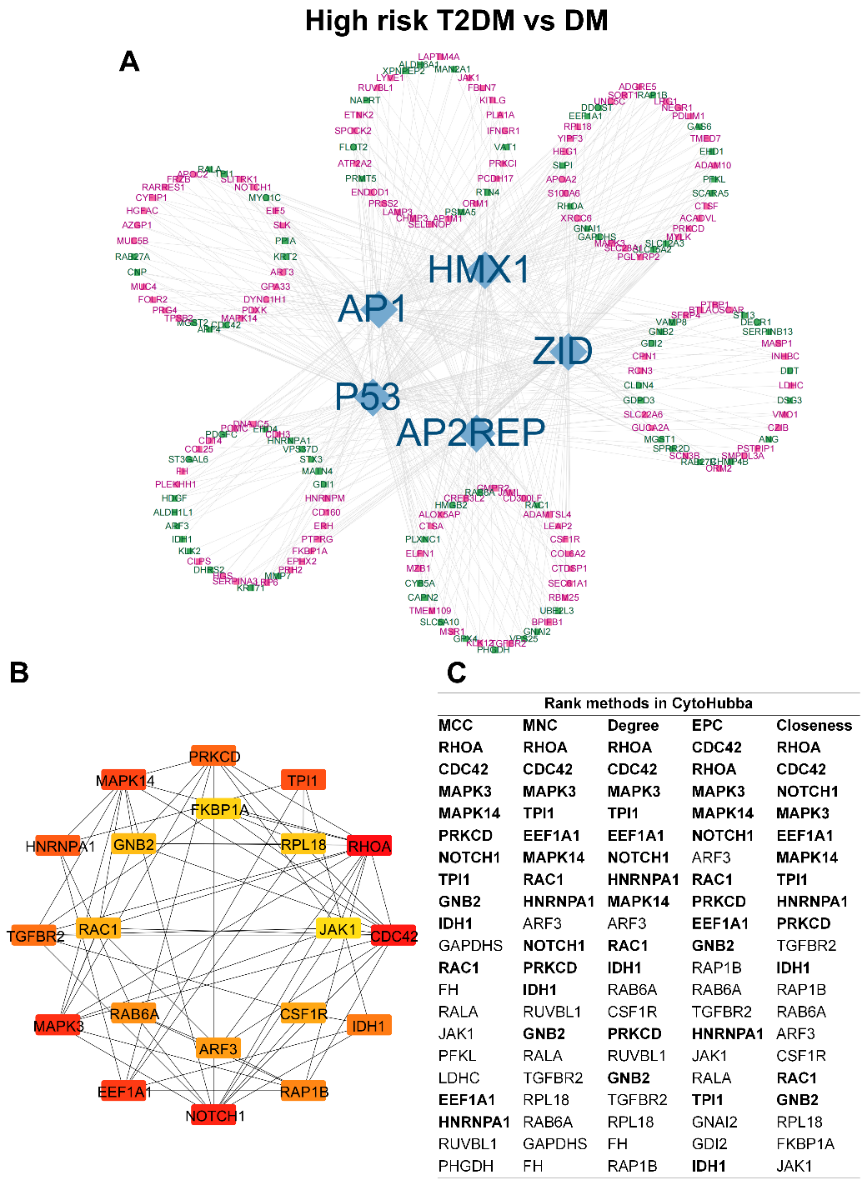


#### Supplementary Figure 19. Screening of transcription factors and hub genes

1. Core transcription factor regulatory network map; B. Protein interaction network analysis (PPI); C. hub genes obtained based on 5 algorithms

The study demonstrates the transcription factors enriched in diabetic kidney disease (DKD), including AP1, HMX1, ZID, P53, and AP2REP. The scores of different genes were calculated using 5 algorithms, and the top 20 genes were selected. The intersection of these genes resulted in the identification of Hub genes, which included RHOA, CDC42, MAPK3, MAPK14, PRKCD, NOTCH1, TPI1, GNB2, IDH1, RAC1, EEF1A1, and HNRNPA1, suggesting their potential role in the progression of DKD.

**DKD and HRDKD group**


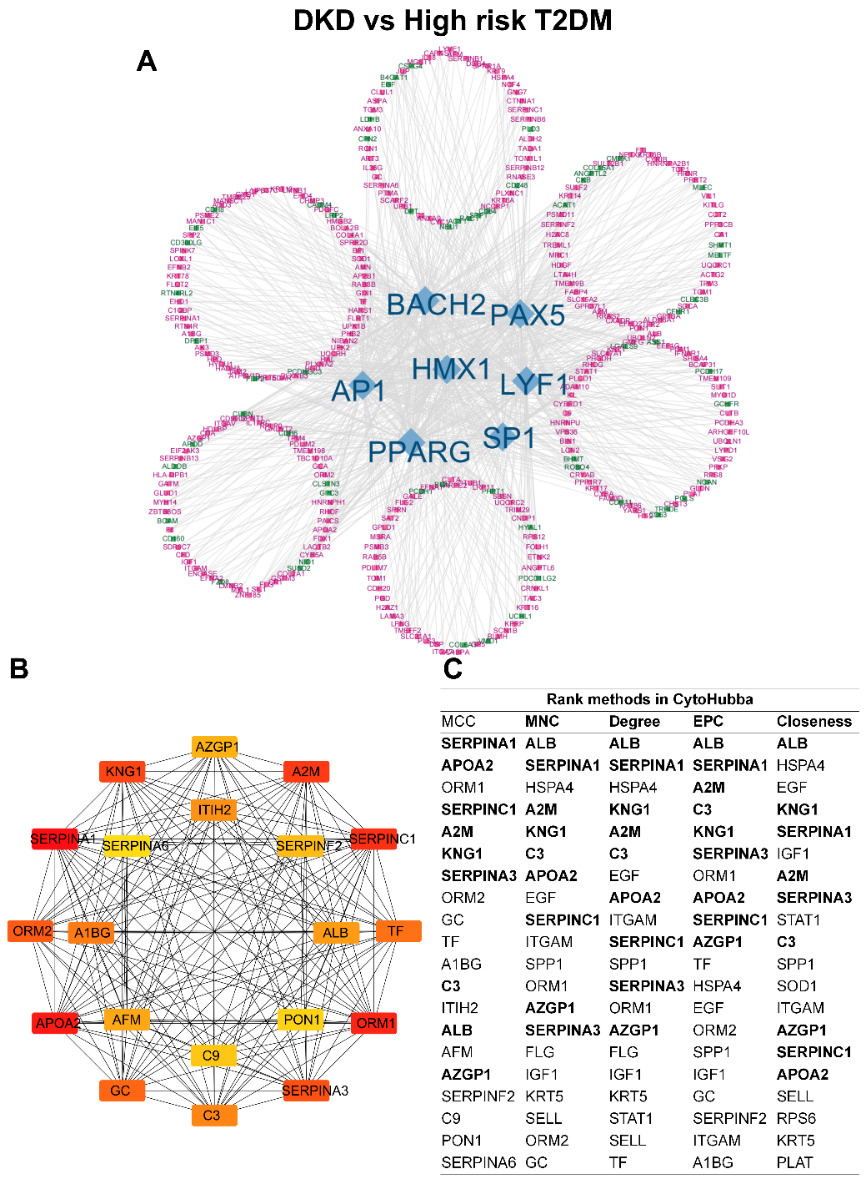


#### Supplementary Figure 20. **Screening of transcription factors and hub genes**

1. Regulatory network map of core transcription factors; B. Protein interaction network analysis (PPI); C. hub genes obtained based on five algorithms

The study showcases a variety of enriched transcription factors, such as AP1, PAX5, HMX1, LYF1, BACH2, SP1, and PPARG, which may play a role in the progression of DKD. Additionally, the PPI analysis reveals the core genes, with scores calculated using five algorithms to identify the top 20 genes. The intersection of these genes results in the identification of Hub genes, which include SERPINA1, APOA2, SERPINC1, A2M, KNG1, SERPINA3, C3, ALB, and AZGP1.

**Shared up- and down-regulated differential proteins among multiple groups**


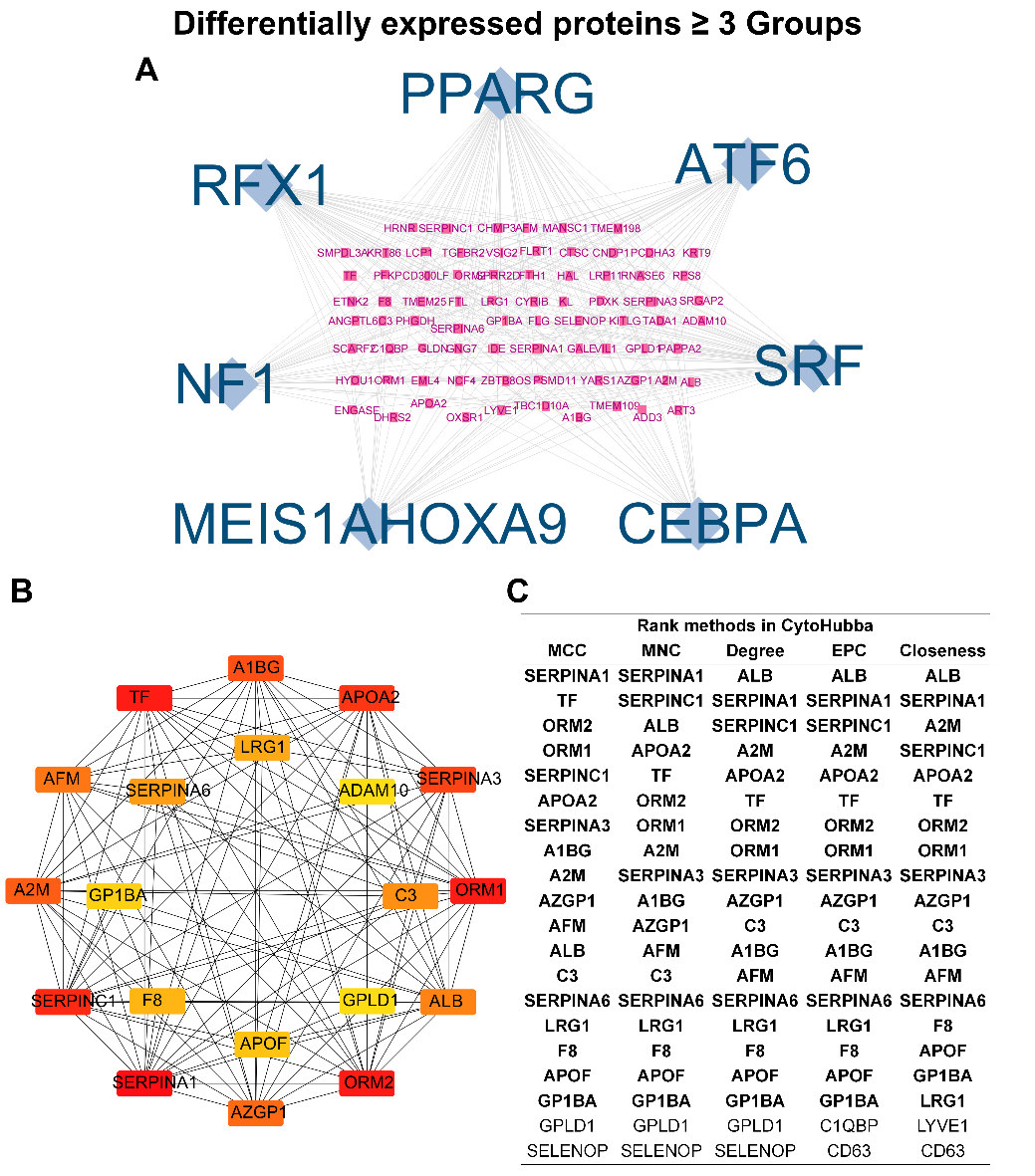


#### Supplementary Figure 21. Screening of transcription factors and hub genes

1. Regulatory network map of core transcription factors; B. Protein interaction network analysis (PPI); C. hub genes obtained based on 5 algorithms.

The enriched transcription factors in this study include PPARG, ATF6, RFX1, NF1, SRF, MEIS1AHOXA9, and CEBPA. These transcription factors may play a role in the progression of Diabetic Kidney Disease (DKD). The core genes identified in the Protein-Protein Interaction (PPI) analysis were scored using five algorithms, with the top 20 genes selected and their intersection revealing the Hub genes. The Hub genes consist of SERPINA1, TF, ORM1, ORM2, SERPINC1, APOA2, SERPINA3, A1BG, A2M, AZGP1, AFM, ALB, C3, SERPINA6, LRG1, APOF, and GP1BA.

### Section 2.10. Evaluation and Pre-screening of Proteomics Biomarkers from Multiple Perspectives


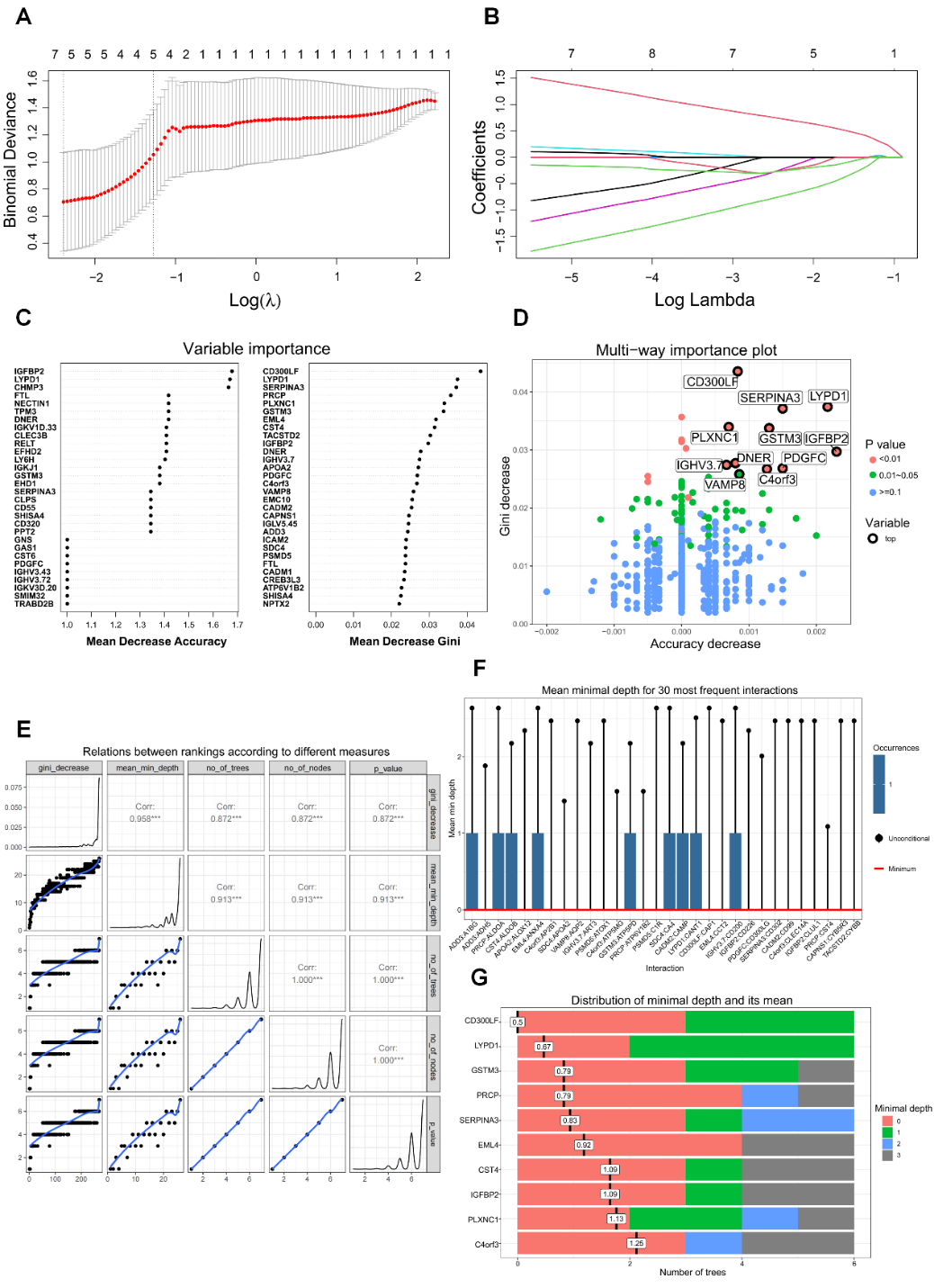


#### Supplementary Figure 22. Multidimensional evaluation and screening of proteomic features

A. Trend of mean square error with λ parameter in LASSO regression model; B. Plot of dynamic process of screening variables based on sparse estimation of LASSO regression; C. Random forest method to assess the importance of variables; D. Plot of multivariate importance; E. Correlation between different comparative measures; F. Plot of the frequency of interactions between the features; G. Plot of mean minimum depth

Figure A illustrates the determination of the λ parameter using the 10-fold cross-validation method. The left dashed line represents the value of λ corresponding to the smallest mean squared error, while the right dashed line indicates the value of λ when the mean squared error is equal to the smallest mean squared error plus one standard error. Moving on to Figure C, it displays the Mean accuracy decrease, which is the average decrease in predictive accuracy of a variable after replacement. On the right side of Figure C, we have the Mean decrease in the Gini index (Mean gini reduce), showing the average decrease in the Gini index of node impurity (indicating an increase in node purity) by splitting the variable. Both of these metrics represent variable importance, with variables appearing more important as we move towards the right on the X axis. Figure D combines the results from the two dimensions of Figure C to evaluate the importance of variables, specifically highlighting the 10 most important variables that can serve as crucial segmentation criteria. Moving on to the E plot, it demonstrates the relationship between different importance metrics, with each grid containing multiple scatters representing sample features. By analyzing the distribution of these scatters, one can identify the more important points and fit a LOESS curve. The F plot showcases the frequency of pairs of features being used together, with higher usage indicating greater contribution to the model. Lastly, the G plot represents the variable minimum depth, aiding in understanding the importance of each feature for prediction; features with smaller minimum depths are considered more important.

Potential proteomics biomarkers are screened using various methods at different levels. LASSO regression screening is highlighted for its ability to achieve sparsity by incorporating a penalty term in the optimization function, which helps identify target variables and simplifies the model while enhancing its generalization capability. The study reveals specific indicators and their corresponding coefficients, such as LILRA5 (-0.0247), SERPINA1 (0.794920434), MELTF (-0.733796423), MLEC (-0.088118223), RCN1 (0.007430372), FZD8 (-0.223734059), and PCDHGC3 (-0.182746546). Additionally, the random forest method was utilized to screen variables and rank them based on importance, resulting in the identification of the top 10 features: CD300LF, LYPD1, SERPINA3, GSTM3, PLXNC1, IGFBP2, DNER, PDGFC, VAMP8, C4orf3, and IGHV3.7. Furthermore, the decision regression tree method was employed for automatic feature selection, and the nested resampling method was used to rank features based on frequency of selection, with the top 10 features being CAPN7, C16orf89, DDR2, FBP2, RPS13, AKR7A2, CD160, HNRNPA1, INHBB, and MASP1.


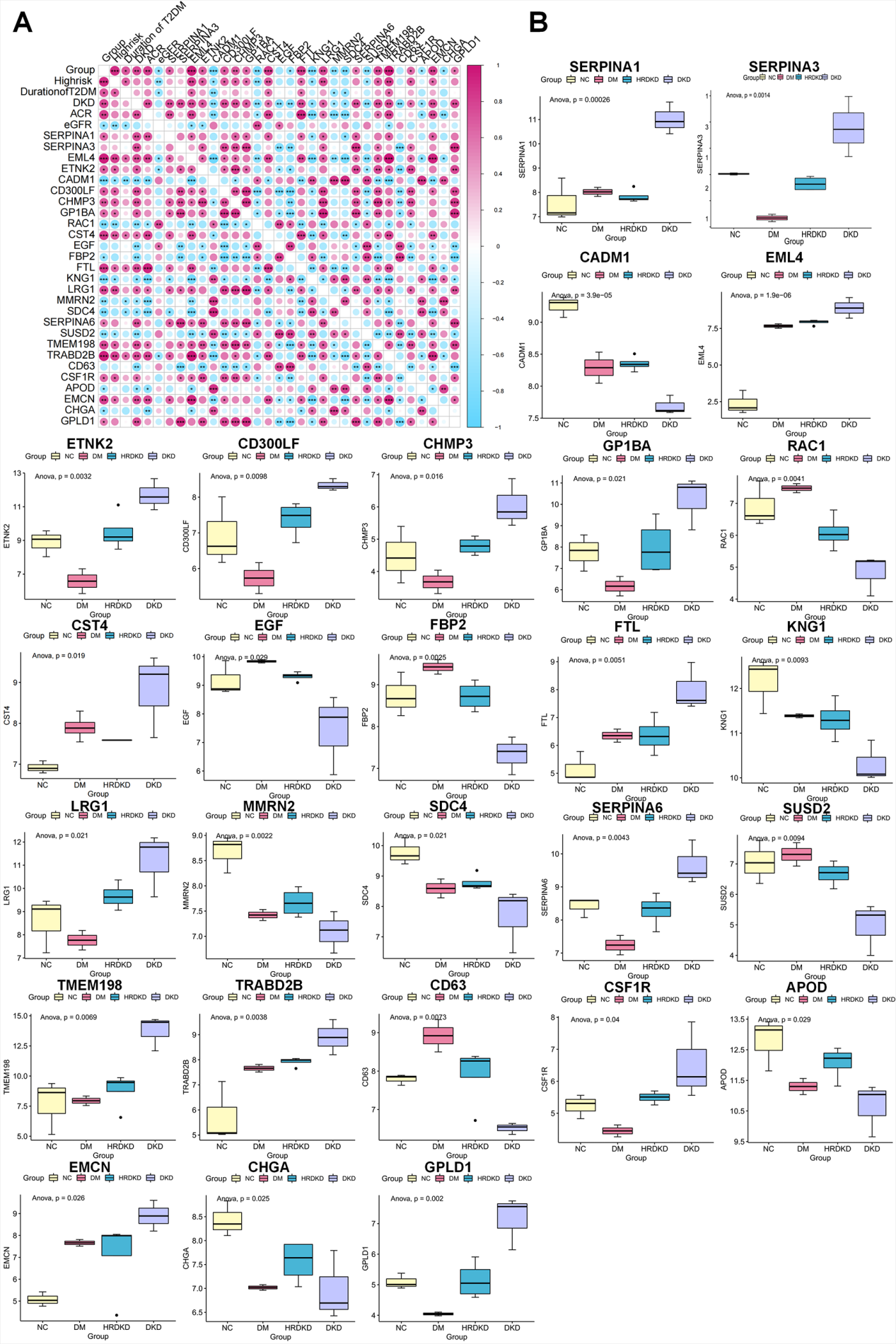


#### Supplementary Figure 23. The correlation between pre-screened proteins and expression trends across subgroups in our cohort.

1. correlation heatmap; B. box line trend plot (ANOVA analysis)

Panel A displays a correlation heatmap. All 27 variables were found to be significantly associated with subgroups. Additionally, renal function indicators such as ACR at baseline showed significant associations with SERPINA1, EML4, CADM1, RAC1, CST4, FTL, KNG1, LRG1, MMRN2, SDC4, TMEM198, TRABD2B, APOD, EMCN, and CHGA. Furthermore, eGFR at baseline was found to be significantly correlated with EML4, ETNK2, RAC1, EGF, and TRABD2B. Panel B shows a box line trend plot with ANOVA analysis. The proteins listed in the box plot are SERPINA1, SERPINA3, CADM1, EML4, ENTK2, CD300LF, CHMP3, GP1BA, RAC1, CST4, EGF, FBP2, FTL, KNG1, LRG1, MMRN2, SDC4, SUSD2, TMEM198, TRABD2B, CD63, CSF1R, APOD, CHGA, GPLD1.

**Abbreviations:** DKD: diabetic kidney disease; HC: healthy control; T2DM: type 2 diabetes mellitus; HR-DKD: high-risk diabetic kidney disease; eGFR: estimated glomerular filtration rate.

### Section 2.11. Validation of Protein Markers in the Combined Dataset


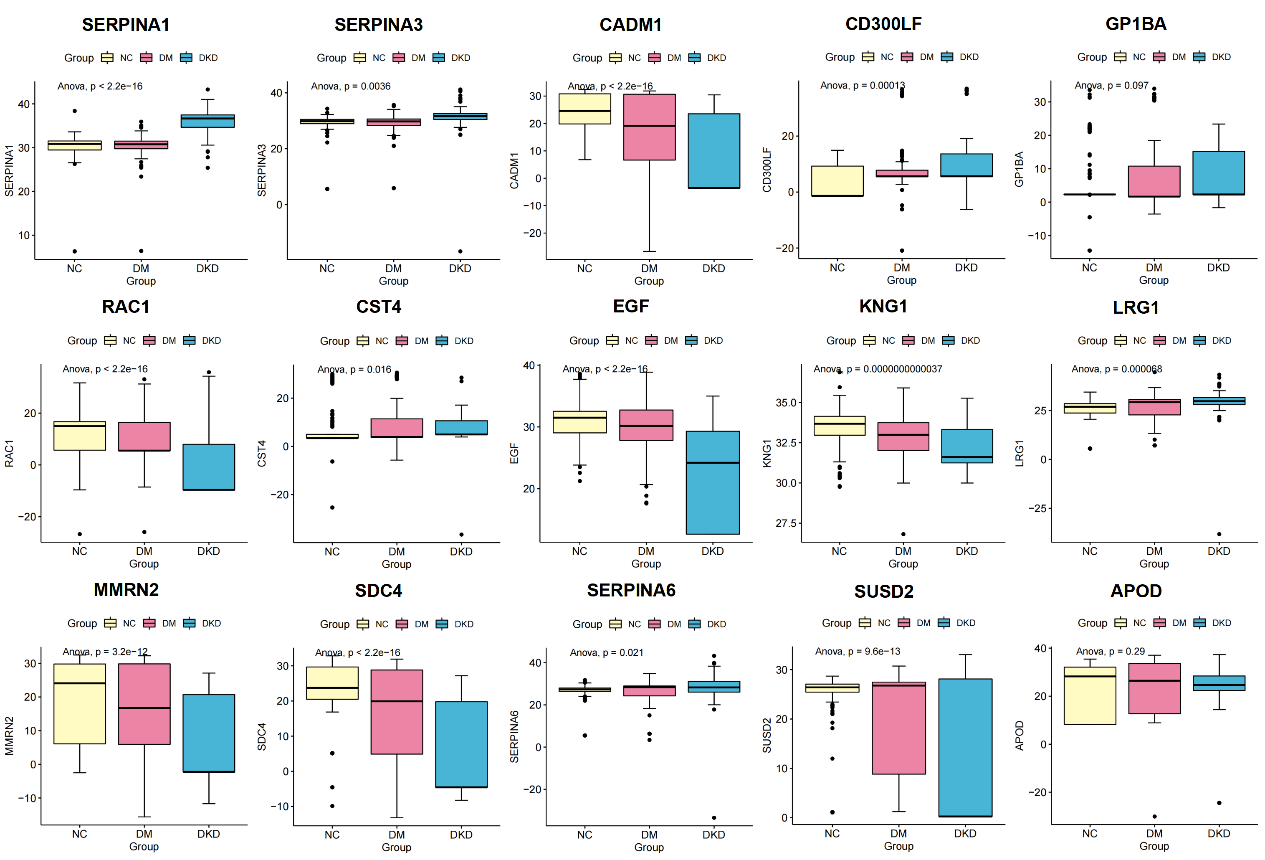


#### Supplementary Figure 24. **Validation of protein marker expression trends in the combined dataset.**

A total of 25 metrics were confirmed, including SERPINA1, SERPINA3, CADM1, CD300LF, GP1BA, RAC1, EGF, FBP2, FTL, KNG1, LRG1, MMRN2, SDC4, APOD, and CHGA. Some of these proteins have been identified as potential DKD biomarkers, such as EGF, LRG1, CD63, SERPINA1, among others. The study displayed the expression trends of the screened indicators in the external fusion dataset, revealing that all indicators exhibited consistent expression trends with the original data, except for APOD, which showed significant difference (P < 0.05).


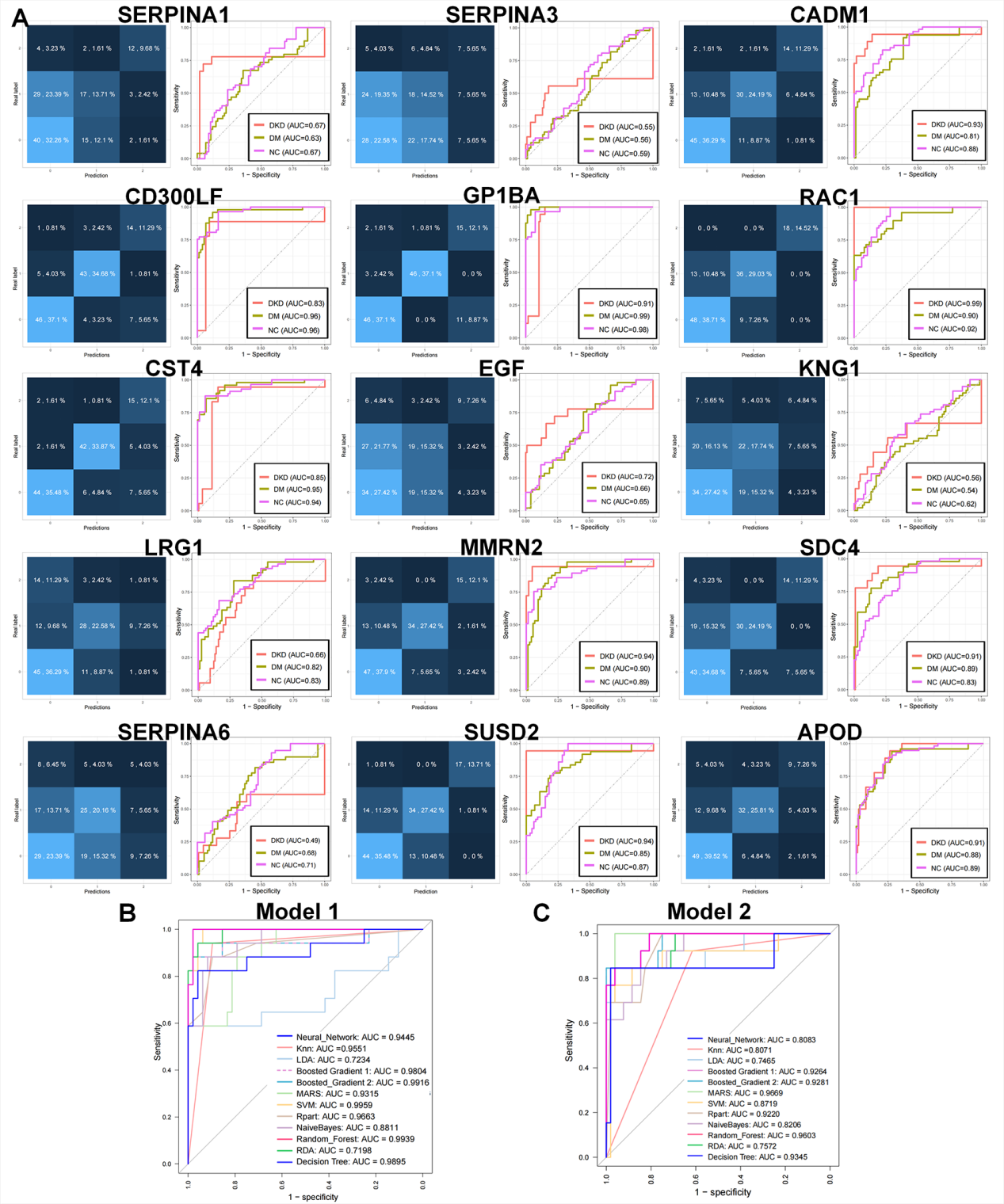


#### Supplementary Figure 25. Predictive performance of pre-screened proteins based on the Random Forest algorithm in the public database of urinary proteomics.

A. Single protein prediction model; B. Combined prediction model of CD300LF, CST4, MMRN2 and SERPINA1 (Model 1); B. Combined prediction model of SERPINA1, CADM1, RAC1 and SDC4 (Model 2);

The figure displays two graphs corresponds to one protein, with the confusion matrix on the left side and the ROC curve on the right side. The ROC curve graph predicts three categories: NC, DM, and DKD groups. Model 1 achieved a mean AUC of 0.923, outperforming model 2 with a mean AUC of 0.871. As a result, CD300LF, CST4, MMRN2, and SERPINA14 were selected for validation in cohort.

**Abbreviations:** DKD: diabetic kidney disease; HC: healthy control; DM:diabetes mellitus; ROC: receiver operating characteristic; AUC: Area under curve.

### Section 2.12. Results of the Quality Control Analysis in **Metabolomics Analysis**


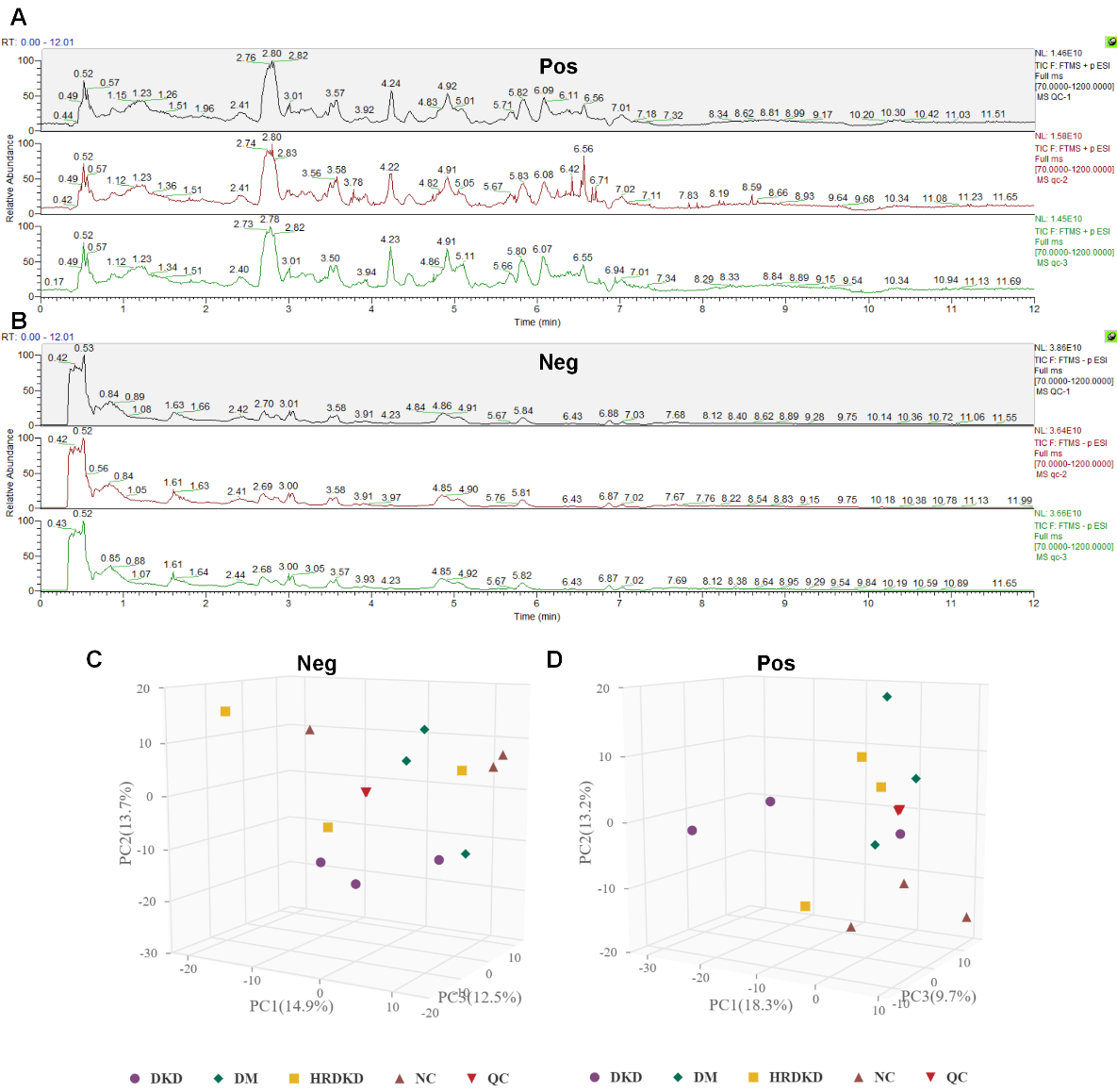


#### Supplementary Figure 26. QC sample evaluation for metabolomics.

1. Overlay spectrum of total ion chromatogram of QC sample in positive ion mode; B. Overlay spectrum of total ion chromatogram of QC sample in negative ion mode; C. PCA analysis of total sample in negative ion mode; D. PCA analysis of population samples in positive ion mode

The spectra of the total ion chromatogram (TIC) of the QC sample were compared, as shown. The experimental data showed that the response intensity and retention time of each chromatographic peak closely matched, indicating minimal variation from instrument error during the experiment. Additionally, PCA analysis was performed on the peaks extracted from all experimental and QC samples, as depicted. The findings demonstrated that the QC samples were closely grouped in both positive and negative ion modes, suggesting excellent repeatability of the experiment.


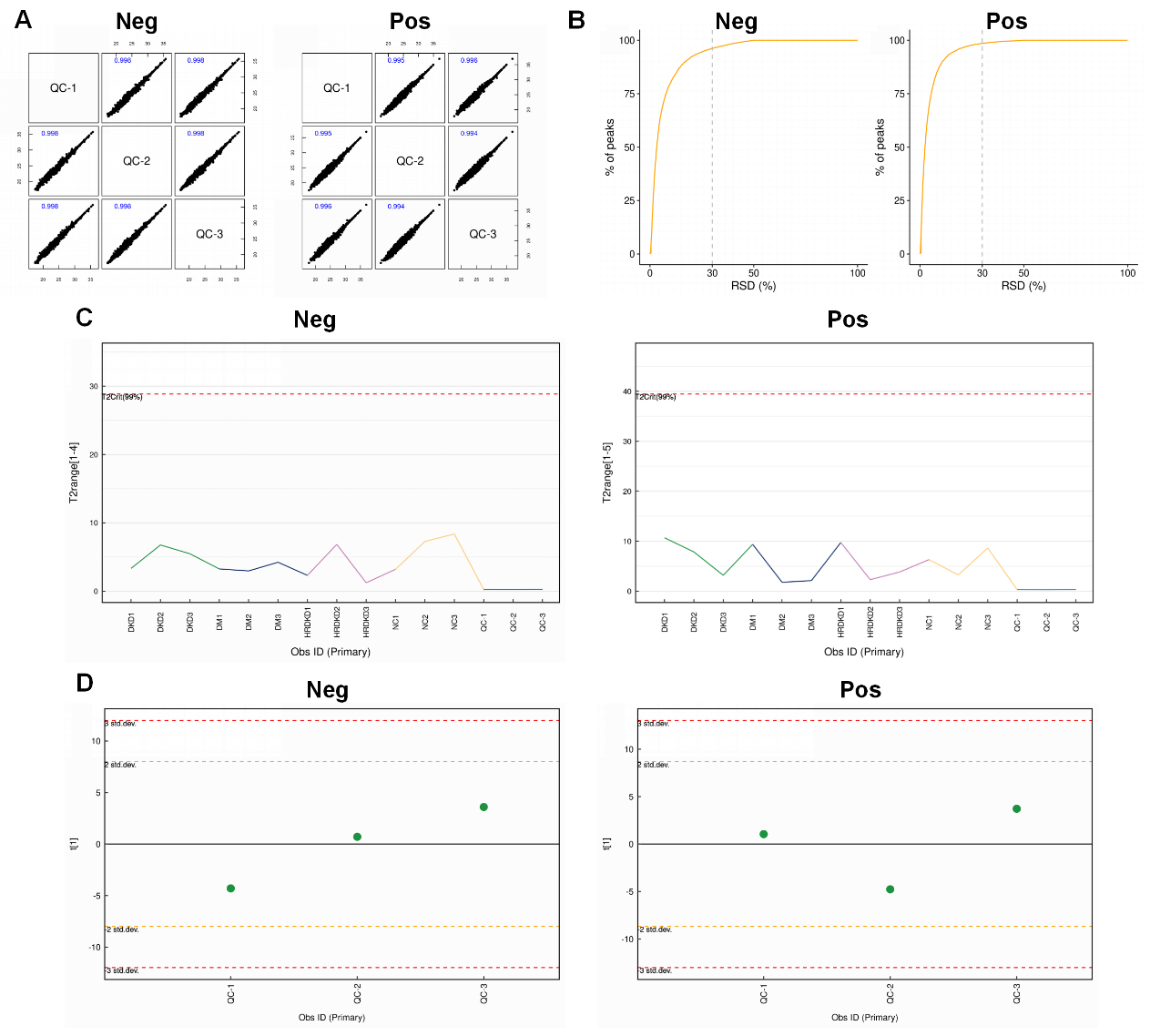


#### Supplementary Figure 27. QC sample correlation and fluctuation range measurement.

1. Correlation chart of QC samples in positive and negative ion modes; B. Hotellings T2 chart of overall samples in positive and negative ion modes; C. MCC chart of QC samples in positive and negative ion modes; D. Relative standard deviation of QC samples in positive and negative ion modes.

Pearson correlation analysis was conducted on quality control (QC) samples. The results indicated that the correlation coefficients between QC samples were all above 0.9, demonstrating good experimental repeatability. Furthermore, over 70% of the peaks in the QC samples had a relative standard deviation (RSD) of less than or equal to 30%, indicating stability in the instrument analysis system. The data generated from this experiment can be reliably used for further analysis. Hotelling's T2 test results showed that all QC samples fell within the 99% confidence interval, reinforcing the experiment's repeatability. Additionally, the multivariable control chart of the QC samples revealed that fluctuations were within plus or minus 3 standard deviations, confirming that the instrument's variations were within normal limits and validating the data for subsequent analysis. The figure displays ion peaks (metabolites) extracted from each QC sample. The logarithm of the ion peak signal intensity value is depicted on both the x and y axes. The x-axis represents all experimental and QC samples, while the y-axis shows the confidence interval with the 99% range indicated by a red line. In Figure C, each QC sample is plotted on the x-axis, and the standard deviation is shown on the y-axis, with the plus and minus 2 and 3 standard difference ranges delineated by yellow and red lines.

### Section 2.13. Differential Metabolite Analysis among Different Groups.


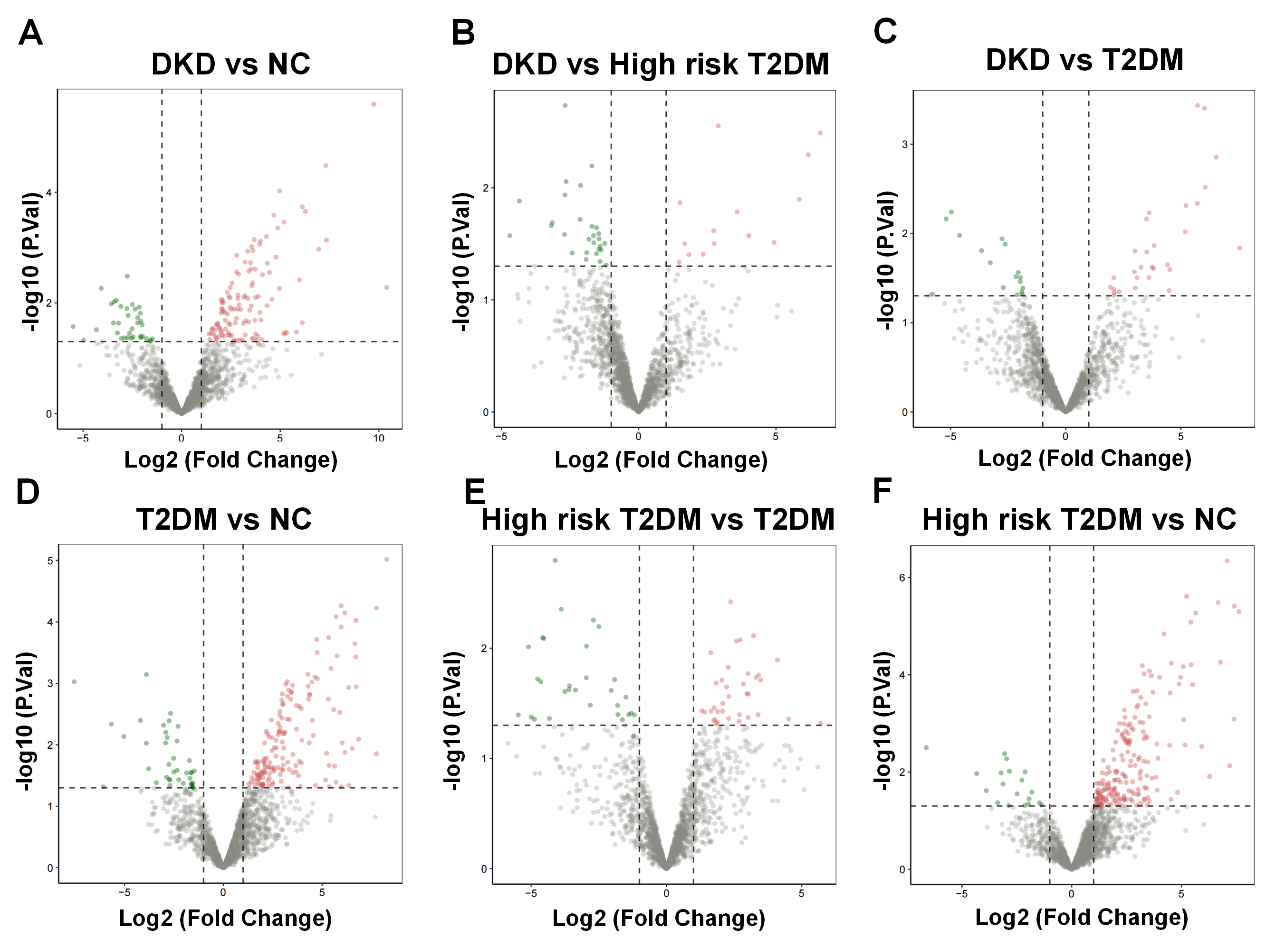


#### Supplementary Figure 28**. Volcano plots**

1. DKD vs HC group; B. DKD vs HR-DKD group; C. DKD vs T2DM group; D. DKD vs HC group; E. DKD vs T2DM group; F. HR-DKD vs HC group.

Differential metabolites are identified by screening for significance with a P-value less than 0.05 and a fold change greater than 0.5. These metabolites are subsequently categorized into groups for pairwise comparison and visually depicted in a volcano diagram.


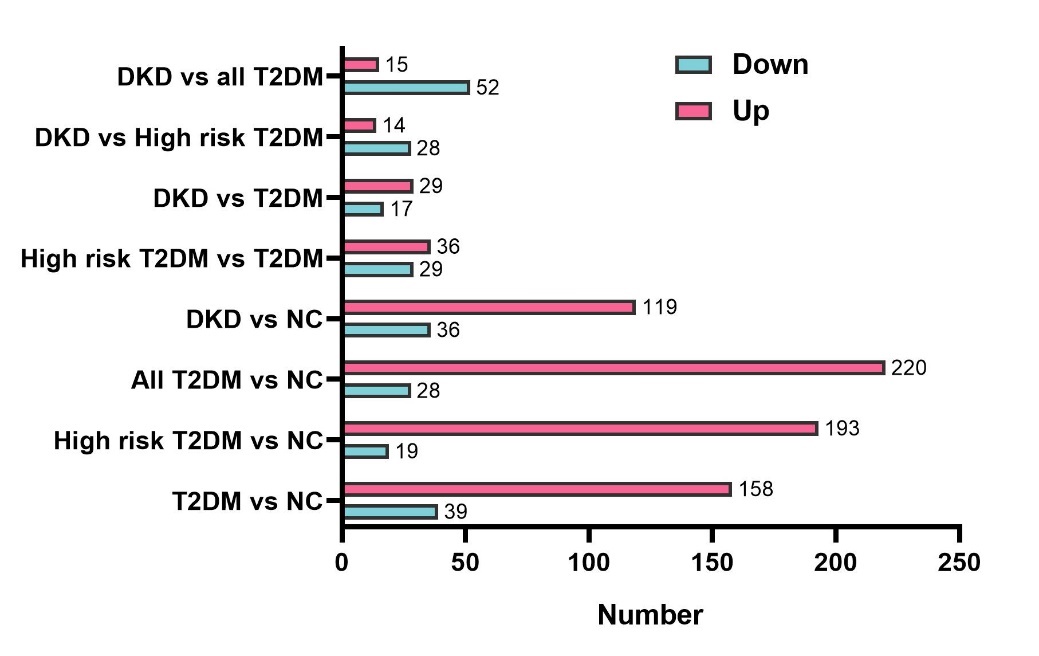


#### Supplementary Figure 29**. Statistics on the number of up-regulated and down-regulated differential metabolites compared between different groups.**

A bar chart was utilized to illustrate the quantity of differential metabolites, allowing for a visual comparison of differential expression patterns across various groups. sFigure 27 showcases unique metabolomics differential expression profiles for both the early and late stages of the disease. The early stage primarily displays up-regulation of metabolites, while down-regulation is more prevalent in the later stages of the disease.

### Section 2.14. Overlap of Differential Metabolite Identifications among Different Groups.


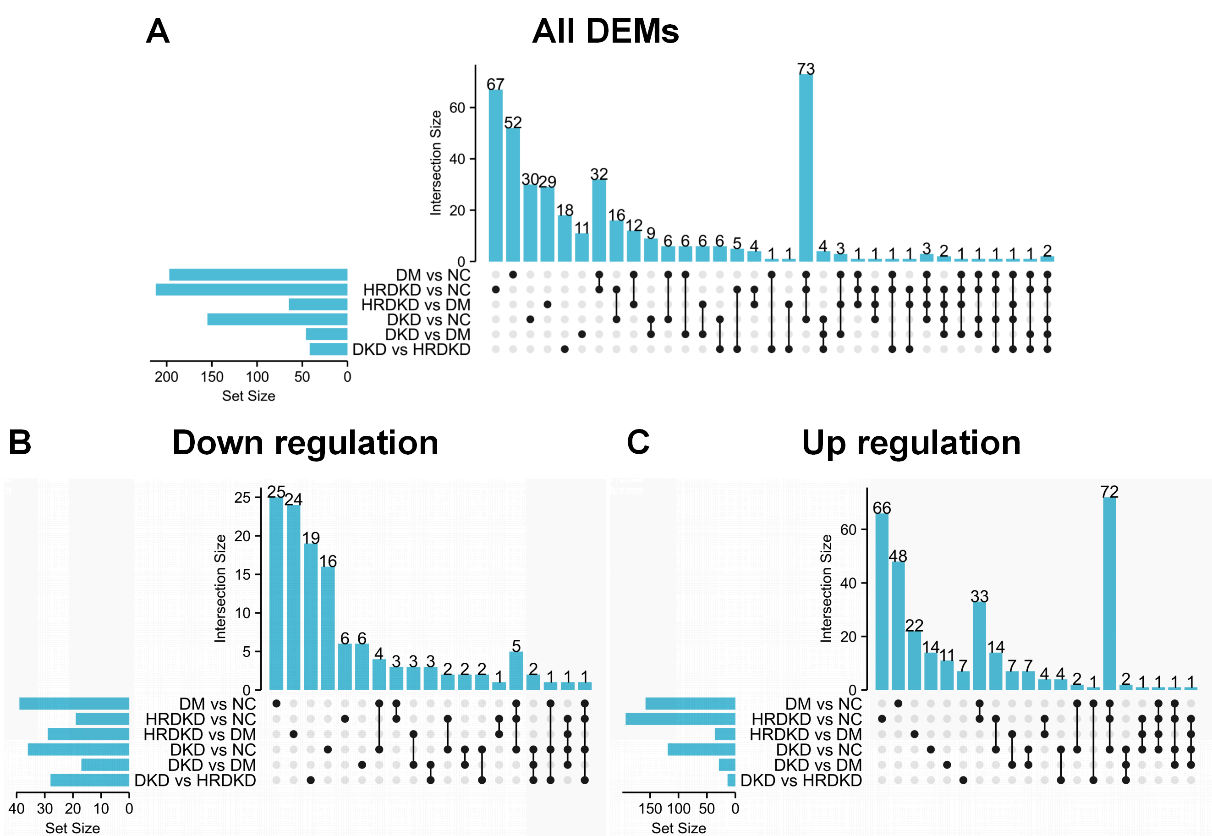


#### Supplementary Figure 30**. Overlap of differential metabolite identification between different groups.**

1. Overlap of all differential metabolites; B. Overlap of down-regulated metabolites between different groups; C. Overlap of up-regulated metabolites between different groups;

By identifying the overlapping differential metabolites across different groups, we conducted a comprehensive analysis and visualized the results using an Upset chart. The analysis revealed three distinct groups: DM vs NC, HRDKD vs NC, and DKD vs NC. Specifically, there were 80 differential metabolites unique to DKD compared to the other three groups, with 6 metabolites showing differential expression. Furthermore, 78 metabolites exhibited increased levels across all three groups, while 10 metabolites showed decreased levels.

### Section 2.15. Heat Map and Functional Analysis of Differential Metabolites between Different Groups.

**T2DM and HC group**


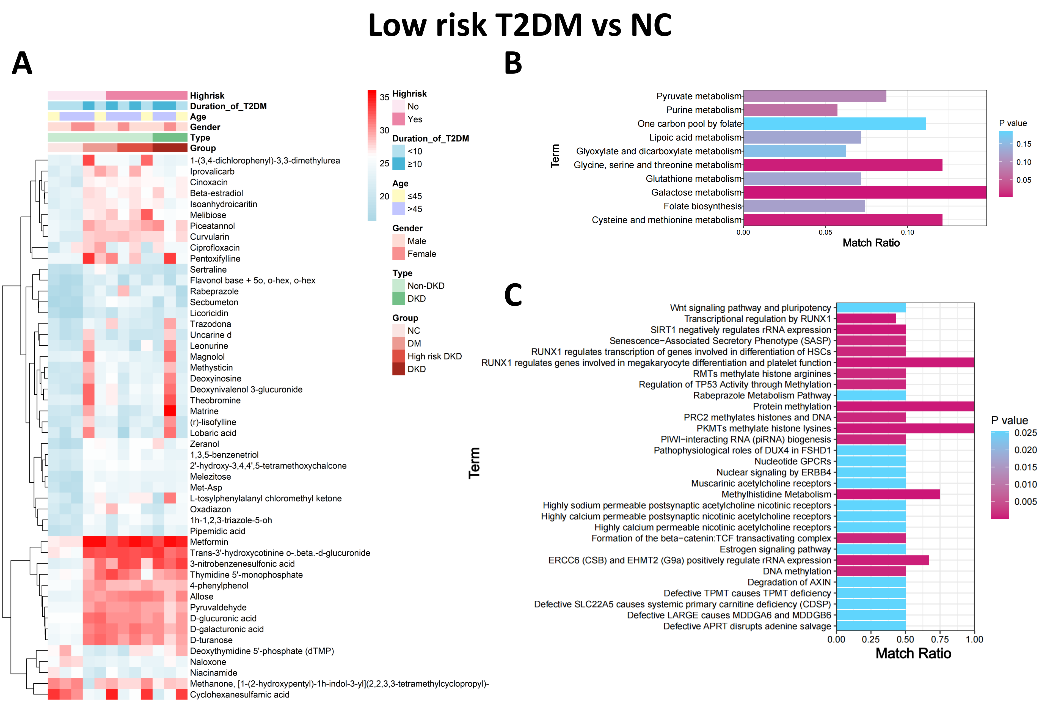


#### Supplementary Figure 31**. Display and functional analysis of the Top50 differential metabolite.**

1. Heat map of the Top 50 DEMs; B. KEGG enrichment analysis bar chart; D. RaMP enrichment analysis bar chart

Following the sorting and screening of the Top50 Differentially Expressed Metabolites (DEMs) based on |log2FC| values, a heat map was generated to visualize the expression of these DEMs. sFigure 29 illustrates the comparison between the diabetic group and the normal control group, highlighting the significant increase in metabolites such as Allose, 3-nitrobenzenesulfonic acid, and Trans-3'-hydroxycotinine o-.beta.-d-glucuronide in diabetic patients compared to normal individuals. The KEGG enrichment analysis revealed enrichment in pathways related to galactose metabolism, glycine, serine, and threonine metabolism, as well as cysteine amino acid and methionine metabolism. Furthermore, the RaMP pathway analysis indicated changes in gene regulation associated with megakaryocyte differentiation, platelet function, and aging-related secretion phenotypes during disease progression at this stage.

**HR-DKD and T2DM group**


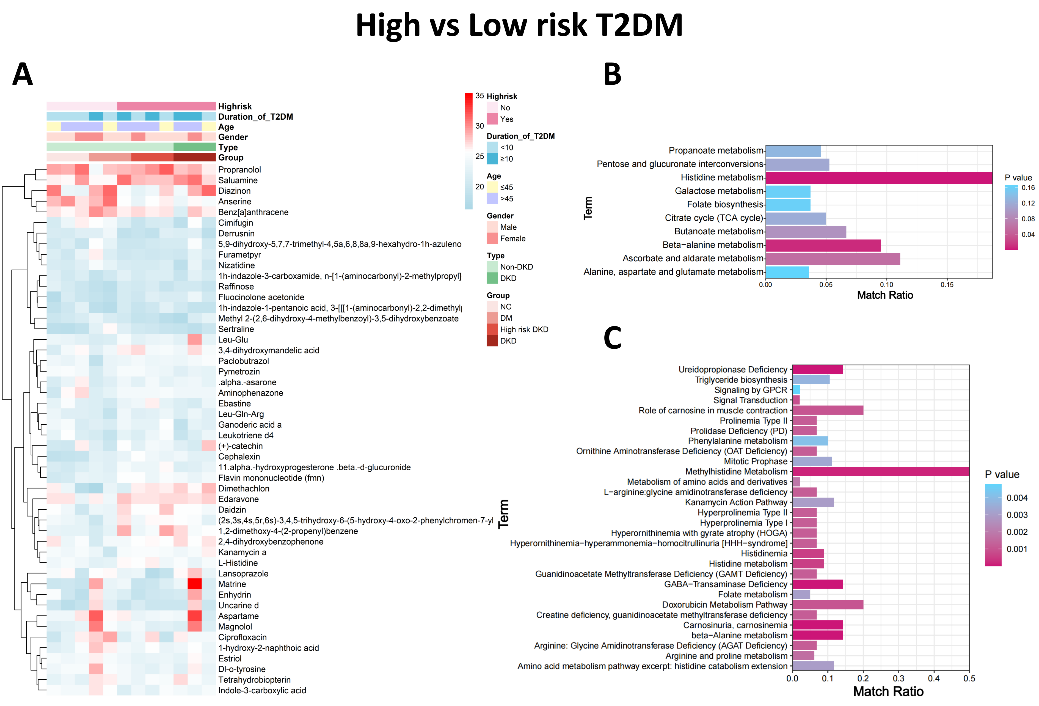


#### Supplementary Figure 32**. Display and functional analysis of the Top50 differential metabolite.**

1. Heat map of the Top 50 DEMs; B. KEGG enrichment analysis bar chart; D. RaMP enrichment analysis bar chart.

In comparison to the low-risk group of diabetic nephropathy, metabolites such as Benzanthracene, Anserine, and Diazinon decreased, while Saluamine increased. For more details. The KEGG analysis showed a significant enrichment in histidine metabolism and β-alanine metabolism. Additionally, the RaMP pathway enrichment changes during disease progression at this stage primarily focused on troponinuria, methylhistidine metabolism, and other related pathways.

**DKD and HR-DKD group**


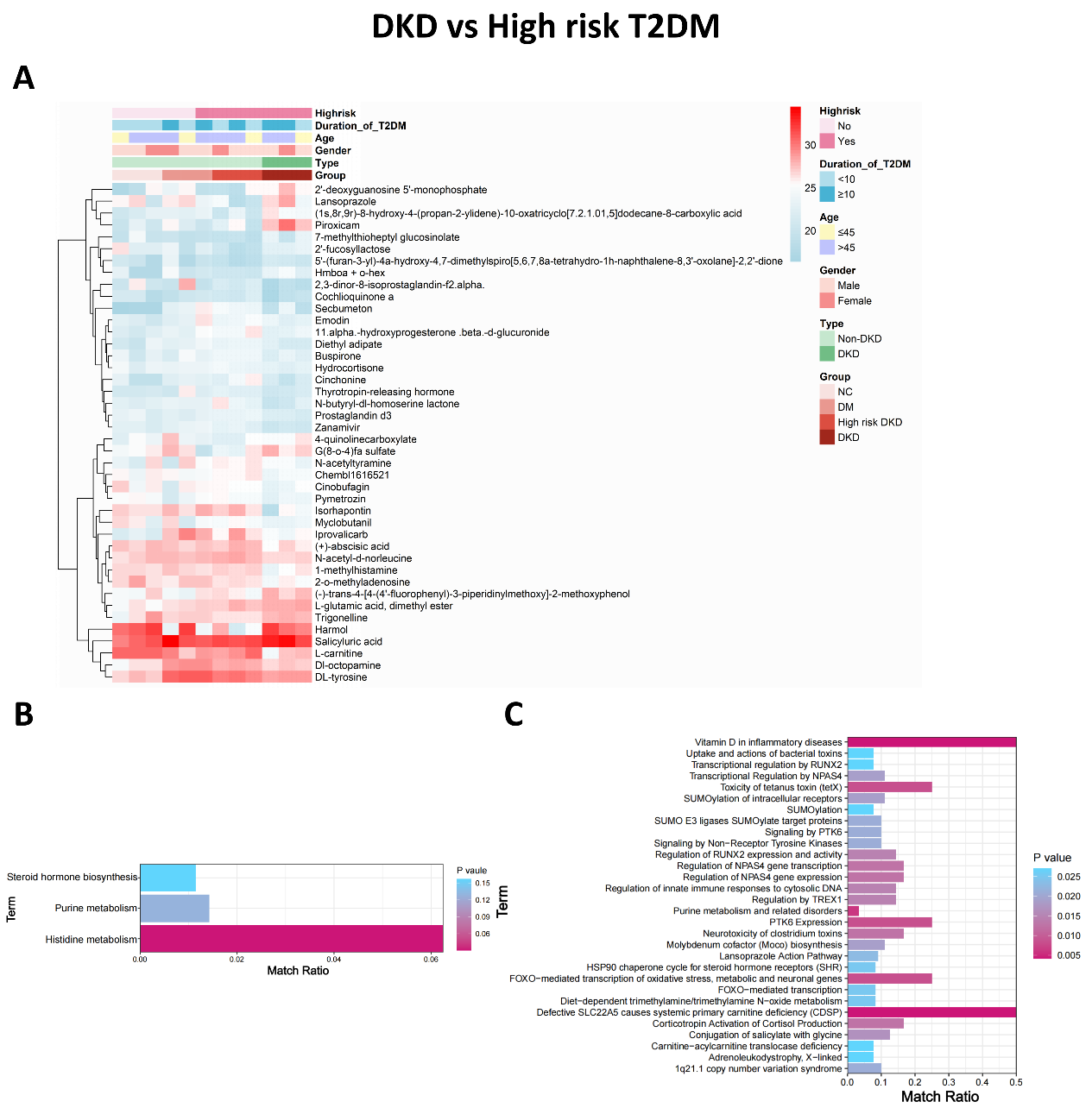


#### Supplementary Figure 33**. Display and functional analysis of the Top50 differential metabolite.**

1. Heat map of the Top 50 DEMs; B. KEGG enrichment analysis bar chart; D. RaMP enrichment analysis bar chart.

Compared to the HRDKD group, metabolites such as Lansoprazole, 2'-deoxyguanosine 5'-monophosphate, 4-quinolinecarboxylate, and G(8-0-4)fa sulfate were found to be elevated, while lsorhapontin and lprovalicarb were significantly reduced. The KEGG analysis revealed significant enrichment in purine metabolism and histidine metabolism. Additionally, the RaMP pathway analysis indicated changes in disease progression, particularly in systemic primary carnitine deficiency and vitamin D-related inflammatory diseases due to SLC22A5 deficiency.

### Section 2.16. Differential Metabolites among Multiple Groups


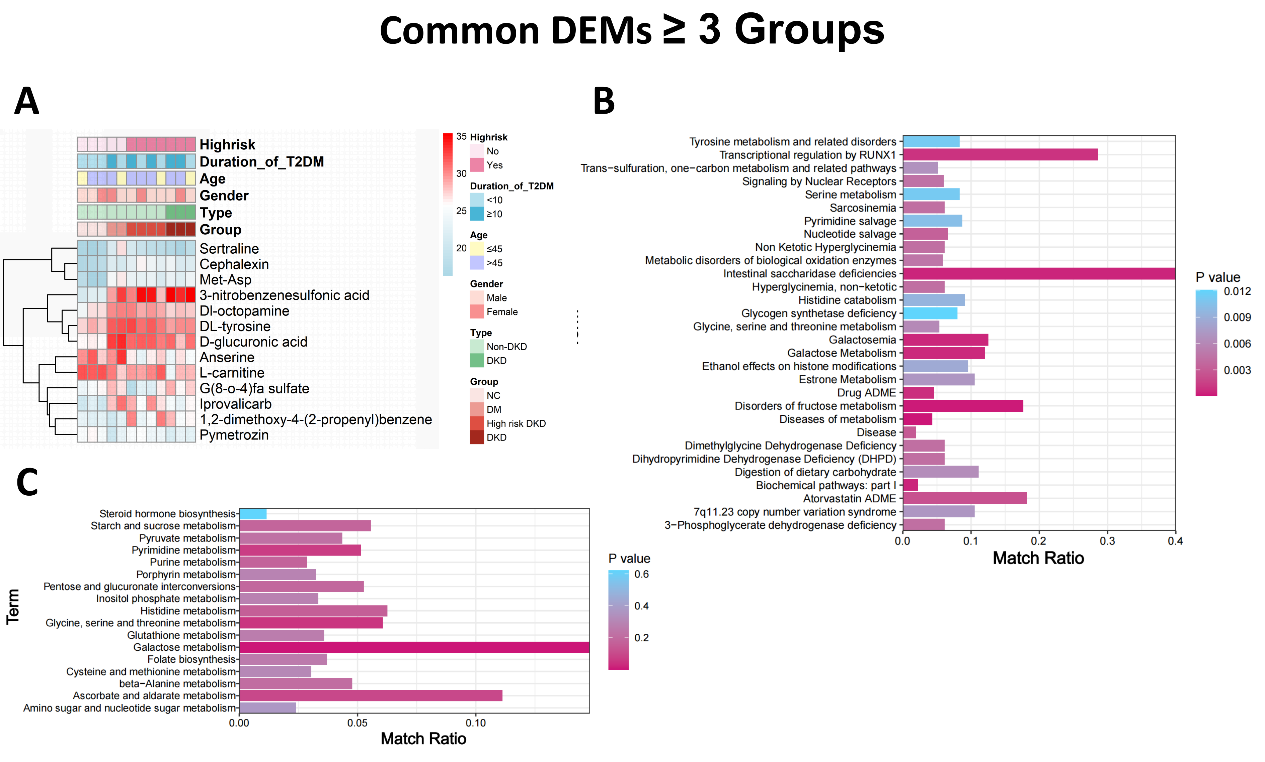


#### Supplementary Figure 34**. Heat map and functional analysis of shared differential metabolites between multiple groups**

1. Heat map of Top 50 DEMs; B. KEGG enrichment analysis bar chart; D. RaMP enrichment analysis bar chart

Thirteen differential metabolites have been identified across multiple groups. Among them, 3-nitrobenzenesulfonic acid and Met-Asp show an increase with disease progression, while Anserine and L-carnitine exhibit a decrease as the disease advances. The KEGG analysis reveals significant enrichment in glycine, serine, and threonine metabolism, pyrimidine metabolism, ascorbic acid and aldehyde metabolism, and galactose metabolism. The RaMP pathway enrichment analysis of these metabolites primarily focuses on disorders in fructose metabolism, galactose metabolism, and transcriptional regulation involving RUNX1.

### Section 2.17. Mfuzz Temporal Expression Cluster Analysis


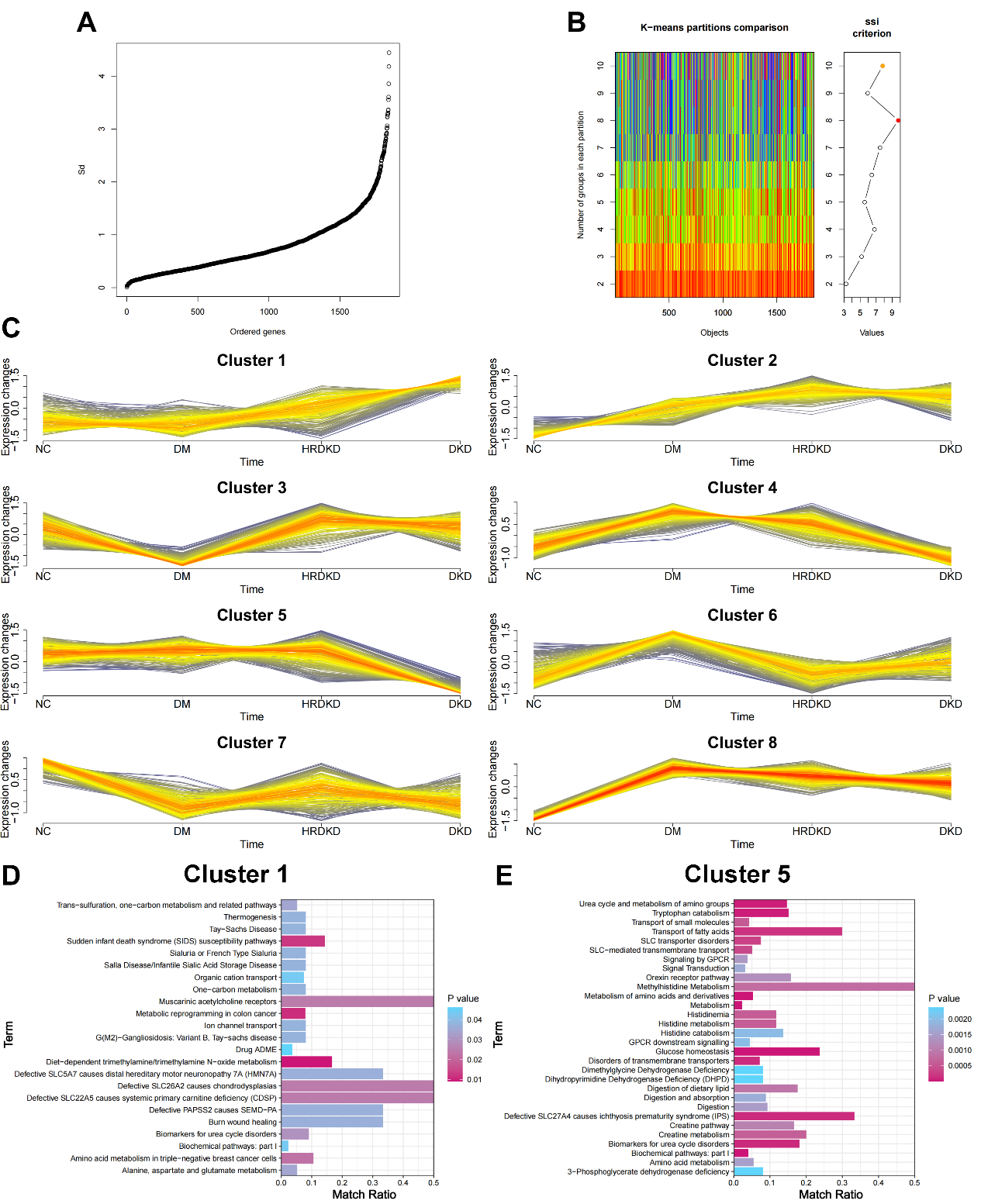


#### Supplementary Figure 35**. Mfuzz Time series expression trend analysis**

1. Standard deviation method to filter proteins; B. Evaluation of the optimal number of clusters by NbClust method; C. Analysis of metabolite levels based on fuzzy c-means algorithm Time series cluster analysis (8 clusters); D. KEGG analysis of Cluster 1; E. KEGG analysis of Cluster 5

Utilizing Mfuzz, this study explores the temporal dynamics of the metabolic map to analyze metabolites with similar patterns, aiding in understanding the relationship between dynamic metabolite patterns and different stages of DKD. Initially, metabolites were filtered, and the NbClust method determined that 8 clusters would be optimal, resulting in 8 distinct groups of metabolite clusters with varying kinetic modes. The kinetic patterns differ among clusters; for example, Cluster 1 shows a positive correlation with disease progression, while Cluster 5 exhibits a negative correlation. The metabolite expression matrix was then analyzed to investigate molecular changes within the clusters. Functional changes in molecular Cluster 1 are primarily associated with diet-dependent trimethylamine/trimethylamine N-oxide (TMAO) metabolism, SLC22A5 deficiency leading to systemic primary carnitine deficiency and urea cycle disorder, as well as biomarkers related to alanine, aspartate, and glutamate metabolism, trans-sulfation, one-carbon metabolism, and similar pathways. On the other hand, molecular functional changes in Cluster 5 focus on tryptophan metabolism, biomarkers for urea cycle disorders, SLC transporter disorders, histidine metabolism, and other pathways, reflecting the active biological processes in the progression of DKD.

### Section 2.18. Statistical Analysis of Molecular Characteristics and Organ Sources of Differential Metabolites.


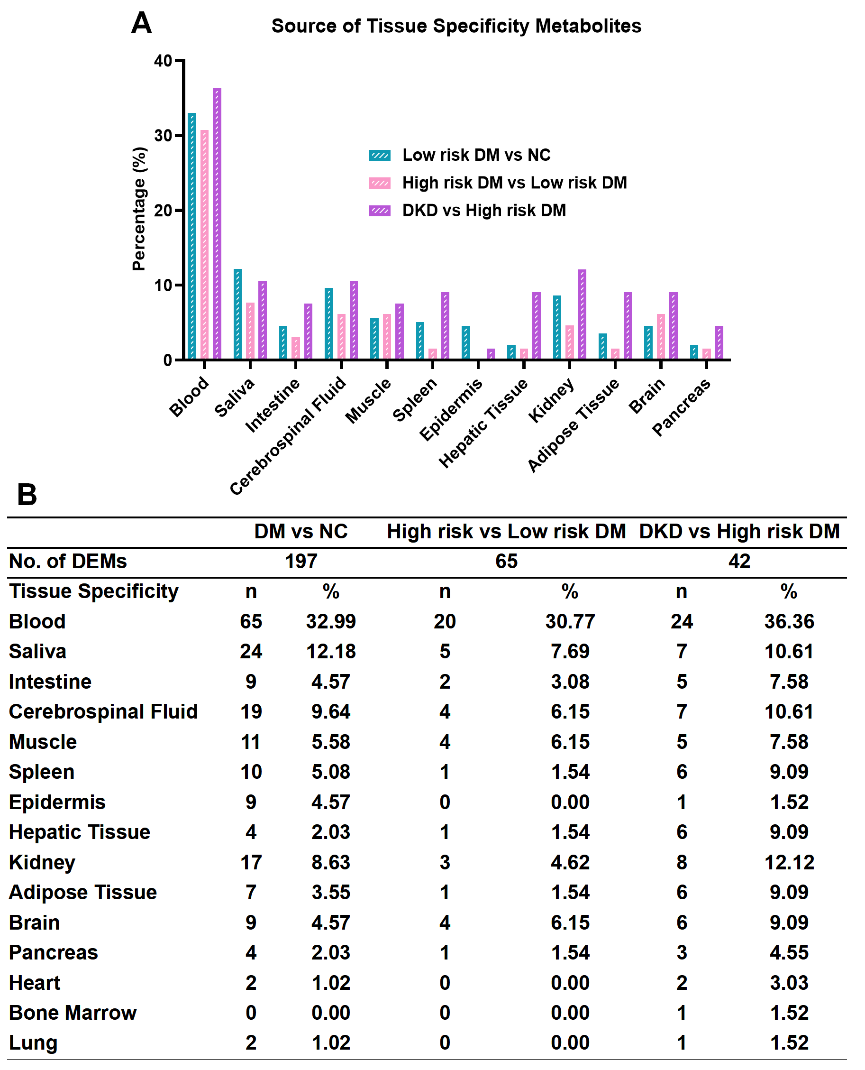


#### Supplementary Figure 36**. Tissue and organ-specific sources of differential metabolites in different stages of DKD**

1. Statistical bar chart; C. Number and percentage of statistical tables

Metabolites originating from various organs and tissues are excreted by the kidneys and may play a role in the progression of diabetic kidney damage. The sources of differential metabolites from different organs and tissues are depicted at various stages of DKD progression. The data illustrates an increase in both the number and percentage of metabolites derived from muscles, kidneys, adipose tissue, brain, and liver as the disease progresses. Notably, there is a significant rise in the proportion of liver-derived metabolites in the critical stage of DKD (9.09% vs 2.03%). Examples of these metabolites include Cortisol, Lansoprazole, L-Carnitine, and others.


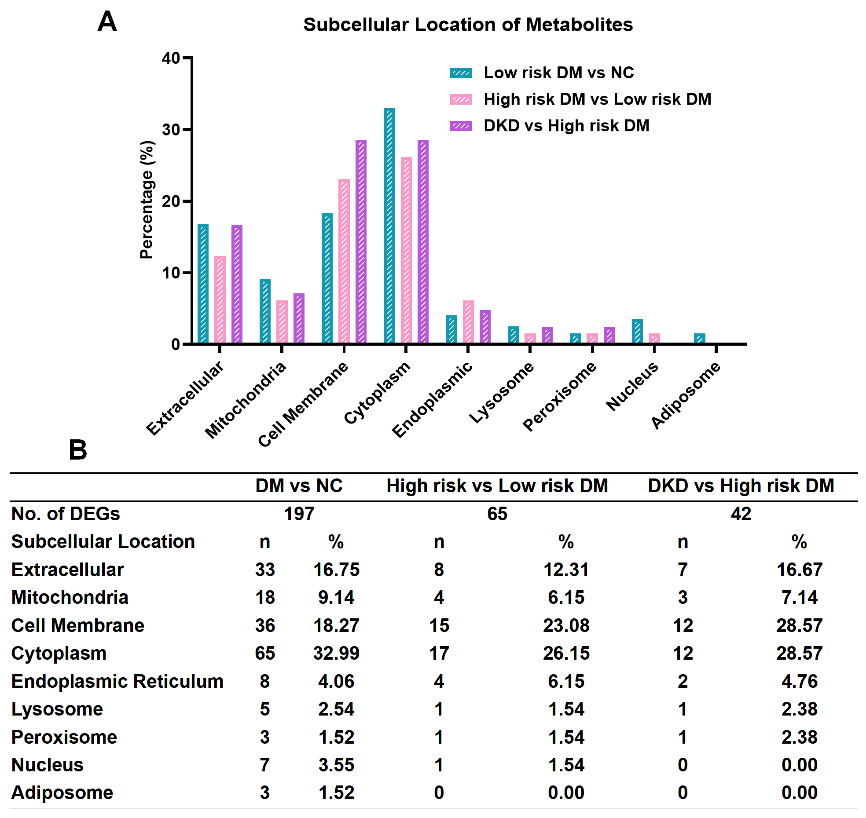


#### Supplementary Figure 37**. Subcellular localization of differential metabolites in different stages of DKD**

1. Statistical bar chart; C. Number and percentage of statistical tables

The study demonstrates the subcellular localization of distinct metabolites at various disease stages. It was observed that metabolites originating from the cell membrane exhibit an increase as diabetic kidney disease (DKD) advances. Some examples of cell membrane-derived metabolites identified include Zanamivir, (S)-Abscisic acid, Lansoprazole, Piroxicam, and Buspirone.


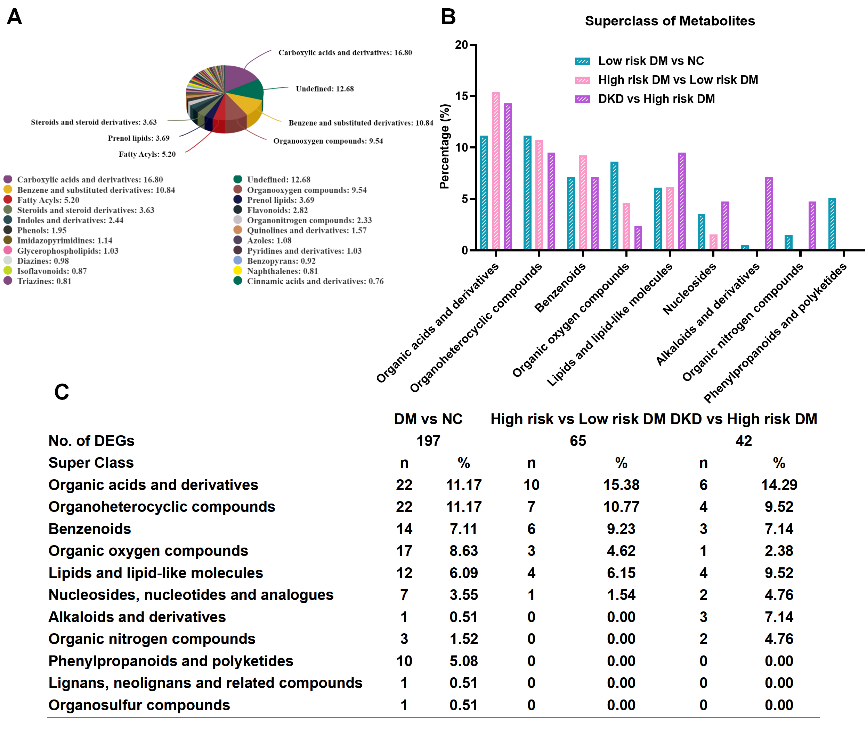


#### Supplementary Figure 38**. Chemical classification and assignment statistics of differential metabolites in different stages of DKD**

1. Chemical classification and assignment statistics of all identified metabolites; B. Bar chart of chemical classification and assignment statistics of differential metabolites in different stages of DKD; C. DKD Statistical table of chemical classification and attribution of differential metabolites at different stages.

The chemical classification attribution statistics of differential metabolites in various stages of DKD indicate that carboxylic acid and its derivatives represent the highest proportion among all identified metabolites (16.8%). Lipid molecules account for 9.52%, nucleosides, nucleotides, and analogs for 4.76%, alkaloids and their derivatives for 7.14%, and organic nitrogen compounds for 4.76% during the critical stage of DKD onset. Interestingly, the proportion of organic oxygen compounds decreases as the disease progresses from 8.63% to 4.62% and finally to 2.38%.

### Section 2.19. Common Pathway Enrichment Changes and Metabolic Pathway Changes in Different DKD Stages.


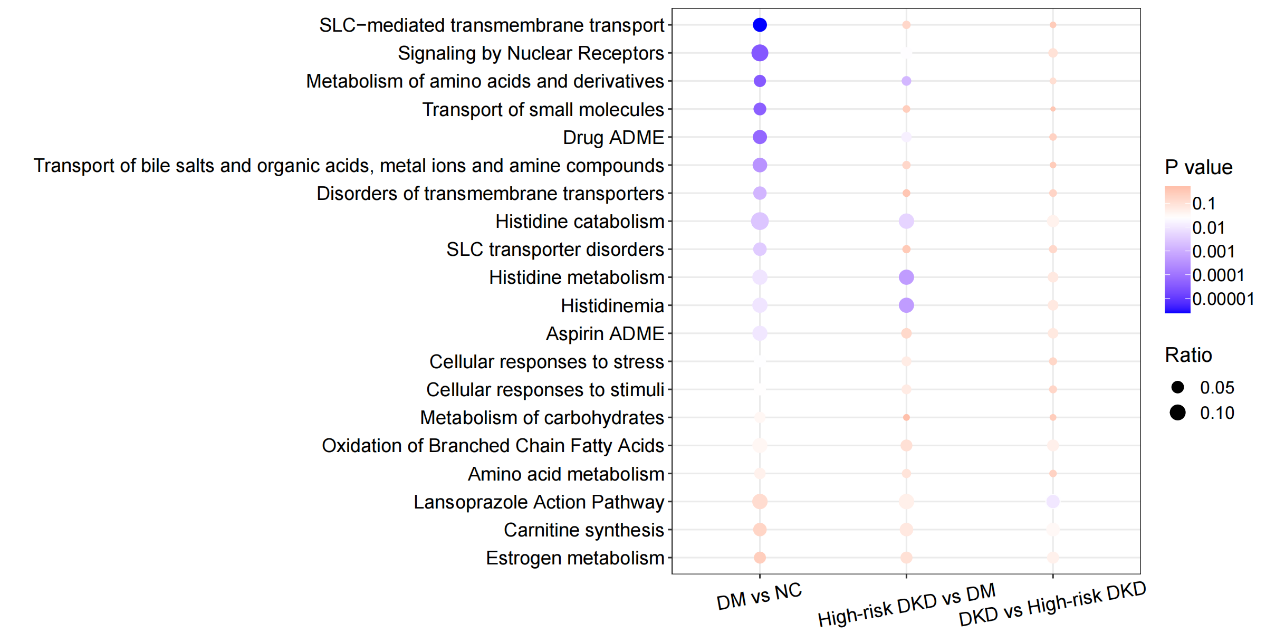


#### Supplementary **Figure 39. Common RaMP pathway enrichment changes and metabolic pathways enriched in different DKD stages**

By comparing the pathways involved in metabolites at different stages of DKD, we can explore the closely related biological processes involved in the progression of DKD and the common mechanisms of regulation. The activity of pathways such as SLC-mediated transmembrane transport, nuclear receptor signaling, transport of bile salts and organic acids, metal ions, and amine compounds decreases with the progression of DKD. Conversely, pathways related to histidine metabolism, amino acid metabolism, cell response to stress, branched-chain fatty acid oxidation, lipid peroxidation, carnitine synthesis, and metabolic pathways associated with the innate immune system remain active throughout the process from DM to DKD. This suggests their significant role in the critical transition process of DKD.

### Section 2.20. WGCNA Analysis


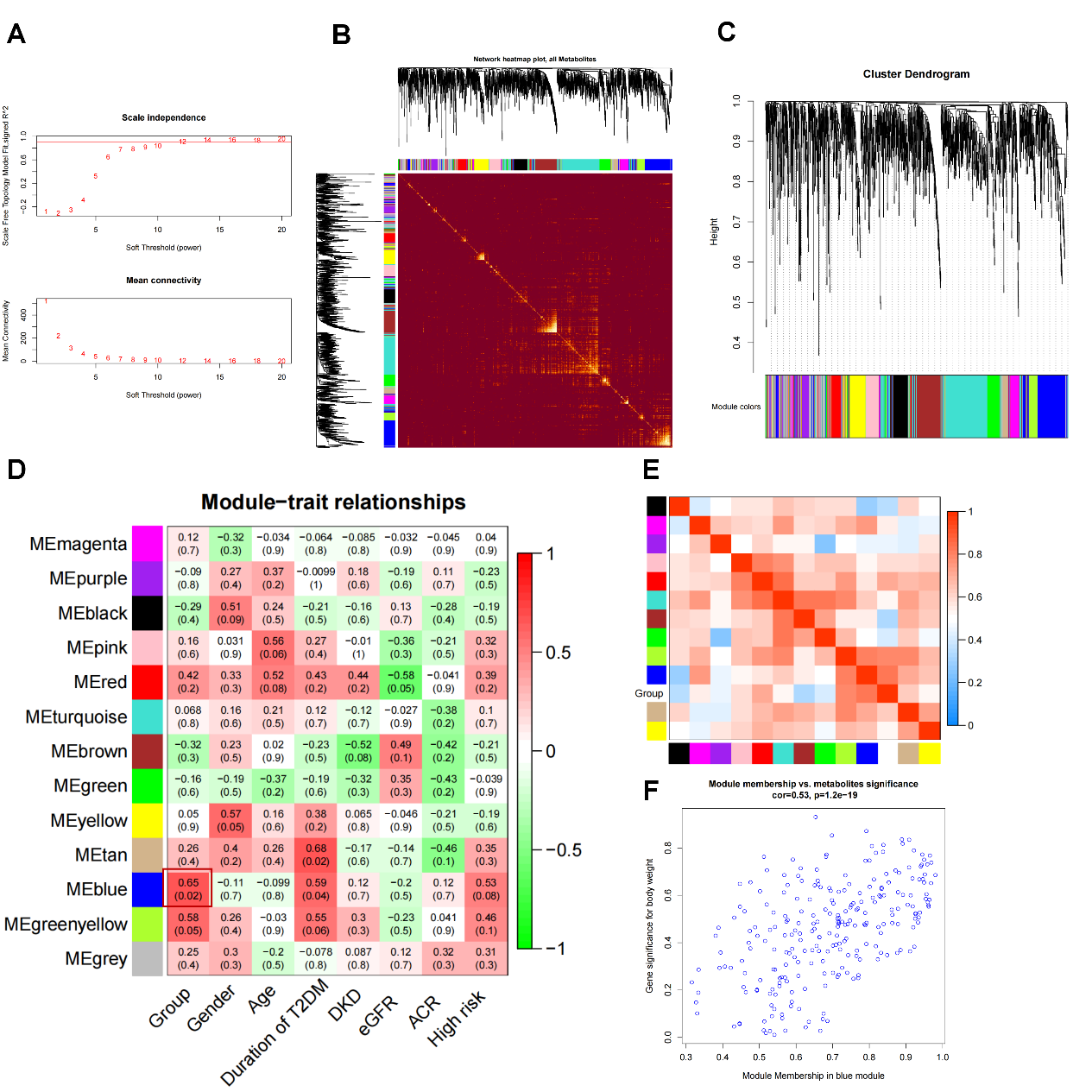


#### Supplementary **Figure 40. Weighted gene co-expression network analysis**

1. Power diagram to select appropriate thresholds; B. Topological overlap matrix to construct co-expression network; C. Clustering dendrogram; D. Module feature correlation heat map; E. Group and Analysis correlation diagram within the module; F. Group and blue module correlation diagram

This section employs WGCNA analysis to cluster metabolites into different modules and pinpoint core modules linked to key features. The process involves calculating the soft threshold using the pickSoftThreshold function, where a higher Power value indicates more reliable research outcomes. With an optimal threshold of 12 chosen, the adjacency matrix is transformed into a topological overlap matrix to reduce noise, leading to the creation of a TOM diagram. Subsequently, a clustering dendrogram is constructed based on the distance matrix derived from the TOM matrix, resulting in the categorization of 1845 metabolites into 13 modules. The correlation map between modules and phenotypic characteristics highlights the significant association of the blue module with grouping (P = 1.2e-18).

### Section 2.21. Enrichment Analysis of Differential Protein Combined Metabolome.

**T2DM and HC group.**


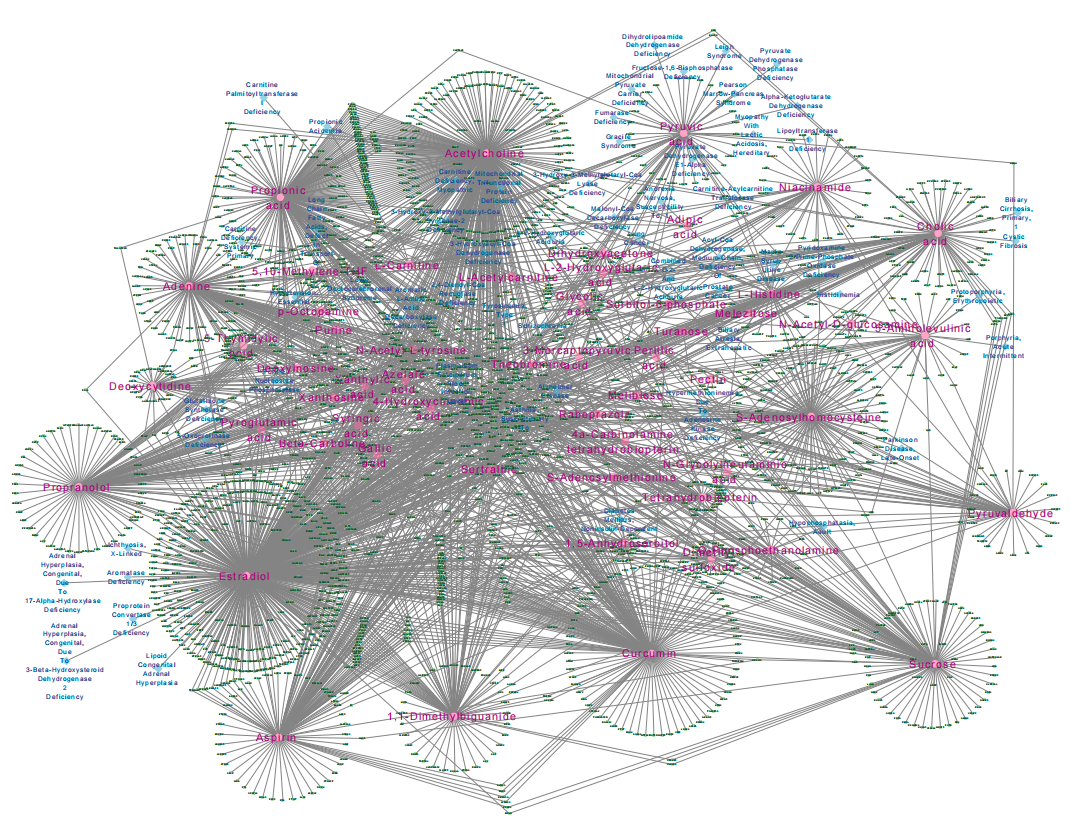


#### Supplementary **Figure 41. Differential protein-metabolite-disease network relationship diagram**

By analyzing the relationship network between differential metabolites and differential proteins in the diabetic group compared to the normal control group, several major differential metabolites were identified. Among these, L-Carnitine stood out as the primary differential metabolite, with strong associations to genes such as SLC22A4, SLC22A5, SLC22A16, CRAT, and others, as well as type 2 diabetes. Additionally, Pyruvaldehyde, 1,5-Anhydrosorbitol, and S-Adenosylmethionine were also found to be linked to type 2 diabetes.

**HR-DKD and T2DM group**


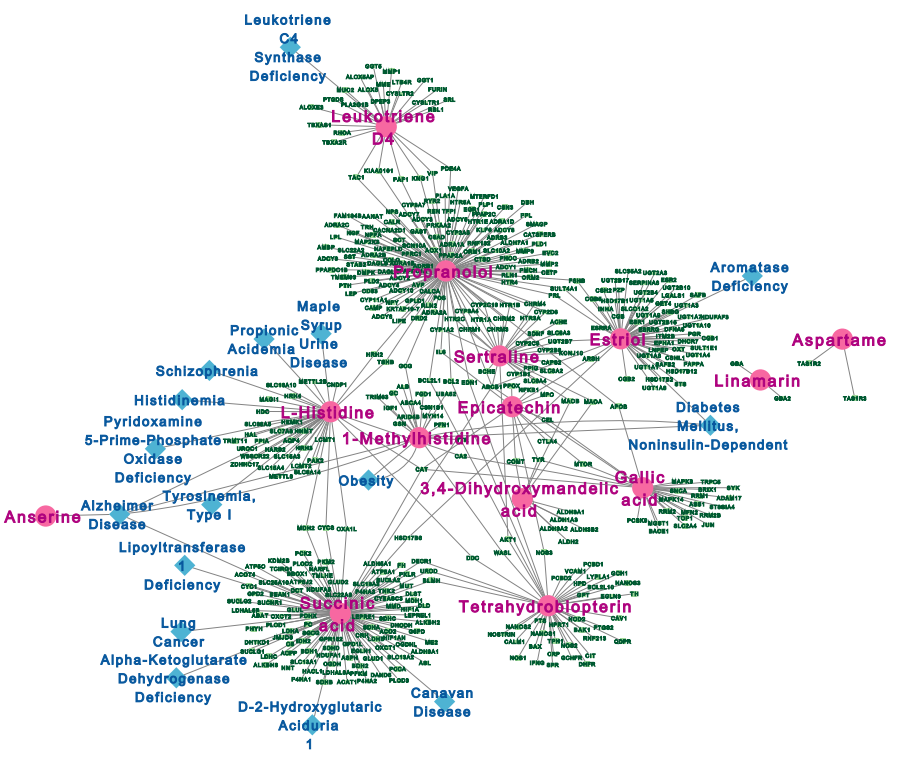


#### Supplementary Figure 42. Differential protein-metabolite-disease network relationship diagram

The network diagram illustrates the relationship between differential metabolites and proteins in the high-risk diabetic nephropathy group compared to the diabetes group. One important finding is that 1-Methylhistidine is a key differential metabolite, showing strong associations with genes like IGF-1, ABCA4, and TTR. Additionally, it is linked to various diseases such as Alzheimer's disease, type 2 diabetes, and obesity. Leukotriene D4 is primarily associated with genes like MMP1, ALOXE3, and ALOX5. Conversely, Gallic acid shows connections with genes such as SYK, ADAM17, and PCSK9.

**DKD and HR-DKD group**


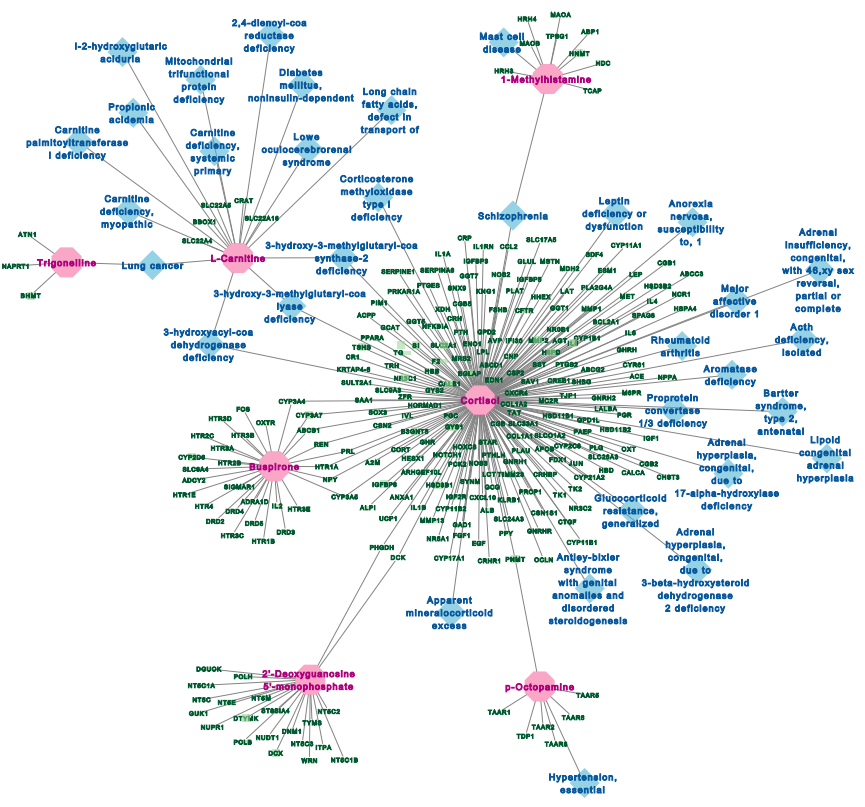


#### Supplementary **Figure 43. Differential protein-metabolite-disease network relationship diagram**

The relationship network diagram illustrates the connections between differential metabolites and proteins in the diabetic nephropathy group compared with the high-risk group. Cortisol, one of the main differential metabolites, is primarily linked with genes such as MMP2, IGF1, CSF2, IL6, and CXCR4, as well as with rheumatoid arthritis.

### Section 2.22. O2PLSDA Analysis


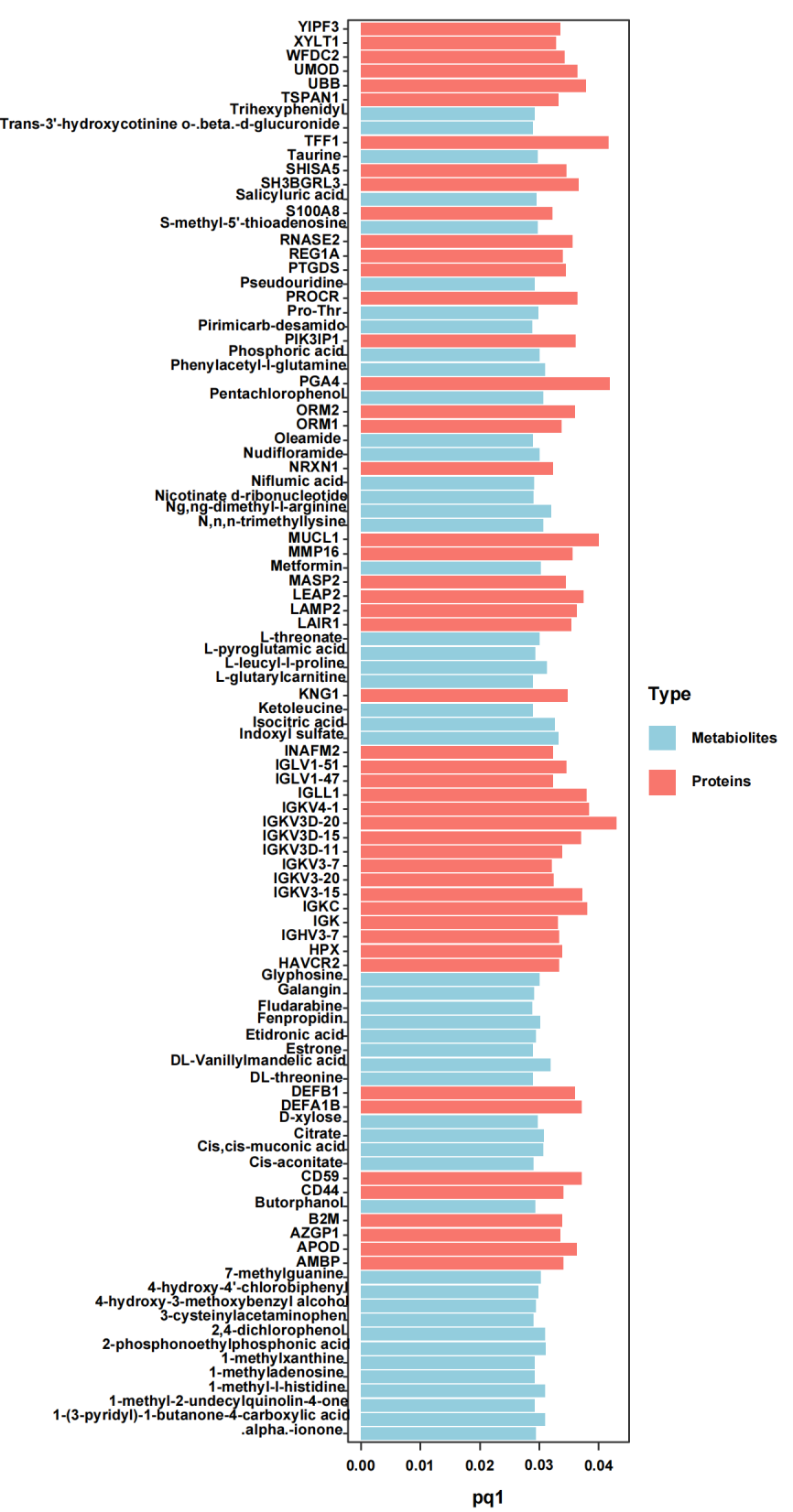


#### Supplementary **Figure 44. O2PLSDA analysis of differential metabolites combined with differential proteins (TOP50)**

By incorporating metabolomics and proteomics into O2PLSDA analysis, researchers can identify common changes in the two groups. The loading diagram visually represents the data correlation and weight between the groups, emphasizing variables like UMOD, TFF1, MMP16, CD59, CD44, and S100A8 with significant correlation and weight.

### Section 2.23. Evaluate and Pre-screen Metabolomic Biomarkers from Multiple Perspectives.


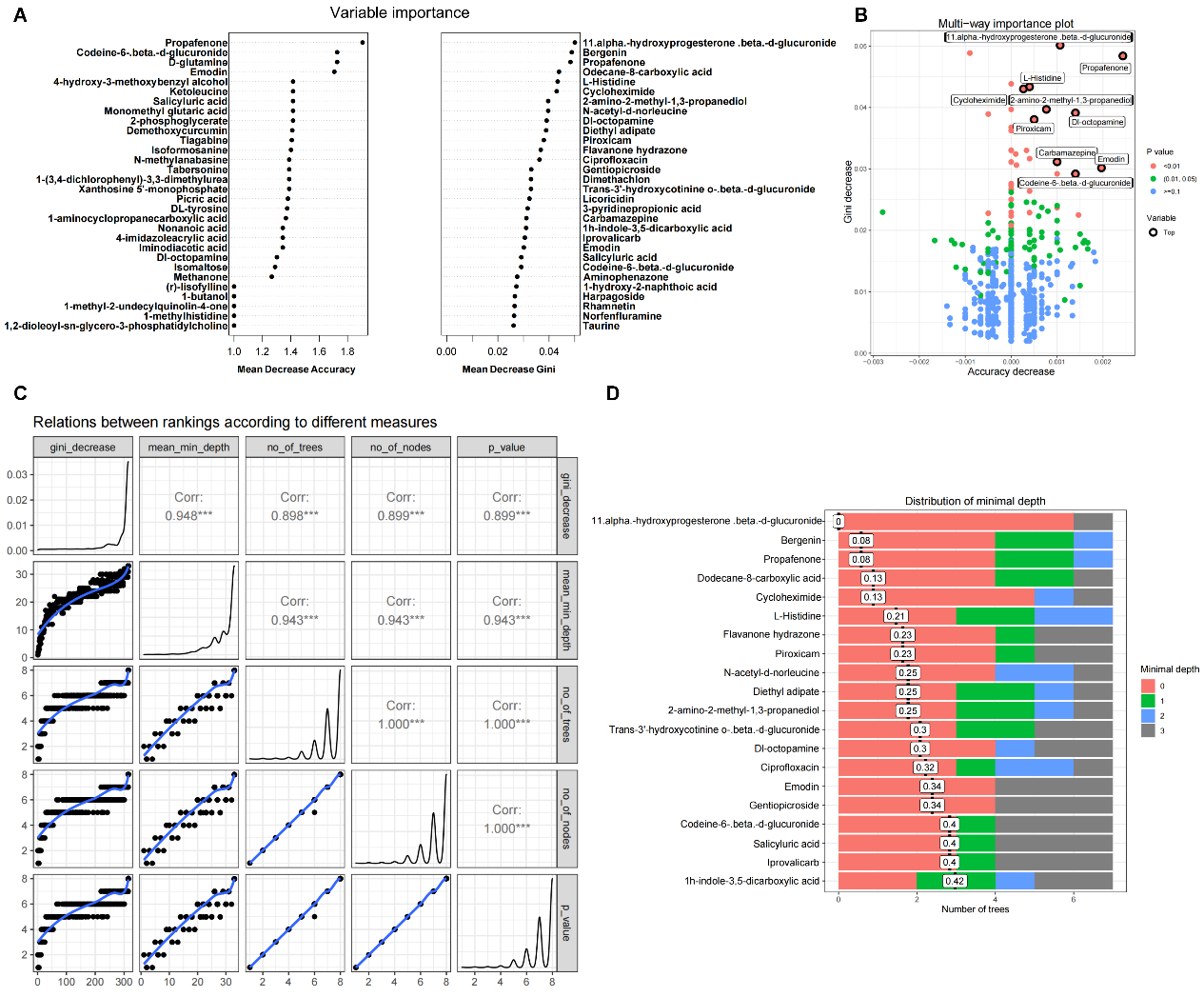


#### Supplementary Figure 45. Using random forest to evaluate and screen metabolomics biomarkers.

A. Random forest method to evaluate variable importance; B. Multivariate importance plot; C. Correlation between different comparison measures; D. Average minimum depth picture

Metabolomics biomarkers were screened using a combination of methods, including random forest for variable screening and decision regression tree for automatic feature selection. Nested resampling was also utilized for additional feature screening. Following this, a comprehensive scoring of metabolites was conducted to determine their importance. Among the top 30 metabolites with high scores were L-glutamic acid dimethyl ester (DLG), Bisphenol E, 3-nitrobenzenesulfonic acid, Trans-3'-hydroxycotinine, and 2-hydroxyatorvastatin lactone.


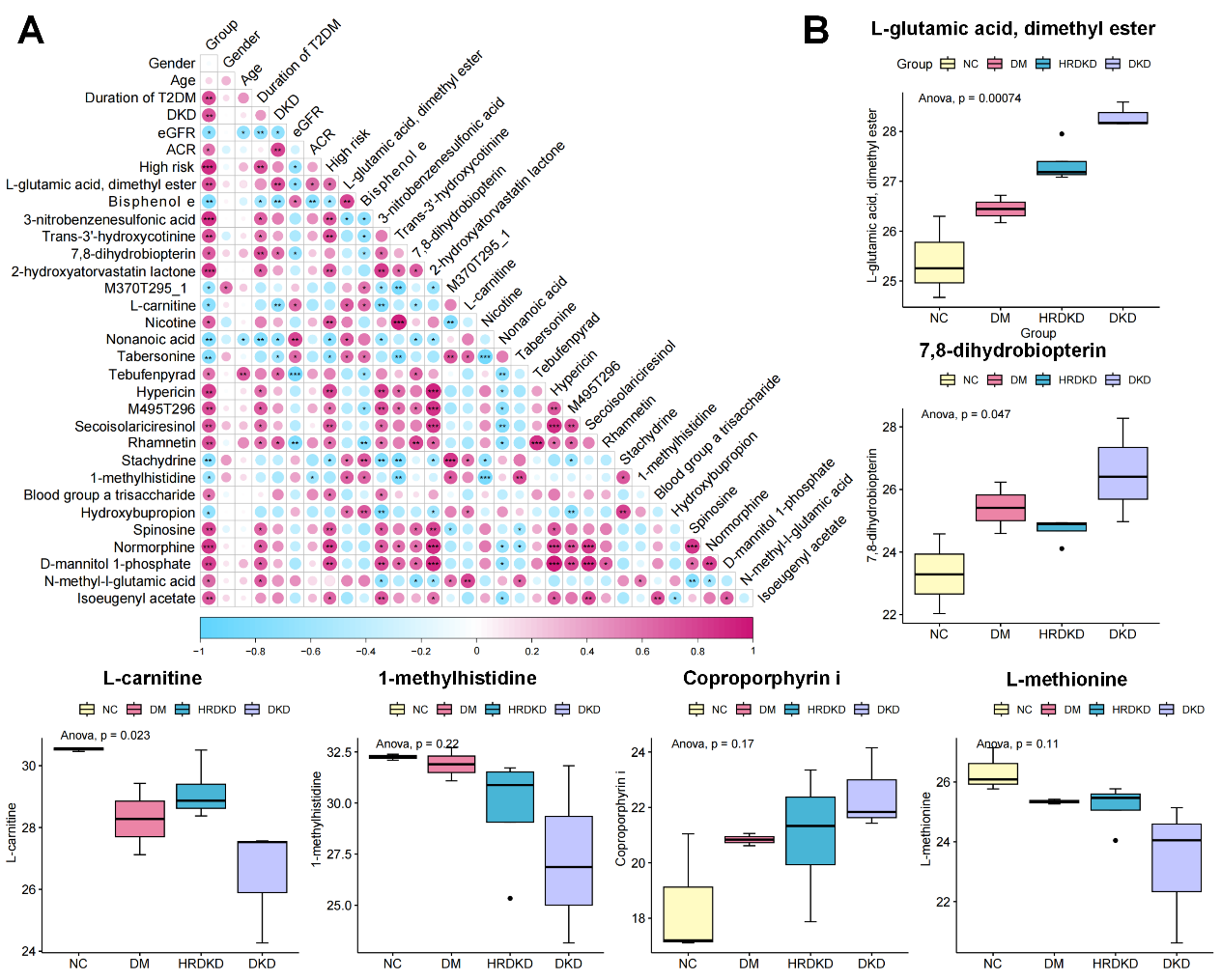


#### Supplementary Figure 46. **The correlation between pre-screened metabolites and expression trends across subgroups in our cohort.**

A. Correlation heatmap between metabolites; B. Correlation heatmap between lipids; C. Box line trend plot (ANOVA analysis)

**Abbreviations:** DKD: diabetic kidney disease; HC: healthy control; DM:diabetes mellitus; HR-DKD: high-risk diabetic kidney disease.

### Section 2.24. Verify the Expression Trend of Metabolic Markers through the Metabolomics Database.


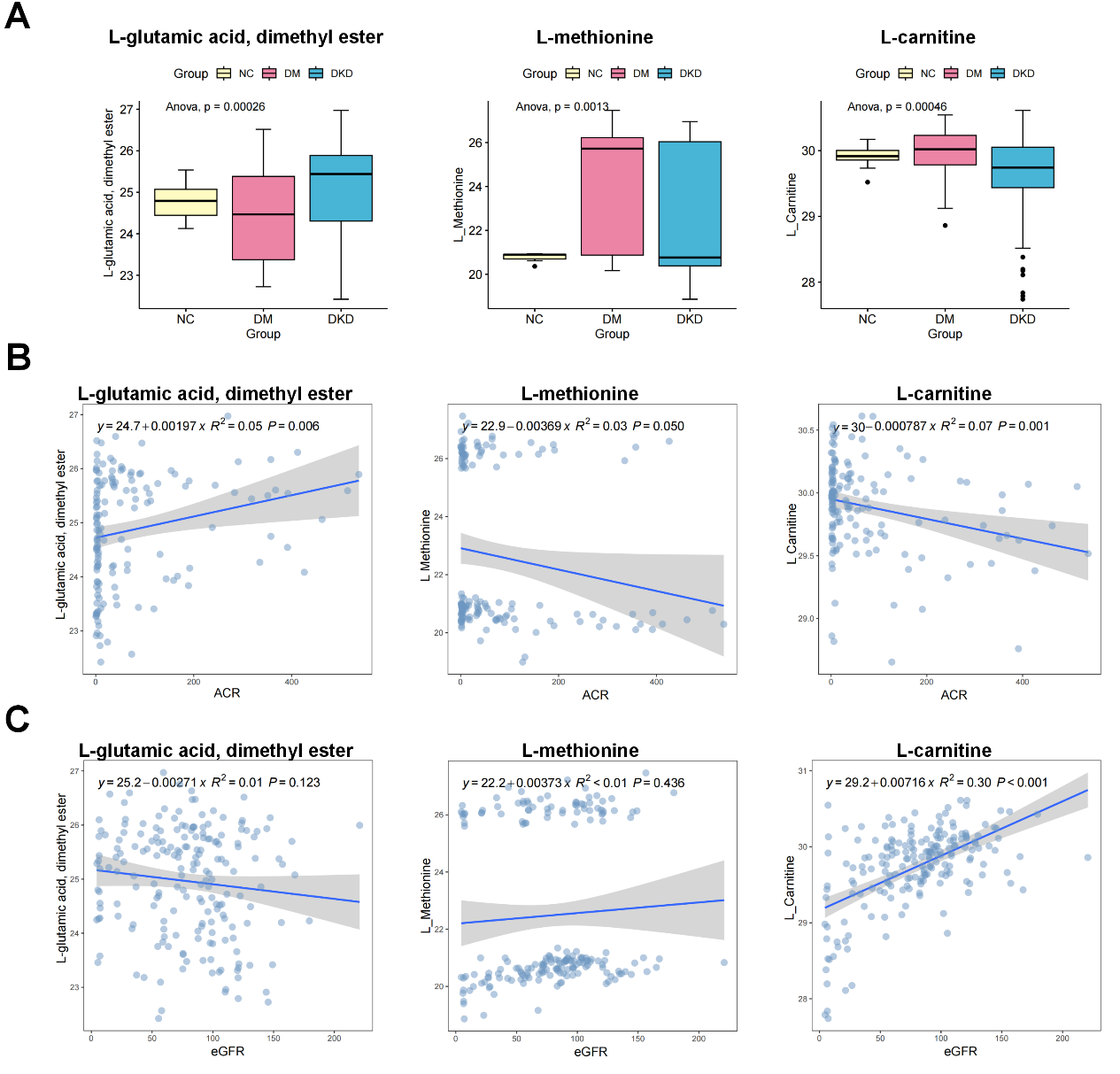


#### Supplementary Figure 47. Expression trend of metabolites screened based on metabolite dataset verification

1. Expression box plot; B. Regression analysis between metabolites and ACR; C. Regression analysis between metabolites and eGFR.

In order to validate the stability of the expression trends of the screened indicators, we analyzed the expression trends of DLG, L-Carnitine, and L-Methionine in external databases and visualized them using box plots. sFigure 44A illustrates the expression boxplot of DLG, L-Carnitine, and L-Methionine in the external metabolite database. All indicators exhibit consistent expression trends with the original data, and the ANOVA analysis shows significant results (P < 0.05). Regression analysis demonstrates that DLG is significantly positively correlated with ACR (P = 0.006), and L-Carnitine is significantly positively correlated with eGFR (P < 0.001).


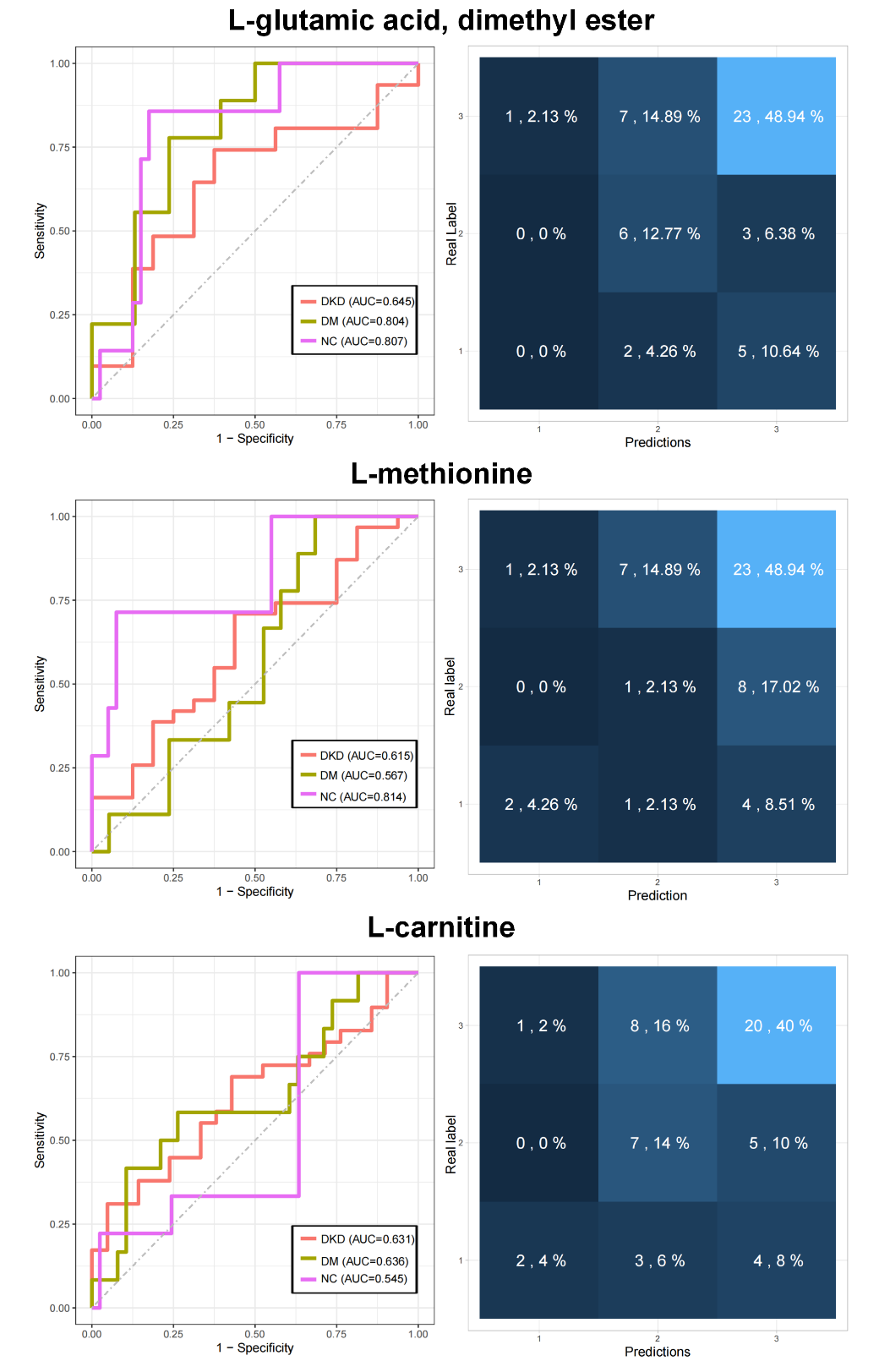


#### Supplementary Figure 48. Evaluate the prediction performance of metabolites in external databases based on the random forest algorithm.

The output includes a confusion matrix and ROC curve, with predictions for three categories: normal control group (NC), diabetes group (DM), and diabetic nephropathy group (DKD).

### Section 2.25. Quality Control Analysis for Lipidomics


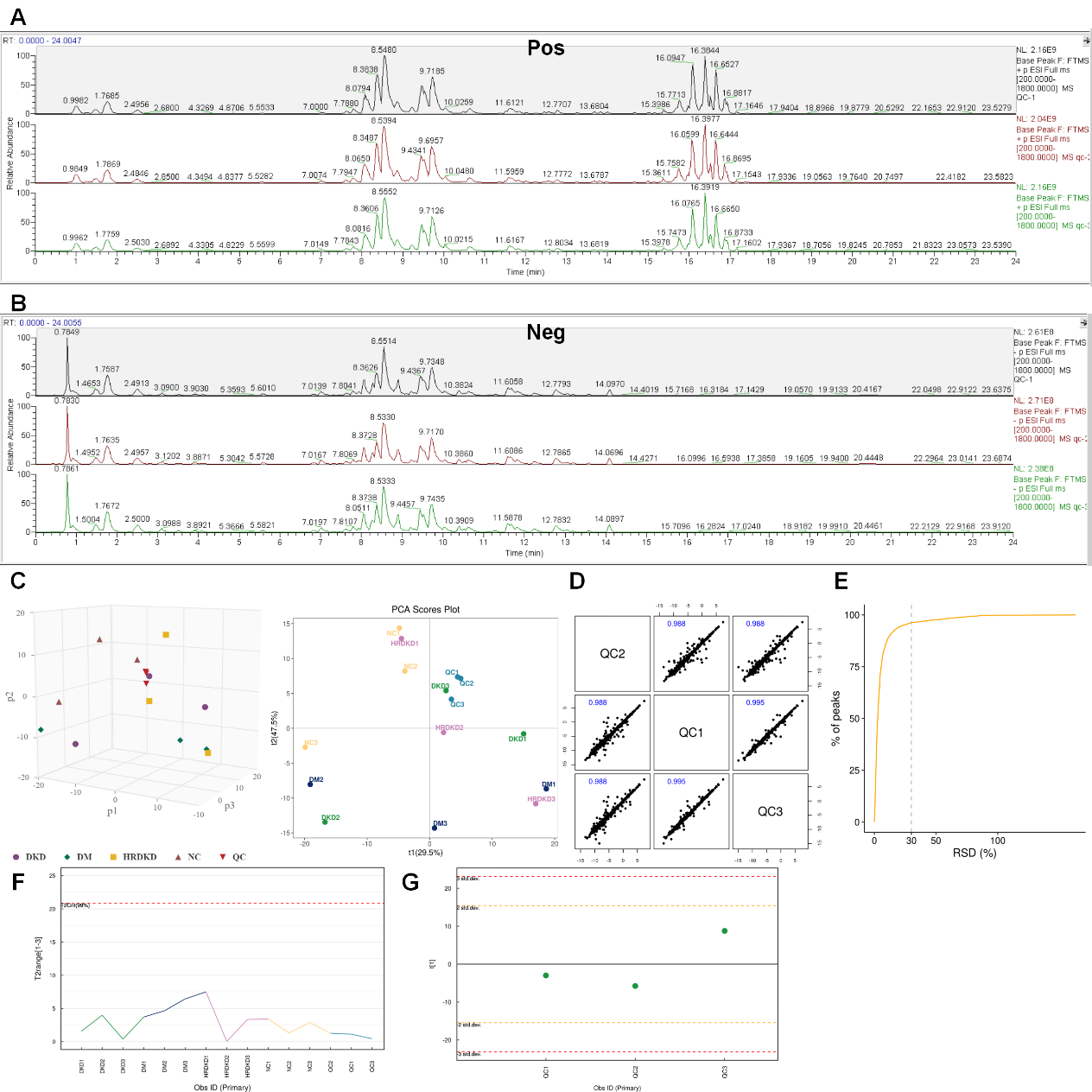


#### Supplementary Figure 49. QC sample evaluation.

1. Positive ion mode QC sample total ion chromatogram overlapping spectrum; B. Negative ion mode QC sample total ion chromatogram overlapping spectrum; C. PCA analysis; D. QC sample correlation spectrum; E. Relative standard deviation of QC samples; F. Hotellings T2 chart of overall sample; G. MCC chart of QC samples;

The grid points in Panel A represent ion peaks (metabolites) extracted from each QC sample, with the horizontal and vertical axes representing the logarithm of the ion peak signal intensity value. In Panel B, the abscissa represents all experimental and QC samples, while the ordinate reflects the confidence interval defined by a red line at the 99% range. Panel C shows each QC sample on the abscissa and standard deviation on the ordinate, with yellow and red lines indicating plus and minus 2 and 3 standard deviation ranges, respectively.

Quality control analysis compared the Base Peak spectrum (BPC) of the QC sample, as shown in the figures. The minimal variation observed was attributed to instrument errors throughout the experiment. The close grouping of the QC samples indicated strong repeatability, with over 80% of Peaks having RSD ≤ 30%, suggesting stable performance of the instrument analysis system. Furthermore, all QC samples fell within the 99% confidence interval, highlighting the excellent repeatability of the experiment. The multivariable control chart displayed for this project revealed that the fluctuations of the QC samples were within plus or minus 3 standard deviations, indicating normal instrument fluctuations and enabling further data analysis.

### Section 2.26. Differential Lipids Analysis between Different Groups.


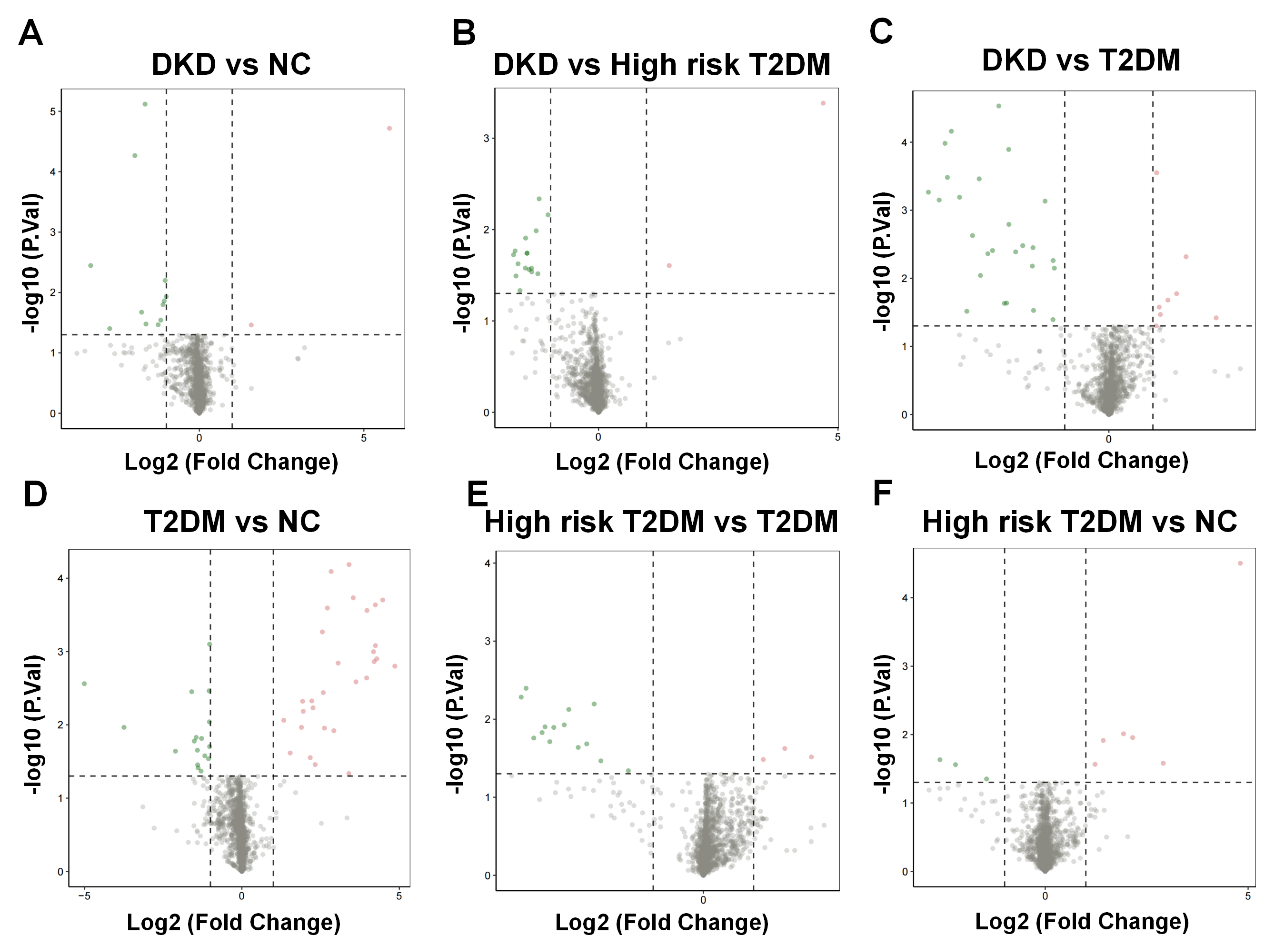


#### Supplementary **Figure 50. Volcano plots**

1. DKD vs HC group; B. DKD vs HR-DKD group; C. DKD vs T2DM group; D. DKD vs HC group; E. DKD vs T2DM group; F. HR-DKD vs HC group.

Differential lipid metabolites are identified using a significance threshold of P < 0.5. These metabolites are then grouped for pairwise comparisons and visualized using a volcano plot.


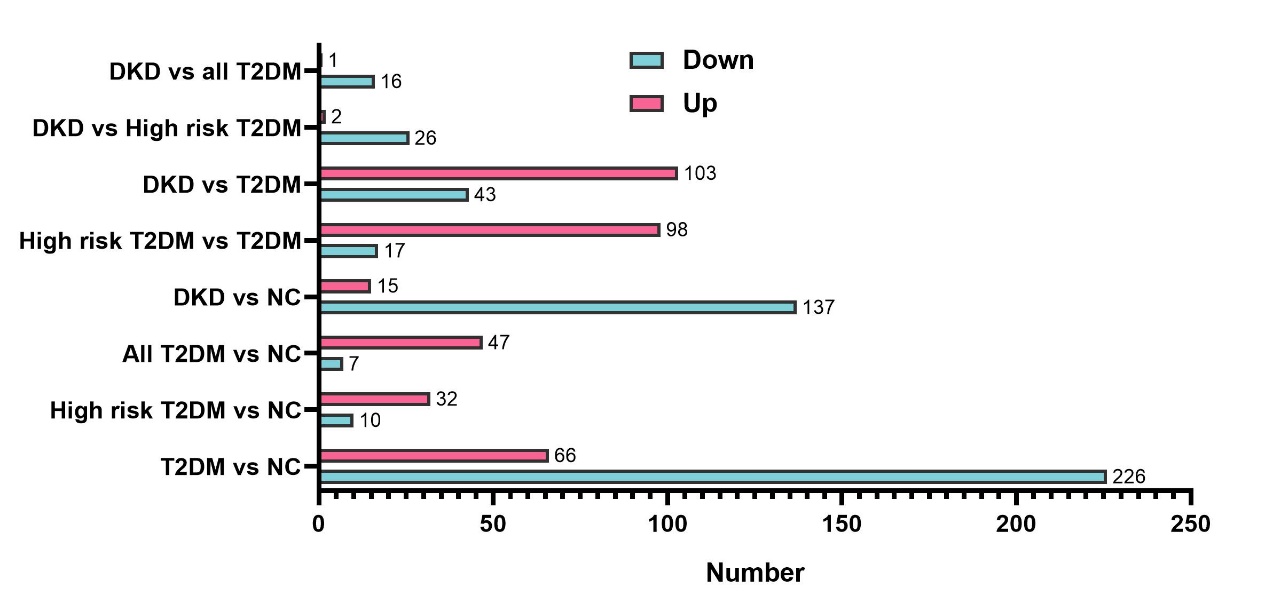


#### Supplementary Figure 51. Statistics on the number of up-regulated and down-regulated differential lipids between different groups

A bar chart was utilized to depict the quantity of differential lipid metabolites, facilitating a more straightforward comparison of expression patterns across various groups. This visualization highlights unique lipid metabolomics distinctions between the initial and advanced stages of the disease. The expression profile reveals that a majority of lipid metabolites were down-regulated in both the early and late stages of disease progression, whereas most lipid metabolites were up-regulated during the intermediate stages of disease advancement.

### Section 2.27. The Overlap of Differential Lipids Identification between Different Groups


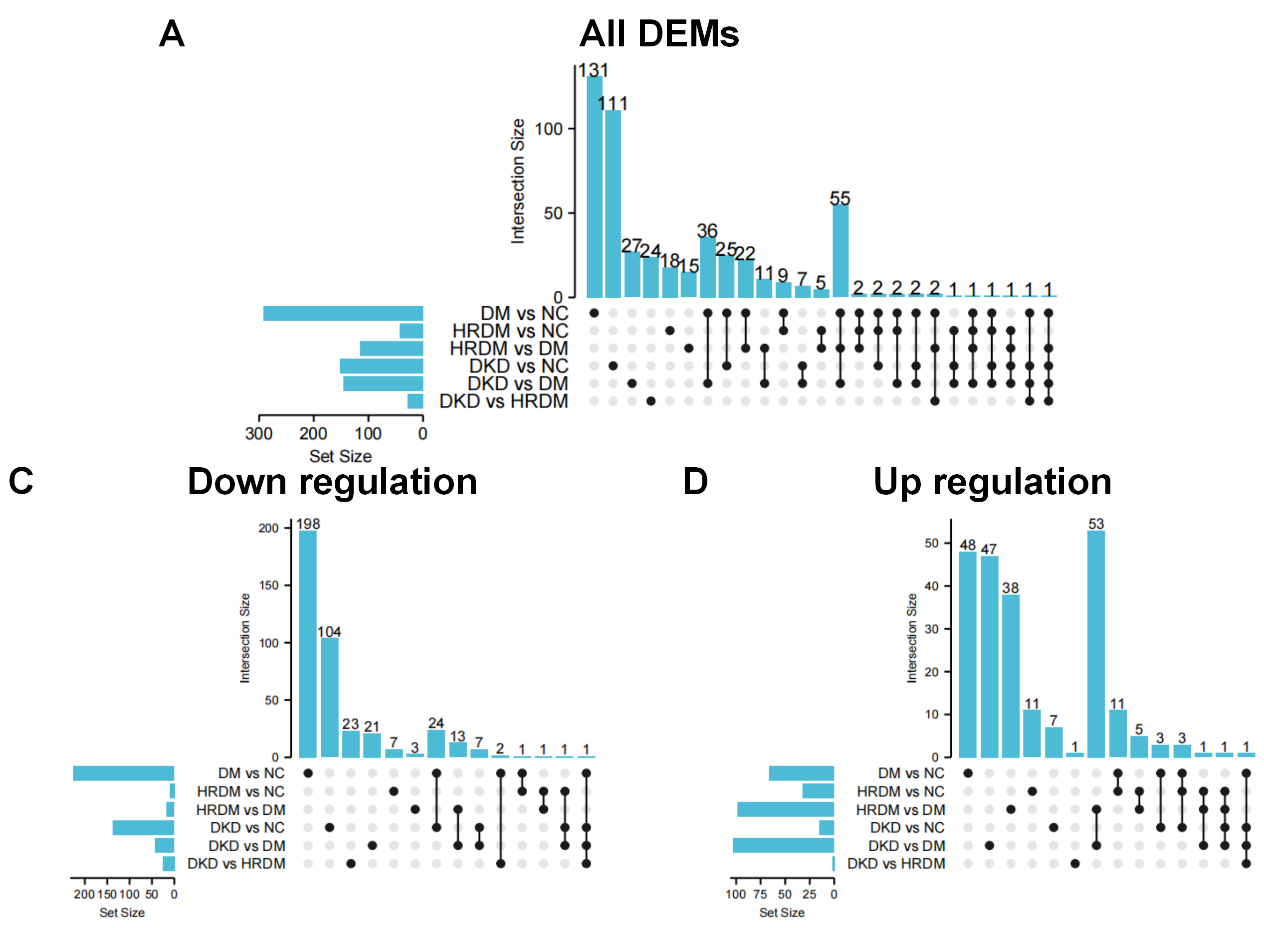


#### Supplementary Figure 52. Overlap and Upset diagram of differential lipid identification between different groups.

1. Overlap of all differential lipid metabolites; B. Overlap of down-regulated lipid metabolites between different groups; C. Between different groups Overlap of up-regulated lipid metabolites.

The study identified overlapping differential lipid metabolites across different groups by analyzing shared compounds and visualizing the results with an Upset plot. Results showed that 3 differential lipid metabolites were common between the DM vs NC, HRDKD vs NC, and DKD vs NC groups (PG(27:0_10:1), PI(18:1_20:3), DG(30:2e)). Furthermore, DKD had 1 unique differential lipid metabolite (PG(27:0_10:1)) not found in the other groups.

### Section 2.2**8.** Lipid Level and Quantity Statistics.


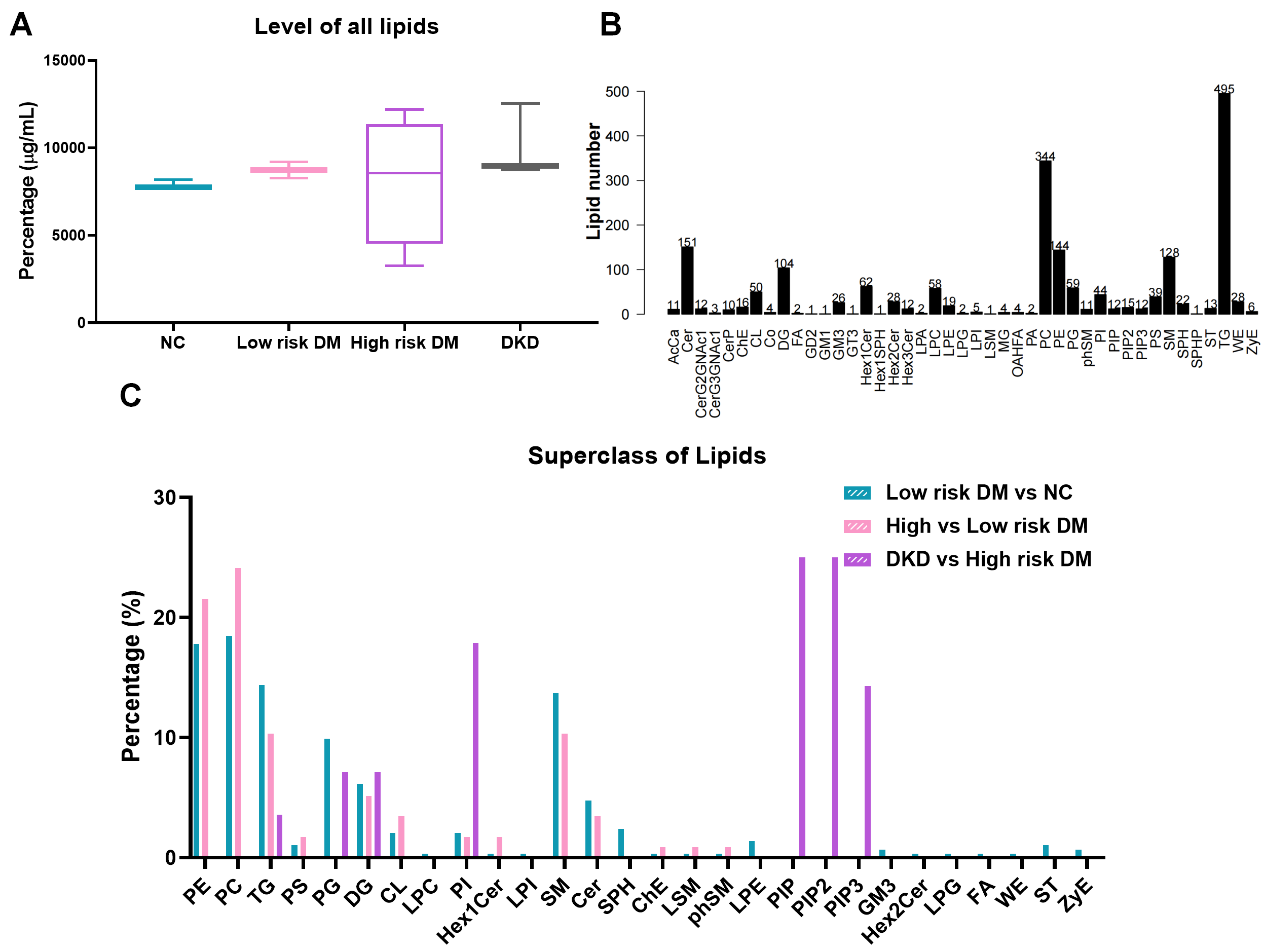


#### Supplementary Figure 53. Statistics of overall lipid levels and differential lipid types.

A. Comparison of overall lipid levels in different groups; B. Statistics of overall lipid molecular subtypes and quantities; C. Percentage of differential lipid subtypes between different groups

LPC: lysophosphatidylcholine; PAF: platelet activating factor; PC: phosphatidylcholine; MePC: methylphosphatidylcholine; LPE: lysophosphatidylethanolamine; LdMePE: lysodimethylphosphatidylethanolamine; PE : Phosphatidylethanolamine; LPS: Lysophosphatidylserine; PS: Phosphatidylserine; LPG: Lysophosphatidylglycerol; PG: Phosphatidylglycerol; LPI: Lysophosphatidylinositol; PI, PIP, PIP2, PIP3: Phosphatidylphosphatidylcholine Alcohol; LPA: lysophosphatidic acid; PA: phosphatidic acid; CL: cardiolipin; SM: sphingomyelin; LSM: lysophosphatidylmyosin; So: sphingomyelin; SoP: sphingomyelin phosphate; SoG1: glucosphingosine; ST: sulfide; Cer: ceramide; CerP: ceramide phosphate; GM3, GM2, GM1, GD1a, GD1b, GD2, GD3, GT1a, GT1b, GT1c, GT2, GT3, GQ1c, GQ1b: ganglioside; MG : Monoglyceride; DG: diglyceride; TG: triglyceride; ChE: cholesterol ester; ZyE: yeast alcohol; Co: coenzyme; WE: waxy lipid; FA: fatty acid.

The lipid levels of various groups are presented, showing a general increasing trend. sFigure 50 illustrates the identified lipid types and their corresponding quantities, with the top 5 types accounting for the largest proportions. Triglyceride (TG), phosphatidylcholine (PC), ceramide (Cer), phosphatidylethanolamine (PE), and sphingomyelin (SM) are ranked in descending order. Upon analyzing the differential lipids, a significant decrease was observed in lipids such as PE, PC, SM, and Cer at the critical stage of DKD onset, while lipids like PI, PIP, PIP2, and PIP3 exhibited a notable increase.

### Section 2.29. Differential Lipid Heat Map and Functional Analysis between Different Groups

**T2DM and HC group**


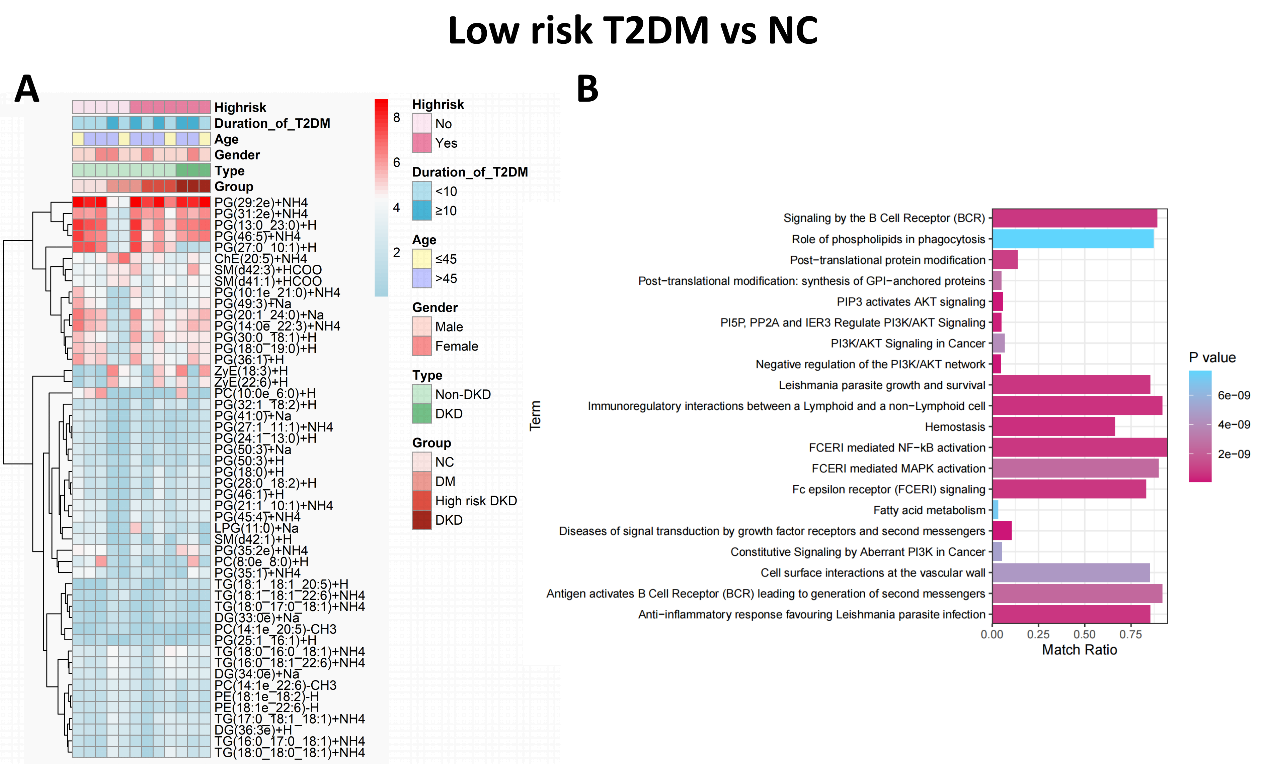


#### Supplementary Figure 54. Differential metabolite and functional enrichment analysis.

1. Heat map of Top 50 differential lipids; B. Reatome enrichment analysis bar chart;

Pathway analysis using Reactome shows enrichment in growth factor receptors, second messenger signal transduction pathways, and specific pathways such as Fc epsilon receptor (FCERI) mediated NF-kB activation induced by antigen, B cell receptor activation leading to second messenger production, and FCERI-mediated MAPK activation.

**HRDKD and T2DM group**


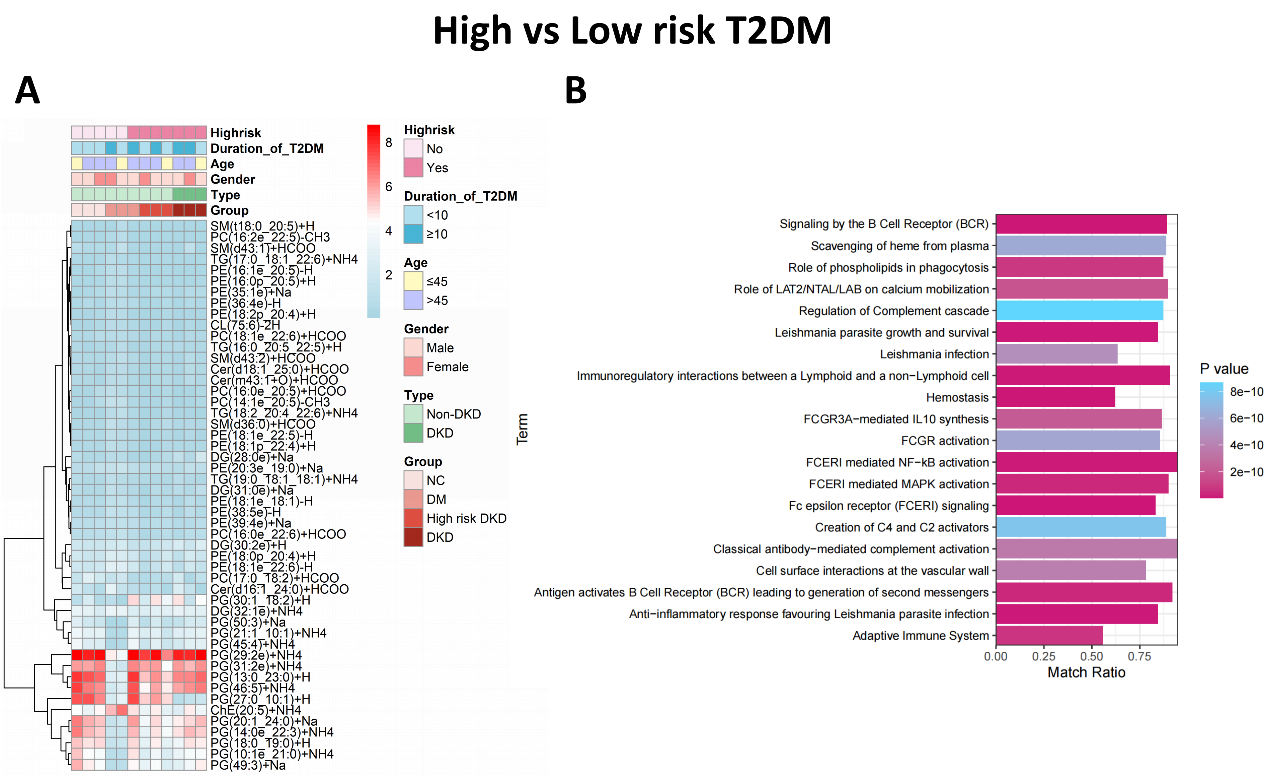


#### Supplementary Figure 55. Differential metabolites and functional enrichment analysis.

1. Heat map of Top 50 differential lipids; B. KEGG enrichment analysis bar chart;

The figure demonstrates that differential lipid types such as PC, PE, and sphingomyelin (SM) are more prevalent in comparison to normal individuals. Additionally, the Reactome analysis reveals significant enrichment in pathways related to FCERI signaling, B cell receptor signaling, immunomodulatory interactions between lymphocytes and non-lymphocytes, phospholipids' role in phagocytosis, FCGR3A-mediated IL10 synthesis, C4 and C2 activators, and regulation of the complement cascade.

**DKD and HRDKD group**


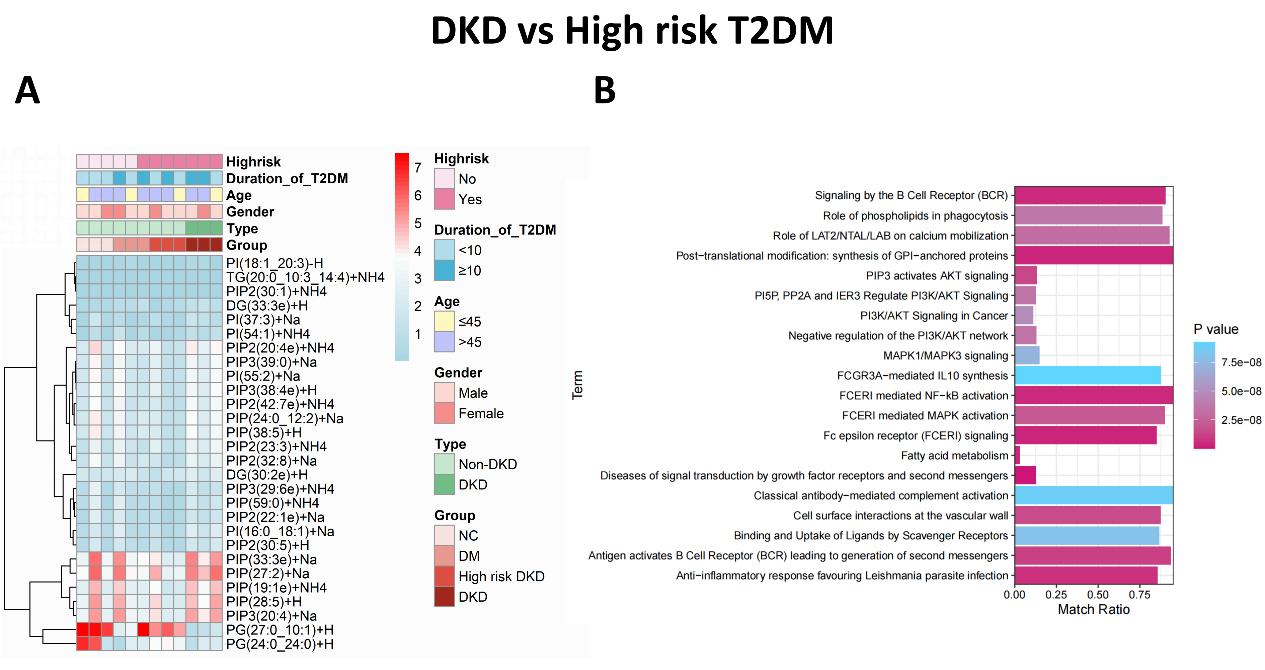


#### Supplementary Figure 56. Differential metabolite and functional enrichment analysis.

1. Heat map of Top 50 differential lipids; B. KEGG enrichment analysis bar chart;

The heat map illustrates the lipid variance between the diabetic nephropathy group and the high-risk group for diabetic nephropathy. The majority of differential lipids identified are phosphatidylinositol (PIP) when compared to individuals without diabetes. Reactome analysis indicates significant enrichment in pathways such as the regulation of PI3K/AKT signaling by PI5P, PP2A, and IER3, FCERI signal transduction, classic antibody-mediated complement activation, interactions on the surface of blood vessel wall cells, and FCGR3A-mediated IL10 synthesis, among others.

### Section 2.30. Mfuzz Temporal Expression Clustering Analysis


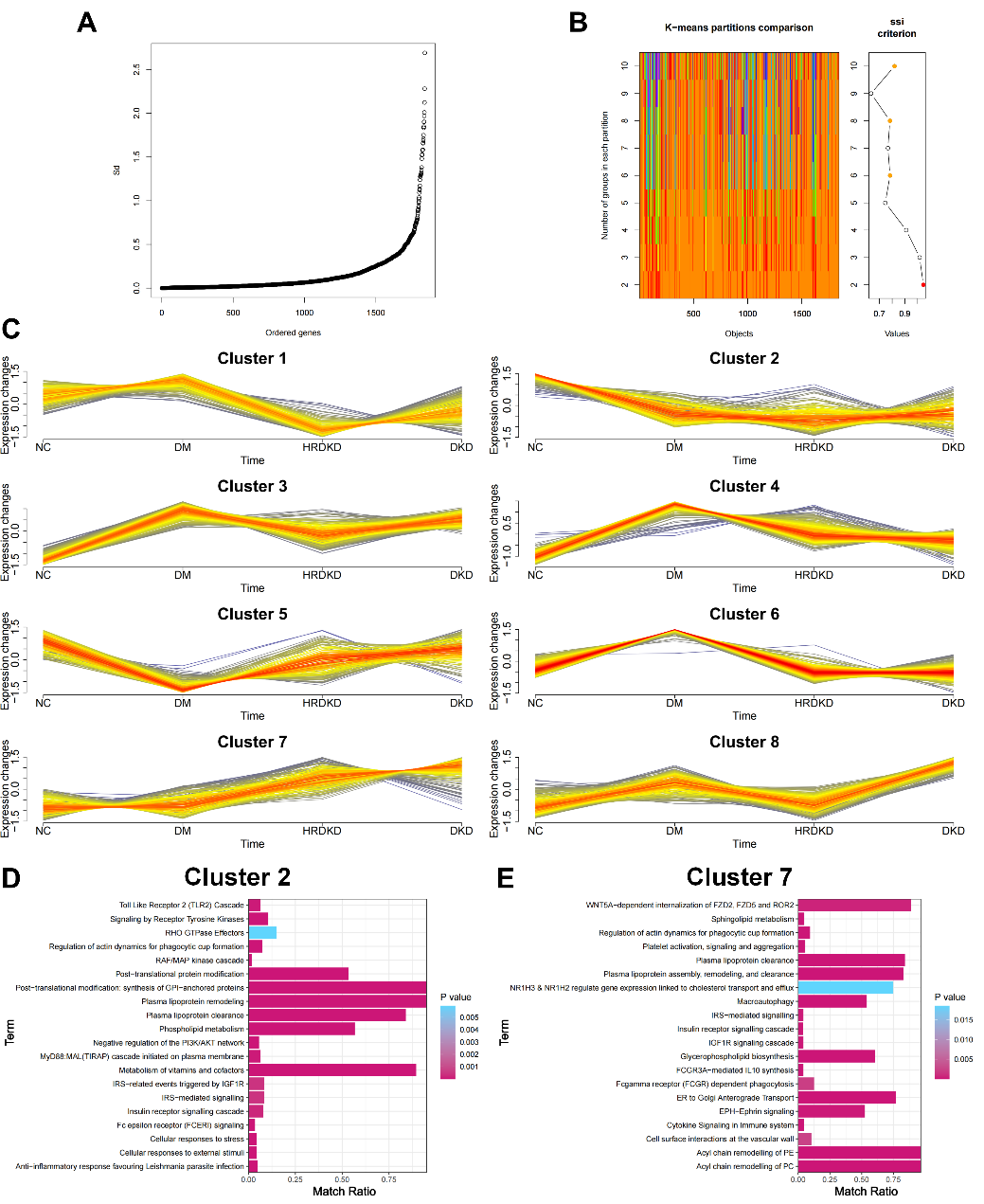


#### Supplementary Figure 57. Mfuzz lipidomics time series expression trend analysis and clustering group classification

A. Standard deviation method to filter proteins; B. Evaluation of the optimal number of clusters by NbClust method; C. Lipid analysis based on fuzzy c-means algorithm Time series clustering analysis of expression (8 clusters); D. KEGG and GO analysis of Cluster 2; E. KEGG and GO analysis of Cluster 7

The study utilized Mfuzz to analyze the temporal dynamic characteristics of blood lipid profiles, clustering lipids with similar patterns to investigate the relationship between lipid changes and different stages of DKD. The NbClust method determined that 8 clusters were appropriate, with Cluster 7 showing a positive correlation with disease progression and Cluster 2 displaying a negative correlation. Further examination of the lipid expression matrix revealed that functional changes in molecular Cluster 2 were primarily associated with phospholipid metabolism, plasma lipoprotein remodeling, cell response to external stimuli, and insulin receptor signaling. On the other hand, molecular Cluster 7 exhibited functional changes related to glycerophospholipid biosynthesis, cytokine signaling, platelet activation, FCGR3A-mediated IL10 synthesis, and other pathways. These findings emphasize the active biological pathways associated with lipid metabolism in the progression of DKD.

### Section 2.31. Weighted Co-expression Network Analysis.


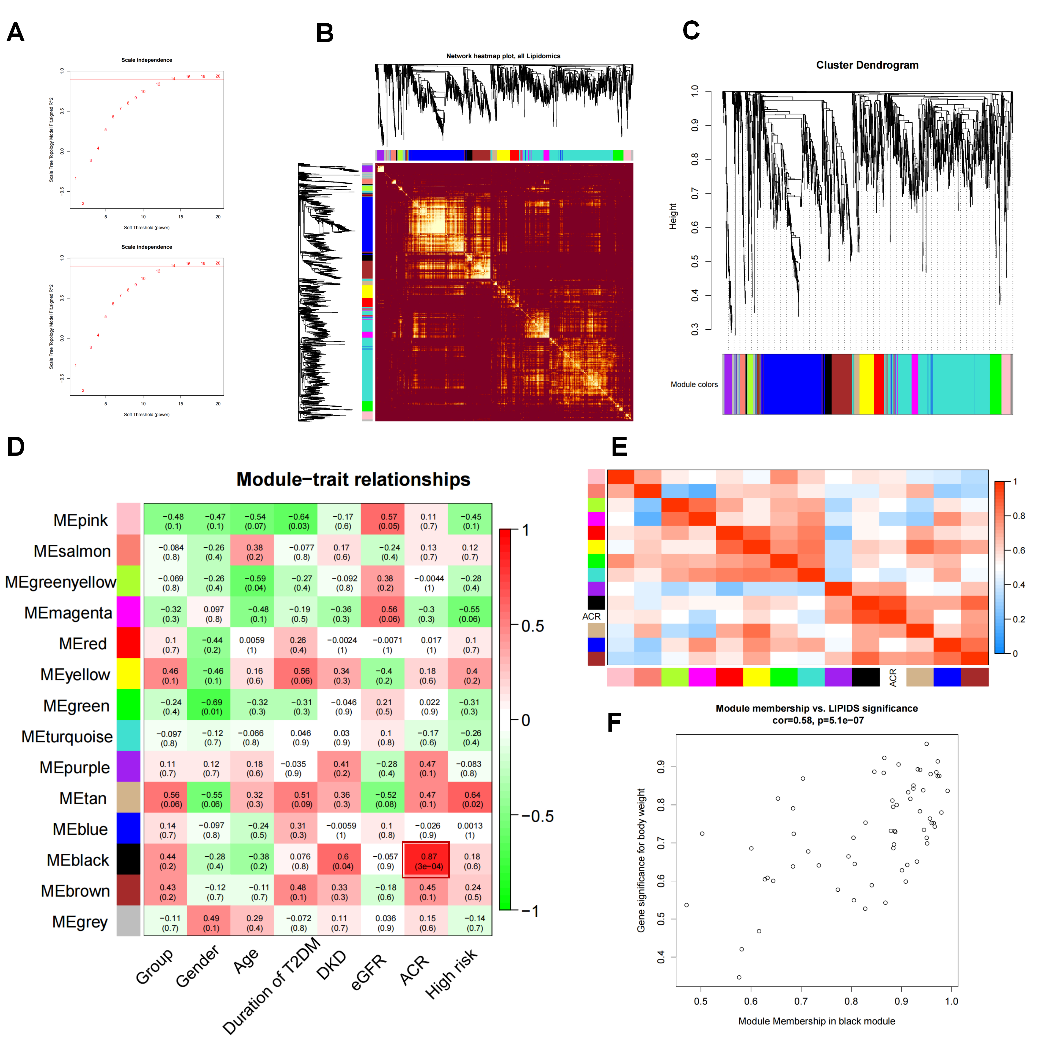


#### Supplementary Figure 58. Weighted gene co-expression network analysis

1. Power diagram to select appropriate thresholds; B. Topological overlap matrix to construct co-expression network; C. Clustering dendrogram; D. Module feature correlation heat map; E. Grouping and Analysis correlation diagram within the module; F. Grouping and black module correlation diagram.

This study involves clustering lipid metabolites into distinct modules using WGCNA analysis to identify core modules associated with key features. The optimal soft threshold of 14 was determined using the pick soft threshold function, transforming the adjacency matrix into a topological overlap matrix for noise reduction. A TOM diagram was then created, followed by the generation of a clustering tree diagram based on the distance matrix (dissTOM) derived from the TOM matrix, resulting in the classification of 1843 lipid metabolites into 15 modules. Examining the correlation map between modules and phenotypic characteristics revealed that the black module shows a significant association with grouping (P value = 5.1e-7).

### Section 2.32. Chain Saturation and Length Analysis.


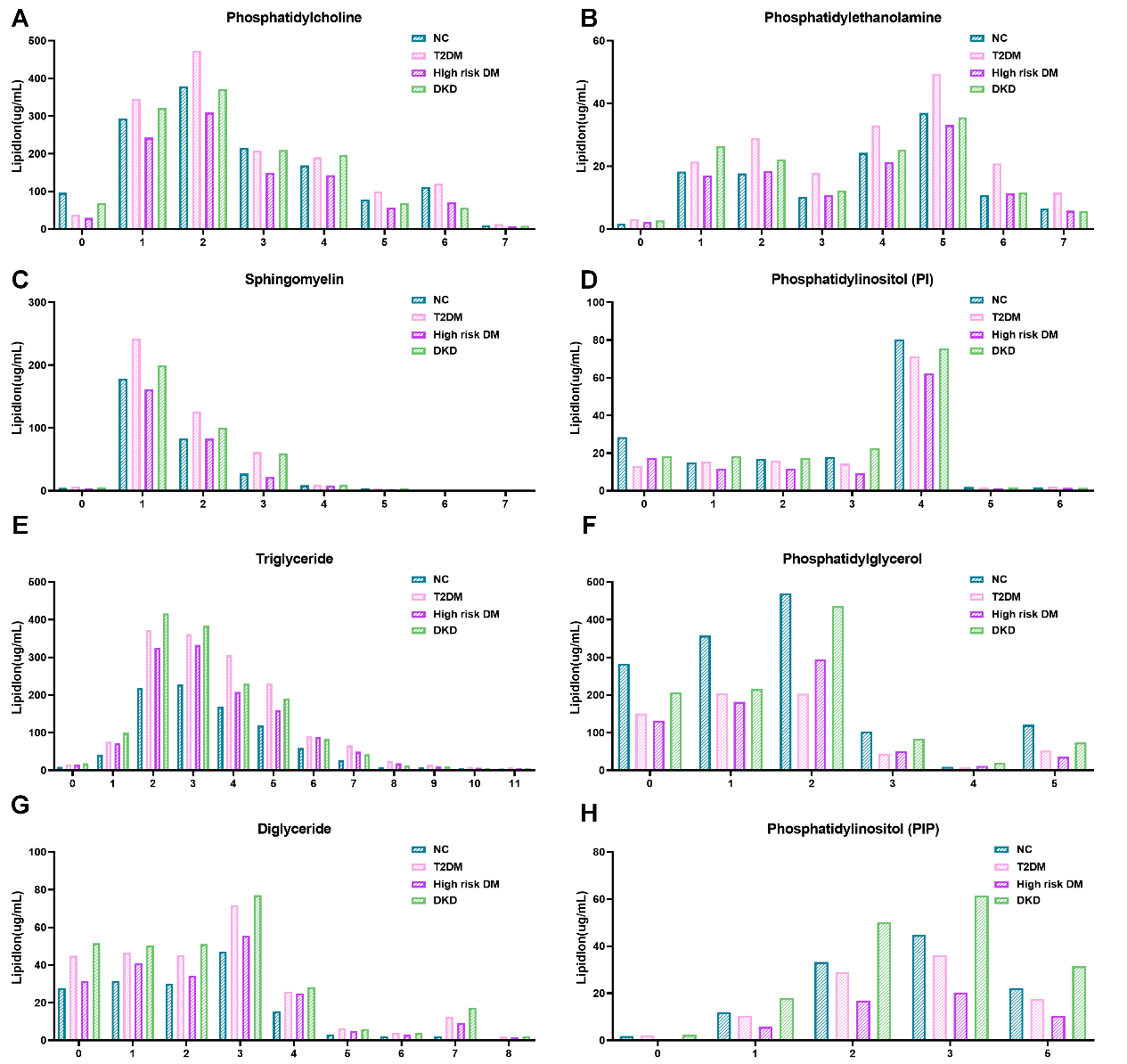


#### Supplementary Figure 59. Chain saturation analysis of the main different lipids

1. Phosphatidylcholine; B. Phosphatidylethanolamine; C. Sphingomyelin; D. Phosphatidylinositol, PI; E. Triglyceride (Triglyceride); F. Phosphatidylglycerol (Phosphatidylglycerol); G. Diglyceride (Diglyceride); H. Phosphatidylinositol (PIP); Note: The abscissa represents the number of unsaturated bonds, and the ordinate represents the The sum of the contents of lipid molecules with the same number of unsaturated bonds.

Chain saturation refers to the total number of double bonds in the fatty acid chains of lipid molecules. The saturation level of lipids can impact cell division and signal transduction by influencing the fluidity of cell membranes. The variations in chain saturation of key lipid types across different groups are illustrated in the figure. In the high-risk diabetic nephropathy group, phosphatidylcholine, phosphatidylethanolamine, sphingomyelin, phospholipids, and diglycerides exhibit distinct differences. Saturated lipid content was lower compared to other groups in this group, while diacylglycerol and triglyceride in the diabetic nephropathy group had higher levels of unsaturated lipids than other groups.


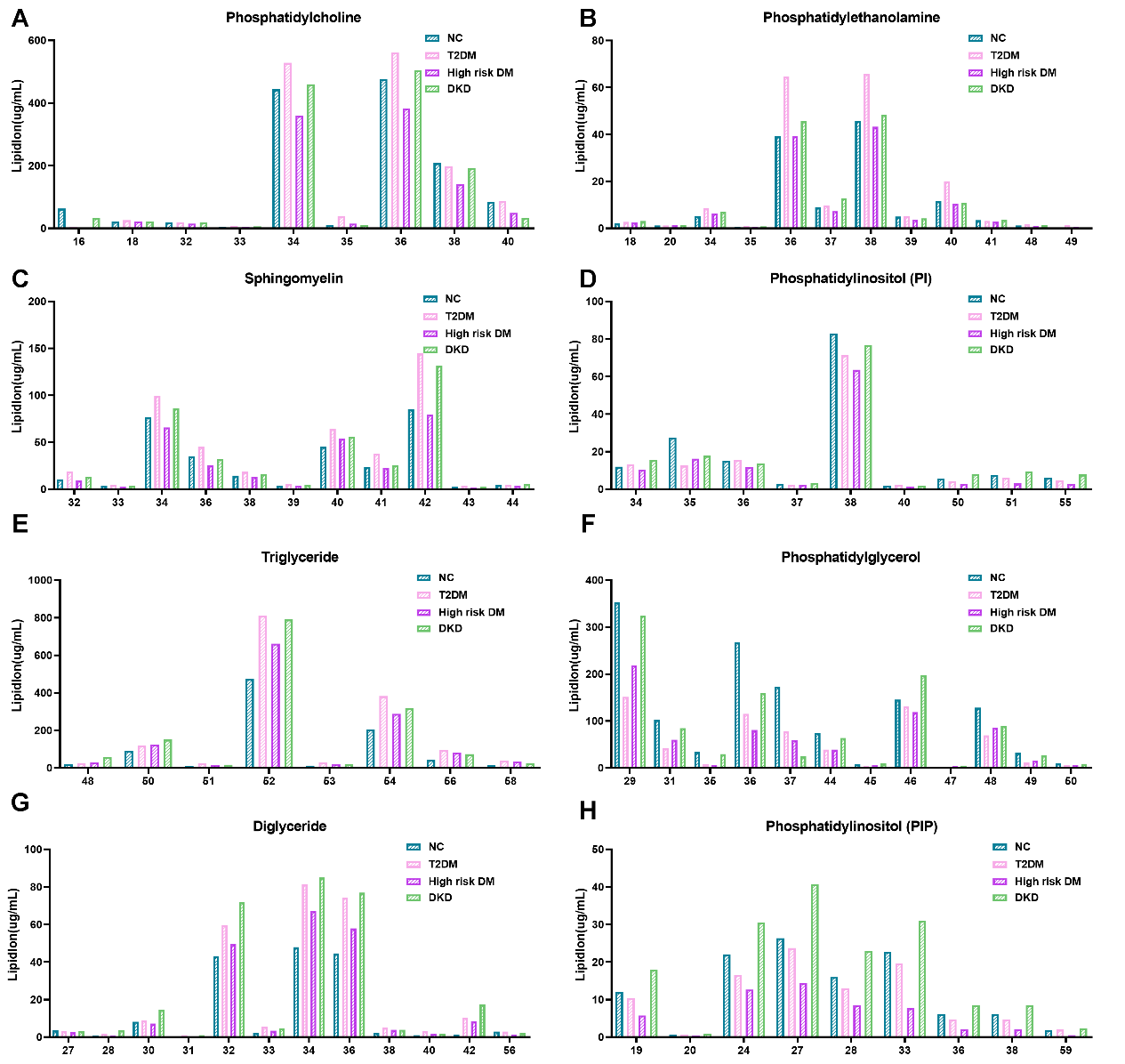


#### Supplementary Figure 60. Chain length analysis of the main different lipids

1. Phosphatidylcholine; B. Phosphatidylethanolamine; C. Sphingomyelin; D. Phosphatidylinositol, PI; E . Triglyceride (Triglyceride); F. Phosphatidylglycerol (Phosphatidylglycerol); G. Diglyceride (Diglyceride); H. Phosphatidylinositol (PIP); Note: The abscissa represents lipid molecules with different carbon chain lengths, and the ordinate The coordinates represent the content of lipid molecules.

The length of a lipid molecule is determined by the total number of carbon atoms in the fatty acid chains. This length influences the thickness and fluidity of the cell membrane, as well as the function of lipid transport proteins and target proteins. Variations in chain length of key differential lipids among different groups are highlighted. In individuals at high risk for diabetic nephropathy, lower levels of phosphatidylcholine (34, 36, 38), phosphatidylethanolamine (36, 37, 38, 40), sphingomyelin (34, 36, 41, 42), phospholipids (34, 36, 38, 50, 51, 55), phosphatidylglycerol (36, 46), and all PIP phospholipids were observed compared to other groups. Conversely, in the type 2 diabetes group, higher levels of phosphatidylcholine (34, 35, 36), phosphatidylethanolamine (34, 36, 38, 40), sphingomyelin (34, 36, 38, 40, 42), and triglycerides (52, 54, 56) were noted compared to other groups.

### Section 2.33. Evaluation and Pre-screening of Lipidomics Biomarkers from Multiple Perspectives.


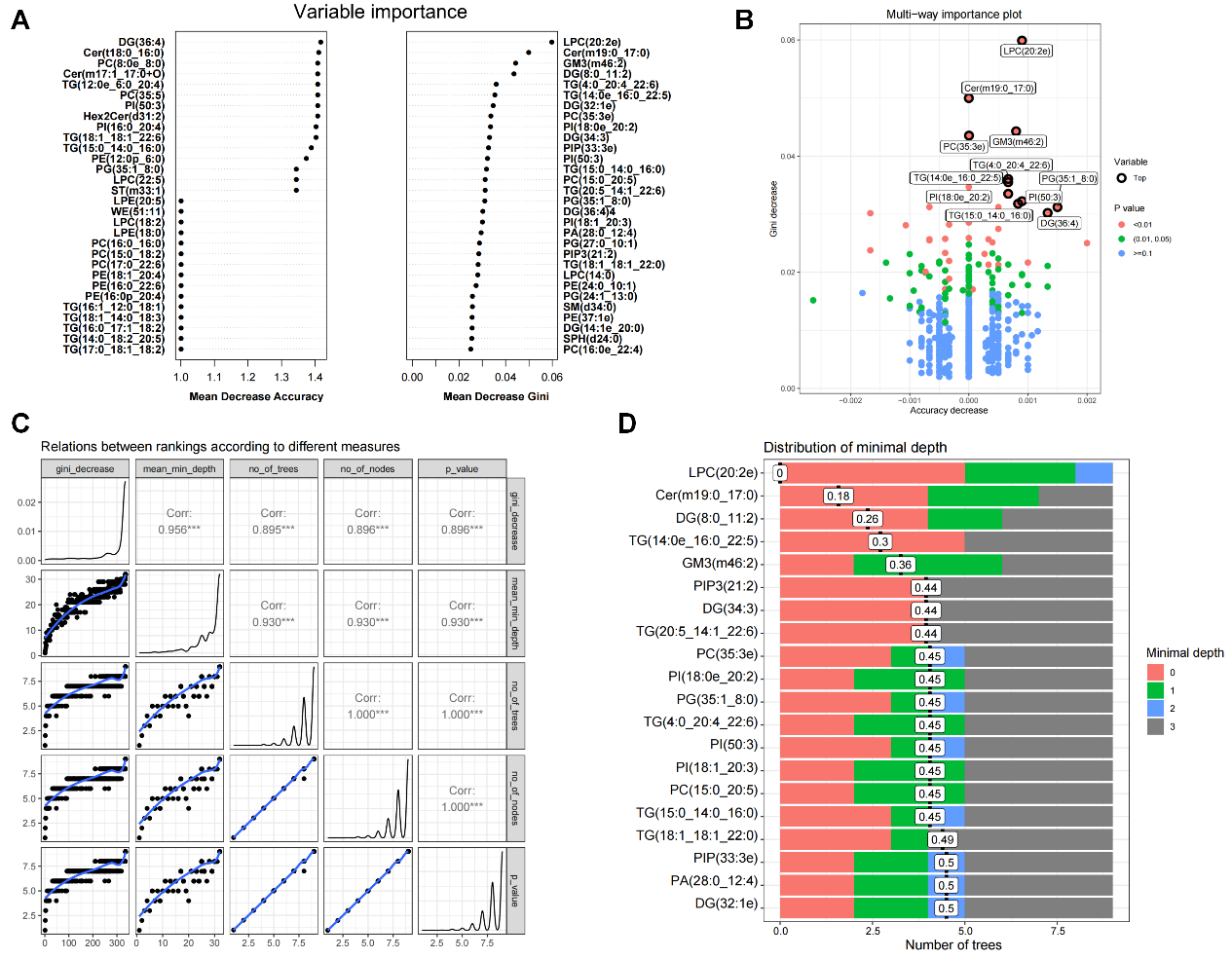


#### Supplementary Figure 61. Use random forest algorithm to evaluate and screen lipidomic features

1. Random forest method to evaluate variable importance; B. Multivariate importance plot; C. Correlation between different comparison measures; D. Average minimum depth picture.

Lipidomics biomarkers were identified through a combination of multi-level and multiple methods. The results presented utilized the random forest method to filter variables and rank features based on variable importance. The top 10 biomarkers identified were LPC (20:2e), Cer (m19:0_17:0), PC (35:3e), GM3 (m46:2), TG (4:0_20:4_22:6), TG (14:0e_16:0_22:5), PG (35:1_8:0), PI (18:0e_20:2), PI (50:3), and TG (15:0_14:0_16:0).


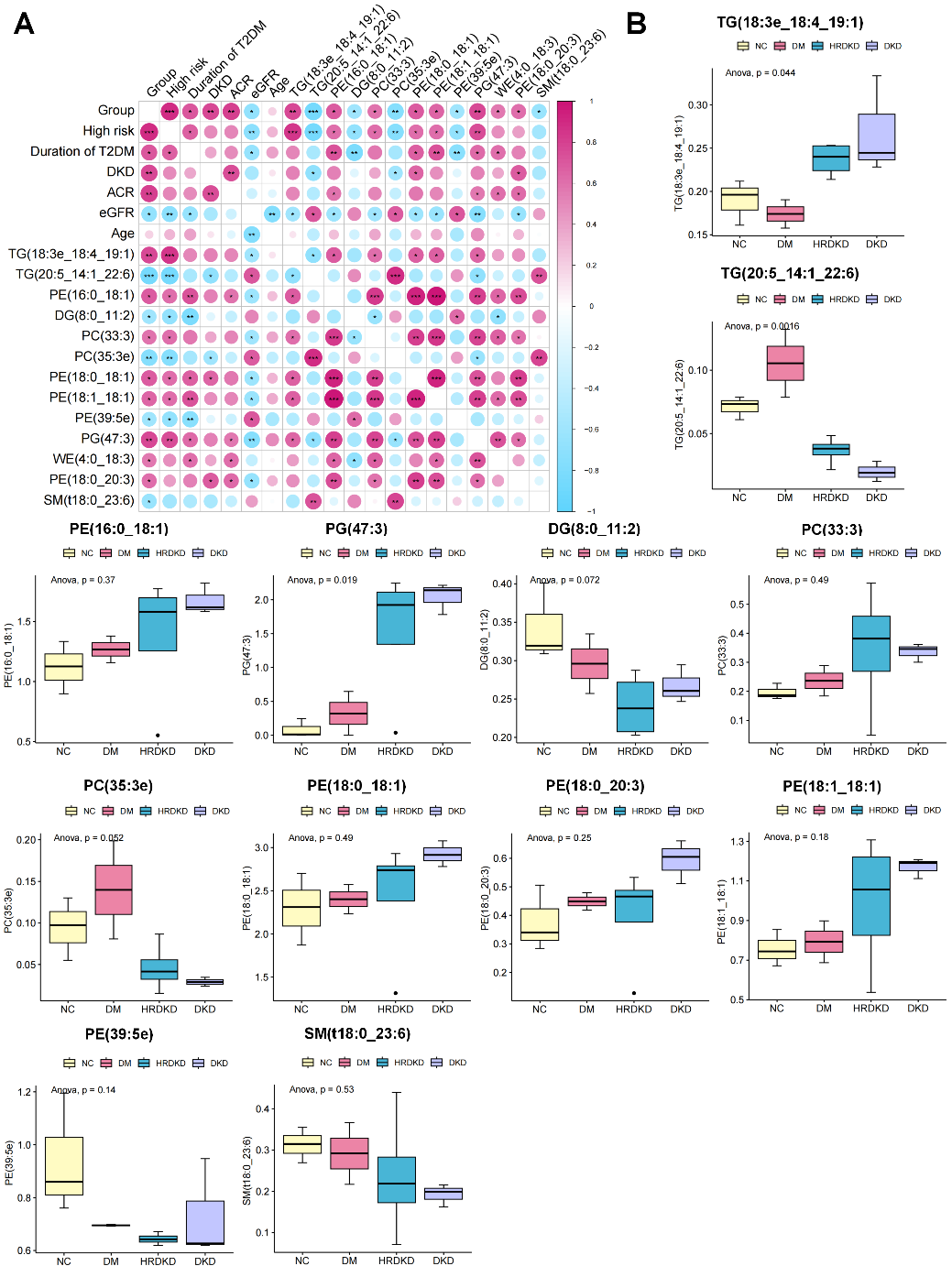


#### **Supplementary Figure 62. The correlation between pre-screened metabolites and expression trends across subgroups in our cohort.**

A. Correlation heatmap between metabolites; B. Correlation heatmap between lipids; C. Box line trend plot (ANOVA analysis)

**Abbreviations:** DKD: diabetic kidney disease; HC: healthy control; DM:diabetes mellitus; HR-DKD: high-risk diabetic kidney disease.

### Section 2.34. Discussion on the biological significance of screening biomarkers

In this study, these protein biomarkers were screened: CST4, CD300LF, MMRN2, and SERPINA1. While no previous studies have reported on the association between these proteins and DKD. CST4, an immune-related gene primarily encoding cystatin D with cysteine protease inhibitory activity, has been detected in saliva and kidney^1^. Its family member CST3, encoding cystatin C, is a recognized marker for renal function^2^. Given the significance of CysC as a DKD marker and the validation in this study, it is reasonable to hypothesize an important link between CST4 and DKD. CD300LF, also known as LMIR3, has been identified as a receptor for sphingomyelin and ceramide, inhibiting mast cell activation^3^ and allergic reactions^4^. MMRN2, an endogenous angiogenesis inhibitor^5^, it anchors CD93 to fibronectin, limiting proteolytic cleavage required for β1 integrin activation and fibrillar formation, thereby reducing endothelial cell adhesion and migration^6^. The role of SERPINA1 in DKD has been documented. SERPINA1, which encodes Alpha-1 antitrypsin, an acute phase inflammatory marker, is closely linked to tumour-infiltrating lymphocytes^7^. Additionally, SERPINA1 has been identified as a potential biomarker for DKD^8^.

Metabolomics can be utilized to provide additional information not captured by proteomics, allowing for a closer examination of individual phenotypic changes^9^. By including patients in the early stages of DKD, metabolomics can identify unique metabolomic characteristics associated with different phase of clinical progression. DLG was identified as a potential metabolomic biomarker of DKD in this study, although there is limited research on its role within the kidney. Existing literature suggests that DLG, a glutamate derivative with proinsulinogenic properties^10^, may serve as a tool for managing T2DM and enhancing the secretory response to GLP-1 proinsulin^11^. Notably, glutamate receptors such as NMDA and mGluR receptors are present in the kidney, with NMDA receptors potentially influencing renal functions, and mGluR receptors playing a role in the development of proteinuria and glomerulosclerosis in knockout mice^12^. Although there is limited information on DLG in the kidney, it is hypothesized that DLG may function as an endogenous L-Glu receptor agonist, activating the receptor and influencing substance transport processes, thereby impacting renal function.

Lipidomics is used to systematically identify complex lipids and investigate lipid-related biomarkers in DKD^13^. In this study, PC was identified as a reliable lipid marker for further validation in the cohort. Previous research has shown a significant increase in the absolute levels of PC in diabetic kidneys^14^. TMAO, a metabolite of PC in the gut microbiota, is known to be pro-atherosclerotic and pro-thrombotic^15^. TMAO has been linked to the onset and progression of DKD, as well as an increased risk of mortality in patients with DKD^16^. Furthermore, the lipid peroxidation product of PC has been found to promote macrophage infiltration in diabetic glomeruli, thereby facilitating the progression of DKD^17^.

**References**

1. Freije J. P., Abrahamson M Fau - Olafsson I., Olafsson I Fau - Velasco G., et al. Structure and expression of the gene encoding cystatin D, a novel human cysteine proteinase inhibitor. 1991;(0021-9258 (Print))

2. Li C., Ma Y., Yang C., et al. Association of Cystatin C Kidney Function Measures With Long-term Deficit-Accumulation Frailty Trajectories and Physical Function Decline. 2022;(2574-3805 (Electronic))

3. Izawa Kumi, Isobe Masamichi, Matsukawa Toshihiro, et al. Sphingomyelin and ceramide are physiological ligands for human LMIR3/CD300f, inhibiting FcεRI-mediated mast cell activation. *Journal of Allergy and Clinical Immunology*. 2014;133(1):270-273.e7. doi:10.1016/j.jaci.2013.08.008

4. Izawa K., Yamanishi Y Fau - Maehara Akie, Maehara A Fau - Takahashi Mariko, et al. The receptor LMIR3 negatively regulates mast cell activation and allergic responses by binding to extracellular ceramide. 2012;(1097-4180 (Electronic))

5. Rao N., Lee Y. F., Ge R. Novel endogenous angiogenesis inhibitors and their therapeutic potential. 2015;(1745-7254 (Electronic))

6. Lugano R., Vemuri K., Yu D., et al. CD93 promotes beta1 integrin activation and fibronectin fibrillogenesis during tumor angiogenesis. *J Clin Invest*. Aug 1 2018;128(8):3280-3297. doi:10.1172/JCI97459

7. Yu Q., Xie T., Zhang Y., et al. Exploration of SERPINA family functions and prognostic value in breast cancer based on transcriptome and in vitro analysis. LID - 10.1002/tox.24079 [doi]. 2023;(1522-7278 (Electronic))

8. Jiang X., Liu X., Qu X., et al. Integration of metabolomics and peptidomics reveals distinct molecular landscape of human diabetic kidney disease. *Theranostics*. 2023;13(10):3188-3203. doi:10.7150/thno.80435

9. Pereira P. R., Carrageta D. F., Oliveira P. F., et al. Metabolomics as a tool for the early diagnosis and prognosis of diabetic kidney disease. *Med Res Rev*. Jul 2022;42(4):1518-1544. doi:10.1002/med.21883

10. Sener A., Conget I Fau - Rasschaert J., Rasschaert J Fau - Leclercq-Meyer V., et al. Insulinotropic action of glutamic acid dimethyl ester. 1994;(0002-9513 (Print))

11. Cancelas J., Villanueva-Peñacarrillo Ml Fau - Valverde I., Valverde I Fau - Malaisse W. J., Malaisse W. J. Potentiation and prolongation of the insulinotropic action of glucagon-like peptide 1 by methyl pyruvate or dimethyl ester of L-glutamic acid in a type 2 diabetes animal model. 2001;(1355-008X (Print))

12. Valdivielso J. M., Eritja À Auid-Orcid, Caus M. Auid-Orcid, Bozic M. Auid-Orcid. Glutamate-Gated NMDA Receptors: Insights into the Function and Signaling in the Kidney. LID - 10.3390/biom10071051 [doi] LID - 1051. 2020;(2218-273X (Electronic))

13. Baek J., He C., Afshinnia F., Michailidis G., Pennathur S. Lipidomic approaches to dissect dysregulated lipid metabolism in kidney disease. *Nat Rev Nephrol*. Jan 2022;18(1):38-55. doi:10.1038/s41581-021-00488-2

14. Pang L. Q., Liang Ql Fau - Wang Yi-Ming, Wang Ym Fau - Ping Li, Ping L Fau - Luo Guo-An, Luo G. A. Simultaneous determination and quantification of seven major phospholipid classes in human blood using normal-phase liquid chromatography coupled with electrospray mass spectrometry and the application in diabetes nephropathy. 2008;(1570-0232 (Print))

15. Li X. S., Obeid S., Klingenberg R., et al. Gut microbiota-dependent trimethylamine N-oxide in acute coronary syndromes: a prognostic marker for incident cardiovascular events beyond traditional risk factors. 2017;(1522-9645 (Electronic))

16. Sapa H., Gutiérrez O. M., Shlipak M. G., et al. Association of Uremic Solutes With Cardiovascular Death in Diabetic Kidney Disease. 2022;(1523-6838 (Electronic))

17. Uesugi N., Sakata N Fau - Horiuchi S., Horiuchi S Fau - Nagai R., et al. Glycoxidation-modified macrophages and lipid peroxidation products are associated with the progression of human diabetic nephropathy. 2001;(1523-6838 (Electronic))
